# Conserved hepatic RNA signatures across multiple cohorts reveal novel mechanistic clues for advanced fibrosis in human MASLD

**DOI:** 10.1097/HC9.0000000000000991

**Published:** 2026-09-16

**Authors:** Jessica L. Maiers, Tiebing Liang, Tingbo Guo, Sha Cao, Prakash Ramachandran, Teresa J. Raba, Stefano Romeo, Oveis Jamialahmadi, Arman Shahrisa Etu, Naga Chalasani

**Affiliations:** 1 Department of Medicine, Indiana University School of Medicine, Indianapolis, Indiana, USA; 2 Department of Biostatistics and Health Data Science, Indiana University School of Medicine, Indianapolis, Indiana, USA; 3 Department of Biomedical Engineering, Oregon Health & Science University, Portland, Oregon, USA; 4 Centre for Inflammation Research, Institute for Regeneration and Repair, The University of Edinburgh, Edinburgh, UK; 5 Department of Medicine (H7), Center for Molecular Medicine (CeRM), Karolinska Institutet, Huddinge, Stockholm, Sweden; 6 Department of Endocrinology, Karolinska University Hospital, Huddinge, Stockholm, Sweden; 7 Department of Molecular and Clinical Medicine, Institute of Medicine, University of Gothenburg, Gothenburg, Sweden; 8 Department of Cardiology, Sahlgrenska University Hospital, Gothenburg, Sweden; 9 Clinical Nutrition Unit, Department of Medical and Surgical Sciences, Magna Graecia University, Catanzaro, Italy; 10 Translational Research for Diabetes UMR 1190, University of Lille, Lille, France

**Keywords:** cell–cell interactions, deconvolution, MASLD, meta-analysis, single-cell genomics

## Abstract

**Background:**

Fibrosis development in patients with metabolic dysfunction–associated steatotic liver disease (MASLD) is a key indicator of disease progression and clinical outcome. Bulk and single-cell transcriptomics on human tissue have advanced understanding of fibrosis progression, but interstudy heterogeneity and limited sample size hinder the identification of consistent and targetable fibrogenic mechanisms.

**Methods:**

To identify conserved fibrogenic mechanisms, we performed a comprehensive meta-analysis of hepatic transcriptomic data with fibrosis stage characterized from ~1000 patients with MASLD.

**Results:**

Our meta-analysis revealed 846 differentially regulated genes associated with fibrosis progression (F3–4 vs. F0–1). Pathway analysis showed that these genes are involved in matrix organization (*THBS2*, *ADAMTSL2*), inflammation (*CXCL6*, *CCL19*), solute transport (*SLC13A5*, *SLC16A10*), and metabolism (*AADAT*, *GRAMD1B*). scRNA-seq-based deconvolution revealed increased proportions of immune (CD4+ T cells), endothelial (HA endo cells), and mesenchymal (myofibroblasts) cell types, and loss of LSECs in patients with advanced fibrosis in the meta-analysis. Finally, analysis of ligand–receptor pairs identified putative cell–matrix interactions (MMP7–CDH6), cell signaling (PDGFD–PDGFRA), and immune cell interactions (ANXA1–FPR1) associated with fibrosis conserved across multiple datasets and enriched in patients with fibrosis.

**Conclusions:**

We identified conserved transcriptomic changes and gene networks in patients with advanced fibrosis, as well as putative ligand–receptor interactions that facilitate cell–cell interactions that drive fibrosis. These findings will inform mechanistic studies and the development of anti-fibrotic therapies.

## Graphical abstract

**Figure:**
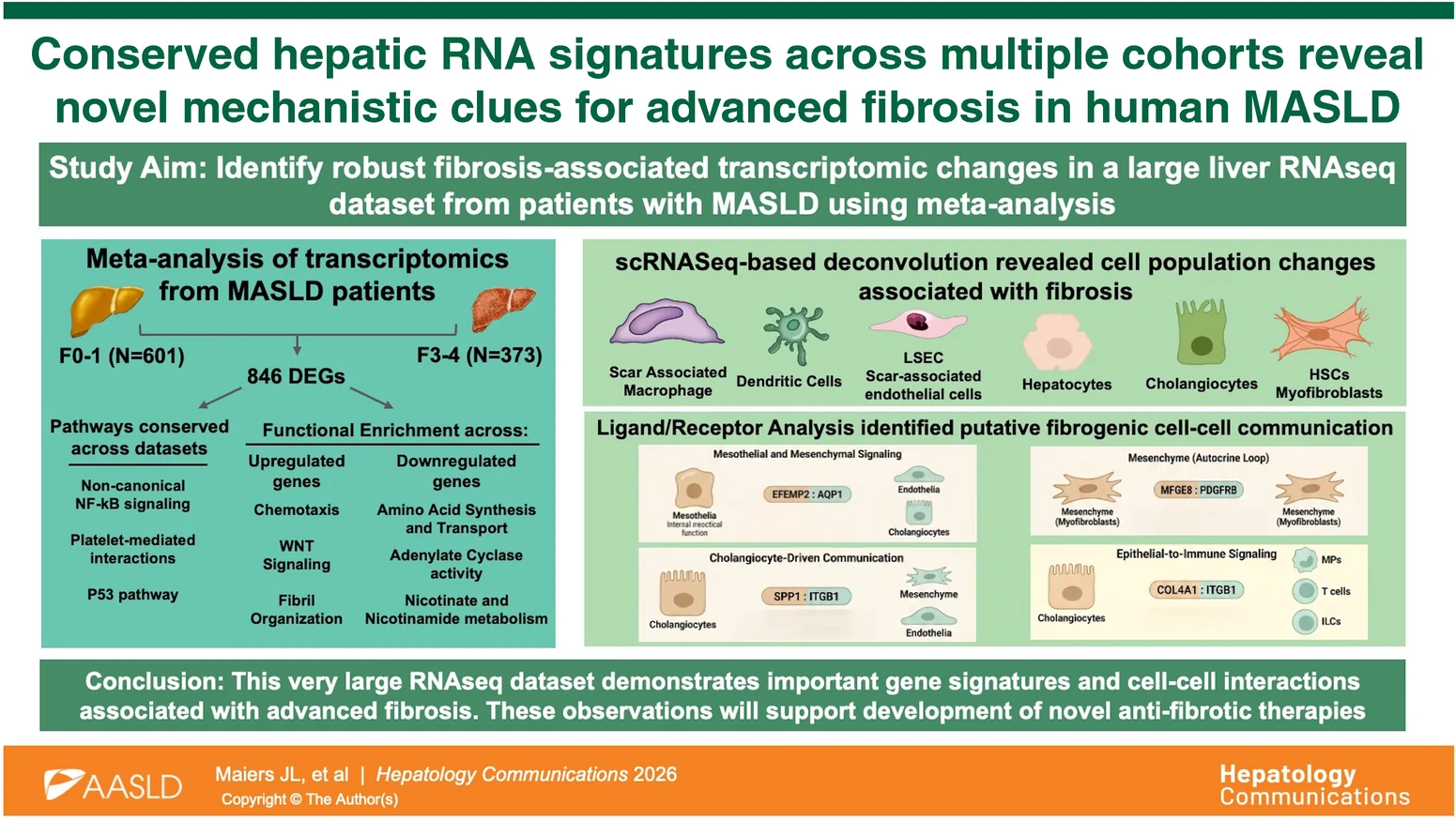

## INTRODUCTION

Metabolic dysfunction–associated steatotic liver disease (MASLD), previously referred to as non-alcoholic fatty liver disease (NAFLD),^1^^,^^2^ impacts almost 40% of adults and 10% of children globally.^3^^,^^4^ In a subset of patients, MASLD progresses to metabolic dysfunction–associated steatohepatitis (MASH), which can lead to cirrhosis and hepatocellular carcinoma (HCC). Necroinflammation and fibrosis are important intermediary steps that can lead to cirrhosis and HCC, and no approved therapies directly target fibrosis. The liver consists of heterogeneous cell populations that function in connected ways, responding through intercellular and intracellular communication under physiological conditions, and adapting to pathological stimuli. Hepatic inflammation and fibrosis involve alterations in hepatic parenchymal and non-parenchymal cell (NPC) populations; however, the link between cellular phenotypic changes and underlying regulatory mechanisms remains incompletely understood.

Multiple studies have published hepatic gene profiles in MASLD, identifying gene sets predicting disease progression, disease severity, or association of select genes with patients at high risk of developing fibrosis. These studies led to key findings, including how inflammation and the extracellular matrix (ECM) drive disease development,^5^ changes in lipid metabolism associate with the progression from MASLD to MASH, and signaling through select metabolic and cell proliferation pathways correlate with fibrosis stage severity.^6^ These studies also identify important pathways involving lipid metabolism, immunomodulation, ECM remodeling, and cell cycle control in MASLD progression.^7^^,^^8^ Unfortunately, direct comparison of these studies is limited by cohort differences in disease stage, patient demographics, limited patient number, and the focus of categorical analysis for each study. Some studies specifically compared patients with or without steatosis, whereas others exclusively included MASLD patients sub-grouped by the presence or absence of MASH or varying stages of fibrosis.^5^^,^^9^^–^^12^ This represents a major issue for developing therapeutics aimed at limiting fibrosis and MASLD progression. The goal of our study is to address this issue using a comprehensive, in-depth meta-analysis.

Conducting a meta-analysis is a powerful approach to leverage multiple thematically linked studies by using previously published bulk hepatic transcriptome analyses to expand the sample size, increase the power of detection of commonly regulated genes among cohorts, define reproducible gene expression signatures, and identify tractable therapeutic targets in a reproducible manner.^13^^,^^14^ Adding to this, recent advances in bioinformatics and data analysis allow for accurate characterization of cell diversity using existing collective data sources.^15^ Incorporating these techniques, we leveraged our own study along with 12 publicly available bulk transcriptomic datasets with fibrosis staging to perform a meta-analysis of almost 1000 patients with MASLD. Given that fibrosis is the strongest predictor of long-term outcomes in MASLD, and that fibrosis stage serves as a quantifiable phenotype, we stratified solely on fibrosis stage to identify signatures associated with advanced fibrosis in patients with MASLD. Leveraging this robust dataset, our meta-analysis compared MASLD patients with advanced fibrosis (F3–4) and mild/no fibrosis (F0–1) and identified 846 differentially regulated genes, robust changes in cell type proportions, and enrichment of receptor–ligand pairs associated with advanced fibrosis. These conserved signatures represent important fibrogenic drivers and potentially targetable pathways that advance our understanding of the molecular pathogenesis of fibrosis progression.

## METHODS

### Dataset collection

To select datasets for our meta-analysis, we searched PubMed and GEO datasets in 2022 for recently published papers and datasets including transcriptomic analysis of patients with NAFLD/MASLD.^16^ Prior studies found high consistency between NAFLD and MASLD diagnoses, allowing us to compare datasets with confidence.^2^ Out of 27 datasets available at the time the meta-analysis was performed, 13 included fibrosis staging. We chose to include all 13 studies, including those involving pediatric MASLD or non-tumor liver tissue from HCC patients, to increase the potential of our meta-analysis to find meaningful and conserved fibrosis-associated genes. Eight hepatic bulk RNA-seq and 4 microarray datasets were downloaded from the National Center for Biotechnology Information GEO datasets (https://www.ncbi.nlm.nih.gov/), including our own RNA-seq data (GSE225740), in addition to the Gerhard dataset obtained from personal communication (patient demographics previously described).^17^ RNA-seq dataset accession numbers are GSE130970, GSE135251, GSE162694, GSE185051, GSE192959, GSE193066, and GSE193080; microarray datasets are GSE49541, GSE89632, GSE96971, and GSE48452. More detailed information is included in Table 1.

**TABLE 1:** Description of datasets included in the meta-analysis

	Dataset information									
Type	First author	Dataset ID	Profiling type	Average age (SD)	Sex (% female)	Diabetes (% with)	F0–1	F3–4	Other^a^	Sum
Bulk transcriptome	Hoang^5^	GSE130970	RNA-seq	50.56 (12.21)	61.54%	N/A	16	53	9	78
	Govaere^12^ ^b^	GSE135251	RNA-seq	54.00 (11.87)	40.29%	53.40%	85	68	63	216
	Pantano^14^	GSE162694	RNA-seq	46.04 (13.02)	72.03%	N/A	65	20	58	143
	Yao^8^	GSE185051	RNA-seq	16.58 (10.69)	43.86%	N/A	43	5	9	57
	Fujiwara^96^	GSE192959	RNA-seq	71.25 (5.92)	66.67%	N/A	2	43	3	48
	Fujiwara^96^ ^c^	GSE193066	RNA-seq	51.84 (12.97)	40.00%	N/A	43	26	95	164
	Fujiwara^96^	GSE193080	RNA-seq	72.00	25.42%	43.00%	25	24	10	59
	Gerhard^d^ ^17^	N/A	RNA-seq	N/A	80.21%	N/A	103	65	24	192
	Powell^16^	GSE225740	RNA-seq	50.00 (12.58)	59.14%	44.87%	54	18	21	93
	Moylan^6^	GSE49541	Microarray	50.57 (11.13)	65.28%	37.50%	40	32	0	72
	Arendt^97^	GSE89632	Microarray	41.48 (11.29)	46.03%	13.11%	45	8	10	63
	Allard^e^	GSE96971	Microarray	49.11 (11.02)	33.33%	22.22%	5	7	6	18
	Ahrens^98^	GSE48452	Microarray	45.91 (11.31)	79.45%	N/A	66	4	3	73
	**Total or average**			**49.95 (14.07)**	**54.87%**	**35.68%**	**592**	**373**	**311**	**1276**
	**First author**	**Dataset ID**	**Profiling type**	**Average age**	**Sex (% female)**	**Control**	**Cirrhosis**	**Cell types**	**Number of cells**	
scRNA-seq	Ramachandran^22^	GSE136103	scRNA-seq	57	30%	5	5	MP, pDC, ILC, T cell, B cell, plasma cell, mast cell, endothelia, mesenchyme, hepatocyte, cholangiocyte	66,135	

*Note:* Information regarding datasets used for the meta-analysis.

^a^ Denotes samples with a mix of other fibrosis stages, second biopsies performed on patients at high risk for HCC, or unavailable fibrosis data.

^b^ Demographics for control patients not available.

^c^ Samples were obtained after a first or second biopsy. The 58 biopsy data points are listed under “Other” because they were repeats from the same patients and were not included in our meta-analysis. The age, sex, diabetes status, and fibrosis counts only summarize the first biopsy measurements.

^d^ Denotes data obtained from personal communication.

^e^ Measurements were taken from the same 9 patients at baseline and after 12 months, and age, sex, and diabetes status were averaged from the baseline measurements. Fibrosis staging takes into account samples from baseline and at follow-up, as these were both used for the meta-analysis.

Abbreviations: F0–1, fibrosis stages 0–1; F3–4, fibrosis stages 3–4; HCC, hepatocellular carcinoma; ILC, innate lymphoid cell; MP, mononuclear phagocyte; N/A, not available; pDC, plasmacytoid dendritic cell; RNA-seq, RNA sequencing; scRNA-seq, single-cell RNA sequencing; SD, standard deviation.

### Meta-analysis of gene expression, cell type proportions across bulk RNA-seq and microarray datasets

To assess the differences in gene expression, we performed differential expression analysis using DESeq2 for each RNA-seq dataset,^18^ and *t* test for each microarray dataset. To assess the differences in deconvoluted cell-type proportions, we employed the Wilcoxon rank-sum test. Meta-analysis across datasets was performed using Edgington’s sum-of-*p*-values method.^19^ Further details are included in the Supplemental Methods, http://links.lww.com/HC9/C393.

### RNA-seq preparation and analysis of MAFALDA (confirmation) cohort

RNA extraction, library preparation, read mapping, and read counting for the MAFALDA (Molecular Architecture of Fatty Liver Disease in individuals with obesity undergoing bariatric surgery) cohort were performed as previously described.^20^ Liver samples with a uniquely mapped reads-to-total reads ratio below 0.7 were excluded. The MAFALDA validation cohort consisted of 191 patients with well-characterized MASLD, including 7 individuals with F3–4 and 184 with F0–1. Differential gene expression analysis was conducted using DESeq2 (v1.40.0) on 191 liver samples, adjusting for RNA integrity, age, and sex as covariates.

### Deconvolution pipeline using single-cell-derived cell type markers

We utilized a semi-supervised deconvolution pipeline based on single-cell reference data to perform deconvolution on frames. This process employs an approach similar to semi-supervised mouse data deconvolution (SSMD),^21^ which involves 3 key steps: (i) identifying and training a list of specific marker genes associated with cell types of interest in liver tissue, (ii) assessing the distinguishability of each cell type and validating corresponding marker genes for every dataset intended for deconvolution, and (iii) determining the proportion of each identified cell type within the samples. The scRNA-seq data used for the deconvolution (GSE136103) were obtained from healthy non-lesional liver tissues from patients undergoing surgical resection, while cirrhotic liver tissue was obtained intraoperatively from patients undergoing liver transplantation (Table 1).^22^ The deconvolution pipeline is described in detail in Supplemental Methods, http://links.lww.com/HC9/C393.

### Quantitative analysis of ligand–receptor expression in cell–cell communication

To identify potential interactions that drive MASLD-associated fibrosis, we performed a ligand–receptor analysis on bulk RNA-seq or microarray datasets (GSE130970, GSE135251, GSE162694, GSE185051, GSE192959, GSE193066, GSE193080, GSE 126848, and GSE49541). For the bulk RNA-seq data, we used BulkSignalR, a statistical framework that infers potential ligand–receptor interactions based on co-expression patterns across bulk samples.^23^ It integrates known ligand–receptor interactions with downstream signaling pathways to enhance the inference of cell–cell communication from bulk transcriptomic data. By evaluating the co-expression of ligands, receptors, and their associated downstream targets within established pathways, BulkSignalR provides a more comprehensive understanding of intercellular signaling events.^23^ BulkSignalR does not indicate which cell type pairs express a given ligand–receptor pair. To address this, we examined the expression of the detected ligand and receptor genes in our single-cell RNA-seq data. To assess whether a gene was expressed by a given cell type or subtype, we calculated the proportion of cells within that group exhibiting non-zero expression of the gene, using a proportion cutoff of 50% to call a gene being expressed by a cell type, and an average expression greater than 1. We used these stringent cutoffs to increase the reliability of our findings based on prior studies.^24^^,^^25^

## RESULTS

### Meta-analysis revealed DEGs related to the fibroinflammatory niche associated with advanced fibrosis

**FIGURE 1:**
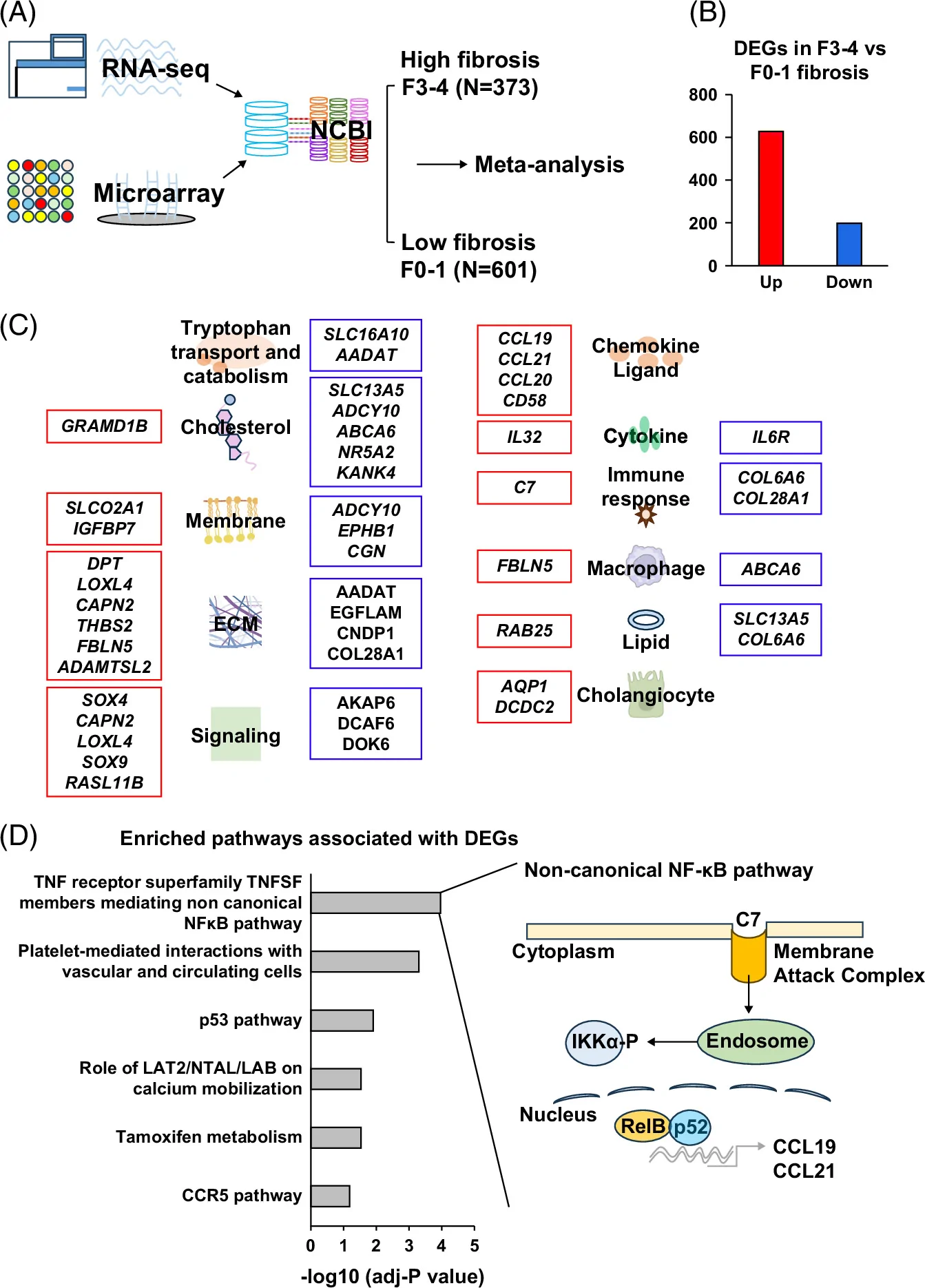
A meta-analysis of ~1000 patients with MASLD identifies conserved DEGs and signaling pathways. (A) Schematic of data sources and sample numbers for meta-analysis. (B) Number of significant upregulated and downregulated genes identified in our meta-analysis (adj. *p*-value <0.05). (C) Major location and/or function of top significantly upregulated (red) and downregulated (blue) DEGs comparing F3–4 versus F0–1 fibrosis. (D) Meta-analysis of enriched pathways across transcriptomic datasets (adj. *p*-value <0.03, except CCR5 adj. *p*-value=0.06). Arrows indicate the flow of the process. Abbreviations: CCR5, C-C motif chemokine receptor 5; DEGs, differentially expressed genes; ECM, extracellular matrix; F0–1, fibrosis stages 0–1; F3–4, fibrosis stages 3–4; MASLD, metabolic dysfunction–associated steatotic liver disease; NCBI, National Center for Biotechnology Information; NF-κB, nuclear factor kappa B; RNA-seq, RNA sequencing; TNF, tumor necrosis factor.

To perform the meta-analysis, we combined RNA-seq and microarray data to identify differentially expressed genes (DEGs) between MASLD patients with F3–4 (n=373) versus F0–1 fibrosis (n=601) (Figure 1A). We omitted patients with F2 in order to better stratify patients with no/early fibrosis and advanced fibrosis. This analysis identified 846 DEGs, with 626 upregulated and 220 downregulated genes between F3–4 versus F0–1 groups (adj *p*-value <0.05, Figure 1B, all DEGs listed in Supplemental Table S1, http://links.lww.com/HC9/C393). We performed a literature search on the top 30 upregulated and top 20 downregulated genes to better understand their roles in liver disease (Supplemental Table S2, http://links.lww.com/HC9/C393), including function, established associations or mechanistic roles in MASLD-fibrosis, their role in other liver diseases (HCC, viral hepatitis, cholestatic liver disease), and the highest expressing hepatic cell types based on scRNA-seq data.^22^ As expected, some of the most significant genes were previously identified as signature genes of MASH and/or liver fibrosis. Several of these genes are involved in ECM organization and cell–cell matrix interactions, such as thrombospondin 2 (*THBS2*),^26^^,^^27^ a disintegrin and metalloproteinase with thrombospondin motifs-like 2 (*ADAMTSL2*),^28^ fibulin 5 (*FBLN5*),^29^ lumican (*LUM*),^29^^,^^30^ C-X-C motif chemokine ligand 6 (*CXCL6*),^31^ and both SRY-box transcription factor 9 (*SOX9*) and SRY-box transcription factor 4 (*SOX4*).^32^^–^^34^ Other genes in common with previous studies include aldo-keto reductase family 1 member B10 (*AKR1B10*)^12^^,^^35^ and fibrillin-1 (*FBN1*).^36^^,^^37^

Importantly, some less characterized DEGs were highly significant in our meta-analysis, highlighting the value of a meta-analysis approach. Solute carrier family 16 member 10 (*SLC16A10*) and aminoadipate aminotransferase (*AADAT*) were 2 of the top downregulated genes, and both participate in aromatic amino acid and tryptophan transportation and catabolism.^38^^,^^39^ A second set of genes differentially regulated in MASLD-fibrosis have functions related to cholesterol transportation or response to cholesterol treatment: with downregulation of solute carrier family 13 member 5 (*SLC13A5*), adenylate cyclase 10 (*ADCY10*), ATP binding cassette subfamily A member 6 (*ABCA6*), nuclear receptor subfamily 5 group A member 2 (*NR5A2*), KN motif and ankyrin repeat domains 4 (*KANK4*), and upregulation of GRAM domain containing 1B (*GRAMD1B*) (Figure 1C). Finally, immune-related chemokines, cytokines, and macrophage- and immune response-related genes are among the top significantly upregulated genes, including chemokine ligands C-C motif chemokine ligand 19 (*CCL19*), *CCL20*, *CCL21*, CD58 molecule (*CD58*), and *CXCL6*, cytokines [interleukin 32 (*IL32*)], and immune response-associated genes like complement C7 (*C7*) (Figure 1C).

### Confirmation of identified DEGs

To confirm our findings, we compared the top 30 upregulated and top 20 downregulated DEGs to transcriptomics obtained from patients in the MAFALDA cohort, comparing MASLD patients with advanced fibrosis (F3–4, n=7) versus mild or no fibrosis (F0–1, n=184). Despite a limited number of patients with advanced fibrosis, 7 out of the top 50 DEGs were significantly changed in the same direction as our meta-analysis (adj *p*-value <0.05), including genes without a prior association with fibrosis, like calpain 2 (*CAPN2*) (Supplemental Table S3, http://links.lww.com/HC9/C393). This confirmation supports the importance and robustness of our meta-analysis.

### DEGs reveal the enrichment of potential biomarkers in MASH

A critical need in the MASLD field is reliable biomarkers to predict disease severity and fibrosis stage. In all, 48 DEGs identified in our meta-analysis encoded proteins are known or predicted to be secreted (Supplemental Table S4, http://links.lww.com/HC9/C393). To inform potential biomarkers, we compared DEGs from our meta-analysis to 2 recent studies where proteomic analysis was performed on serum from patients with MASLD. The first study by Govaere et al^12^ compared serum from patients with MASLD-fibrosis (F4) to patients with MASLD (F0), while the second study by Sanyal et al^40^ compared serum from MASLD patients with F2–4 to F0–1. Proteins encoded by several of the DEGs from our meta-analysis were similarly increased or decreased in both proteomic studies, including ADAMTSL2, C7, and neurofascin (NFASC) (Supplemental Table S5, http://links.lww.com/HC9/C393).

### Pathway analysis of DEGs highlights immune-associated and cancer-associated pathways

To better understand the differentially regulated pathways in advanced fibrosis, we performed a meta-analysis of pathway enrichment across the transcriptomic datasets. Pathway analysis was performed on the individual RNA-seq and microarray datasets using BioCarta, Reactome, and Wikipathways to compare F3–4 to F0–1, followed by a meta-analysis and identification of significant pathways. Four out of the top 6 pathways represented potential immune-mediated pathological mechanisms: non-canonical NF-κB signaling, platelet-mediated interactions with circulating cells, LAT2-mediated calcium mobilization, and C-C motif chemokine receptor 5 (*CCR5*) pathways (Figure 1D). Consistent with a potential role for NF-κB in MASLD progression, several DEGs associated with non-canonical NF-κB signaling were altered, including *C7*, *RELB*, and *NFKB2/p52*. Differential activity of these transcriptional regulators could alter expression of chemokines such as *CCL19* and *CCL21*.^41^^,^^42^ C7 is a constituent of the membrane attack complex (MAC), which forms pores, acts as a membrane anchor, and is a critical component of the innate immune system and inflammatory response. Endocytosis of the MAC complex drives a signaling cascade leading to RelB/p52 complex translocation to the nucleus, which subsequently enhances *CCL19* and *CCL21* expression to drive inflammation (Figure 1D, right panel).

In addition to inflammatory signatures, DEGs associated with cancer and p53 signaling were enriched in advanced fibrosis. DEGs previously found to be associated with HCC include carnosine dipeptidase 1 (*CNDP1*),^43^ nuclear receptor subfamily 5, group A, member 2 (*NR5A2*),^44^ and lysyl oxidase-like 4 (*LOXL4*).^45^^,^^46^ Other DEGs are linked to several types of cancer: HORMA domain containing 2 (*HORMAD2*),^13^^,^^47^ *KANK4* (bladder cancer),^48^ *DCAF6* (prostate cancer),^49^ and ATPase copper transporting beta (*ATP7B*) (breast cancer).^50^ These data indicate that cancer-associated gene signatures are already present at stage F3–4.

To complement our gene-based pathway analyses, we analyzed how proteins encoded by the DEGs may interact. STRING analysis was performed using the top 100 upregulated or top 100 downregulated genes as input to identify enriched clusters with associated functions. Upregulated proteins formed multiple clusters, including ECM organization [collagen type I alpha 2 chain (COL1A2), COL3A1, LUM, fibromodulin (FMOD), THBS2]; neutrophil chemotaxis (CCL19, CCL20, CCL21, CXCL6); WNT signaling (WNT4, LEF1, SOX4); and angiogenesis (CLEC14A and EMCN) (Supplemental Figure S1, http://links.lww.com/HC9/C393). Enrichment of these pathways was expected, as ECM organization is central to fibrosis development, neutrophil recruitment is associated with MASLD and MASH, and angiogenesis is tightly associated with fibrosis progression.^51^^–^^54^ STRING analysis of downregulated genes revealed several functional clusters which relate to amino acid transportation and metabolism, fatty acid metabolism, and mitochondrial function (Supplemental Figure S2, http://links.lww.com/HC9/C393).

Together, we identified alterations in hepatic gene expression with advanced fibrosis associated with ECM organization, immune response activation, changes in metabolism, amino acid synthesis and transportation, intercellular and intracellular transport, and other potentially impactful signaling pathways.

### ScRNA-based deconvolution of the bulk gene expression to identify alterations in the proportion of cell populations in fibrosis

**FIGURE 2:**
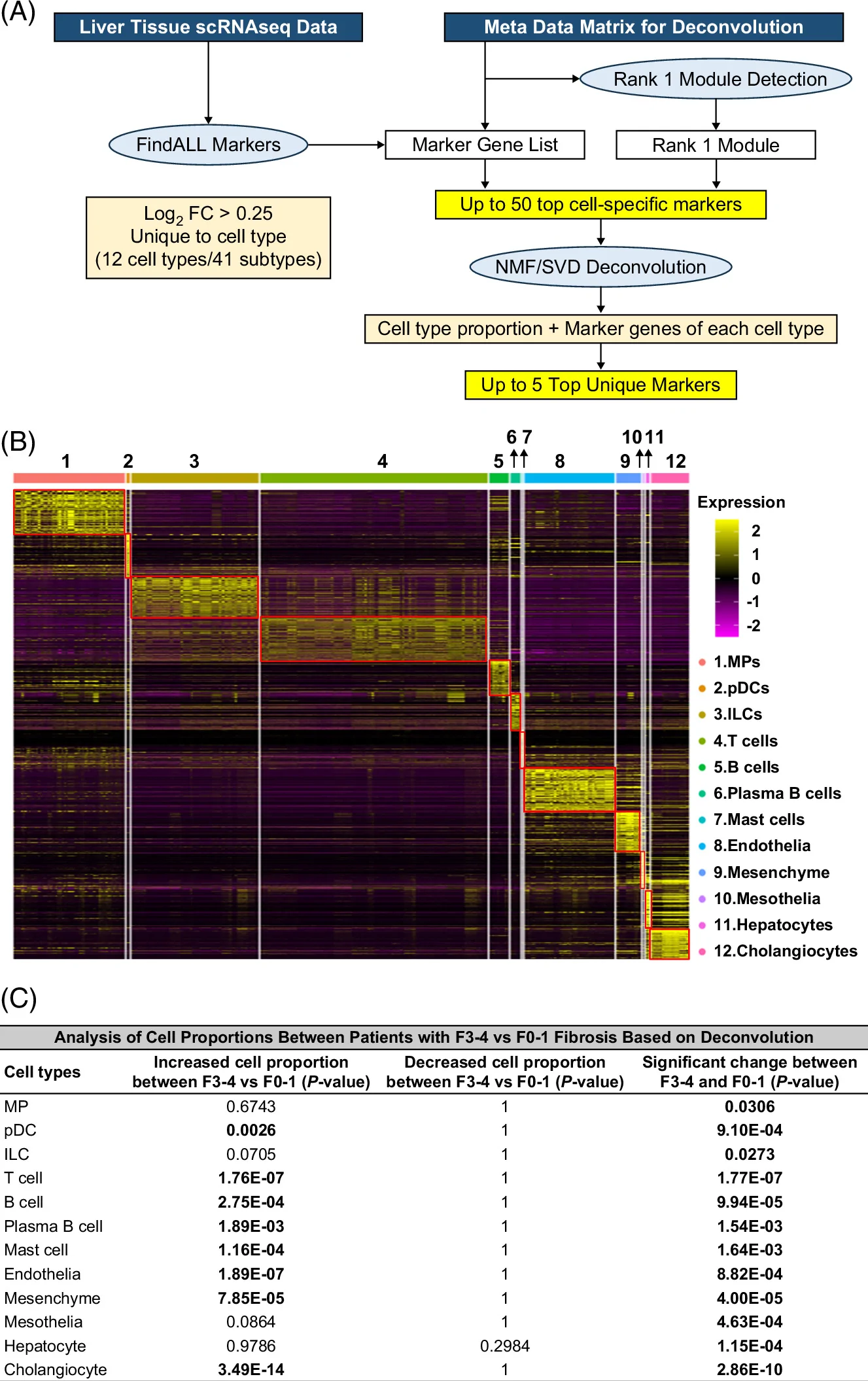
Deconvolution of transcriptomic data identifies 12 major hepatic cell types. (A) Flowchart depicting the deconvolution approach. Light blue boxes indicate methods, tan boxes indicate criteria or filters, and yellow boxes indicate marker genes. (B) Heatmap of the single-cell RNA-seq dataset of liver tissue. The columns represent the 12 cell types (containing subtypes) of interest, while the rows represent the expression of up to 50 highly expressed gene markers in all cell types. Marker genes for each cell type are highlighted by red boxes. (C) Deconvolution of single-cell types in meta-analysis in patients with F3–4 compared with patients with F0–1. Bold numbers indicate a significant difference between groups (*p*<0.05). Abbreviations: F0–1, fibrosis stages 0–1; F3–4, fibrosis stages 3–4; RNA-seq, RNA sequencing; scRNA-seq, single-cell RNA sequencing.

In addition to understanding transcriptomic changes, it is crucial to study changes in cell types and cell-specific expression in MASLD progression. We sought to identify changes in relative abundance and proportion of cell types associated with advanced fibrosis through deconvolution. To perform a deconvolution meta-analysis, we used scRNA-seq data generated from liver tissue samples from 10 patients with healthy or cirrhotic livers, in which 12 major cell types have been identified and annotated.^22^ First, we identified cell-type-specific candidate marker genes using scRNA-seq data. Second, we set a log2 fold change (FC) cutoff of >0.25 and cell type-specific expression selection to identify up to 50 highly expressed marker genes (Figure 2A). The heatmap displays all gene expression levels with the top gene markers boxed in each cell type (Figure 2B and marker genes listed in Supplemental Table S6, http://links.lww.com/HC9/C393). Yellow indicates higher expression, and these markers are more specific to the corresponding cell types.

Using the cell type-specific marker genes, we performed a semi-supervised, single-cell-based meta-analysis approach to detect cell types and to determine whether any were proportionally imbalanced between F3–4 and F0–1 groups by calculating the relative cell type proportions for both the combined meta-analysis as well as each RNA-seq dataset. Remarkably, there were significant disproportions in the prevalence of 8 of the 12 major cell types between F3–4 and F0–1 groups, with higher presence of pDC, T cell, B cell, plasma B cell, mast cell, endothelia, mesenchymal cell, and cholangiocytes in F3–4 (*p*<0.05; Figure 2C).

**FIGURE 3:**
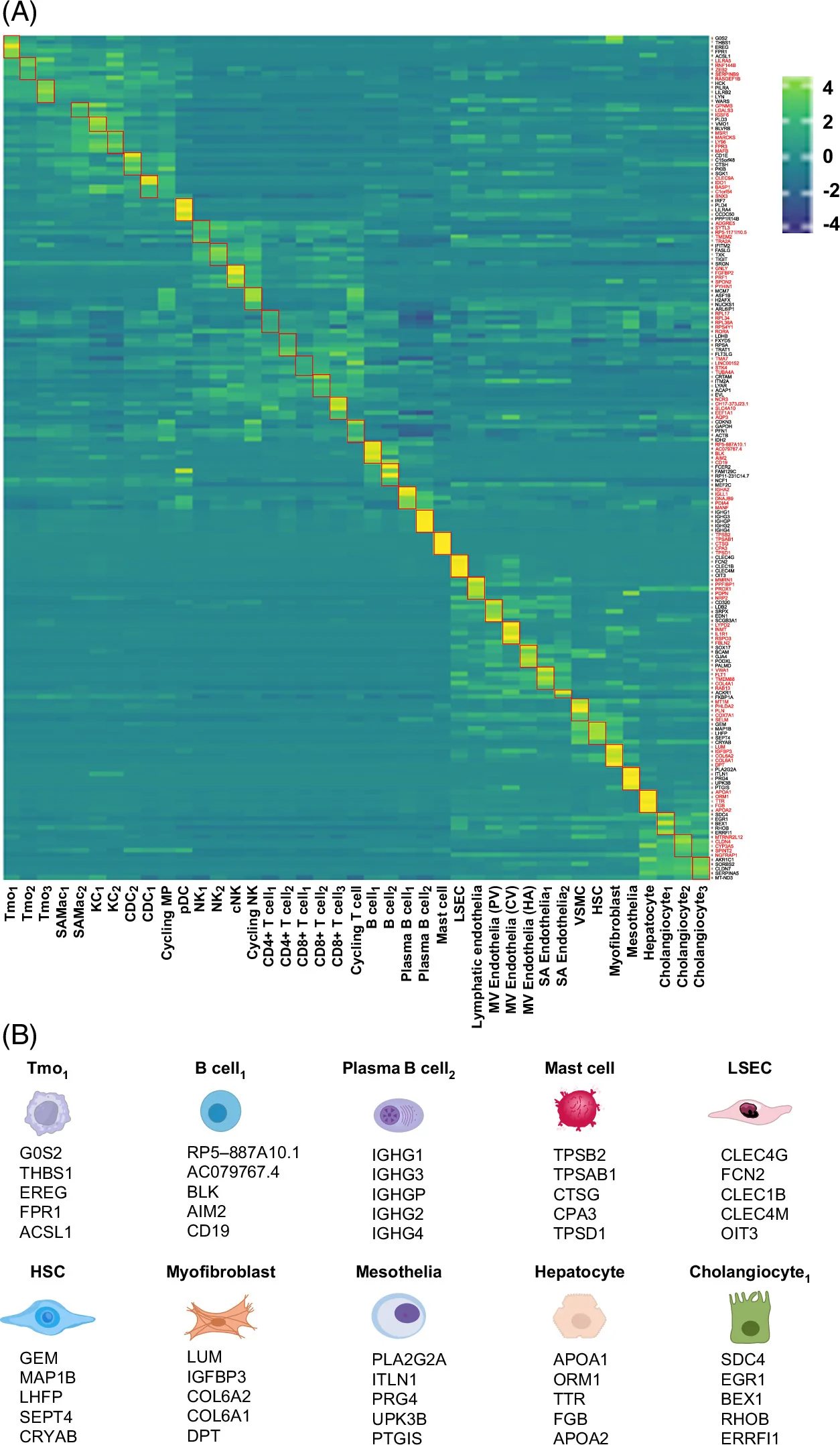
Deconvolution of transcriptomic datasets identifies specific cell subtypes based on marker genes. (A) Heatmap visualizing gene expression of up to 5 cell-specific gene markers in each cell subtype. Marker genes for each cell subtype are highlighted by red boxes. Although 41 subtypes are shown, unique marker genes were not identified for 2 of them and are therefore not included in subsequent analyses. (B) Representative subtype-specific gene markers. Abbreviations: HSC, hepatic stellate cell; LSEC, liver sinusoidal endothelial cell.

We expanded our analysis to cell subtypes by identifying cell-type-specific marker genes common in both single-cell and meta-analysis datasets using iterative hierarchical clustering and bi-cross-validation methods. The heatmap (Figure 3A) depicts the expression patterns of the top cell-specific marker genes in 41 cell subtypes, though no specific markers were found in 2 subtypes. We chose to retain the top 5 marker genes, with the exception of cell subtypes that possess fewer than 5 unique marker genes. The top unique cell type-specific marker genes are presented in Supplemental Table S7, http://links.lww.com/HC9/C393, and representative cell subtypes with unique marker genes are presented in Figure 3B.

**FIGURE 4:**
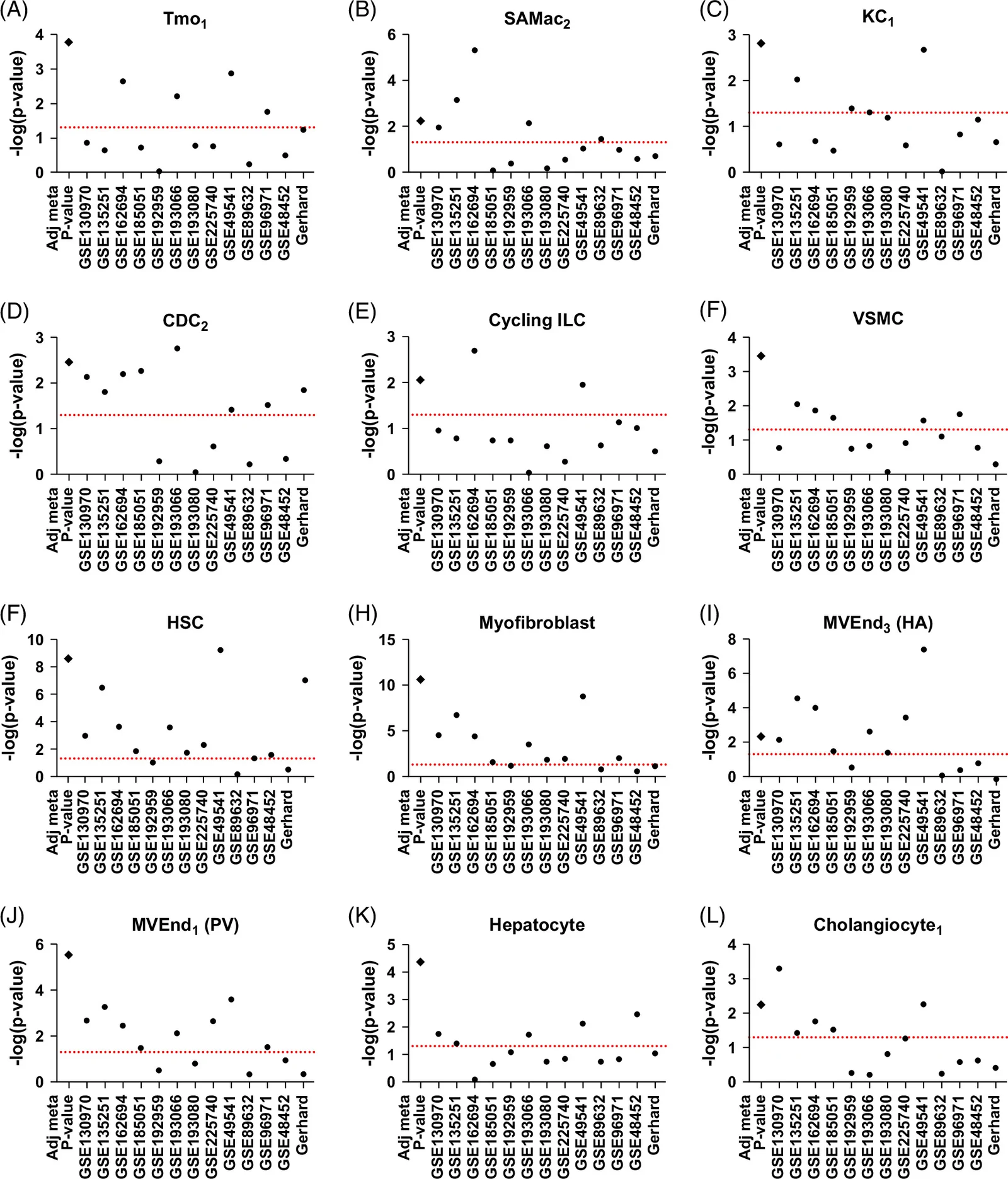
Deconvolution identifies multiple hepatic cell types with significantly changed proportions in patients with advanced fibrosis. Changes in cell subtype proportions were calculated for each of the 13 datasets, as well as the meta-analysis, and are shown as scatter plots. The adjusted meta *p-*value for all 13 datasets is shown as a diamond, and each individual dataset as circles. Cell subtypes with a meta-analysis *p*-value <0.01 are shown, with the full dataset in Supplemental Table S6, http://links.lww.com/HC9/C393. A red dashed line is positioned at −log_10_(0.05), and the dots above this line represent statistically significant values. Abbreviations: Tmo, Tissue monocyte; SAM, scar associated macrophage; KC, kupffer cell; cDC, classical dendritic cell; ILC, innate lymphoid cell; VSMC, vascular smooth muscle cells; HSCs, hepatic stellate cell; MVEnd, microvascular enothelial cell; HA, hepatic artery; PV, portal vein.

Analysis of the 13 datasets revealed significant differences in the mean proportion of 29 cell subtypes in F3–4 compared with F0–1, including several MP and ILC subtypes, as well as mesenchyme subtypes (Figure 4). Furthermore, several of the *p*-values from the one-sided upper tail test show significance, highlighting increased proportions of these cell types in the F3–4 group compared with the F0–1 group (Supplemental Table S8, http://links.lww.com/HC9/C393).

### Quantitative analysis of ligand–receptor expression in cell–cell communication

Cell–cell communication through ligand–receptor interactions is crucial to liver physiology, while dysregulation of these interactions drives liver fibrosis. To explore ligand–receptor interactions dysregulated in advanced fibrosis, we analyzed each transcriptomic dataset to identify ligand–receptor pairs that were enriched across multiple analyses and compared these ligands/receptors with the DEGs from our meta-analysis. We identified 8 ligand–receptor pairs enriched in all 11 datasets analyzed, as well as several others enriched in at least 6 datasets and containing DEGs identified in the meta-analysis. Several pairs were associated with HSC activation, cell–matrix interactions, and cytokine signaling (Table 2). Of the 8 ligand–receptor interactions enriched across all datasets, 4 involved genes significantly increased in patients with F3–4 fibrosis: jagged canonical Notch ligand 1 (JAG1)–Notch receptor 3 (NOTCH3), nephronectin (NPNT)–ITGA8, platelet-derived growth factor D (PDGFD)–PDGFRA, and vimentin (VIM)–CD44. Signaling through JAG1–NOTCH3, ITGA8, PDGFD–PDGFRA, VIM, and CD44 has all been previously implicated in liver fibrosis.^55^^–^^59^

**TABLE 2:** Enrichment of ligand–receptor pairs in F3–4 patients predicts signaling pathways that may drive fibrosis

Ligand–receptor pairs enriched in multiple studies								
Ligand	Receptor	Number of studies	Ligand	Receptor	Number of studies	Ligand	Receptor	Number of studies
**JAG1**	NOTCH3	11	**COL6A3**	ITGA2	9	**BMP5**	BMPR2	8
KITLG	KIT	11	**CXCL6**	**ADRA2A**	9	**LAMA2**	**ITGA6**	8
LGALS9	HAVCR2	11	HLA-B	**CD3D**	9	**LAMB1**	**ITGAV**	8
**NPNT**	ITGA8	11	**LAMC2**	ITGA2	9	MFGE8	**PDGFRB**	8
**PDGFD**	**PDGFRA**	11	**PLAU**	ITGA3	9	**PDGFD**	**PDGFRB**	8
SELPLG	SELL	11	**PLAU**	ITGAM	9	**PLAU**	**ITGAV**	8
**VIM**	CD44	11	TLN1	**ITGB5**	9	**VCAN**	TLR2	8
SEMA4G	PLXNB2	11	**CCL2**	CCR5	9	CCL2	**CCR2**	8
**CCL21**	**ADRA2A**	10	**COL1A1**	ITGA11	9	ADAM9	**ITGA6**	8
HLA-A	**CD3D**	10	**COL1A2**	ITGA11	9	**COL14A1**	CD44	8
**LAMA2**	ITGA2	10	**COL4A1**	ITGB1	9	**COL1A2**	CD44	8
**LAMA2**	ITGA3	10	LAMC1	**ITGAV**	9	**COL1A2**	**ITGAV**	8
**LAMB1**	ITGA3	10	**F13A1**	ITGA4	9	**CXCL1**	**ADRA2A**	8
**LAMC2**	ITGA3	10	**LUM**	ITGB1	9	EDIL3	**ITGAV**	8
**LAMC3**	ITGA3	10	**SPP1**	CD44	9	**LAMA2**	ITGB1	8
**VCAN**	CD44	10	**SPP1**	**ITGAV**	9	LAMC1	**ITGA6**	8
**VCAN**	ITGA4	10	B2M	**CD3D**	8	**PDGFA**	**PDGFRA**	8
**EFEMP2**	**AQP1**	10	**CCL19**	**ADRA2A**	8	**SPP1**	ITGB1	8
ADAM9	**ITGAV**	10	**CD40LG**	ITGB2	8	THBS1	**ITGA6**	8
**COL4A1**	**ITGAV**	10	**CXCL12**	**ADRA2A**	8	**VCAN**	ITGB1	8
**COL4A4**	**ITGAV**	10	**HSPG2**	PTPRS	8	**BMP5**	BMPR2	8
**FBN1**	**ITGAV**	10	ICAM1	**IL2RG**	8	**LAMA2**	**ITGA6**	8
**FBN1**	ITGB1	10	**LAMB1**	ITGA2	8	**CCL20**	**ADRA2A**	7
**MMP7**	**CDH6**	10	**LAMC2**	ITGB4	8	**CCL19**	**CXCR3**	7
**SLIT2**	**ROBO1**	10	**LAMC3**	ITGB4	8	**F13A1**	**ITGA9**	7
**THBS2**	**ITGA6**	10	**PLAU**	ITGB2	8	**ANXA1**	**FPR3**	7
SYK	**FCGR2A**	10	**PLAU**	PLAUR	8	**PDGFA**	**PDGFRB**	7
**ALOX5AP**	ALOX5	9	TGFB3	**ITGB5**	8	**SPP1**	**ITGA9**	7
**ANXA1**	FPR1	9	**THBS2**	NOTCH3	8	**LAMB1**	**ITGA6**	7
**COL18A1**	GPC1	9	ITGB2	**THY1**	8	**ANXA1**	**ADRA2A**	6
**COL1A2**	ITGA2	9	**COL3A1**	DDR2	8	**COL1A1**	**ITGAV**	6
**COL4A2**	ITGB5	9	**ANGPT1**	ITGB1	8	**CTHRC1**	**FZD6**	6
**COL4A4**	ITGA2	9						

*Note:* RNA-seq data were analyzed to identify ligand–receptor pairs enriched in advanced fibrosis. Included pairs were enriched across the 11 analyzed datasets, enriched in 8–10 datasets, and contained a gene identified as significantly changed in our meta-analysis (bolded), or enriched in 6–7 datasets with both the ligand and receptor significantly changed in our meta-analysis.

To determine which cell types express the genes identified in our ligand–receptor pairs, we compared L/R pairs containing a DEG from our meta-analysis with cell-specific expression profiles derived from the scRNA-seq dataset used for our deconvolution, using a cutoff for gene expression as an average expression above 1 in >50% of cells. We have identified and highlighted the expression of ligand–receptor pairs across major cell types (Table 3), with the expression of the ligand or receptor in all cell types listed and ligand–receptor interactions that have potential implications for liver fibrosis development highlighted in Supplemental Table S9, http://links.lww.com/HC9/C393. These include pairs involved in cell–matrix interactions (MMP7–CDH6, SPP1–ITGB1), immune cell interactions (ALOX5AP–ALOX5, VCAN–CD44), and potential recruitment of immune cells to sites of injury (HLA-A–CD3D, LUM–ITGB1). Other representative interactions between ligand and receptors and their respective cell types are located in Supplemental Figure S3, http://links.lww.com/HC9/C393.

**TABLE 3:** Ligand and receptor average and proportional expression in major cell types

Process	Ligand Receptor	Expression	MP	pDC	ILC	T cell	B cell	Plasma B cell	Mast cell	Endothelia	Mesenchyme	Mesothelia	Hepatocyte	Cholangiocyte
Unknown	**EFEMP2**	Avg.	—	—	—	—	—	—	—	—	—	***1.63***	—	—
		Prop.	—	—	—	—	—	—	—	—	—	***76.85***	—	—
	**AQP1**	Avg.	—	—	—	—	—	—	—	3.30	—	***3.22***	—	3.31
		Prop.	—	—	—	—	—	—	—	57.51	—	***87.96***	—	66.46
ECM Interactions	**COL4A1**	Avg.	—	—	—	—	—	—	—	**2.16**	—	—	—	—
		Prop.	—	—	—	—	—	—	—	**50.83**	—	—	—	—
	ITGB1	Avg.	1.12	—	1.23	1.03	—	—	—	**3.93**	5.29	1.77	2.02	3.44
		Prop.	43.63*	—	26.45*	21.70*	—	—	—	**76.35**	76.00	81.48	78.13	68.77
ECM Interactions	**LUM**	Avg.	—	—	—	—	—	—	—	—	3.55	—	—	—
		Prop.	—	—	—	—	—	—	—	—	14.27*	—	—	—
	ITGB1	Avg.	1.12	—	1.23	1.03	—	—	—	3.93	5.29	1.77	2.02	3.44
		Prop.	43.63*	—	26.45*	21.70*	—	—	—	76.35	76.00	81.48	78.13	68.77
ECM Interactions	**SPP1**	Avg.	—	—	—	—	—	—	—	—	—	—	3.10	**14.32**
		Prop.	—	—	—	—	—	—	—	—	—	—	46.88*	**79.58**
	ITGB1	Avg.	1.12	—	1.23	1.03	—	—	—	3.93	5.29	1.77	2.02	**3.44**
		Prop.	43.63*	—	26.45*	21.70*	—	—	—	76.35	76.00	81.48	78.13	**68.77**
ECM Interactions	**MMP7**	Avg.	—	—	—	—	—	—	—	—	—	—	—	4.73
		Prop.	—	—	—	—	—	—	—	—	—	—	—	57.94
	**CDH6**	Avg.	—	—	—	—	—	—	—	—	1.24	—	—	—
		Prop.	—	—	—	—	—	—	—	—	28.71*	—	—	—
ECM Interactions	**VIM**	Avg.	**13.24**	11.33	2.77	8.28	4.15	4.71	**16.72**	34.99	30.07	14.69	1.26	3.88
		Prop.	**91.60**	96.14	41.45*	73.28	64.08	91.69	**87.14**	87.98	96.69	97.22	59.06	66.58
	CD44	Avg.	**3.47**	1.04	1.35	2.78	1.34	—	**5.76**	—	—	—	—	—
		Prop.	**68.70**	41.05*	26.87*	49.05*	35.34*	—	**67.14**	—	—	—	—	—
Immune cell recruitment and interactions	SELPLG	Ave.	—	***1.71***	—	—	—	—	—	—	—	—	—	—
		Prop.	—	***56.14***	—	—	—	—	—	—	—	—	—	—
	SELL	Ave.	—	***4.27***	—	—	2.70	—	—	—	—	—	—	—
		Prop.	—	***84.21***	—	—	50.78	—	—	—	—	—	—	—
Immune cell recruitment and interactions	HLA-A	Avg.	18.08	14.09	40.26	**36.04**	20.58	7.93	13.47	30.29	7.45	7.57	8.93	12.90
		Prop.	95.51	97.19	98.58	**98.37**	97.34	95.06	80.00	99.40	80.30	96.30	96.25	91.30
	**CD3D**	Avg.	—	—	—	**8.88**	—	—	—	—	—	—	—	—
		Prop.	—	—	—	**82.88**	—	—	—	—	—	—	—	—
Immune cell recruitment and interactions	HLA-B	Avg.	35.75	39.06	61.16	***56.65***	39.35	23.44	17.27	50.44	9.75	14.32	9.56	12.33
		Prop.	98.71	99.30	99.59	***99.48***	99.07	99.55	91.43	99.87	84.56	99.07	97.19	91.54
	**CD3D**	Avg.	—	—	—	***8.88***	—	—	—	—	—	—	—	—
		Prop.	—	—	—	***82.88***	—	—	—	—	—	—	—	—
Immune cell recruitment and interactions	B2M	Avg.	156.36	126.50	191.19	**208.10**	118.13	83.89	139.11	152.99	51.39	51.06	35.47	42.84
		Prop.	99.98	100.00	100.00	**100.00**	100.00	99.89	100.00	100.00	99.32	100.00	100.00	99.41
	**CD3D**	Avg.	—	—	—	**8.88**	—	—	—	—	—	—	—	—
		Prop.	—	—	—	**82.88**	—	—	—	—	—	—	—	—
Immune cell recruitment and interactions	**CCL19**	Avg.	—	—	—	—	—	—	—	—	2.22	—	—	—
		Prop.	—	—	—	—	—	—	—	—	5.21*	—	—	—
	**CXCR3**	Avg.	—	2.25	—	—	—	—	—	—	—	—	—	—
		Prop.	—	60.00	—	—	—	—	—	—	—	—	—	—
Immune cell recruitment and interactions	SYK	Avg.	—	1.37	—	—	—	—	—	—	—	—	—	—
		Prop.	—	49.47*	—	—	—	—	—	—	—	—	—	—
	**FCGR2A**	Avg.	1.33	—	—	—	—	—	—	—	—	—	—	—
		Prop.	44.00*	—	—	—	—	—	—	—	—	—	—	—
Immune cell recruitment and interactions	**VCAN**	Avg.	3.39	—	—	—	—	—	—	—	—	—	—	—
		Prop.	29.44*	—	—	—	—	—	—	—	—	—	—	—
	CD44	Avg.	3.47	1.04	1.35	2.78	1.34	—	5.76	—	—	—	—	—
		Prop.	68.70	41.05*	26.87*	49.05*	35.34*	—	67.14	—	—	—	—	—
Immune cell recruitment and interactions	**ANXA1**	Avg.	3.53	—	2.97	6.16	—	—	30.24	2.89	1.62	6.17	—	—
		Prop.	65.82	—	44.85*	63.73	—	—	88.57	54.36	31.93*	93.52	—	—
	FPR1	Avg.	1.11	—	—	—	—	—	—	—	—	—	—	—
		Prop.	32.91*	—	—	—	—	—	—	—	—	—	—	—
Immune cell recruitment and interactions	**ANXA1**	Avg.	3.53	—	2.97	6.16	—	—	30.24	2.89	1.62	6.17	—	—
		Prop.	65.82	—	44.85*	63.73	—	—	88.57	54.36	31.93*	93.52	—	—
	**FPR3**	Avg.	1.25	—	—	—	—	—	—	—	—	—	—	—
		Prop.	36.95*	—	—	—	—	—	—	—	—	—	—	—
Fibrosis	**PDGFD**	Avg.	—	—	—	—	—	—	—	—	—	—	—	1.04
		Prop.	—	—	—	—	—	—	—	—	—	—	—	37.87*
	**PDGFRA**	Avg.	—	—	—	—	—	—	—	—	—	0.91*	—	—
		Prop.	—	—	—	—	—	—	—	—	—	58.33	—	—
Endothelial regulation	MFGE8	Avg.	—	—	—	—	—	—	—	—	***6.61***	—	—	—
		Prop.	—	—	—	—	—	—	—	—	***75.54***	—	—	—
	**PDGFRB**	Avg.	—	—	—	—	—	—	—	—	***3.82***	—	—	—
		Prop.	—	—	—	—	—	—	—	—	***64.09***	—	—	—

Ligand-receptor pairs enriched in transcriptomic datasets were assessed for cell type-specific expression based on scRNASeq data.^59^ Ligands and receptors with an average (Avg) expression greater than 1 and proportional (Prop) expression in more than 50% of cells were used as initial cutoff criteria. Interactions between ligands and receptors within the same cell type are italic, as well as potential cell-to-cell communication where both average and proportional expression met the cutoff thresholds. Some ligands or receptors meet only one threshold and the values not meeting the criteria are indicated by (*). Dashes (-) indicate neither average or proportional expression meet criteria. Bolded gene symbols indicated differentially expressed genes between F3-4 versus F0-1 patients per our meta-analysis.

Similarly, we analyzed the expression of ligand–receptor pairs in liver cell subtypes using the criteria described above. Supplemental Table S10, http://links.lww.com/HC9/C419 illustrates the ligand–receptor pairs meeting our expression criteria as well as potential interactions between cell subtypes. One interaction of note is EFEMP2–AQP1. This pair is enriched across 10 datasets, and both genes increased with fibrosis stage in our meta-analysis. *EFEMP2* encodes fibulin, which functions in the assembly of elastic fibers and is expressed by myofibroblasts, while *AQP1* is expressed in endothelia and cholangiocytes. The mechanistic impact of this interaction is not well understood but could represent a mechanistic and targetable driver of fibrosis in MASLD patients. Expression values of the top ligand–receptor pairs that showed cell-specific expression in cell subtypes are included in Supplemental Table S11, http://links.lww.com/HC9/C393. The data matrix, enriched through meta-analysis, highlights evidence of expression and suggests potential cell-to-cell interaction networks.

## DISCUSSION

The increasing prevalence of MASLD and the development of fibrosis in these patients represent a major global health issue. Despite the large number of transcriptomic analyses and advances in scRNA-seq methods, understanding cell type-specific transcriptional signals in MASLD and MASH remains a challenge due to several factors, including patient heterogeneity, cohort composition, and staging of MASLD/MASH, the small number of patient samples used for scRNA-seq analysis, and technique variation during isolation, amplification, and sequencing. Our meta-analysis investigated changes in gene expression in almost 1000 patients with MASLD and identified 846 DEGs between patients with F3-4 fibrosis compared with F0–1, representing both consistent gene signatures and new therapeutic candidates (Figure 1B). This is exciting considering the broad composition of the patient cohorts used for the meta-analysis and highlights the potential impact of these genes on improving anti-fibrotic strategies. Deconvolution of transcriptomic data identified significant changes in the proportions of several hepatic cell types in advanced fibrosis (Figure 2C and Supplemental Table S8, http://links.lww.com/HC9/C393). Finally, we identified ligand–receptor pairs enriched across multiple RNA-seq datasets, which could drive fibrosis (Tables 2,3). We will discuss the top DEGs and pathways identified in our meta-analysis, focusing on those less characterized in fibrosis, as well as the implications of ligand–receptor interactions and cell-type-specific expression of these genes in MASLD-fibrosis.

### Meta-analysis revealed changes in amino acid regulation, ECM organization, and immune signaling associated with advanced fibrosis

Pathway enrichment analysis of DEGs from our meta-analysis revealed changes in several pathways known to be altered with fibrosis (ECM organization, angiogenesis, inflammation), consistent with published datasets. Other similarities included changes in p53-associated genes, and HCC (*GNMT*, *MAT1A*), gene signatures identified in previous studies.^5^^,^^6^^,^^12^ Excitingly, the meta-analysis also revealed changes in pathways not reported by previous analyses. These changes were primarily associated with downregulated genes, including alterations in amino acid biosynthesis and transport, bile acid transport, and metabolism associated with sulfur amino acids, nicotinate, and nicotinamide. Downregulated pathways are often overlooked in their roles as disease drivers and therapeutic targets, especially with MASLD patients typically exhibiting more upregulated than downregulated DEGs. The increase in patient samples allows for a robust analysis of downregulated genes and pathways, providing insight into fibrosis progression and treatment.

#### Regulation of amino acid biosynthesis, transportation, and metabolism: Potential implications for fibrosis and immune regulation

Numerous DEGs from our meta-analysis were identified as playing important roles in amino acid biosynthesis, metabolism, or transportation, including genes also implicated in immune homeostasis such as *SLC16A10, AADAT,* and *CNDP1* (Supplemental Figure S2, http://links.lww.com/HC9/C393). *SLC16A10/*TAT1 facilitates Na^+^-independent aromatic amino acid unipolar transportation and regulates hepatic homeostasis of tryptophan.^60^ Hepatic tryptophan affects immune homeostasis, evidenced by *SLC16A10* levels correlating with immune infiltration, while *Slc16a10* deficiency led to severe hypertryptophanemia and disrupted immune signaling in mice.^61^^,^^62^ *AADAT* is highly expressed in the liver, plays a role in immunity through mediating leukocyte recruitment, and has enzymatic activity involved in L-lysine and tryptophan catabolism.^39^^,^^63^^,^^64^ CNDP1 is the rate-limiting enzyme of carnosine hydrolysis and facilitates amino acid metabolism. Expression of *CNDP1* is associated with cirrhosis and HCC, with *CNDP1* identified as a potential marker for HCC.^65^^,^^66^ Interestingly, CNDP1 associates with SLC16A10, but the implication of this interaction is unclear.^67^ Thus, downregulation of *SLC16A10*, *CNDP1*, and *AADAT* may drive inflammation and fibrosis through disrupting amino acid homeostasis.

#### Differential regulation of genes involved in cholesterol transportation or signaling

Cholesterol dysregulation is a common feature of MASLD, with alterations in cholesterol levels, transport, and biosynthesis; however, it is unclear whether targeting these processes impacts fibrosis. Our meta-analysis revealed several DEGs associated with cholesterol in F3–4 fibrosis, which are understudied in fibrogenesis (upregulated: *GRAMD1B*; downregulated: *SLC13A5*, *ADCY10*, *NR5A2*, and *KANK4*). *GRAMD1B* is an Aster family protein that facilitates non-vesicular cholesterol movement between membranes and is responsible for modifying cholesterol distribution at ER–Golgi contact sites and within the endoplasmic reticulum (ER) and plasma membrane.^68^^–^^70^ *Gramd1b* loss protected mice from diet-induced hypercholesterolemia and was associated with modulating inflammatory responses in a human genome-wide association study (GWAS), further supporting pathogenic dysregulation.^70^^,^^71^ Downregulation of cholesterol-associated genes provides further mechanistic insight into how altered cholesterol metabolism contributes to MASLD-fibrosis. *SLC13A5* encodes a Na^+^-dependent citrate cotransporter highly expressed in the liver, with roles in fatty acid and cholesterol synthesis, citrate transport, and maintaining hepatic energy homeostasis.^72^^,^^73^ While *SLC13A5* expression reduced with advanced fibrosis here, *SLC13A5* was also identified as a driver of steatosis. Newly developed SLC13A5 inhibitors showed promise in treating hyperlipidemia, highlighting their potential targetability in MASLD; however, their role in MASH and fibrogenesis requires further study.^74^^–^^76^ Targeting these genes could restore key hepatic functions and limit fibrosis progression.

#### Chemokines and their receptors are key in immune regulation in fibrosis

Chemokines and their receptors are crucial regulators of cell migration and play key roles in recruiting various immune cells to sites of injury. Our meta-analysis revealed changes in inflammatory regulators from a gene expression perspective, as well as enriched ligand–receptor interactions that could represent novel therapeutic strategies for liver fibrosis. Inflammatory ligands *CCL19*, *CCL20*, and *CCL21* significantly increased in our meta-analysis. Previously, CCL21 and CCL19 were shown to modulate lymphocyte recruitment and fibrosis in patients with hepatitis C or inflammatory liver disease.^77^^,^^78^ The mechanisms of CCL19/CCL21 signaling in fibrosis may involve activation of extracellular signaling pathways, specifically Akt and NF-κB. Non-canonical NF-κB signaling was enriched in our meta-analysis and regulates genes encoding lymphoid-specific chemokines and cytokines such as CCL19 and CCL21.^79^^,^^80^

Chemokines could also influence fibrosis through dysregulation of platelet-mediated interactions. Upregulated genes associated with this pathway include *CCL5*, *CCL2*, and CD40 ligand (*CD40LG*), while *CCL2–CCR2* and *CCL2–CCR5* interactions were enriched (Table 2), coupled with predicted changes in *CCR5* signaling (Figure 1D). Targeting these pathways using cenicriviroc, a dual CCR2/CCR5 antagonist, had encouraging preclinical data and clinical results in phase II trials, but lacked efficacy in a phase III trial.^81^^,^^82^ *FCER1A*, the gene encoding FcεRI, is significantly downregulated in our F3–4 group. FcεRI aggregation leads to phosphorylation of LAT2, mast cell degranulation, and impaired autoimmune and T-cell regulation. In this way, loss of FcεRI could limit immune suppression and promote inflammation and fibrosis.^83^^,^^84^ Additional studies could provide crucial insight into how FcεRI, LAT2, and CCL2/CCR5 signaling influence fibrosis in MASLD patients.

### Dysregulated genes and their impact on cell–cell interactions may drive fibrosis progression

To gain additional insight into how DEGs identified in our meta-analysis regulate fibrosis, we performed ligand–receptor analysis on the transcriptomic datasets. Several ligand–receptor interactions involving DEGs from the meta-analysis were enriched in the datasets, highlighting their potential role in pro-fibrotic signaling. Furthermore, insight into cell-specific expression of these genes allows for inference into cell–cell interactions.

Alterations in ECM organization are a hallmark of fibrosis. Consistent with this, many ECM organizations and collagen-associated genes were differentially expressed in advanced fibrosis (Supplemental Figure S1, http://links.lww.com/HC9/C393). Several of these genes strongly correlate with steatohepatitis and/or fibrosis; however, less is understood regarding their cell-specific expression and interactions. *THBS2* is an established driver of HSC activation and fibrosis.^26^^,^^27^^,^^57^^,^^85^ Our ligand–receptor analysis revealed that THBS2 interacting with either integrin subunit alpha 6 (ITGA6, expressed by endothelial subtypes) or NOTCH3 may be important in fibrosis progression. A number of other interactions were identified where both ligand and receptor were increased with advanced fibrosis, including COL4A1–ITGAV, COL4A4–ITGAV, FBN1–ITGAV, EFEMP2–AQP1, and MMP7–CDH6 (Table 2). Studies previously highlighted the importance of ITGAV in MASH-fibrosis, with ITGAV levels positively correlating with disease progression and ITGAV inhibition blocking HSC activation;^86^ however, the impact of ITGAV interacting with collagen 4 isoforms or FBN1 in liver disease is unknown. An additional pair that merits discussion is EFEMP2–AQP1. *EFEMP2/*fibulin-4 is a critical component of elastic fibers and a regulator of ECM organization. *EFEMP2* increased in F3–4 livers and was enriched in myofibroblasts. It binds several proteins relevant to fibrogenesis, including LOXL1 and LTBP-4L (latent TGF-binding protein-4), and can regulate TGFβ activity.^87^^–^^89^ *AQP1* encodes aquaporin, a water channel expressed in endothelial cells and cholangiocytes, which acts in capillary proliferation and bile secretion.^90^^,^^91^ The impact of an aquaporin–fibulin-4 interaction is unclear but could drive fibrosis through regulating AQP1 activity and endothelial cell or cholangiocyte function.

Other enriched ligand–receptor interactions involving the ECM displayed increased ligand expression, potentially indicating increased signaling through cell–cell adhesion. These ligands include vimentin (VIM), nephronectin (NPNT), FBN1, versican (VCAN), LUM, and several collagen and laminin isoforms. Increased ligand interactions with integrins and cell surface adhesion receptors could alter matrix composition and stiffness, as well as immune cell recruitment, even without increased expression of their receptors, thus representing potential anti-fibrotic targets.

## IMPACT OF COVARIATES WITHIN THE META-ANALYSIS

Comparing the meta-analysis to those identified in individual datasets, we found that only 8 out of the 846 DEGs from the meta-analysis were also identified as DEGS in all of the analyzed datasets. This highlights the statistical power of the meta-analysis as well as the heterogeneity of the cohorts. Some datasets had lower power due to fewer samples (eg, GSE96971), while others (ie, the pediatric cohort) showed differences that could be attributed to the composition of the cohort. This highlights a few important points, the first being the impact of sample size and the benefits of performing meta-analyses to gain insight into heterogeneous diseases such as MASLD. Second, the pediatric cohort displayed the least similarity to our meta-analysis. Less than half of the top 100 upregulated genes were also significantly changed in the pediatric cohort. This could be influenced by the small number of patients with advanced fibrosis (5); however, GSE96971 contained only 7 samples from adults with F3–4 and showed significant changes in 63/100 upregulated genes. Consistent with unique changes in the pediatric cohort, prior analysis of this dataset showed some correlation with adult patients (changes in fibrotic and inflammatory signaling pathways), but key differences, including enrichment of DEGs associated with mitochondrial dysfunction, oxidative phosphorylation, and EIF2 signaling.^8^

Other important covariates to consider are genetics, diabetes, body mass index, lifestyle factors, and more.^92^^–^^94^ Historically, many studies included only males, leading to biased translation of findings and therapeutic strategies that worked poorly among women.^95^ To gain insight into whether gender impacted our meta-analysis, we compared DEGs between cohorts that had similar numbers of patients, but consisted of more female or male patients, and did not observe overt differences between cohorts with more male versus female patients. Additional analysis to determine whether sex, diabetes, or medications taken at the time of biopsy would be impactful in the future. As more transcriptomic analyses are performed, we will continue to update our meta-analysis cohort to gain more insight into these factors.

## FUTURE DIRECTIONS

The DEGs identified in our meta-analysis, particularly those encoding secreted proteins (Supplemental Table S4, http://links.lww.com/HC9/C393), should be investigated as potential biomarker signatures to predict fibrosis stage in patients. From both gene and protein expression studies, signature genes could be grouped and evaluated as components of a polygenic risk score for liver fibrosis. Furthermore, understanding cell–cell interactions by obtaining scRNA-seq and snRNA-seq data from a wider range of patients and combining single-cell technologies with spatial transcriptomics will enable further studies on the dynamic change in molecular regulation between cells during fibrosis progression. This will help to overcome the bias of scRNA-seq toward immune cell populations by allowing deeper insight into the transcriptomic changes occurring in hepatocytes and mesenchymal cells. Current research has identified cell-specific markers and ligand–receptor axes as new targets for future therapeutic development. In all, our approach strengthens the understanding of liver fibrosis development at the cellular level and future molecular diagnosis and intervention.

## Supplementary Material
