# Supplementary material for: Conserved hepatic RNA signatures across multiple cohorts reveal novel mechanistic clues for advanced fibrosis in human MASLD
Source: Hepatol Commun. 2026 Sep 16;10(10):e0991. doi: 10.1097/HC9.0000000000000991 (PMC13585325; doi:10.1097/HC9.0000000000000991)
Supplement: Supplementary file 1 [file hc9-10-e0991-s001.docx]

**Table S10. ExorLigand and receptor average and proportional expression in cell subtypes.**

| **Process** | **Ligand Receptor** | **Expression** | **Tmo_1_** | **Tmo_2_** | **Tmo_3_** | **SAMac_1_** | **SAMac_2_** | **KC_1_** | **KC_2_** | **CDC_1_** | **CDC_2_** | **Cycling MP_1_** | **Cycling MP_2_** | **Cycling MP_3_** | **Cycling MP_4_** | **pDC** | **NK_1_** | **NK_2_** | **NK_3_** | **Cycling NK_1_** | **Cycling NK_1_** | **CD4+ T cell_1_** | **CD4+ T cell_2_** | **CD8+ T cell_1_** | **CD8+ T cell_2_** | **CD8+ T cell_3_** | **Cycling T cell** | **B cell_1_** | **B cell_2_** | **Plasma B cell_1_** | **Plasma B cell_2_** | **Mast cell** | **LSEC** | **Lymphatic Endothelia** | **MV Endothelia (PV)** | **MV Endothelia (CV)** | **MV Endothelia (HA)** | **SA Endothelia_1_** | **SA Endothelia_2_** | **VSMC** | **HSC** | **Myofibroblast** | **Mesothelia** | **Hepatocyte** | **Cholangiocyte_1_** | **Cholangiocyte_2_** | **Cholangiocyte_3_** |
| --- | --- | --- | --- | --- | --- | --- | --- | --- | --- | --- | --- | --- | --- | --- | --- | --- | --- | --- | --- | --- | --- | --- | --- | --- | --- | --- | --- | --- | --- | --- | --- | --- | --- | --- | --- | --- | --- | --- | --- | --- | --- | --- | --- | --- | --- | --- | --- |
| **Immune cell interactions** | SELPLG | Avg. | - | - | - | - | - | - | - | - | - | - | - | <1* | - | **2** | - | - | 1 | - | 1 | - | - | - | - | - | - | - | - | - | - | - | - | - | - | - | - | - | - | - | - | - | - | - | - | - | - |
|  |  | Prop. | - | - | - | - | - | - | - | - | - | - | - | 57 | - | **56** | - | - | 28* | - | 46* | - | - | - | - | - | - | - | - | - | - | - | - | - | - | - | - | - | - | - | - | - | - | - | - | - | - |
|  | SELL | Avg. | 3 | 1 | - | - | - | - | - | - | - | - | - | 1 | - | **4** | - | - | 1 | - | 2 | - | 1 | - | - | - | - | 2 | 3 | - | - | - | - | - | - | - | - | - | - | - | - | - | - | - | - | - | - |
|  |  | Prop. | 46* | 18* | - | - | - | - | - | - | - | - | - | 82 | - | **84** | - | - | 21* | - | 41* | - | 23* | - | - | - | - | 49* | 53 | - | - | - | - | - | - | - | - | - | - | - | - | - | - | - | - | - | - |
|  | **VCAN** | Avg. | **21** | 7 | 1 | 2 | - | - | - | - | - | - | - | - | - | - | - | - | - | - | - | - | - | - | - | - | - | - | - | - | - | - | - | - | - | - | - | - | - | - | - | 2 | - | - | - | - | - |
|  |  | Prop. | **94** | 49* | 34* | 46* | - | - | - | - | - | - | - | - | - | - | - | - | - | - | - | - | - | - | - | - | - | - | - | - | - | - | - | - | - | - | - | - | - | - | - | 46* | - | - | - | - | - |
|  | CD44 | Avg. | **8** | 5 | 3 | 3 | 2 | 3 | 5 | 3 | 1 | 2 | 3 | <1* | 3 | 1 | 2 | 1 | 1 | 1 | 1 | 3 | 3 | 3 | 3 | 2 | 3 | 2 | - | - | <1* | 6 | - | - | - | - | - | - | - | - | - | - | - | - | - | - | - |
|  |  | Prop. | **80** | 52 | 76 | 70 | 60 | 79 | 62 | 78 | 43* | 77 | 86 | 57 | 50 | 41* | 17* | 32* | 26* | 40* | 35* | 36* | 61 | 32* | 53 | 48* | 70 | 42* | - | - | 52 | 67 | - | - | - | - | - | - | - | - | - | - | - | - | - | - | - |
|  | **COL4A1** | Avg. | - | - | - | - | - | - | - | - | - | - | - | - | - | - | - | - | - | - | - | - | - | - | - | - | - | - | - | - | - | - | **3** | - | - | - | 1 | **4** | 2 | - | - | **2** | - | - | - | - | - |
|  |  | Prop. | - | - | - | - | - | - | - | - | - | - | - | - | - | - | - | - | - | - | - | - | - | - | - | - | - | - | - | - | - | - | **59** | - | - | - | 41* | **74** | 46* | - | - | **54** | - | - | - | - | - |
|  | ITGB1 | Avg. | - | 1 | 1 | - | 1 | 1 | 2 | <1* | - | <1* | 1 | 1 | - | - | 1 | 1 | 1 | 1 | 2 | - | 1 | - | - | - | 1 | - | - | - | - | - | **5** | 4 | 4 | 3 | 5 | **4** | 3 | 5 | 6 | **5** | 2 | 2 | 4 | 3 | 5 |
|  |  | Prop. | - | 21* | 49* | - | 52 | 60 | 33* | 54 | - | 56 | 70 | 68 | - | - | 15* | 29* | 29* | 49* | 53 | - | 33* | - | - | - | 44* | - | - | - | - | - | **78** | 78 | 76 | 84 | 82 | **78** | 69 | 69 | 82 | **84** | 81 | 78 | 59 | 65 | 87 |
| **Unknown** | **EFEMP2** | Avg. | - | - | - | - | - | - | - | - | - | - | - | - | - | - | - | - | - | - | - | - | - | - | - | - | - | - | - | - | - | - | - | - | - | - | - | - | - | - | - | 2 | **2** | - | - | - | - |
|  |  | Prop. | - | - | - | - | - | - | - | - | - | - | - | - | - | - | - | - | - | - | - | - | - | - | - | - | - | - | - | - | - | - | - | - | - | - | - | - | - | - | - | 71 | **77** | - | - | - | - |
|  | **AQP1** | Avg. | - | - | - | - | - | - | - | - | - | - | - | - | - | - | - | - | - | - | - | - | - | - | - | - | - | - | - | - | - | - | - | - | 5 | - | 5 | 4 | 4 | - | - | - | **3** | - | 3 | 3 | 5 |
|  |  | Prop. | - | - | - | - | - | - | - | - | - | - | - | - | - | - | - | - | - | - | - | - | - | - | - | - | - | - | - | - | - | - | - | - | 83 | - | 74 | 60 | 74 | - | - | - | **88** | - | 52 | 67 | 81 |
| I**mmune cell recruitment** | SYK | Avg. | - | - | - | - | - | - | - | - | - | <1* | **<1*** | <1* | - | 1 | - | - | - | - | - | - | - | - | - | - | - | - | - | - | - | - | - | - | - | - | - | - | - | - | - | - | - | - | - | - | - |
|  |  | Prop. | - | - | - | - | - | - | - | - | - | 57 | **73** | 64 | - | 49* | - | - | - | - | - | - | - | - | - | - | - | - | - | - | - | - | - | - | - | - | - | - | - | - | - | - | - | - | - | - | - |
|  | **FCGR2A** | Avg. | 1 | - | 1 | 1 | 2 | 1 | - | 2 | - | 1 | **2** | - | - | - | - | - | - | - | - | - | - | - | - | - | - | - | - | - | - | - | - | - | - | - | - | - | - | - | - | - | - | - | - | - | - |
|  |  | Prop. | 30* | - | 48* | 47* | 69 | 48* | - | 64 | - | 69 | **84** | - | - | - | - | - | - | - | - | - | - | - | - | - | - | - | - | - | - | - | - | - | - | - | - | - | - | - | - | - | - | - | - | - | - |
|  | **ANXA1** | Avg. | **7** | 3 | 3 | 3 | 3 | 1 | 3 | 5 | 3 | 6 | 5 | 2 | 3 | - | 1 | 2 | 5 | 2 | 5 | 5 | 8 | 4 | 6 | 4 | 5 | - | - | - | - | 30 | - | 3 | 3 | - | 3 | 3 | 4 | - | 1 | 5 | 6 | - | - | - | - |
|  |  | Prop. | **75** | 37* | 68 | 71 | 73 | 53 | 40* | 90 | 57 | 93 | 91 | 79 | 58 | - | 17* | 40* | 66 | 53 | 83 | 47* | 80 | 32* | 73 | 76 | 72 | - | - | - | - | 89 | - | 54 | 52 | - | 62 | 62 | 76 | - | 33* | 76 | 94 | - | - | - | - |
|  | FPR1 | Avg. | **3** | 2 | 1 | 1 | - | - | - | - | - | - | 1 | - | - | - | - | - | - | - | - | - | - | - | - | - | - | - | - | - | - | - | - | - | - | - | - | - | - | - | - | - | - | - | - | - | - |
|  |  | Prop. | **53** | 23* | 49* | 42* | - | - | - | - | - | - | 48* | - | - | - | - | - | - | - | - | - | - | - | - | - | - | - | - | - | - | - | - | - | - | - | - | - | - | - | - | - | - | - | - | - | - |
| **Immune cell signaling** | **ANXA1** | Avg. | 7 | 3 | 3 | 3 | **3** | **1** | 3 | 5 | 3 | 6 | 5 | 2 | 3 | - | 1 | 2 | 5 | 2 | 5 | 5 | 8 | 4 | 6 | 4 | 5 | - | - | - | - | 30 | - | 3 | 3 | - | 3 | 3 | 4 | - | 1 | 5 | 6 | - | - | - | - |
|  |  | Prop. | 75 | 37* | 68 | 71 | **73** | **53** | 40* | 90 | 57 | 93 | 91 | 79 | 58 | - | 17* | 40* | 66 | 53 | 83 | 47* | 80 | 32* | 73 | 76 | 72 | - | - | - | - | 89 | - | 54 | 52 | - | 62 | 62 | 76 | - | 33* | 76 | 94 | - | - | - | - |
|  | **FPR3** | Avg. | - | - | - | - | **2** | **3** | 4 | - | - | - | - | - | - | - | - | - | - | - | - | - | - | - | - | - | - | - | - | - | - | - | - | - | - | - | - | - | - | - | - | - | - | - | - | - | - |
|  |  | Prop. | - | - | - | - | **61** | **76** | 56 | - | - | - | - | - | - | - | - | - | - | - | - | - | - | - | - | - | - | - | - | - | - | - | - | - | - | - | - | - | - | - | - | - | - | - | - | - | - |
|  | **LUM** | Avg. | - | - | - | - | - | - | - | - | - | - | - | - | - | - | - | - | - | - | - | - | - | - | - | - | - | - | - | - | - | - | - | - | - | - | - | - | - | - | - | **24** | - | - | - | - | - |
|  |  | Prop. | - | - | - | - | - | - | - | - | - | - | - | - | - | - | - | - | - | - | - | - | - | - | - | - | - | - | - | - | - | - | - | - | - | - | - | - | - | - | - | **88** | - | - | - | - | - |
|  | ITGB1 | Avg. | - | 1 | 1 | - | 1 | 1 | 2 | <1* | - | <1* | 1 | 1 | - | - | 1 | 1 | 1 | 1 | 2 | - | 1 | - | - | - | 1 | - | - | - | - | - | 5 | 4 | 4 | 3 | 5 | 4 | 3 | 5 | 6 | **5** | 2 | 2 | 4 | 3 | 5 |
|  |  | Prop. | - | 21* | 49* | - | 52 | 60 | 33* | 54 | - | 56 | 70 | 68 | - | - | 15* | 29* | 29* | 49* | 53 | - | 33* | - | - | - | 44* | - | - | - | - | - | 78 | 78 | 76 | 84 | 82 | 78 | 69 | 69 | 82 | **84** | 81 | 78 | 59 | 65 | 87 |
|  | **SPP1** | Avg. | - | - | - | - | 3 | - | - | - | - | - | **20** | - | - | - | - | - | - | - | - | - | - | - | - | - | - | - | - | - | - | - | - | - | - | - | - | - | - | - | - | - | - | 3 | 6 | 19 | 15 |
|  |  | Prop. | - | - | - | - | 16* | - | - | - | - | - | **52** | - | - | - | - | - | - | - | - | - | - | - | - | - | - | - | - | - | - | - | - | - | - | - | - | - | - | - | - | - | - | 47* | 57 | 90 | 86 |
|  | CD44 | Avg. | 8 | 5 | 3 | 3 | 2 | 3 | 5 | 3 | 1 | 2 | **3** | <1* | 3 | 1 | 2 | 1 | 1 | 1 | 1 | 3 | 3 | 3 | 3 | 2 | 3 | 2 | - | - | <1* | 6 | - | - | - | - | - | - | - | - | - | - | - | - | - | - | - |
|  |  | Prop. | 80 | 52 | 76 | 70 | 60 | 79 | 62 | 78 | 43* | 77 | **86** | 57 | 50 | 41* | 17* | 32* | 26* | 40* | 35* | 36* | 61 | 32* | 53 | 48* | 70 | 42* | - | - | 52 | 67 | - | - | - | - | - | - | - | - | - | - | - | - | - | - | - |
| **Immune cell signaling (cont.)** | **SPP1** | Avg. | - | - | - | - | 3 | - | - | - | - | - | **20** | - | - | - | - | - | - | - | - | - | - | - | - | - | - | - | - | - | - | - | - | - | - | - | - | - | - | - | - | - | - | 3 | **6** | **19** | **15** |
|  |  | Prop. | - | - | - | - | 16* | - | - | - | - | - | **52** | - | - | - | - | - | - | - | - | - | - | - | - | - | - | - | - | - | - | - | - | - | - | - | - | - | - | - | - | - | - | 47* | **57** | **90** | **86** |
|  | ITGB1 | Avg. | - | 1 | 1 | - | 1 | 1 | 2 | <1* | - | <1* | **1** | 1 | - | - | 1 | 1 | 1 | 1 | 2 | - | 1 | - | - | - | 1 | - | - | - | - | - | 5 | 4 | 4 | 3 | 5 | 4 | 3 | 5 | 6 | 5 | 2 | 2 | **4** | **3** | **5** |
|  |  | Prop. | - | 21* | 49* | - | 52 | 60 | 33* | 54 | - | 56 | **70** | 68 | - | - | 15* | 29* | 29* | 49* | 53 | - | 33* | - | - | - | 44* | - | - | - | - | - | 78 | 78 | 76 | 84 | 82 | 78 | 69 | 69 | 82 | 84 | 81 | 78 | **59** | **65** | **87** |
|  | **CD40LG** | Avg. | - | - | - | - | - | - | - | - | - | - | - | - | - | - | - | - | - | - | - | 2 | 2 | - | - | **2** | - | - | - | - | - | - | - | - | - | - | - | - | - | - | - | - | - | - | - | - | - |
|  |  | Prop. | - | - | - | - | - | - | - | - | - | - | - | - | - | - | - | - | - | - | - | 25* | 43* | - | - | **52** | - | - | - | - | - | - | - | - | - | - | - | - | - | - | - | - | - | - | - | - | - |
|  | ITGB2 | Avg. | 5 | 4 | 5 | 5 | 4 | 2 | 2 | 5 | 4 | 5 | 4 | 4 | 1 | 1 | 6 | 5 | 8 | 4 | 7 | 2 | 2 | 4 | 4 | **2** | 4 | 1 | - | - | <1* | - | - | - | - | - | - | - | - | - | - | - | - | - | - | - | - |
|  |  | Prop. | 70 | 54 | 87 | 83 | 82 | 65 | 33* | 91 | 72 | 93 | 98 | 89 | 33* | 44* | 54 | 76 | 81 | 82 | 88 | 31* | 53 | 40* | 68 | **60** | 76 | 30* | - | - | 51 | - | - | - | - | - | - | - | - | - | - | - | - | - | - | - | - |
|  | MFGE8 | Avg. | - | - | - | - | - | - | - | - | - | - | - | - | - | - | - | - | - | - | - | - | - | - | - | - | - | - | - | - | - | - | - | - | - | - | - | - | - | **9** | **6** | 1 | - | - | - | - | - |
|  |  | Prop. | - | - | - | - | - | - | - | - | - | - | - | - | - | - | - | - | - | - | - | - | - | - | - | - | - | - | - | - | - | - | - | - | - | - | - | - | - | **82** | **80** | 42* | - | - | - | - | - |
|  | **PDGFRB** | Avg. | - | - | - | - | - | - | - | - | - | - | - | - | - | - | - | - | - | - | - | - | - | - | - | - | - | - | - | - | - | - | - | - | - | - | - | - | - | **5** | **4** | 2 | - | - | - | - | - |
|  |  | Prop. | - | - | - | - | - | - | - | - | - | - | - | - | - | - | - | - | - | - | - | - | - | - | - | - | - | - | - | - | - | - | - | - | - | - | - | - | - | **62** | **73** | 48* | - | - | - | - | - |
|  | **VIM** | Avg. | **18** | **9** | **10** | **11** | **11** | **10** | **15** | **18** | 14 | **23** | **20** | 25 | **12** | 11 | 1 | 3 | 3 | 9 | 11 | 6 | **11** | 4 | **9** | 8 | **15** | 5 | 3 | 6 | 3 | **17** | 3 | 58 | 37 | 8 | 50 | 31 | 48 | 30 | 29 | 35 | 15 | 1 | 4 | 3 | 6 |
|  |  | Prop. | **93** | **68** | **96** | **93** | **91** | **98** | **87** | **98** | 87 | **99** | **100** | 100 | **92** | 96 | 15* | 48* | 47* | 85 | 91 | 54 | **90** | 37* | **85** | 88 | **91** | 72 | 53 | 93 | 91 | **87** | 41* | 100 | 98 | 84 | 99 | 98 | 100 | 95 | 97 | 100 | 97 | 59 | 57 | 63 | 84 |
|  | CD44 | Avg. | **8** | **5** | **3** | **3** | **2** | **3** | **5** | **3** | 1 | **2** | **3** | <1* | **3** | 1 | 2 | 1 | 1 | 1 | 1 | 3 | **3** | 3 | **3** | 2 | **3** | 2 | - | - | <1* | **6** | - | - | - | - | - | - | - | - | - | - | - | - | - | - | - |
|  |  | Prop. | **80** | **52** | **76** | **70** | **60** | **79** | **62** | **78** | 43* | **77** | **86** | 57 | **50** | 41* | 17* | 32* | 26* | 40* | 35* | 36* | **61** | 32* | **53** | 48* | **70** | 42* | - | - | 52 | **67** | - | - | - | - | - | - | - | - | - | - | - | - | - | - | - |
|  | HLA-A | Avg. | 14 | 13 | 20 | 18 | 23 | 21 | 15 | 18 | 15 | 16 | 18 | 20 | 14 | 14 | 33 | 39 | 47 | 27 | 39 | **29** | **32** | **39** | **45** | **38** | **35** | 23 | 18 | 8 | 7 | 13 | 32 | 23 | 25 | 33 | 33 | 33 | 27 | 5 | 8 | 12 | 8 | 9 | 10 | 14 | 15 |
|  |  | Prop. | 90 | 82 | 99 | 97 | 99 | 100 | 90 | 99 | 94 | 99 | 100 | 100 | 100 | 97 | 96 | 100 | 99 | 99 | 99 | **95** | **100** | **97** | **100** | **100** | **100** | 98 | 96 | 95 | 96 | 80 | 99 | 100 | 100 | 100 | 100 | 99 | 99 | 69 | 89 | 94 | 96 | 96 | 80 | 95 | 98 |
|  | **CD3D** | Avg. | - | - | - | - | - | - | - | - | - | - | - | - | - | - | 1 | - | - | - | - | **8** | **8** | **10** | **10** | **9** | **10** | - | - | - | <1* | - | - | - | - | - | - | - | - | - | - | - | - | - | - | - | - |
|  |  | Prop. | - | - | - | - | - | - | - | - | - | - | - | - | - | - | 12* | - | - | - | - | **72** | **88** | **67** | **92** | **94** | **91** | - | - | - | 54 | - | - | - | - | - | - | - | - | - | - | - | - | - | - | - | - |
|  | HLA-B | Avg. | 23 | 26 | 42 | 35 | 42 | 47 | 33 | 33 | 33 | 30 | 41 | 43 | 30 | 39 | 47 | 61 | 71 | 44 | 64 | **45** | **52** | **53** | **71** | **73** | **50** | 44 | 33 | 22 | 25 | 17 | 51 | 23 | 46 | 60 | 64 | 56 | 40 | 7 | 12 | 14 | 14 | 10 | 12 | 10 | 17 |
|  |  | Prop. | 96 | 97 | 100 | 100 | 100 | 100 | 97 | 100 | 98 | 100 | 100 | 100 | 100 | 99 | 98 | 100 | 100 | 100 | 100 | **99** | **100** | **99** | **100** | **100** | **100** | 99 | 99 | 99 | 100 | 91 | 100 | 99 | 100 | 100 | 100 | 100 | 100 | 74 | 93 | 97 | 99 | 97 | 86 | 91 | 98 |
|  | **CD3D** | Avg. | - | - | - | - | - | - | - | - | - | - | - | - | - | - | 1 | - | - | - | - | **8** | **8** | **10** | **10** | **9** | **10** | - | - | - | <1* | - | - | - | - | - | - | - | - | - | - | - | - | - | - | - | - |
|  |  | Prop. | - | - | - | - | - | - | - | - | - | - | - | - | - | - | 12* | - | - | - | - | **72** | **88** | **67** | **92** | **94** | **91** | - | - | - | 54 | - | - | - | - | - | - | - | - | - | - | - | - | - | - | - | - |
|  | B2M | Avg. | 114 | 108 | 166 | 139 | 173 | 249 | 200 | 125 | 108 | 120 | 133 | 99 | 264 | 127 | 161 | 167 | 243 | 137 | 200 | **184** | **202** | **213** | **234** | **204** | **181** | 135 | 95 | 90 | 77 | 139 | 175 | 87 | 157 | 190 | 176 | 156 | 123 | 42 | 58 | 63 | 51 | 35 | 40 | 40 | 50 |
|  |  | Prop. | 100 | 100 | 100 | 100 | 100 | 100 | 100 | 100 | 100 | 100 | 100 | 100 | 100 | 100 | 100 | 100 | 100 | 100 | 100 | **100** | **100** | **100** | **100** | **100** | **100** | 100 | 100 | 100 | 100 | 100 | 100 | 100 | 100 | 100 | 100 | 100 | 100 | 99 | 100 | 100 | 100 | 100 | 99 | 100 | 100 |
|  | **CD3D** | Avg. | - | - | - | - | - | - | - | - | - | - | - | - | - | - | 1 | - | - | - | - | **8** | **8** | **10** | **10** | **9** | **10** | - | - | - | <1* | - | - | - | - | - | - | - | - | - | - | - | - | - | - | - | - |
|  |  | Prop. | - | - | - | - | - | - | - | - | - | - | - | - | - | - | 12* | - | - | - | - | **72** | **88** | **67** | **92** | **94** | **91** | - | - | - | 54 | - | - | - | - | - | - | - | - | - | - | - | - | - | - | - | - |
|  | **CCL19** | Avg. | - | - | - | - | - | - | - | - | - | - | - | - | - | - | - | - | - | - | - | - | - | - | - | - | - | - | - | - | - | - | - | - | - | - | - | - | - | - | - | 13 | - | - | - | - | - |
|  |  | Prop. | - | - | - | - | - | - | - | - | - | - | - | - | - | - | - | - | - | - | - | - | - | - | - | - | - | - | - | - | - | - | - | - | - | - | - | - | - | - | - | 20* | - | - | - | - | - |
|  | **CXCR3** | Avg. | - | - | - | - | - | - | - | - | - | - | - | - | - | 2 | - | - | - | - | - | - | 1 | - | 1 | - | 2 | - | - | - | - | - | - | - | - | - | - | - | - | - | - | - | - | - | - | - | - |
|  |  | Prop. | - | - | - | - | - | - | - | - | - | - | - | - | - | 60 | - | - | - | - | - | - | 31* | - | 30* | - | 47* | - | - | - | - | - | - | - | - | - | - | - | - | - | - | - | - | - | - | - | - |
| **ECM** | **MMP7** | Avg. | - | - | - | - | - | - | - | - | - | - | - | - | - | - | - | - | - | - | - | - | - | - | - | - | - | - | - | - | - | - | - | - | - | - | - | - | - | - | - | - | - | - | 2 | 5 | 7 |
|  |  | Prop. | - | - | - | - | - | - | - | - | - | - | - | - | - | - | - | - | - | - | - | - | - | - | - | - | - | - | - | - | - | - | - | - | - | - | - | - | - | - | - | - | - | - | 36* | 70 | 59 |
|  | **CDH6** | Avg. | - | - | - | - | - | - | - | - | - | - | - | - | - | - | - | - | - | - | - | - | - | - | - | - | - | - | - | - | - | - | - | - | - | - | - | - | - | 2 | - | - | - | - | - | - | - |
|  |  | Prop. | - | - | - | - | - | - | - | - | - | - | - | - | - | - | - | - | - | - | - | - | - | - | - | - | - | - | - | - | - | - | - | - | - | - | - | - | - | 35* | - | - | - | - | - | - | - |
| **Fibrosis** | **PDGFD** | Avg. | - | - | - | - | - | - | - | - | - | - | - | - | - | - | - | - | - | - | - | - | - | - | - | - | - | - | - | - | - | - | - | - | - | - | - | - | - | - | - | - | - | - | 1 | - | 1 |
|  |  | Prop. | - | - | - | - | - | - | - | - | - | - | - | - | - | - | - | - | - | - | - | - | - | - | - | - | - | - | - | - | - | - | - | - | - | - | - | - | - | - | - | - | - | - | 28* | - | 45* |
|  | **PDGFRA** | Avg. | - | - | - | - | - | - | - | - | - | - | - | - | - | - | - | - | - | - | - | - | - | - | - | - | - | - | - | - | - | - | - | - | - | - | - | - | - | - | - | 2 | <1* | - | - | - | - |
|  |  | Prop. | - | - | - | - | - | - | - | - | - | - | - | - | - | - | - | - | - | - | - | - | - | - | - | - | - | - | - | - | - | - | - | - | - | - | - | - | - | - | - | 60 | 58 | - | - | - | - |

Ligands and receptors with an average (Avg) expression greater than 1 and proportional (Prop) expression in more than 50% of cells were used as cutoff criteria. Potential interactions between ligands and receptors within the same cell type were highlighted (boxed). Some ligands or receptors meet only one threshold, and the values not meeting the criteria are indicated by (*). Dashes (-) indicate neither average nor proportional expression meet criteria. Bolded gene symbols are differentially expressed status between F3-4 vs F0-1 per our meta-analysis. Full data are included in supplemental material (Table S11).
