# Supplementary material for: Conserved hepatic RNA signatures across multiple cohorts reveal novel mechanistic clues for advanced fibrosis in human MASLD
Source: Hepatol Commun. 2026 Sep 16;10(10):e0991. doi: 10.1097/HC9.0000000000000991 (PMC13585325; doi:10.1097/HC9.0000000000000991)
Supplement: Supplementary file 2 [file hc9-10-e0991-s002.docx]

**Supplementary Material**

Table of contents

Supplementary methods…………………………………………………………………….….….…...........………2 - 5

Supplemental Figure 1…………………………………………………………………………………………...........…6

Supplemental Figure 2………………………………………………………………………………………...........……7

Supplemental Figure 3……………………………………………………………………………………………...........8

Supplementary Table 1…………………………………………………………………….….….…………..........9 – 36

Supplementary Table 2……………………………………………………………………………………...........37 – 40

Supplementary Table 3………………………………………………………………………….……………..............41

Supplementary Table 4……………………………………………………………………………….……....…..........42

Supplementary Table 5……………………………………………………………………………………….…...........43

Supplementary Table 6……………………………………………………………………………………...........44 – 45

Supplementary Table 7……………………………………………………………………………….…….….............46

Supplementary Table 8………………………………………………………………………………….....……..........47

Supplementary Table 9…………………………………………………………………………...………...........48 – 49

Supplementary Table 11…..……………………………………………………………………….…….............50 – 51

References…………………………………………………………………………………………………...........52 – 58

**Supplementary Methods**

***Differential expression analysis for single cell RNAseq dataset***

To identify marker genes specific to the cell types of interest, deconvolution was conducted after thorough differential gene expression analysis. Initially, the liver organ scRNA-sequencing dataset was annotated with 12 major hepatic cell types: Mononuclear Phagocytes (MPs), plasmacytoid Dendritic Cells (pDCs), Innate Lymphoid Cells (ILCs), T cells, B cells, plasma B cells, Mast Cells, Endothelial cells, Mesenchymal cells, Hepatocytes, and Cholangiocytes. To provide additional refinement, the 'FindAllMarkers()' function in R was employed to aid in this intricate analysis. The focus of this study are genes with a positive log2 fold change exceeding 0.25 and set 'min.pct' (minimum percentage of cells) to >25% to exclude genes with minimal expression. Subsequently, the number of differentially expressed genes was refined to the top 50, and unique genes among the cell types were identified. The resulting list of genes represents the core markers that are exclusively expressed in a specific cell type among the 39 cell types of interest.

These major cell types were further separated into subtypes. MP subtypes: Tissue monocyte (Tmo)_1, 2, & 3_, Scar associated macrophage (SAMac_1 & 2_), Kupffer cell (KC)_1 & 2_, classical dendritic cells (cDCs)_1 & 2_, and plasmacytoid dendritic cells (pDCs); ILC subtypes: Natural killer (NK) cells_1 & 2_, cycling NK cells (cNK), and Cycling ILCs. T-cell subtypes: CD4+ T cell_1 & 2_, CD8+ T cell_1, 2, & 3_, and Cycling T cells. B cell subtypes: B cell_1 &2_; Plasma B cell subtypes: Plasma B cell_1 &2_. Endothelial cell subtypes: Liver sinusoidal endothelial cells (LSEC), Lymphatic endothelia, microvascular endothelial cells (portal vein, central vein, and hepatic artery), and scar-associated endothelial cells (SAendo_1 & 2_). Mesenchymal cell subtypes: vascular smooth muscle cells (VSMC), HSCs, Scar-Associated Mesenchymal cells (SAMes), Myofibroblasts. Cholangiocyte subtypes: Cholangiocyte_1, 2, & 3_. Other cell types can function as progenitor cells: Mast cells and Mesothelia cells.

**Differential Expression Analysis per Dataset**

For RNA-seq datasets, raw count matrices and phenotype data were analyzed using DESeq2 (1). Samples were classified as Case (fibrosis stage 3–4) or Control (stage 1–2) based on clinical metadata. For each dataset, we constructed a DESeqDataSet with design ~ condition, removed low-count genes (rowSums > 1), and fitted a negative binomial generalized linear model using DESeq(). We extracted one-sided Wald test p-values for the Case vs Control comparison: Up-regulated genes (Case > Control): results(dd, lfcThreshold = 0, altHypothesis = "greater"); Down-regulated genes (Case < Control): results(dd, lfcThreshold = 0, altHypothesis = "less"). Extracting p-values for both directions was to ensure that the subsequent meta-analysis specifically aggregated evidence for consistent directional changes across independent datasets, preventing the conflation of opposing expression profiles.

For microarray datasets, normalized expression matrices were analyzed using Welch’s two-sample t-test for each gene, which accounts for unequal variances. Similarly, both one-sided p-values were computed for *Case > Control* and *Case < Control* using t.test() with alternative = "greater" or "less".

**Meta-analysis of Bulk RNA-seq and Microarray Datasets**

To identify genes consistently associated with fibrosis across multiple transcriptomic datasets, we performed a multi-step meta-analysis.

**Meta-analysis of gene expression across datasets**

For each gene, one-sided p-values across datasets (RNA-seq only, microarray only, or RNA-seq+microarray) were combined using Edgington’s sum-of-p-values method (2), implemented in the R package metap. This approach sums p-values across studies and computes a combined significance measure without assuming effect-size homogeneity, making it suitable for heterogeneous datasets. The formula is:

$$S=\sum_{i=1}^{k} p_{i},\text{metaP}=\frac{S^{k}}{k!}-\binom{k}{1}\frac{\left( S - 1)^{k} \right.}{k!}+\binom{k}{2}\frac{\left( S - 2)^{k} \right.}{k!}-\ldots$$

where $k$is the number of datasets and $p_{i}$ are the one-sided p-values for gene $g$. This procedure was applied separately for up- and down-regulated directions.

**Multiple Testing Correction**

Meta p-values were adjusted for multiple comparisons using Holm’s step-down procedure (p.adjust(method = "holm")) to control the family-wise error rate. Genes with adjusted meta p-values ≤ 0.05 were considered significant.

**Deconvolution Pipeline**

Let $\tilde{X}_{U*V}$ represent tissue data consisting of $M$genes and $V$ samples. A deconvolution analysis models $\tilde{X}_{U*V}$ as a non-negative product of the following form:

$$\tilde{X}_{U_{0}*V}=\tilde{A}_{U_{0}*K_{0}}*\tilde{B}_{U_{0}*V}+E,\tilde{A}_{U_{0}*K_{0}} \geq0, where \tilde{B}_{K_{0}*V}\geq0. (1)$$

In this context, $\tilde{X}_{U_{0}*V}$ denotes the observed gene expression matrix for $U_{0}$ selected genes (a subset of $U$) across $V$ tissue samples. The columns of $\tilde{X}_{U_{0}*V}$ and the rows of $\tilde{B}_{V_{0}*V}$ represent the expression signatures and the relative proportions of the $K\_0$ cell types, respectively. The parameters $\tilde{A}_{U_{0}*K_{0}}$, $U_{0}$, and $K_{0}$ are unknown variables. Accurately identifying the cell types ($K_{0}$) and selecting relevant cell type marker genes ($U_{0}$) can substantially improve the prediction accuracy of $\tilde{B}_{V_{0}*V}$.

Leveraging cell type specific marker genes identified from scRNA-sequencing data of liver tissue, the process begins by detecting all rank-1 modules within the target dataset using a method of iterative hierarchical clustering combined with bicross-validation (BCV). Subsequently, rank-1 modules that significantly overlap with the core marker genes of a singular cell type are selected, indicating their potential as cell type-specific markers for the dataset in question. Modules exhibiting high collinearity are merged to refine the analysis. As a result, genes within each module are designated as markers for a specific cell type, meeting the criteria for being 'transcriptionally identifiable'. The overall count of these modules provides an estimate of the number of identifiable cell types, denoted as $K_{0}$.

The cell type proportions are then predicted using a constrained non-negative matrix factorization (NMF) approach, which addresses the optimization problem outlined as follows:

$${}_{\tilde{A}_{U_{0}*K_{0}},\tilde{B}_{U_{0}*V}}^{min}{(\left| \left| \tilde{X}_{U_{0}*V}-\tilde{A}_{U_{0}*K_{0}}*\tilde{B}_{U_{0}*V} \right| \right|_{F}^{2}+\lambda*trace(\tilde{A}_{U_{0}*K_{0}}^{T}*(1_{U_{0}}*1_{K_{0}}^{T}-C_{U_{0}*K_{0}})))} (2)$$

In this context, the constraint matrix $C_{U_{0}*K_{0}}$ is defined such that $C_{U_{0}*K_{0}}\left[ i, j \right]=1$ if gene $i$ is a marker for cell type $j$, and 0 elsewhere. Here, $1_{d}$ represents a column vector of ones with length d, λ is a hyperparameter optimized through cross-validation, and other variables adhere to the definitions provided in equation (1). The inclusion of $C_{U_{0}*K_{0}}$ in the regular NMF process ensures that the resulting signature matrix $\tilde{A}_{U_{0}*K_{0}}$ aligns with the constraints posed by $C_{U_{0}*K_{0}}$, specifically that in the $kth$ column of $\tilde{A}_{U_{0}*K_{0}}$, genes identified as markers for cell type $k$ exhibit higher expression levels.

The solution to this optimization (referred to as equation 2) employs an alternating update strategy, where one of $\tilde{A}_{U_{0}*K_{0}}$ or $\tilde{B}_{U_{0}*V}$ is fixed, and the other is updated in each step. The parameter λ is adjustable based on simulations with tissue data of known cell proportions. For this research, λ was fine-tuned and empirically set to 10 for datasets that are either log-normalized microarray data or log-transformed (X + 1) for normalized RNA-seq data.

**Meta-analysis of deconvoluted cell type proportions**

After estimating cell-type proportions for each dataset, we tested whether each cell type differed between Case and Control groups using the Wilcoxon rank-sum test, which is a non-parametric method robust to non-normal distributions and small sample sizes. This provided p-values for each cell type within each dataset. Note here, to account for consistent change of directions across multiple datasets, we performed one-sided tests for both directions, in addition to the two-sided tests. To integrate evidence across multiple datasets, we applied Edgington’s sum-of-p-values method, similar to the meta-analysis applied in the gene expression analysis above. We computed meta p-values for each cell type and adjusted for multiple testing using Holm’s step-down procedure to control the family-wise error rate. Cell types with adjusted meta p-values ≤ 0.05 were considered significantly different between groups.

**STRING analysis of differentially expressed genes:** STRING analysis was applied to explore network connectivity and edge weights. The top 100 significantly upregulated and downregulated DEGs were uploaded, and clustering was conducted using the Markov Cluster Algorithm (MCL) with the inflation parameter set to 2. A clustering map was generated, excluding disconnected nodes from the network.

**Supplementary Figure 1.** **Putative interactions of the top 100 upregulated genes reveal changes in ECM organization, neutrophil chemotaxis, and signaling pathways.** The top 100 upregulated genes were analyzed for protein networks using STRING (<https://string-db.org/>).(3) Clustering of significant upregulated genes shows enrichment of genes associated with ECM organization, VEGF2 signaling, and neutrophil chemotaxis.

**Supplementary Figure 2.** **Putative interactions of the top 100 downregulated genes reveal changes in metabolism and trafficking pathways.** The top 100 downregulated genes were analyzed for protein networks using STRING (<https://string-db.org/>).(3) Clustering of significantly downregulated genes show alterations in transportation of amino acids, bile acids, and steroids, as well as metabolism, mitochondrial function, and amino acid biosynthesis/metabolism.

**Supplemental Figure 3. Identification of Ligand/Receptor interactions enriched in patients with advanced fibrosis.** Four potentially novel ligand-receptor interactions involved in fibrosis are displayed. This crosstalk between ligands (dark blue circles with white lettering) with receptors (light blue circles with black lettering) expressed by specific cell types provides insight into potential fibrogenic cell-cell and ligand-receptor interactions.

**Supplementary Table 1: Top 100 upregulated and top 50 downregulated genes identified in our meta-analysis.**

| **Upregulated Genes** | ***P*-value of Individual Dataset** | | | | | | | | | | **Meta *P*-value** | **Adjusted Meta  *P*-value** |
| --- | --- | --- | --- | --- | --- | --- | --- | --- | --- | --- | --- | --- |
|  | **Gerhard** | **GSE130970** | **GSE135251** | **GSE162694** | **GSE225740** | **GSE185051** | **GSE193066** | **GSE49541** | **GSE89632** | **GSE96971** |  |  |
| CCL19 | 6.24E-10 | 3.95E-05 | 9.40E-17 | 4.33E-05 | 2.35E-04 | 5.55E-03 | 1.03E-05 | 2.65E-05 | 3.24E-03 | 3.34E-03 | 2.53E-26 | 2.99E-22 |
| DPT | 1.92E-13 | 1.23E-03 | 1.84E-23 | 9.29E-05 | 3.22E-04 | 1.68E-02 | 1.18E-04 | 4.83E-15 | 4.76E-03 | 9.44E-03 | 3.94E-22 | 4.66E-18 |
| SOX4 | 1.70E-08 | 1.94E-12 | 2.54E-21 | 4.67E-20 | 4.80E-09 | 3.00E-03 | 1.18E-06 | 2.19E-09 | 2.17E-02 | 1.17E-02 | 1.12E-21 | 1.32E-17 |
| IGFBP7 | 4.36E-17 | 1.23E-15 | 2.89E-09 | 2.22E-15 | 8.20E-07 | 1.04E-02 | 3.09E-05 | 5.44E-12 | 1.04E-02 | 1.60E-02 | 1.28E-21 | 1.51E-17 |
| AQP1 | 1.83E-12 | 5.19E-06 | 7.60E-21 | 2.72E-10 | 2.73E-06 | 3.13E-03 | 8.82E-08 | 1.35E-11 | 2.03E-02 | 2.47E-02 | 1.84E-20 | 2.18E-16 |
| GRAMD1B | 3.73E-02 | 3.78E-05 | 8.61E-16 | 4.18E-05 | 5.87E-04 | 8.89E-03 | 7.56E-05 | 7.13E-03 | 1.38E-02 | 8.03E-03 | 1.74E-18 | 2.07E-14 |
| CAPN2 | 1.74E-06 | 8.57E-06 | 9.93E-25 | 7.05E-12 | 2.64E-06 | 1.13E-02 | 2.09E-08 | 3.15E-06 | 4.66E-03 | 6.34E-02 | 2.73E-18 | 3.23E-14 |
| LOXL4 | 1.43E-12 | 1.03E-07 | 5.14E-44 | 9.15E-23 | 9.17E-05 | 6.09E-02 | 1.86E-07 | 2.19E-07 | 1.85E-02 | 2.89E-03 | 3.98E-18 | 4.71E-14 |
| SOX9 | 6.00E-16 | 1.41E-11 | 1.39E-25 | 2.40E-18 | 8.37E-08 | 1.81E-03 | 1.59E-09 | 1.79E-08 | 2.32E-02 | 5.99E-02 | 5.32E-18 | 6.30E-14 |
| CCL21 | 4.68E-12 | 2.26E-06 | 3.98E-13 | 1.46E-06 | 9.57E-07 | 4.45E-04 | 8.14E-06 | 1.18E-04 | 7.99E-02 | 5.97E-03 | 6.39E-18 | 7.56E-14 |
| IL32 | 2.55E-04 | 1.41E-04 | 1.15E-14 | 2.26E-07 | 1.61E-02 | 6.86E-02 | 2.02E-03 | 5.28E-04 | 2.91E-03 | 1.26E-04 | 1.04E-17 | 1.23E-13 |
| RASL11B | 4.14E-02 | 4.05E-09 | 8.40E-14 | 9.70E-04 | 3.81E-03 | 2.59E-02 | 6.72E-07 | 7.61E-05 | 2.21E-02 | 6.10E-03 | 2.86E-17 | 3.38E-13 |
| CCL20 | 1.00E-02 | 2.12E-05 | 1.06E-21 | 4.81E-19 | 7.13E-03 | 2.40E-02 | 4.77E-05 | 1.69E-08 | 4.72E-03 | 5.54E-02 | 3.16E-17 | 3.74E-13 |
| DCDC2 | 1.45E-20 | 1.99E-10 | 2.53E-25 | 7.10E-13 | 1.08E-08 | 2.94E-04 | 9.35E-08 | 7.24E-12 | 1.45E-03 | 1.10E-01 | 8.20E-17 | 9.70E-13 |
| THBS2 | 4.02E-15 | 7.98E-12 | 5.23E-35 | 1.36E-16 | 6.22E-05 | 6.61E-02 | 1.46E-12 | 6.71E-14 | 5.24E-02 | 1.02E-04 | 1.52E-16 | 1.80E-12 |
| BACE2 | 5.94E-09 | 2.56E-04 | 2.72E-13 | 1.99E-09 | 1.15E-05 | 1.33E-02 | 7.95E-06 | 9.85E-06 | 9.61E-02 | 1.15E-02 | 1.87E-16 | 2.21E-12 |
| FBLN5 | 3.23E-22 | 6.84E-11 | 6.94E-28 | 3.02E-12 | 1.10E-04 | 9.71E-02 | 8.38E-13 | 1.19E-08 | 1.18E-02 | 2.32E-02 | 4.50E-16 | 5.32E-12 |
| ADAMTSL2 | 8.34E-11 | 1.80E-14 | 2.73E-08 | 1.15E-03 | 2.26E-04 | 9.11E-02 | 6.14E-11 | 8.39E-03 | 1.17E-02 | 2.41E-02 | 6.23E-16 | 7.37E-12 |
| CXCL6 | 5.64E-07 | 1.57E-18 | 1.10E-26 | 6.92E-28 | 1.01E-08 | 2.58E-03 | 3.05E-06 | 4.51E-12 | 3.11E-02 | 1.06E-01 | 7.96E-16 | 9.41E-12 |
| SH3YL1 | 1.02E-07 | 2.93E-05 | 7.88E-11 | 2.82E-06 | 7.17E-04 | 4.18E-03 | 3.37E-06 | 8.10E-07 | 5.52E-03 | 1.32E-01 | 9.30E-16 | 1.10E-11 |
| LUM | 1.40E-31 | 1.55E-16 | 2.34E-25 | 1.18E-13 | 2.15E-05 | 2.97E-02 | 1.48E-09 | 1.37E-15 | 4.60E-03 | 1.08E-01 | 9.36E-16 | 1.11E-11 |
| PODN | 1.69E-20 | 9.97E-06 | 3.65E-20 | 9.70E-10 | 8.36E-04 | 1.37E-01 | 3.61E-12 | 3.11E-04 | 8.97E-03 | 1.84E-03 | 1.48E-15 | 1.75E-11 |
| CCDC146 | 9.66E-04 | 8.17E-04 | 8.87E-17 | 5.48E-06 | 2.10E-07 | 1.21E-01 | 2.50E-02 | 2.09E-09 | 5.46E-04 | 2.08E-03 | 1.63E-15 | 1.93E-11 |
| TMED3 | 2.23E-02 | 1.03E-05 | 1.31E-07 | 1.18E-07 | 1.69E-02 | 4.56E-02 | 3.15E-02 | 1.29E-03 | 2.69E-02 | 7.79E-03 | 1.84E-15 | 2.17E-11 |
| CCND2 | 1.07E-11 | 2.45E-03 | 1.89E-06 | 1.61E-04 | 9.30E-04 | 3.24E-02 | 4.16E-04 | 2.92E-06 | 1.16E-01 | 4.10E-03 | 2.43E-15 | 2.87E-11 |
| PRR15L | 3.88E-03 | 6.52E-03 | 6.15E-15 | 1.35E-02 | 1.86E-03 | 7.91E-02 | 4.43E-02 | 4.00E-04 | 1.30E-02 | 3.27E-03 | 4.32E-15 | 5.11E-11 |
| SUSD2 | 5.34E-03 | 5.46E-18 | 2.35E-16 | 1.31E-13 | 3.63E-02 | 1.20E-01 | 1.85E-04 | 4.39E-07 | 2.35E-03 | 8.33E-03 | 6.60E-15 | 7.79E-11 |
| RAB25 | 3.60E-02 | 2.92E-06 | 8.57E-19 | 1.18E-09 | 1.45E-05 | 1.91E-02 | 6.76E-03 | 1.22E-05 | 2.37E-02 | 8.89E-02 | 7.20E-15 | 8.51E-11 |
| C7 | 9.45E-20 | 4.95E-11 | 3.37E-17 | 3.57E-08 | 2.74E-04 | 1.74E-01 | 3.06E-07 | 4.65E-13 | 4.51E-03 | 1.21E-03 | 9.76E-15 | 1.15E-10 |
| CD58 | 6.40E-04 | 3.75E-06 | 2.65E-03 | 8.04E-06 | 2.67E-03 | 5.47E-02 | 3.71E-02 | 1.20E-02 | 3.67E-04 | 7.28E-02 | 1.16E-14 | 1.37E-10 |
| IFI16 | 1.18E-03 | 2.17E-06 | 1.31E-04 | 2.64E-11 | 1.47E-03 | 8.90E-02 | 4.20E-03 | 4.18E-03 | 4.68E-02 | 4.64E-02 | 2.02E-14 | 2.39E-10 |
| ARRDC2 | 5.23E-02 | 7.88E-03 | 5.94E-12 | 6.77E-11 | 1.41E-05 | 4.38E-02 | 2.06E-03 | 2.13E-03 | 8.42E-02 | 2.58E-03 | 2.18E-14 | 2.57E-10 |
| ITGBL1 | 2.60E-27 | 1.75E-08 | 1.49E-44 | 1.68E-11 | 9.14E-06 | 1.83E-01 | 7.55E-14 | 2.03E-16 | 6.37E-03 | 1.30E-02 | 3.26E-14 | 3.85E-10 |
| MFAP4 | 1.48E-19 | 3.90E-09 | 6.40E-17 | 1.35E-07 | 2.85E-03 | 3.43E-02 | 9.93E-05 | 1.07E-01 | 5.78E-02 | 1.85E-03 | 3.44E-14 | 4.06E-10 |
| GSTP1 | 3.45E-05 | 6.42E-08 | 2.87E-06 | 1.16E-06 | 6.18E-04 | 1.39E-01 | 2.11E-03 | 6.50E-03 | 4.55E-02 | 1.07E-02 | 3.50E-14 | 4.13E-10 |
| THY1 | 9.23E-06 | 9.83E-13 | 1.40E-32 | 2.67E-12 | 4.42E-05 | 3.98E-02 | 1.48E-05 | 5.18E-09 | 1.67E-01 | 2.10E-03 | 4.43E-14 | 5.23E-10 |
| COL3A1 | 3.59E-14 | 3.28E-09 | 3.56E-19 | 6.96E-04 | 3.68E-03 | 1.52E-01 | 5.07E-07 | 1.69E-12 | 2.61E-02 | 2.76E-02 | 4.62E-14 | 5.45E-10 |
| GPC3 | 1.40E-07 | 1.32E-07 | 4.90E-14 | 7.53E-08 | 1.54E-04 | 1.96E-01 | 6.61E-03 | 1.82E-03 | 1.16E-03 | 4.57E-03 | 4.64E-14 | 5.48E-10 |
| GPX8 | 7.99E-09 | 1.97E-07 | 2.27E-11 | 8.09E-06 | 1.80E-05 | 1.07E-01 | 5.09E-04 | 6.01E-11 | 6.54E-02 | 3.83E-02 | 4.88E-14 | 5.76E-10 |
| LEF1 | 4.32E-02 | 1.19E-07 | 3.44E-11 | 6.68E-09 | 1.18E-02 | 3.23E-02 | 8.78E-04 | 1.58E-03 | 2.71E-03 | 1.27E-01 | 7.21E-14 | 8.51E-10 |
| KRT7 | 7.79E-02 | 1.76E-08 | 1.28E-24 | 1.26E-14 | 1.08E-06 | 2.16E-04 | 7.18E-05 | 9.68E-06 | 8.73E-02 | 5.51E-02 | 7.53E-14 | 8.89E-10 |
| LAMC3 | 4.06E-09 | 7.81E-15 | 1.09E-24 | 5.80E-11 | 4.00E-04 | 8.95E-02 | 1.40E-15 | 1.15E-01 | 1.52E-03 | 1.90E-02 | 9.47E-14 | 1.12E-09 |
| ENPP2 | 1.78E-03 | 1.68E-02 | 1.55E-03 | 3.05E-06 | 1.45E-04 | 7.18E-02 | 9.45E-03 | 1.83E-05 | 4.48E-08 | 1.25E-01 | 9.74E-14 | 1.15E-09 |
| JAG1 | 8.07E-12 | 3.13E-08 | 1.86E-19 | 2.61E-07 | 5.81E-08 | 1.42E-02 | 6.92E-13 | 9.64E-11 | 2.16E-01 | 2.67E-03 | 1.27E-13 | 1.50E-09 |
| GLS | 8.20E-11 | 3.27E-04 | 1.03E-10 | 1.49E-11 | 5.31E-06 | 1.76E-01 | 1.02E-06 | 6.66E-08 | 1.37E-02 | 4.52E-02 | 1.44E-13 | 1.70E-09 |
| HEYL | 2.95E-04 | 7.93E-12 | 4.84E-07 | 1.01E-06 | 8.77E-03 | 1.00E-01 | 9.92E-04 | 3.17E-03 | 1.13E-01 | 9.05E-03 | 1.46E-13 | 1.72E-09 |
| WNT4 | 3.39E-02 | 8.33E-08 | 3.56E-19 | 2.67E-06 | 1.04E-01 | 6.71E-02 | 2.30E-06 | 2.10E-03 | 1.76E-02 | 1.23E-02 | 1.57E-13 | 1.85E-09 |
| AEBP1 | 9.76E-24 | 1.13E-10 | 1.17E-20 | 5.24E-11 | 5.28E-05 | 2.28E-01 | 1.25E-06 | 4.83E-06 | 1.16E-02 | 2.77E-03 | 1.89E-13 | 2.23E-09 |
| SPINT2 | 1.66E-06 | 8.98E-06 | 7.55E-12 | 4.09E-05 | 9.42E-07 | 1.66E-01 | 1.99E-05 | 7.59E-05 | 7.50E-03 | 6.87E-02 | 1.94E-13 | 2.29E-09 |
| STMN2 | 7.78E-13 | 2.20E-11 | 2.31E-42 | 2.50E-30 | 2.63E-04 | 1.41E-01 | 1.56E-09 | 4.73E-14 | 7.73E-04 | 1.02E-01 | 2.06E-13 | 2.43E-09 |
| LTBP2 | 1.02E-19 | 2.25E-09 | 1.67E-36 | 3.54E-10 | 3.44E-05 | 1.75E-01 | 5.51E-15 | 5.74E-09 | 5.79E-02 | 1.18E-02 | 2.10E-13 | 2.48E-09 |
| LAYN | 1.62E-02 | 2.41E-03 | 5.44E-09 | 8.94E-07 | 1.56E-05 | 1.36E-01 | 8.64E-04 | 3.16E-06 | 6.93E-02 | 2.00E-02 | 2.17E-13 | 2.56E-09 |
| FXYD2 | 1.25E-02 | 1.26E-06 | 4.06E-13 | 1.34E-05 | 6.61E-06 | 1.19E-02 | 8.67E-05 | 7.01E-10 | 9.47E-02 | 1.28E-01 | 2.36E-13 | 2.78E-09 |
| GPRC5B | 5.82E-11 | 1.13E-03 | 1.77E-23 | 1.71E-08 | 1.88E-06 | 3.60E-02 | 7.85E-05 | 1.79E-06 | 2.07E-01 | 3.65E-03 | 2.39E-13 | 2.82E-09 |
| LXN | 1.21E-21 | 3.88E-09 | 4.35E-10 | 1.39E-14 | 2.06E-05 | 1.62E-01 | 9.06E-06 | 3.38E-03 | 3.50E-02 | 5.11E-02 | 2.81E-13 | 3.31E-09 |
| FAP | 2.61E-03 | 3.20E-08 | 4.27E-40 | 2.10E-16 | 2.28E-05 | 2.50E-01 | 9.72E-09 | 6.35E-05 | 4.52E-03 | 3.05E-03 | 3.92E-13 | 4.62E-09 |
| UBD | 6.60E-09 | 1.97E-06 | 1.52E-09 | 3.79E-09 | 1.20E-02 | 2.42E-01 | 5.14E-06 | 3.21E-07 | 4.32E-07 | 6.75E-03 | 3.99E-13 | 4.71E-09 |
| FMOD | 4.71E-07 | 4.74E-10 | 2.43E-10 | 5.54E-09 | 7.10E-03 | 9.55E-02 | 1.22E-05 | 4.41E-06 | 1.19E-01 | 5.24E-02 | 6.53E-13 | 7.69E-09 |
| SERPINE2 | 1.03E-03 | 1.45E-11 | 4.93E-11 | 9.07E-07 | 1.57E-05 | 2.39E-02 | 9.68E-03 | 1.66E-08 | 1.38E-01 | 1.09E-01 | 8.49E-13 | 1.00E-08 |
| TC2N | 6.59E-22 | 7.49E-03 | 1.74E-07 | 1.02E-10 | 2.83E-05 | 2.25E-01 | 1.98E-06 | 1.75E-05 | 1.71E-02 | 3.53E-02 | 9.81E-13 | 1.16E-08 |
| TIMP1 | 2.64E-09 | 4.20E-15 | 6.65E-09 | 1.62E-14 | 1.04E-03 | 2.05E-01 | 1.33E-08 | 2.47E-03 | 7.60E-02 | 1.12E-03 | 9.98E-13 | 1.18E-08 |
| SLCO2A1 | 7.08E-05 | 6.73E-04 | 5.74E-13 | 2.70E-09 | 5.46E-05 | 6.71E-02 | 7.52E-05 | 2.34E-07 | 5.30E-02 | 1.66E-01 | 1.06E-12 | 1.25E-08 |
| MCM6 | 2.02E-03 | 3.80E-07 | 7.17E-03 | 1.07E-07 | 1.87E-03 | 1.35E-01 | 2.14E-05 | 7.36E-04 | 3.44E-03 | 1.38E-01 | 1.10E-12 | 1.29E-08 |
| ASPN | 1.22E-10 | 1.08E-12 | 1.31E-03 | 9.62E-10 | 3.18E-03 | 3.97E-02 | 1.06E-01 | 2.28E-07 | 7.18E-02 | 6.80E-02 | 1.15E-12 | 1.35E-08 |
| PLVAP | 5.36E-08 | 7.92E-06 | 3.74E-14 | 2.91E-04 | 1.70E-07 | 3.00E-02 | 1.00E-03 | 3.44E-02 | 9.60E-02 | 1.28E-01 | 1.16E-12 | 1.37E-08 |
| PDGFA | 6.10E-12 | 1.08E-02 | 2.20E-25 | 2.83E-13 | 1.26E-04 | 9.03E-02 | 2.65E-04 | 1.63E-06 | 1.54E-01 | 3.71E-02 | 1.28E-12 | 1.50E-08 |
| YWHAH | 5.13E-02 | 3.40E-04 | 1.06E-04 | 5.66E-13 | 8.44E-03 | 1.25E-01 | 1.49E-04 | 6.35E-02 | 2.52E-02 | 2.01E-02 | 1.35E-12 | 1.59E-08 |
| TES | 7.25E-04 | 3.13E-03 | 2.78E-03 | 5.52E-11 | 2.58E-05 | 2.70E-02 | 6.64E-02 | 7.21E-10 | 1.41E-01 | 5.49E-02 | 1.42E-12 | 1.67E-08 |
| SAMD11 | 3.23E-02 | 5.71E-11 | 5.23E-12 | 3.28E-07 | 3.25E-02 | 1.91E-01 | 3.82E-11 | 1.38E-02 | 2.39E-02 | 7.15E-03 | 1.68E-12 | 1.98E-08 |
| ANXA2 | 1.81E-02 | 1.00E-09 | 1.92E-20 | 2.79E-17 | 1.25E-03 | 2.28E-01 | 3.00E-04 | 4.41E-04 | 6.75E-03 | 5.16E-02 | 2.02E-12 | 2.38E-08 |
| NDN | 2.30E-06 | 3.92E-05 | 1.63E-02 | 1.91E-04 | 3.34E-03 | 2.50E-02 | 3.97E-02 | 8.12E-02 | 1.19E-01 | 2.20E-02 | 2.03E-12 | 2.38E-08 |
| EFEMP2 | 2.86E-03 | 3.91E-08 | 4.48E-08 | 7.46E-04 | 4.89E-04 | 1.68E-01 | 2.88E-08 | 1.86E-05 | 1.27E-01 | 7.04E-03 | 2.03E-12 | 2.39E-08 |
| HSPB8 | 1.22E-03 | 1.04E-04 | 7.44E-13 | 2.70E-15 | 1.84E-01 | 6.69E-02 | 4.08E-04 | 1.01E-03 | 1.32E-02 | 4.28E-02 | 2.20E-12 | 2.59E-08 |
| BEX2 | 1.76E-08 | 4.47E-03 | 2.49E-13 | 3.99E-07 | 8.74E-05 | 7.07E-02 | 1.33E-01 | 1.10E-05 | 3.81E-02 | 6.46E-02 | 2.34E-12 | 2.76E-08 |
| SFRP5 | 2.67E-05 | 1.02E-04 | 3.55E-12 | 3.44E-06 | 8.48E-05 | 2.81E-04 | 2.19E-03 | 6.46E-06 | 2.46E-02 | 2.88E-01 | 2.69E-12 | 3.17E-08 |
| HMGN4 | 2.48E-05 | 3.65E-03 | 3.59E-07 | 4.76E-03 | 3.37E-03 | 7.91E-02 | 1.68E-01 | 3.13E-04 | 2.11E-03 | 5.44E-02 | 2.73E-12 | 3.21E-08 |
| CCDC3 | 1.82E-12 | 1.57E-13 | 1.77E-06 | 1.08E-10 | 8.93E-03 | 1.43E-01 | 4.08E-03 | 6.14E-05 | 1.07E-01 | 5.51E-02 | 2.94E-12 | 3.46E-08 |
| MFSD6 | 1.49E-08 | 5.76E-03 | 3.22E-06 | 2.29E-05 | 2.35E-05 | 6.40E-02 | 1.24E-03 | 1.53E-04 | 3.30E-02 | 2.14E-01 | 2.95E-12 | 3.47E-08 |
| TUBA1A | 7.24E-04 | 6.23E-07 | 7.21E-09 | 1.02E-07 | 1.46E-03 | 7.00E-02 | 1.24E-02 | 2.41E-07 | 2.33E-01 | 3.67E-03 | 3.22E-12 | 3.79E-08 |
| LMCD1 | 1.27E-02 | 2.93E-06 | 2.34E-14 | 3.55E-14 | 1.99E-02 | 6.59E-02 | 3.50E-09 | 3.60E-05 | 2.16E-01 | 6.89E-03 | 3.23E-12 | 3.80E-08 |
| MGP | 1.09E-08 | 3.41E-02 | 3.73E-09 | 2.34E-07 | 5.96E-03 | 1.75E-01 | 6.24E-05 | 4.82E-08 | 1.01E-01 | 7.52E-03 | 3.46E-12 | 4.07E-08 |
| CLEC14A | 7.37E-04 | 5.69E-05 | 3.35E-06 | 2.31E-08 | 6.18E-05 | 1.29E-02 | 3.99E-02 | 4.76E-03 | 2.29E-01 | 3.83E-02 | 3.76E-12 | 4.42E-08 |
| SERPINH1 | 1.01E-01 | 3.39E-05 | 1.20E-06 | 6.54E-06 | 6.98E-04 | 3.19E-02 | 1.05E-01 | 5.13E-04 | 8.62E-02 | 4.21E-03 | 4.16E-12 | 4.89E-08 |
| UBE2Q2 | 1.17E-11 | 1.02E-07 | 3.54E-03 | 4.00E-10 | 7.05E-06 | 2.33E-01 | 2.91E-02 | 1.45E-05 | 5.15E-02 | 1.32E-02 | 4.23E-12 | 4.97E-08 |
| NTS | 2.59E-07 | 1.72E-05 | 3.40E-04 | 4.32E-05 | 8.86E-02 | 2.66E-02 | 1.19E-03 | 1.07E-06 | 2.09E-01 | 8.93E-03 | 4.87E-12 | 5.73E-08 |
| CD2 | 1.18E-20 | 2.43E-08 | 2.67E-06 | 2.88E-11 | 1.95E-02 | 1.27E-01 | 3.66E-05 | 2.58E-02 | 3.92E-05 | 1.63E-01 | 4.97E-12 | 5.84E-08 |
| FAM102B | 9.42E-03 | 4.79E-05 | 3.57E-02 | 7.52E-13 | 1.01E-03 | 1.74E-01 | 3.04E-06 | 8.06E-07 | 9.73E-02 | 1.94E-02 | 5.19E-12 | 6.10E-08 |
| CD96 | 3.42E-14 | 5.68E-03 | 1.95E-03 | 3.43E-05 | 5.98E-03 | 6.66E-02 | 4.03E-06 | 2.27E-05 | 1.42E-02 | 2.48E-01 | 6.03E-12 | 7.09E-08 |
| VIM | 3.19E-13 | 2.40E-08 | 3.39E-07 | 8.83E-15 | 1.31E-05 | 2.24E-01 | 6.81E-04 | 5.31E-02 | 5.30E-02 | 1.20E-02 | 6.23E-12 | 7.33E-08 |
| RFTN2 | 6.47E-04 | 1.95E-02 | 7.87E-04 | 5.56E-05 | 1.19E-03 | 2.43E-02 | 2.04E-02 | 1.82E-01 | 6.60E-02 | 3.49E-02 | 7.49E-12 | 8.80E-08 |
| ELOVL7 | 9.66E-02 | 1.10E-04 | 3.81E-13 | 1.80E-07 | 4.52E-04 | 3.95E-03 | 5.97E-03 | 2.00E-08 | 8.03E-02 | 1.65E-01 | 8.05E-12 | 9.46E-08 |
| FZD1 | 2.64E-05 | 8.97E-07 | 4.27E-05 | 2.61E-06 | 1.01E-02 | 3.04E-02 | 6.65E-04 | 2.05E-03 | 4.99E-05 | 3.10E-01 | 8.24E-12 | 9.68E-08 |
| CDH11 | 3.07E-02 | 1.16E-04 | 3.24E-10 | 5.56E-04 | 1.74E-03 | 2.36E-01 | 3.56E-11 | 2.49E-12 | 8.89E-02 | 3.66E-03 | 1.05E-11 | 1.23E-07 |
| PEA15 | 6.14E-04 | 1.53E-07 | 9.72E-03 | 1.75E-09 | 2.90E-03 | 8.09E-02 | 3.61E-02 | 1.58E-05 | 9.53E-02 | 1.38E-01 | 1.11E-11 | 1.31E-07 |
| CTSK | 2.37E-05 | 6.02E-06 | 1.44E-13 | 1.48E-07 | 5.03E-04 | 2.89E-01 | 1.19E-02 | 4.83E-06 | 1.71E-02 | 4.69E-02 | 1.18E-11 | 1.39E-07 |
| EMCN | 9.60E-08 | 2.80E-02 | 1.64E-03 | 6.21E-05 | 8.58E-05 | 1.88E-03 | 2.06E-01 | 2.93E-05 | 1.80E-02 | 1.14E-01 | 1.33E-11 | 1.56E-07 |
| COL1A2 | 5.06E-20 | 7.08E-10 | 9.88E-25 | 1.61E-09 | 1.80E-03 | 1.94E-01 | 8.70E-07 | 2.39E-12 | 2.86E-03 | 1.73E-01 | 1.36E-11 | 1.60E-07 |
| INMT | 2.53E-10 | 1.06E-04 | 2.38E-10 | 1.94E-14 | 4.47E-03 | 1.71E-01 | 1.22E-05 | 1.19E-04 | 1.82E-01 | 1.59E-02 | 1.43E-11 | 1.69E-07 |
| FGFR2 | 1.00E-08 | 5.13E-04 | 2.62E-03 | 1.19E-05 | 2.04E-04 | 3.70E-02 | 2.91E-05 | 1.43E-05 | 2.12E-01 | 1.21E-01 | 1.45E-11 | 1.70E-07 |
| ITM2A | 1.69E-09 | 7.81E-02 | 5.08E-02 | 7.29E-02 | 8.27E-02 | 1.55E-02 | 8.64E-03 | 3.57E-05 | 1.74E-02 | 4.87E-02 | 1.51E-11 | 1.78E-07 |
| RAMP2 | 7.87E-04 | 1.35E-03 | 1.20E-02 | 7.20E-02 | 1.68E-04 | 8.10E-02 | 5.31E-02 | 6.32E-04 | 1.49E-01 | 9.89E-03 | 1.72E-11 | 2.02E-07 |
| LBH | 6.24E-03 | 1.49E-06 | 6.90E-08 | 2.64E-10 | 3.17E-07 | 1.24E-01 | 4.10E-05 | 3.07E-06 | 2.48E-01 | 1.72E-03 | 1.74E-11 | 2.04E-07 |
| TMEM51 | 1.85E-02 | 2.36E-05 | 2.59E-05 | 8.11E-08 | 6.28E-04 | 1.54E-01 | 4.62E-03 | 2.19E-02 | 1.61E-01 | 2.05E-02 | 1.76E-11 | 2.07E-07 |
| PLK2 | 2.60E-02 | 1.07E-08 | 5.14E-15 | 1.50E-11 | 1.86E-05 | 4.13E-02 | 9.44E-05 | 3.74E-04 | 3.11E-01 | 4.94E-03 | 1.88E-11 | 2.21E-07 |
| CCND1 | 4.71E-07 | 2.49E-07 | 7.11E-08 | 4.82E-05 | 4.60E-02 | 4.32E-02 | 4.91E-05 | 1.21E-04 | 4.23E-02 | 2.52E-01 | 1.91E-11 | 2.24E-07 |
| CXCL1 | 9.42E-03 | 1.42E-07 | 1.48E-18 | 3.62E-23 | 8.88E-06 | 1.77E-03 | 6.11E-04 | 2.36E-05 | 3.72E-01 | 3.71E-04 | 1.91E-11 | 2.24E-07 |
| RAI2 | 1.95E-02 | 5.41E-05 | 9.45E-03 | 9.16E-07 | 3.35E-04 | 1.61E-01 | 4.62E-05 | 1.96E-02 | 1.61E-01 | 1.54E-02 | 2.02E-11 | 2.37E-07 |
| F3 | 3.10E-07 | 6.36E-07 | 5.58E-18 | 1.05E-15 | 7.65E-06 | 2.46E-01 | 2.99E-09 | 4.73E-07 | 1.24E-01 | 2.13E-02 | 2.32E-11 | 2.72E-07 |
| PLCXD3 | 5.86E-15 | 8.54E-13 | 9.77E-19 | 1.84E-11 | 1.88E-04 | 7.55E-02 | 9.63E-07 | 5.44E-12 | 1.12E-02 | 3.05E-01 | 2.33E-11 | 2.73E-07 |
| LDHB | 5.13E-12 | 1.13E-01 | 4.23E-09 | 3.01E-07 | 1.78E-01 | 4.82E-02 | 1.23E-03 | 4.04E-05 | 5.46E-04 | 4.99E-02 | 2.33E-11 | 2.73E-07 |
| ANGPTL2 | 8.20E-05 | 8.41E-06 | 4.82E-04 | 1.36E-06 | 3.93E-02 | 2.29E-01 | 2.81E-04 | 3.29E-04 | 7.26E-02 | 4.96E-02 | 2.33E-11 | 2.74E-07 |
| SRPX | 2.31E-03 | 1.40E-06 | 5.80E-07 | 9.15E-05 | 9.67E-03 | 1.50E-01 | 3.90E-02 | 1.10E-08 | 1.63E-01 | 3.05E-02 | 2.58E-11 | 3.02E-07 |
| ARHGEF3 | 7.62E-12 | 2.86E-06 | 2.90E-03 | 2.27E-06 | 3.91E-03 | 3.93E-02 | 4.35E-04 | 2.31E-05 | 1.59E-01 | 1.90E-01 | 2.60E-11 | 3.05E-07 |
| EPCAM | 1.06E-15 | 1.06E-07 | 6.86E-25 | 4.60E-13 | 7.05E-05 | 3.13E-03 | 4.20E-06 | 4.39E-11 | 8.44E-03 | 3.95E-01 | 3.45E-11 | 4.04E-07 |
| RNASE1 | 3.58E-08 | 1.20E-07 | 6.94E-09 | 3.18E-11 | 1.64E-02 | 3.87E-01 | 6.18E-04 | 3.13E-03 | 6.28E-04 | 2.14E-03 | 3.65E-11 | 4.28E-07 |
| BLVRA | 6.52E-03 | 2.59E-03 | 2.91E-02 | 2.74E-03 | 1.84E-02 | 1.85E-01 | 5.17E-02 | 7.38E-03 | 7.32E-02 | 4.11E-02 | 4.42E-11 | 5.18E-07 |
| ADRA2A | 9.22E-06 | 5.78E-08 | 2.45E-22 | 1.05E-12 | 2.93E-05 | 6.89E-02 | 1.23E-02 | 7.59E-05 | 6.28E-02 | 2.74E-01 | 4.48E-11 | 5.25E-07 |
| CXCL12 | 3.12E-17 | 1.59E-10 | 8.22E-03 | 9.13E-07 | 6.69E-03 | 9.49E-02 | 1.96E-02 | 9.18E-05 | 7.65E-02 | 2.13E-01 | 4.56E-11 | 5.35E-07 |
| MMP7 | 2.76E-07 | 2.50E-13 | 6.76E-30 | 1.80E-21 | 5.48E-08 | 9.54E-03 | 1.91E-09 | 1.47E-08 | 5.73E-02 | 3.52E-01 | 4.58E-11 | 5.37E-07 |
| PTGDS | 1.60E-15 | 3.99E-07 | 4.06E-19 | 3.93E-02 | 7.18E-05 | 3.29E-01 | 1.27E-04 | 3.35E-05 | 3.48E-02 | 1.67E-02 | 4.68E-11 | 5.49E-07 |
| CLDN11 | 2.31E-04 | 5.91E-02 | 5.79E-23 | 5.80E-14 | 2.56E-03 | 3.27E-01 | 3.81E-04 | 3.67E-11 | 1.64E-03 | 2.94E-02 | 4.72E-11 | 5.53E-07 |
| APOBEC3G | 2.08E-02 | 4.82E-05 | 2.45E-05 | 2.76E-04 | 1.24E-01 | 6.11E-02 | 4.15E-03 | 7.80E-02 | 1.25E-01 | 1.15E-02 | 5.22E-11 | 6.11E-07 |
| TTL | 5.87E-03 | 7.18E-02 | 6.24E-14 | 2.34E-04 | 6.36E-02 | 2.36E-01 | 1.04E-02 | 7.09E-04 | 8.28E-03 | 3.00E-02 | 5.57E-11 | 6.52E-07 |
| CHST9 | 1.03E-06 | 4.65E-04 | 3.56E-11 | 6.11E-12 | 1.18E-03 | 1.78E-01 | 4.42E-03 | 6.72E-12 | 2.75E-04 | 2.50E-01 | 6.58E-11 | 7.71E-07 |
| LRRC32 | 8.88E-05 | 1.33E-13 | 6.23E-07 | 1.58E-13 | 2.20E-04 | 5.17E-02 | 3.92E-08 | 1.11E-01 | 2.63E-01 | 8.75E-03 | 6.69E-11 | 7.84E-07 |
| NCK2 | 4.38E-08 | 6.69E-07 | 2.18E-03 | 3.05E-07 | 2.99E-04 | 6.15E-02 | 9.85E-02 | 1.58E-05 | 2.28E-01 | 4.86E-02 | 7.38E-11 | 8.65E-07 |
| PMP22 | 1.16E-08 | 2.03E-05 | 1.87E-07 | 2.60E-04 | 3.31E-03 | 8.38E-02 | 5.66E-05 | 5.91E-07 | 3.03E-01 | 5.85E-02 | 9.17E-11 | 1.07E-06 |
| TACSTD2 | 1.02E-03 | 1.09E-04 | 2.21E-20 | 2.84E-11 | 5.11E-05 | 1.26E-03 | 9.05E-06 | 1.27E-08 | 1.56E-01 | 2.92E-01 | 9.42E-11 | 1.10E-06 |
| SPATS2 | 9.15E-02 | 2.41E-01 | 2.44E-08 | 4.51E-03 | 4.92E-03 | 1.11E-01 | 5.22E-06 | 1.25E-04 | 1.26E-03 | 1.63E-03 | 1.07E-10 | 1.25E-06 |
| PHLDA3 | 2.06E-01 | 2.97E-09 | 3.81E-11 | 6.32E-08 | 4.42E-03 | 1.72E-01 | 1.27E-02 | 1.10E-03 | 5.20E-02 | 8.57E-03 | 1.10E-10 | 1.29E-06 |
| GBGT1 | 4.80E-02 | 2.36E-04 | 9.40E-06 | 5.38E-03 | 1.65E-03 | 3.44E-01 | 1.02E-02 | 1.70E-02 | 1.31E-02 | 2.13E-02 | 1.18E-10 | 1.38E-06 |
| CARD16 | 3.51E-02 | 8.24E-08 | 1.71E-06 | 3.38E-05 | 5.15E-03 | 3.35E-01 | 5.42E-02 | 1.09E-02 | 1.99E-03 | 1.86E-02 | 1.19E-10 | 1.40E-06 |
| GPR176 | 1.03E-01 | 7.57E-06 | 2.43E-07 | 4.04E-11 | 8.53E-06 | 8.42E-03 | 7.74E-07 | 2.49E-02 | 3.14E-01 | 1.42E-02 | 1.27E-10 | 1.48E-06 |
| CD34 | 2.60E-04 | 1.67E-04 | 8.52E-13 | 4.27E-08 | 4.80E-05 | 1.47E-02 | 1.26E-03 | 3.07E-04 | 3.80E-01 | 7.08E-02 | 1.38E-10 | 1.62E-06 |
| SOD3 | 8.23E-02 | 1.75E-06 | 1.36E-14 | 1.28E-06 | 1.05E-04 | 7.82E-02 | 6.69E-05 | 5.91E-05 | 6.76E-02 | 2.45E-01 | 1.54E-10 | 1.81E-06 |
| MS4A1 | 2.02E-05 | 3.89E-03 | 1.49E-03 | 3.14E-04 | 8.54E-03 | 2.98E-02 | 5.11E-02 | 2.07E-02 | 1.70E-02 | 3.43E-01 | 1.64E-10 | 1.92E-06 |
| TYMS | 3.17E-02 | 1.10E-08 | 4.04E-10 | 1.71E-07 | 7.57E-03 | 3.68E-01 | 4.48E-02 | 1.82E-04 | 2.24E-07 | 2.74E-02 | 1.79E-10 | 2.10E-06 |
| ANXA5 | 5.61E-04 | 2.96E-03 | 3.73E-04 | 1.44E-08 | 1.83E-03 | 1.70E-03 | 6.04E-02 | 2.15E-03 | 2.38E-03 | 4.09E-01 | 1.84E-10 | 2.15E-06 |
| COL4A2 | 2.00E-05 | 5.22E-05 | 2.10E-09 | 1.50E-17 | 1.74E-04 | 3.15E-01 | 3.36E-09 | 1.02E-07 | 1.64E-01 | 3.96E-03 | 1.90E-10 | 2.22E-06 |
| TRAF1 | 6.39E-02 | 4.17E-05 | 2.16E-06 | 1.10E-05 | 1.58E-05 | 1.11E-01 | 1.54E-03 | 7.18E-02 | 2.35E-01 | 3.23E-03 | 2.08E-10 | 2.44E-06 |
| TMCO3 | 2.34E-02 | 2.78E-04 | 6.62E-04 | 7.00E-05 | 2.78E-02 | 2.76E-01 | 1.88E-03 | 4.17E-06 | 2.61E-03 | 1.57E-01 | 2.19E-10 | 2.56E-06 |
| CXCR3 | 7.90E-02 | 9.21E-05 | 6.05E-07 | 7.60E-02 | 6.97E-03 | 1.86E-01 | 1.68E-02 | 1.13E-01 | 1.95E-03 | 1.12E-02 | 2.29E-10 | 2.68E-06 |
| RCAN2 | 2.33E-12 | 6.04E-02 | 4.59E-10 | 3.79E-05 | 1.31E-04 | 2.29E-01 | 3.58E-05 | 1.30E-07 | 7.34E-02 | 1.29E-01 | 2.33E-10 | 2.72E-06 |
| STMN3 | 8.40E-04 | 3.47E-03 | 1.10E-13 | 2.55E-02 | 2.38E-04 | 1.84E-01 | 1.70E-05 | 9.13E-02 | 1.87E-01 | 1.09E-03 | 2.34E-10 | 2.74E-06 |
| CD48 | 1.65E-08 | 1.91E-06 | 5.79E-03 | 3.12E-06 | 6.48E-02 | 2.03E-01 | 3.64E-02 | 1.15E-01 | 3.65E-02 | 3.34E-02 | 2.43E-10 | 2.84E-06 |
| TMSB10 | 4.56E-02 | 3.70E-09 | 1.16E-02 | 3.75E-09 | 1.13E-03 | 3.49E-01 | 7.04E-02 | 2.69E-03 | 1.37E-02 | 4.83E-03 | 2.65E-10 | 3.10E-06 |
| ARL4C | 1.66E-02 | 9.01E-06 | 1.34E-02 | 1.33E-17 | 4.17E-05 | 3.20E-03 | 2.01E-02 | 3.75E-02 | 3.96E-01 | 2.04E-02 | 3.08E-10 | 3.61E-06 |
| PJA1 | 1.35E-01 | 4.59E-05 | 1.93E-02 | 2.20E-05 | 4.45E-03 | 3.03E-02 | 1.66E-02 | 2.71E-01 | 2.24E-02 | 1.11E-02 | 3.29E-10 | 3.85E-06 |
| CCDC112 | 2.17E-01 | 1.54E-01 | 5.18E-04 | 5.51E-03 | 3.25E-03 | 3.17E-02 | 3.78E-02 | 8.81E-05 | 4.57E-02 | 2.04E-02 | 3.68E-10 | 4.30E-06 |
| MAP9 | 5.14E-05 | 2.28E-07 | 4.07E-08 | 1.92E-06 | 1.70E-02 | 2.12E-02 | 3.66E-02 | 1.26E-08 | 2.96E-01 | 1.47E-01 | 3.83E-10 | 4.48E-06 |
| PMEPA1 | 3.23E-12 | 6.78E-06 | 5.39E-17 | 6.64E-05 | 6.01E-07 | 2.64E-03 | 2.25E-03 | 4.46E-07 | 5.06E-01 | 1.44E-02 | 4.46E-10 | 5.21E-06 |
| SPP1 | 3.14E-15 | 3.15E-02 | 1.67E-11 | 4.41E-09 | 4.06E-05 | 2.26E-01 | 1.59E-03 | 7.45E-06 | 5.74E-05 | 2.69E-01 | 4.64E-10 | 5.42E-06 |
| TNFRSF21 | 1.66E-08 | 1.84E-11 | 9.10E-05 | 5.25E-15 | 3.12E-07 | 4.30E-03 | 6.46E-05 | 6.71E-03 | 3.36E-01 | 1.86E-01 | 5.11E-10 | 5.97E-06 |
| ST14 | 1.71E-03 | 5.55E-04 | 4.11E-15 | 5.31E-10 | 1.46E-05 | 5.09E-01 | 3.84E-11 | 1.77E-02 | 5.73E-03 | 2.66E-03 | 5.54E-10 | 6.47E-06 |
| DDB2 | 4.59E-07 | 1.23E-05 | 5.41E-07 | 7.03E-12 | 8.83E-03 | 4.42E-01 | 3.63E-02 | 1.18E-02 | 1.59E-03 | 5.00E-02 | 7.09E-10 | 8.28E-06 |
| DCN | 1.17E-17 | 2.09E-14 | 1.47E-08 | 7.54E-07 | 9.96E-03 | 9.70E-03 | 1.38E-05 | 9.55E-09 | 2.71E-02 | 5.08E-01 | 7.55E-10 | 8.82E-06 |
| CMTM7 | 1.09E-03 | 1.31E-04 | 2.02E-03 | 6.12E-04 | 3.70E-03 | 3.92E-01 | 6.30E-02 | 5.73E-02 | 1.27E-02 | 2.76E-02 | 8.45E-10 | 9.87E-06 |
| BMP5 | 7.48E-06 | 2.73E-04 | 6.33E-02 | 1.43E-04 | 5.23E-02 | 4.66E-02 | 1.10E-05 | 5.45E-02 | 1.38E-03 | 3.44E-01 | 8.80E-10 | 1.03E-05 |
| GPD1L | 2.01E-04 | 1.38E-01 | 2.40E-03 | 7.62E-05 | 4.40E-02 | 1.74E-01 | 8.73E-04 | 4.24E-04 | 1.28E-01 | 7.59E-02 | 8.88E-10 | 1.04E-05 |
| MSRB3 | 3.13E-06 | 6.75E-02 | 2.20E-04 | 7.85E-06 | 2.05E-02 | 9.14E-02 | 6.71E-03 | 8.97E-06 | 2.40E-01 | 1.43E-01 | 9.87E-10 | 1.15E-05 |
| BICC1 | 6.23E-21 | 1.97E-10 | 4.68E-20 | 5.92E-12 | 6.98E-09 | 4.48E-03 | 2.77E-07 | 4.35E-13 | 8.01E-02 | 4.87E-01 | 1.02E-09 | 1.19E-05 |
| JAZF1 | 4.08E-05 | 2.91E-03 | 8.67E-03 | 6.60E-04 | 5.41E-04 | 4.94E-01 | 1.77E-02 | 2.36E-06 | 2.01E-04 | 5.05E-02 | 1.09E-09 | 1.28E-05 |
| TNFSF13B | 6.39E-05 | 1.17E-03 | 1.24E-01 | 6.11E-04 | 2.47E-02 | 3.33E-01 | 6.08E-02 | 2.43E-03 | 2.80E-02 | 1.70E-03 | 1.12E-09 | 1.30E-05 |
| PLP2 | 6.59E-07 | 8.53E-06 | 3.98E-10 | 1.98E-09 | 3.11E-03 | 4.51E-01 | 5.72E-06 | 1.83E-02 | 9.48E-02 | 1.01E-02 | 1.13E-09 | 1.31E-05 |
| PPT1 | 8.52E-04 | 3.22E-05 | 6.30E-02 | 1.25E-04 | 2.77E-02 | 1.00E-01 | 1.77E-01 | 1.24E-01 | 2.50E-03 | 8.28E-02 | 1.14E-09 | 1.33E-05 |
| TMEM200A | 3.59E-02 | 1.62E-08 | 3.26E-14 | 4.20E-16 | 9.15E-04 | 4.07E-01 | 6.86E-05 | 1.14E-10 | 9.31E-02 | 4.43E-02 | 1.21E-09 | 1.41E-05 |
| NALCN | 1.77E-07 | 2.47E-04 | 2.40E-27 | 2.24E-07 | 7.34E-05 | 3.03E-01 | 7.07E-18 | 4.61E-12 | 2.55E-01 | 2.41E-02 | 1.23E-09 | 1.43E-05 |
| TXNDC9 | 1.23E-02 | 1.08E-05 | 1.16E-01 | 4.47E-06 | 3.60E-02 | 9.50E-02 | 1.37E-01 | 1.40E-01 | 1.38E-02 | 3.61E-02 | 1.31E-09 | 1.53E-05 |
| XRCC4 | 1.75E-01 | 2.91E-03 | 8.25E-02 | 1.55E-05 | 1.14E-02 | 6.92E-02 | 9.20E-02 | 4.43E-02 | 4.26E-03 | 1.07E-01 | 1.37E-09 | 1.59E-05 |
| TBX2 | 9.05E-02 | 4.42E-07 | 1.33E-03 | 2.66E-02 | 8.22E-03 | 2.54E-01 | 7.54E-04 | 6.87E-02 | 1.32E-01 | 1.30E-02 | 1.53E-09 | 1.79E-05 |
| ZDHHC13 | 1.33E-01 | 5.88E-05 | 1.74E-08 | 2.10E-03 | 7.74E-04 | 5.12E-03 | 1.83E-04 | 3.04E-07 | 7.12E-02 | 3.83E-01 | 1.56E-09 | 1.82E-05 |
| RFC4 | 4.50E-03 | 2.19E-04 | 1.22E-03 | 2.12E-04 | 9.19E-03 | 1.64E-01 | 1.39E-01 | 2.18E-02 | 2.19E-02 | 2.36E-01 | 1.63E-09 | 1.91E-05 |
| RASSF3 | 1.37E-02 | 3.49E-06 | 2.65E-04 | 5.44E-07 | 8.90E-05 | 4.69E-01 | 4.08E-04 | 2.83E-08 | 1.18E-01 | 4.33E-03 | 1.81E-09 | 2.11E-05 |
| CASP1 | 3.50E-03 | 3.62E-05 | 8.95E-09 | 2.56E-05 | 1.47E-02 | 1.82E-01 | 3.29E-02 | 2.72E-01 | 4.32E-03 | 9.49E-02 | 1.81E-09 | 2.11E-05 |
| ANTXR1 | 9.09E-18 | 3.74E-10 | 2.20E-08 | 1.45E-06 | 8.20E-04 | 4.70E-01 | 1.05E-09 | 1.75E-08 | 1.29E-01 | 6.87E-03 | 1.86E-09 | 2.17E-05 |
| FCGR2A | 1.56E-02 | 5.41E-05 | 5.71E-03 | 7.14E-08 | 2.74E-02 | 3.94E-01 | 1.71E-02 | 4.65E-02 | 1.00E-02 | 9.10E-02 | 1.87E-09 | 2.18E-05 |
| SLIT2 | 1.33E-07 | 1.94E-05 | 4.19E-08 | 1.97E-04 | 6.26E-03 | 4.61E-01 | 1.43E-08 | 8.66E-07 | 1.13E-01 | 2.69E-02 | 1.88E-09 | 2.19E-05 |
| PAPLN | 9.01E-13 | 5.08E-04 | 1.13E-21 | 1.59E-04 | 1.01E-07 | 2.50E-01 | 3.71E-14 | 3.32E-04 | 1.44E-02 | 3.43E-01 | 1.93E-09 | 2.25E-05 |
| COL4A1 | 1.28E-06 | 9.26E-06 | 2.24E-08 | 1.41E-19 | 1.30E-04 | 3.32E-01 | 5.45E-09 | 1.18E-11 | 2.37E-01 | 4.13E-02 | 1.98E-09 | 2.31E-05 |
| HKDC1 | 6.56E-04 | 5.43E-02 | 4.20E-11 | 6.61E-12 | 4.74E-03 | 2.88E-01 | 1.52E-05 | 3.67E-04 | 6.32E-04 | 2.65E-01 | 2.08E-09 | 2.42E-05 |
| SMOC2 | 3.44E-04 | 1.36E-02 | 4.56E-15 | 1.40E-05 | 3.93E-04 | 4.29E-01 | 9.39E-05 | 2.24E-09 | 1.59E-01 | 1.12E-02 | 2.11E-09 | 2.46E-05 |
| TMEM204 | 2.23E-01 | 1.37E-09 | 2.07E-02 | 2.63E-06 | 1.61E-03 | 6.06E-02 | 1.17E-03 | 1.81E-02 | 2.68E-01 | 2.46E-02 | 2.22E-09 | 2.59E-05 |
| ANGPT1 | 6.92E-08 | 3.49E-04 | 7.71E-04 | 3.04E-05 | 1.83E-02 | 1.05E-01 | 1.22E-06 | 1.84E-02 | 9.84E-02 | 3.80E-01 | 2.36E-09 | 2.75E-05 |
| LCK | 6.27E-02 | 6.27E-02 | 1.20E-06 | 1.83E-03 | 6.74E-02 | 6.28E-02 | 5.36E-03 | 3.36E-01 | 7.79E-03 | 1.51E-02 | 2.36E-09 | 2.75E-05 |
| NFASC | 7.57E-06 | 5.76E-09 | 8.49E-22 | 2.80E-06 | 1.70E-04 | 3.96E-01 | 8.29E-13 | 5.92E-02 | 1.48E-01 | 1.91E-02 | 2.40E-09 | 2.80E-05 |
| PFKP | 3.22E-03 | 4.21E-05 | 1.18E-12 | 3.57E-09 | 8.49E-08 | 7.92E-02 | 4.14E-07 | 1.38E-02 | 5.04E-01 | 2.52E-02 | 2.54E-09 | 2.96E-05 |
| SLC35C1 | 1.66E-02 | 8.55E-02 | 4.35E-04 | 2.92E-05 | 1.12E-01 | 2.14E-01 | 8.01E-03 | 5.16E-02 | 9.43E-02 | 4.52E-02 | 2.59E-09 | 3.02E-05 |
| PAQR5 | 9.93E-04 | 1.63E-09 | 7.41E-30 | 4.90E-07 | 6.71E-07 | 2.20E-02 | 4.08E-04 | 1.47E-05 | 5.17E-01 | 9.03E-02 | 2.74E-09 | 3.19E-05 |
| LGALS3 | 9.36E-06 | 5.80E-10 | 8.77E-12 | 1.00E-17 | 1.06E-02 | 5.72E-01 | 1.14E-04 | 4.57E-04 | 4.38E-02 | 1.16E-02 | 3.11E-09 | 3.63E-05 |
| BCL11B | 2.16E-03 | 1.40E-04 | 3.18E-05 | 4.27E-08 | 2.35E-02 | 2.52E-01 | 1.49E-04 | 4.46E-02 | 2.47E-01 | 7.03E-02 | 3.15E-09 | 3.67E-05 |
| CD248 | 1.90E-01 | 1.11E-05 | 3.93E-06 | 1.07E-03 | 6.85E-02 | 5.13E-02 | 7.66E-03 | 3.50E-04 | 3.03E-01 | 2.34E-02 | 3.43E-09 | 3.99E-05 |
| PERP | 2.44E-03 | 1.65E-12 | 9.62E-04 | 7.10E-16 | 8.88E-02 | 1.99E-01 | 1.89E-02 | 1.04E-02 | 2.95E-02 | 2.95E-01 | 3.47E-09 | 4.04E-05 |
| DTNA | 9.83E-12 | 2.14E-03 | 4.53E-24 | 1.02E-12 | 1.97E-04 | 2.85E-01 | 3.83E-12 | 9.48E-09 | 6.08E-04 | 3.59E-01 | 3.53E-09 | 4.12E-05 |
| CD3E | 1.34E-03 | 9.74E-04 | 6.71E-04 | 6.53E-07 | 7.38E-02 | 3.11E-01 | 2.30E-01 | 1.17E-02 | 6.83E-03 | 1.20E-02 | 3.62E-09 | 4.22E-05 |
| TPM1 | 4.88E-12 | 2.24E-02 | 5.50E-18 | 1.05E-12 | 6.51E-02 | 3.60E-01 | 1.93E-04 | 1.95E-07 | 1.23E-01 | 7.89E-02 | 3.68E-09 | 4.29E-05 |
| MAPK10 | 6.66E-04 | 8.35E-04 | 6.87E-11 | 1.07E-02 | 1.21E-02 | 9.41E-02 | 2.70E-06 | 9.12E-02 | 3.95E-01 | 4.62E-02 | 3.78E-09 | 4.40E-05 |
| FAM3B | 5.22E-01 | 6.32E-03 | 2.27E-16 | 1.65E-08 | 9.21E-04 | 4.87E-02 | 4.96E-04 | 2.32E-05 | 2.18E-03 | 7.20E-02 | 3.86E-09 | 4.50E-05 |
| SEL1L3 | 1.64E-09 | 1.37E-07 | 2.48E-06 | 1.89E-06 | 5.98E-06 | 2.66E-01 | 1.72E-10 | 8.13E-03 | 6.11E-03 | 3.73E-01 | 3.90E-09 | 4.54E-05 |
| S100A11 | 4.27E-06 | 9.14E-09 | 8.63E-04 | 5.80E-10 | 6.81E-03 | 3.48E-01 | 8.98E-05 | 4.46E-02 | 2.32E-01 | 2.07E-02 | 3.91E-09 | 4.55E-05 |
| ITM2C | 6.37E-03 | 1.25E-05 | 1.09E-01 | 6.09E-04 | 1.10E-02 | 1.01E-01 | 1.04E-02 | 1.21E-02 | 3.53E-01 | 5.25E-02 | 4.07E-09 | 4.74E-05 |
| COL5A1 | 9.43E-11 | 1.20E-11 | 4.26E-16 | 2.26E-06 | 5.75E-03 | 6.24E-01 | 8.34E-11 | 5.37E-05 | 1.34E-02 | 1.32E-02 | 4.10E-09 | 4.78E-05 |
| NEXN | 2.78E-05 | 2.36E-01 | 9.95E-08 | 1.24E-02 | 2.94E-02 | 1.32E-01 | 1.07E-01 | 1.14E-09 | 7.85E-02 | 6.36E-02 | 4.23E-09 | 4.92E-05 |
| SVEP1 | 1.13E-12 | 9.03E-08 | 3.46E-17 | 2.73E-10 | 1.93E-04 | 5.36E-01 | 6.21E-16 | 3.36E-04 | 9.97E-02 | 2.64E-02 | 4.49E-09 | 5.23E-05 |
| ISLR | 2.82E-06 | 2.28E-08 | 1.24E-09 | 2.30E-05 | 1.86E-03 | 2.40E-01 | 1.57E-06 | 6.98E-02 | 3.17E-01 | 4.39E-02 | 5.21E-09 | 6.06E-05 |
| APOBEC3C | 3.17E-09 | 1.65E-05 | 9.62E-07 | 1.02E-05 | 1.13E-03 | 1.63E-01 | 2.16E-03 | 3.13E-02 | 4.35E-01 | 4.14E-02 | 5.34E-09 | 6.21E-05 |
| CYTH3 | 3.03E-02 | 8.84E-03 | 3.46E-04 | 9.16E-06 | 4.10E-03 | 1.35E-01 | 1.50E-05 | 7.80E-05 | 4.85E-01 | 1.16E-02 | 5.43E-09 | 6.32E-05 |
| S100A6 | 5.25E-03 | 4.24E-09 | 1.12E-09 | 5.96E-13 | 1.04E-04 | 2.38E-01 | 1.90E-04 | 7.93E-09 | 4.27E-01 | 6.77E-03 | 5.55E-09 | 6.46E-05 |
| EFEMP1 | 1.09E-26 | 5.04E-04 | 6.24E-22 | 1.71E-17 | 4.34E-04 | 3.54E-01 | 5.72E-13 | 1.38E-11 | 3.32E-02 | 2.92E-01 | 5.83E-09 | 6.79E-05 |
| CYBRD1 | 1.93E-14 | 1.41E-03 | 1.50E-03 | 4.52E-06 | 2.42E-02 | 4.38E-01 | 8.18E-05 | 4.78E-09 | 1.01E-01 | 1.14E-01 | 5.87E-09 | 6.82E-05 |
| ARPC1B | 7.51E-04 | 1.96E-03 | 7.09E-02 | 6.32E-05 | 4.03E-03 | 3.45E-01 | 6.89E-05 | 2.32E-01 | 1.73E-02 | 1.10E-02 | 6.13E-09 | 7.13E-05 |
| CD3G | 9.89E-12 | 2.15E-02 | 3.32E-04 | 4.29E-02 | 8.36E-02 | 3.48E-01 | 8.24E-03 | 1.64E-01 | 9.27E-04 | 2.04E-02 | 6.78E-09 | 7.89E-05 |
| S100A4 | 3.03E-03 | 1.20E-07 | 2.31E-03 | 4.25E-10 | 6.62E-02 | 3.46E-01 | 1.49E-01 | 1.08E-02 | 9.25E-02 | 2.26E-02 | 6.92E-09 | 8.05E-05 |
| STAT1 | 9.44E-04 | 8.46E-02 | 2.28E-02 | 1.97E-05 | 3.05E-01 | 2.39E-01 | 2.79E-03 | 1.39E-03 | 3.16E-03 | 3.75E-02 | 7.50E-09 | 8.72E-05 |
| GZMK | 1.40E-13 | 5.65E-03 | 1.81E-03 | 8.38E-03 | 5.28E-02 | 6.35E-02 | 9.67E-02 | 1.33E-01 | 1.15E-01 | 2.22E-01 | 7.68E-09 | 8.93E-05 |
| B3GNT9 | 3.98E-03 | 8.47E-08 | 8.76E-05 | 1.71E-02 | 1.95E-01 | 2.34E-01 | 3.96E-03 | 1.56E-01 | 7.81E-02 | 1.26E-02 | 7.94E-09 | 9.23E-05 |
| CLIC6 | 1.61E-12 | 1.45E-10 | 2.19E-30 | 2.85E-11 | 6.77E-10 | 4.87E-04 | 1.06E-06 | 2.36E-05 | 4.07E-02 | 6.62E-01 | 8.17E-09 | 9.50E-05 |
| TSHZ3 | 7.96E-03 | 7.54E-03 | 1.24E-03 | 3.32E-04 | 3.37E-03 | 1.63E-01 | 7.26E-02 | 1.02E-02 | 4.29E-01 | 8.04E-03 | 8.21E-09 | 9.54E-05 |
| CRIP2 | 2.25E-03 | 3.32E-05 | 3.59E-04 | 2.21E-02 | 3.46E-04 | 4.62E-02 | 1.19E-01 | 2.41E-01 | 2.18E-01 | 5.37E-02 | 8.23E-09 | 9.57E-05 |
| SHBG | 4.77E-03 | 3.42E-02 | 1.03E-01 | 2.04E-04 | 3.84E-03 | 3.77E-01 | 7.34E-02 | 6.71E-02 | 1.33E-04 | 4.12E-02 | 8.38E-09 | 9.74E-05 |
| AKR1B1 | 1.09E-03 | 1.64E-05 | 1.80E-02 | 1.32E-08 | 1.09E-01 | 2.22E-01 | 6.81E-05 | 1.95E-01 | 1.25E-01 | 3.88E-02 | 8.84E-09 | 1.03E-04 |
| SCPEP1 | 2.19E-09 | 1.48E-02 | 5.54E-08 | 2.86E-04 | 1.71E-01 | 6.49E-02 | 3.82E-02 | 1.07E-03 | 3.38E-01 | 8.18E-02 | 8.90E-09 | 1.03E-04 |
| FKBP10 | 1.07E-01 | 1.79E-04 | 6.47E-08 | 3.39E-04 | 1.77E-03 | 2.75E-02 | 2.02E-03 | 7.51E-02 | 4.68E-01 | 3.08E-02 | 9.33E-09 | 1.08E-04 |
| GEM | 2.23E-06 | 6.17E-11 | 4.25E-11 | 4.47E-29 | 2.69E-07 | 2.04E-02 | 5.06E-09 | 1.62E-12 | 6.81E-01 | 1.17E-02 | 9.35E-09 | 1.09E-04 |
| MOXD1 | 2.89E-15 | 2.70E-19 | 3.25E-34 | 1.35E-14 | 3.82E-05 | 1.61E-01 | 1.49E-05 | 1.67E-12 | 4.52E-03 | 5.48E-01 | 9.48E-09 | 1.10E-04 |
| GLIS2 | 7.44E-08 | 6.01E-10 | 2.69E-11 | 1.71E-03 | 3.36E-04 | 6.11E-01 | 9.23E-05 | 3.78E-05 | 5.62E-02 | 4.69E-02 | 9.74E-09 | 1.13E-04 |
| COL14A1 | 7.44E-12 | 3.18E-07 | 4.83E-11 | 6.83E-08 | 2.40E-03 | 4.44E-01 | 3.84E-07 | 2.11E-07 | 2.63E-01 | 9.02E-03 | 1.01E-08 | 1.17E-04 |
| FHL2 | 3.68E-02 | 8.72E-03 | 1.54E-09 | 5.78E-15 | 2.85E-03 | 3.27E-01 | 8.03E-04 | 3.47E-04 | 1.43E-01 | 2.04E-01 | 1.09E-08 | 1.26E-04 |
| IL2RG | 2.31E-07 | 5.23E-04 | 7.93E-04 | 7.59E-07 | 1.76E-01 | 1.39E-02 | 1.14E-03 | 1.69E-01 | 3.21E-01 | 4.39E-02 | 1.12E-08 | 1.30E-04 |
| GDF15 | 1.08E-04 | 4.80E-09 | 5.15E-09 | 3.10E-14 | 1.10E-03 | 5.92E-01 | 3.75E-03 | 5.32E-02 | 6.73E-02 | 8.44E-03 | 1.13E-08 | 1.31E-04 |
| MDK | 5.60E-03 | 1.66E-06 | 2.31E-06 | 2.05E-03 | 2.64E-04 | 5.16E-01 | 4.52E-07 | 9.08E-02 | 1.08E-01 | 3.66E-03 | 1.13E-08 | 1.31E-04 |
| VCAN | 4.91E-08 | 1.24E-07 | 2.60E-15 | 1.37E-27 | 4.15E-07 | 4.26E-02 | 1.02E-07 | 1.44E-09 | 2.88E-01 | 3.96E-01 | 1.14E-08 | 1.32E-04 |
| FLI1 | 1.47E-03 | 2.29E-02 | 4.16E-01 | 1.56E-05 | 7.80E-03 | 3.26E-02 | 7.45E-04 | 1.41E-01 | 7.75E-02 | 2.98E-02 | 1.18E-08 | 1.36E-04 |
| EHF | 5.16E-06 | 1.25E-03 | 2.79E-08 | 8.06E-05 | 1.71E-04 | 1.82E-02 | 6.51E-09 | 1.93E-10 | 2.42E-02 | 6.87E-01 | 1.19E-08 | 1.38E-04 |
| DPYSL3 | 1.75E-10 | 1.13E-05 | 5.70E-13 | 2.33E-12 | 8.80E-04 | 3.61E-01 | 1.32E-08 | 4.06E-08 | 3.67E-01 | 1.81E-03 | 1.20E-08 | 1.39E-04 |
| PTPN14 | 1.07E-04 | 2.18E-01 | 2.20E-12 | 8.65E-11 | 2.84E-04 | 2.48E-01 | 1.83E-04 | 1.46E-06 | 2.61E-01 | 3.96E-03 | 1.21E-08 | 1.40E-04 |
| EMB | 1.41E-12 | 5.54E-03 | 3.10E-04 | 3.00E-08 | 2.30E-02 | 2.74E-01 | 1.31E-01 | 1.58E-01 | 1.32E-01 | 1.12E-02 | 1.27E-08 | 1.47E-04 |
| VTCN1 | 5.33E-02 | 4.29E-11 | 3.22E-24 | 5.66E-10 | 1.24E-06 | 4.93E-03 | 1.62E-05 | 2.09E-03 | 3.61E-02 | 6.41E-01 | 1.31E-08 | 1.52E-04 |
| TMEM165 | 1.94E-02 | 1.02E-03 | 7.10E-04 | 1.05E-10 | 7.61E-03 | 2.07E-01 | 1.02E-01 | 1.48E-01 | 4.64E-02 | 2.06E-01 | 1.32E-08 | 1.53E-04 |
| PKDCC | 2.04E-04 | 1.01E-05 | 7.37E-10 | 5.02E-04 | 6.58E-04 | 4.73E-01 | 1.22E-04 | 2.48E-05 | 2.61E-01 | 5.50E-03 | 1.39E-08 | 1.61E-04 |
| SLAMF8 | 2.27E-05 | 4.26E-08 | 3.38E-07 | 2.92E-05 | 1.91E-01 | 3.91E-01 | 6.58E-03 | 2.48E-02 | 9.91E-02 | 2.96E-02 | 1.40E-08 | 1.63E-04 |
| CDR2L | 5.19E-01 | 2.57E-04 | 8.43E-04 | 5.41E-09 | 7.94E-04 | 4.96E-02 | 2.73E-03 | 1.46E-01 | 2.24E-02 | 1.56E-03 | 1.41E-08 | 1.64E-04 |
| PDGFRA | 2.05E-15 | 1.86E-14 | 4.10E-10 | 2.55E-11 | 2.71E-03 | 2.40E-01 | 1.70E-12 | 6.90E-13 | 1.64E-01 | 3.47E-01 | 1.63E-08 | 1.90E-04 |
| IL18 | 3.52E-03 | 3.56E-07 | 9.09E-08 | 8.92E-05 | 8.33E-04 | 4.87E-01 | 8.35E-03 | 1.03E-01 | 8.81E-02 | 6.34E-02 | 1.65E-08 | 1.91E-04 |
| CCDC80 | 7.27E-23 | 6.37E-02 | 4.42E-11 | 5.65E-10 | 2.02E-04 | 3.98E-01 | 8.13E-05 | 3.08E-10 | 2.08E-01 | 8.38E-02 | 1.65E-08 | 1.91E-04 |
| IL27RA | 2.56E-02 | 3.87E-02 | 9.04E-04 | 8.03E-06 | 3.41E-03 | 1.37E-01 | 3.86E-02 | 4.94E-01 | 1.90E-03 | 1.48E-02 | 1.65E-08 | 1.91E-04 |
| CD3D | 1.55E-03 | 1.16E-03 | 3.11E-05 | 7.28E-03 | 8.35E-02 | 1.74E-01 | 6.52E-03 | 2.31E-01 | 2.34E-01 | 1.64E-02 | 1.68E-08 | 1.94E-04 |
| FCER1A | 1.31E-07 | 9.33E-05 | 8.48E-03 | 1.52E-03 | 9.84E-03 | 7.19E-03 | 4.18E-01 | 1.37E-02 | 1.26E-01 | 1.77E-01 | 1.81E-08 | 2.10E-04 |
| CRISPLD2 | 4.40E-03 | 5.29E-06 | 2.61E-11 | 5.69E-11 | 1.28E-02 | 2.47E-01 | 1.39E-06 | 1.23E-05 | 4.31E-01 | 7.06E-02 | 1.90E-08 | 2.20E-04 |
| ABR | 2.26E-02 | 5.57E-02 | 6.46E-06 | 5.03E-04 | 9.36E-03 | 4.09E-01 | 5.82E-07 | 1.30E-01 | 1.14E-01 | 2.80E-02 | 1.99E-08 | 2.30E-04 |
| UNC5B | 2.05E-07 | 1.04E-06 | 3.07E-06 | 3.49E-05 | 3.06E-04 | 2.10E-01 | 1.52E-09 | 1.93E-03 | 6.38E-02 | 4.93E-01 | 1.99E-08 | 2.31E-04 |
| CHST4 | 2.76E-07 | 1.80E-05 | 2.65E-14 | 5.59E-08 | 1.17E-03 | 6.22E-03 | 1.37E-03 | 4.61E-08 | 7.74E-02 | 6.84E-01 | 2.02E-08 | 2.34E-04 |
| RPP25 | 2.31E-03 | 4.05E-08 | 3.42E-02 | 6.62E-04 | 9.54E-04 | 4.31E-01 | 1.52E-02 | 2.44E-01 | 4.11E-02 | 9.11E-04 | 2.03E-08 | 2.35E-04 |
| MSH2 | 1.40E-02 | 3.28E-06 | 3.07E-01 | 2.18E-01 | 2.01E-02 | 2.05E-01 | 2.59E-04 | 2.13E-04 | 3.50E-03 | 5.55E-03 | 2.10E-08 | 2.43E-04 |
| ZBTB38 | 2.44E-06 | 1.89E-02 | 9.74E-02 | 2.07E-07 | 3.85E-02 | 4.47E-01 | 4.98E-05 | 1.73E-06 | 6.06E-03 | 1.69E-01 | 2.22E-08 | 2.57E-04 |
| CH25H | 1.43E-01 | 4.78E-10 | 1.18E-05 | 6.63E-15 | 6.70E-03 | 4.44E-02 | 4.81E-10 | 1.97E-07 | 5.05E-01 | 7.88E-02 | 2.24E-08 | 2.59E-04 |
| PCNA | 2.54E-03 | 1.54E-08 | 1.10E-02 | 3.07E-09 | 1.20E-02 | 3.44E-02 | 4.00E-01 | 2.36E-02 | 2.42E-01 | 5.44E-02 | 2.29E-08 | 2.66E-04 |
| GGT5 | 9.28E-03 | 3.28E-09 | 2.20E-04 | 5.44E-04 | 1.45E-02 | 7.58E-02 | 2.67E-08 | 7.47E-02 | 5.92E-01 | 1.42E-02 | 2.32E-08 | 2.69E-04 |
| ELK3 | 9.48E-08 | 3.60E-05 | 6.12E-01 | 9.77E-04 | 5.95E-04 | 1.87E-03 | 8.73E-03 | 2.64E-06 | 4.56E-02 | 1.12E-01 | 2.35E-08 | 2.72E-04 |
| TSPAN4 | 3.76E-02 | 1.20E-01 | 7.01E-02 | 6.29E-02 | 2.12E-02 | 2.47E-01 | 1.39E-01 | 3.16E-02 | 3.15E-02 | 1.99E-02 | 2.35E-08 | 2.72E-04 |
| PROCR | 1.08E-01 | 3.27E-06 | 1.89E-04 | 1.69E-08 | 7.15E-02 | 1.53E-01 | 3.09E-01 | 7.68E-03 | 9.86E-02 | 3.68E-02 | 2.46E-08 | 2.85E-04 |
| ABCC4 | 4.19E-01 | 1.16E-02 | 3.66E-12 | 1.95E-04 | 1.80E-06 | 2.88E-01 | 2.15E-04 | 8.19E-09 | 6.38E-03 | 6.11E-02 | 2.49E-08 | 2.89E-04 |
| STMN1 | 1.72E-01 | 7.16E-06 | 1.24E-04 | 1.73E-06 | 9.63E-02 | 1.51E-01 | 1.49E-01 | 2.36E-02 | 1.77E-01 | 1.96E-02 | 2.56E-08 | 2.97E-04 |
| COMMD2 | 1.26E-03 | 5.31E-05 | 7.40E-02 | 2.55E-04 | 8.09E-02 | 3.76E-02 | 2.50E-01 | 4.29E-03 | 2.98E-01 | 4.19E-02 | 2.57E-08 | 2.97E-04 |
| ASCC3 | 7.26E-05 | 1.34E-03 | 8.46E-02 | 3.59E-06 | 6.17E-02 | 5.51E-01 | 4.29E-03 | 9.13E-03 | 1.88E-02 | 5.81E-02 | 2.59E-08 | 3.00E-04 |
| KCTD10 | 5.36E-02 | 9.12E-08 | 1.19E-04 | 1.74E-09 | 1.03E-02 | 4.56E-01 | 7.16E-05 | 3.16E-05 | 2.38E-01 | 3.15E-02 | 2.60E-08 | 3.01E-04 |
| PCDH17 | 3.08E-02 | 3.72E-03 | 8.44E-03 | 1.95E-07 | 1.79E-04 | 1.47E-01 | 1.13E-02 | 9.78E-04 | 5.70E-01 | 1.70E-02 | 2.61E-08 | 3.02E-04 |
| CDKN1A | 3.23E-01 | 1.04E-07 | 2.08E-06 | 1.91E-15 | 1.15E-02 | 1.08E-01 | 2.04E-03 | 1.28E-02 | 1.68E-01 | 1.64E-01 | 2.61E-08 | 3.02E-04 |
| ARPC2 | 8.91E-02 | 5.17E-05 | 1.15E-01 | 3.58E-09 | 3.08E-01 | 2.33E-01 | 3.93E-03 | 4.02E-02 | 5.43E-05 | 8.43E-03 | 2.86E-08 | 3.31E-04 |
| CAP2 | 1.19E-06 | 6.22E-06 | 1.31E-12 | 8.85E-07 | 2.39E-01 | 5.22E-01 | 1.54E-05 | 2.18E-04 | 3.00E-04 | 3.67E-02 | 2.88E-08 | 3.34E-04 |
| SCRN1 | 6.98E-06 | 7.88E-06 | 4.48E-07 | 6.23E-06 | 2.87E-05 | 4.37E-02 | 1.06E-09 | 2.31E-01 | 4.50E-01 | 7.52E-02 | 2.96E-08 | 3.42E-04 |
| COL6A3 | 4.05E-06 | 4.67E-03 | 3.99E-07 | 4.04E-10 | 1.39E-02 | 5.86E-01 | 1.99E-10 | 6.33E-09 | 1.70E-01 | 2.55E-02 | 2.96E-08 | 3.43E-04 |
| GPR171 | 4.86E-10 | 3.23E-03 | 1.43E-01 | 4.05E-03 | 5.24E-02 | 2.87E-02 | 1.38E-03 | 2.57E-01 | 1.54E-01 | 1.61E-01 | 3.13E-08 | 3.62E-04 |
| PDGFRB | 1.65E-11 | 6.51E-05 | 4.74E-05 | 1.70E-06 | 5.57E-03 | 2.99E-01 | 8.38E-06 | 1.32E-01 | 3.71E-02 | 3.34E-01 | 3.28E-08 | 3.79E-04 |
| GSN | 3.49E-10 | 3.69E-05 | 2.11E-26 | 9.89E-20 | 1.37E-03 | 1.96E-01 | 2.34E-07 | 1.15E-05 | 6.01E-01 | 1.05E-02 | 3.29E-08 | 3.81E-04 |
| ANXA1 | 7.52E-06 | 7.17E-06 | 1.19E-04 | 1.39E-13 | 8.38E-03 | 4.59E-02 | 1.39E-03 | 1.75E-03 | 7.41E-01 | 1.05E-02 | 3.32E-08 | 3.84E-04 |
| CD40LG | 2.83E-04 | 1.39E-05 | 4.83E-05 | 2.08E-03 | 6.99E-02 | 3.50E-01 | 1.27E-05 | 2.02E-01 | 7.64E-02 | 1.09E-01 | 3.37E-08 | 3.90E-04 |
| E2F3 | 5.97E-02 | 1.61E-06 | 1.76E-08 | 1.01E-14 | 1.20E-03 | 1.79E-01 | 3.57E-01 | 5.35E-05 | 2.07E-01 | 6.67E-03 | 3.39E-08 | 3.92E-04 |
| GLT8D2 | 1.97E-01 | 6.81E-04 | 3.86E-04 | 8.32E-04 | 8.00E-03 | 1.52E-03 | 3.61E-02 | 2.47E-08 | 3.77E-02 | 5.31E-01 | 3.52E-08 | 4.07E-04 |
| PTGFRN | 5.70E-05 | 4.05E-05 | 4.76E-06 | 5.07E-08 | 9.90E-04 | 5.67E-01 | 1.58E-03 | 3.82E-03 | 1.02E-01 | 1.40E-01 | 3.55E-08 | 4.10E-04 |
| DBNDD1 | 1.34E-01 | 1.33E-03 | 1.68E-08 | 6.30E-07 | 1.11E-03 | 3.14E-01 | 8.97E-03 | 3.37E-01 | 1.56E-02 | 3.72E-03 | 3.60E-08 | 4.16E-04 |
| PNMA1 | 1.94E-08 | 7.05E-10 | 1.64E-05 | 2.02E-08 | 3.97E-06 | 4.63E-01 | 4.60E-04 | 5.61E-07 | 3.23E-01 | 3.00E-02 | 3.65E-08 | 4.22E-04 |
| NUSAP1 | 2.94E-01 | 2.11E-01 | 1.29E-05 | 6.29E-02 | 1.25E-01 | 7.08E-02 | 2.72E-02 | 4.02E-05 | 1.10E-04 | 2.65E-02 | 3.70E-08 | 4.28E-04 |
| CXCL10 | 8.65E-03 | 9.46E-04 | 6.21E-09 | 8.61E-02 | 2.90E-01 | 4.30E-01 | 3.65E-04 | 7.10E-04 | 1.73E-04 | 3.93E-03 | 3.81E-08 | 4.40E-04 |
| BBS12 | 2.43E-02 | 1.71E-04 | 3.41E-03 | 1.94E-03 | 4.95E-02 | 3.43E-01 | 3.84E-02 | 1.52E-01 | 1.67E-01 | 4.28E-02 | 3.98E-08 | 4.60E-04 |
| COL1A1 | 4.60E-19 | 3.12E-09 | 1.00E-12 | 9.79E-08 | 2.89E-02 | 6.83E-01 | 2.52E-15 | 9.57E-10 | 9.04E-02 | 2.34E-02 | 4.06E-08 | 4.69E-04 |
| PCDH18 | 2.37E-05 | 9.29E-04 | 2.42E-01 | 3.06E-03 | 2.00E-02 | 1.38E-01 | 3.66E-05 | 9.10E-06 | 1.60E-01 | 2.62E-01 | 4.10E-08 | 4.74E-04 |
| SLC28A3 | 5.34E-01 | 1.85E-03 | 6.20E-08 | 1.18E-02 | 1.87E-03 | 7.12E-02 | 3.83E-04 | 4.07E-02 | 1.19E-01 | 5.48E-02 | 4.55E-08 | 5.25E-04 |
| CD8A | 2.09E-04 | 2.60E-04 | 1.03E-02 | 1.45E-05 | 1.01E-01 | 2.36E-01 | 5.72E-04 | 3.71E-01 | 2.16E-03 | 1.14E-01 | 4.55E-08 | 5.26E-04 |
| INTS9 | 9.84E-02 | 8.81E-03 | 2.39E-04 | 1.65E-02 | 3.47E-01 | 1.20E-01 | 7.01E-06 | 6.96E-02 | 1.78E-01 | 2.44E-03 | 4.85E-08 | 5.60E-04 |
| FSTL3 | 3.86E-03 | 7.03E-16 | 5.14E-16 | 9.66E-07 | 1.87E-03 | 7.88E-02 | 4.94E-08 | 3.23E-01 | 4.36E-01 | 2.95E-04 | 5.04E-08 | 5.82E-04 |
| DACT1 | 9.54E-05 | 8.32E-07 | 8.44E-06 | 4.30E-08 | 1.21E-04 | 2.02E-01 | 7.73E-08 | 1.82E-05 | 1.82E-01 | 4.60E-01 | 5.05E-08 | 5.84E-04 |
| NPNT | 3.23E-10 | 1.11E-01 | 1.10E-10 | 4.82E-03 | 6.13E-05 | 2.72E-01 | 7.11E-06 | 9.78E-05 | 2.14E-01 | 2.42E-01 | 5.10E-08 | 5.89E-04 |
| ARL15 | 6.81E-06 | 2.70E-04 | 1.64E-02 | 6.49E-06 | 5.43E-04 | 2.74E-01 | 8.88E-03 | 1.20E-09 | 4.65E-01 | 7.99E-02 | 5.15E-08 | 5.94E-04 |
| PRTFDC1 | 1.35E-01 | 3.07E-05 | 2.84E-04 | 1.15E-04 | 7.99E-03 | 7.31E-02 | 3.09E-03 | 1.34E-03 | 1.25E-01 | 5.01E-01 | 5.19E-08 | 5.99E-04 |
| WIPF3 | 2.42E-02 | 1.43E-01 | 3.05E-10 | 2.36E-05 | 1.27E-01 | 4.31E-01 | 1.51E-04 | 4.01E-02 | 4.55E-02 | 3.72E-02 | 5.36E-08 | 6.19E-04 |
| ADAMTS2 | 7.54E-12 | 2.73E-06 | 2.59E-12 | 6.86E-05 | 3.71E-03 | 4.45E-01 | 4.67E-08 | 2.21E-04 | 4.02E-01 | 1.40E-03 | 5.55E-08 | 6.40E-04 |
| CYP3A7 | 8.84E-03 | 2.06E-03 | 4.79E-12 | 6.88E-07 | 6.56E-03 | 7.87E-02 | 2.11E-06 | 6.30E-01 | 2.59E-04 | 1.26E-01 | 5.60E-08 | 6.46E-04 |
| NEURL1B | 7.98E-08 | 2.92E-13 | 1.64E-07 | 3.47E-02 | 6.50E-03 | 7.56E-01 | 2.11E-02 | 4.09E-06 | 2.52E-03 | 3.59E-02 | 5.86E-08 | 6.76E-04 |
| FBN1 | 1.04E-07 | 6.40E-03 | 1.26E-11 | 2.25E-07 | 1.39E-04 | 5.71E-01 | 8.96E-06 | 2.34E-12 | 1.17E-01 | 1.65E-01 | 6.05E-08 | 6.98E-04 |
| HSPA4L | 8.84E-04 | 1.76E-03 | 3.34E-05 | 1.73E-14 | 1.29E-02 | 2.67E-02 | 3.68E-01 | 5.40E-04 | 1.24E-03 | 4.49E-01 | 6.11E-08 | 7.05E-04 |
| MXRA8 | 6.08E-04 | 6.77E-07 | 1.63E-10 | 4.83E-03 | 8.99E-04 | 6.35E-01 | 3.82E-05 | 1.78E-02 | 6.30E-02 | 1.39E-01 | 6.18E-08 | 7.13E-04 |
| F13A1 | 5.27E-07 | 1.52E-06 | 2.00E-07 | 1.35E-11 | 2.09E-03 | 7.88E-02 | 1.93E-05 | 2.67E-02 | 6.69E-02 | 6.87E-01 | 6.18E-08 | 7.13E-04 |
| SASH3 | 1.01E-01 | 1.98E-04 | 9.32E-02 | 4.63E-03 | 1.89E-01 | 1.18E-01 | 3.92E-03 | 1.88E-01 | 9.87E-02 | 6.44E-02 | 6.19E-08 | 7.14E-04 |
| PLA2G2A | 1.62E-03 | 8.69E-03 | 5.04E-02 | 1.65E-04 | 4.25E-02 | 2.69E-01 | 7.34E-03 | 1.82E-01 | 4.19E-02 | 2.58E-01 | 6.22E-08 | 7.18E-04 |
| INPP5D | 1.17E-02 | 1.09E-03 | 2.14E-01 | 1.82E-06 | 2.09E-02 | 2.09E-01 | 2.02E-03 | 1.83E-01 | 1.87E-01 | 3.33E-02 | 6.23E-08 | 7.19E-04 |
| LAX1 | 1.69E-02 | 1.51E-01 | 2.19E-03 | 5.92E-04 | 9.09E-02 | 2.56E-01 | 1.45E-03 | 2.19E-01 | 7.53E-02 | 4.99E-02 | 6.37E-08 | 7.34E-04 |
| SPATA18 | 1.08E-04 | 1.71E-02 | 1.70E-07 | 5.42E-05 | 1.15E-02 | 1.44E-01 | 2.52E-03 | 2.12E-02 | 2.35E-06 | 6.70E-01 | 6.57E-08 | 7.57E-04 |
| ANKRD29 | 2.34E-08 | 3.15E-02 | 4.42E-29 | 8.13E-07 | 3.20E-04 | 5.12E-01 | 3.75E-03 | 1.06E-03 | 1.07E-04 | 3.18E-01 | 6.61E-08 | 7.62E-04 |
| LRRC1 | 5.58E-01 | 3.91E-03 | 8.84E-21 | 5.81E-11 | 6.94E-06 | 7.37E-02 | 8.52E-06 | 3.94E-06 | 1.15E-01 | 1.20E-01 | 6.89E-08 | 7.95E-04 |
| PEAR1 | 1.19E-01 | 3.23E-02 | 3.20E-02 | 1.95E-02 | 2.06E-03 | 4.86E-01 | 2.14E-03 | 3.90E-02 | 1.28E-01 | 1.17E-02 | 6.97E-08 | 8.04E-04 |
| ANXA4 | 3.15E-07 | 6.84E-10 | 6.35E-07 | 2.96E-03 | 3.67E-05 | 5.13E-04 | 7.14E-02 | 1.96E-06 | 9.53E-03 | 7.88E-01 | 7.08E-08 | 8.16E-04 |
| FGF23 | 2.88E-01 | 7.03E-03 | 6.42E-02 | 2.53E-02 | 1.59E-02 | 1.16E-01 | 2.86E-03 | 1.66E-01 | 9.58E-02 | 9.48E-02 | 7.27E-08 | 8.38E-04 |
| ID4 | 7.26E-07 | 6.88E-05 | 7.03E-09 | 2.41E-18 | 6.78E-06 | 4.18E-01 | 1.74E-03 | 2.50E-09 | 4.15E-01 | 5.02E-02 | 8.09E-08 | 9.33E-04 |
| AIF1L | 2.22E-02 | 1.36E-01 | 6.93E-11 | 1.15E-02 | 1.38E-04 | 2.98E-01 | 3.66E-02 | 6.44E-03 | 2.53E-01 | 1.22E-01 | 8.20E-08 | 9.45E-04 |
| CFTR | 7.01E-14 | 9.60E-02 | 3.62E-18 | 6.54E-01 | 2.16E-06 | 8.85E-03 | 4.25E-10 | 9.35E-07 | 7.35E-02 | 5.66E-02 | 8.46E-08 | 9.75E-04 |
| KCTD12 | 1.73E-10 | 1.97E-08 | 3.11E-02 | 1.87E-08 | 5.60E-07 | 5.56E-01 | 2.05E-07 | 4.16E-07 | 4.34E-02 | 2.58E-01 | 8.49E-08 | 9.78E-04 |
| FNDC1 | 5.36E-02 | 2.67E-16 | 2.39E-08 | 2.14E-11 | 1.17E-05 | 3.28E-01 | 2.54E-04 | 9.18E-08 | 6.71E-02 | 4.42E-01 | 8.69E-08 | 1.00E-03 |
| GRK5 | 9.29E-03 | 7.17E-05 | 1.86E-02 | 3.51E-06 | 2.99E-02 | 2.97E-01 | 2.98E-03 | 2.44E-05 | 4.91E-01 | 4.31E-02 | 8.79E-08 | 1.01E-03 |
| GPX2 | 2.93E-04 | 2.83E-05 | 1.39E-05 | 8.82E-06 | 1.93E-01 | 4.53E-01 | 1.85E-05 | 2.20E-01 | 5.17E-03 | 2.33E-02 | 9.05E-08 | 1.04E-03 |
| EPDR1 | 1.21E-02 | 1.23E-01 | 1.93E-04 | 2.69E-11 | 7.58E-02 | 2.23E-02 | 1.48E-01 | 8.42E-02 | 1.60E-01 | 2.70E-01 | 9.20E-08 | 1.06E-03 |
| KIAA1522 | 7.75E-04 | 1.51E-06 | 5.51E-07 | 1.31E-04 | 2.49E-05 | 8.03E-01 | 7.39E-03 | 2.32E-02 | 3.18E-02 | 3.18E-02 | 9.41E-08 | 1.08E-03 |
| GZMA | 6.13E-13 | 6.66E-05 | 7.21E-03 | 3.00E-03 | 2.88E-01 | 8.47E-02 | 1.10E-01 | 1.59E-01 | 9.33E-05 | 2.47E-01 | 9.51E-08 | 1.10E-03 |
| CCND3 | 3.18E-02 | 3.64E-06 | 3.08E-01 | 2.46E-04 | 4.76E-02 | 2.27E-01 | 8.85E-02 | 1.31E-02 | 1.65E-01 | 1.80E-02 | 9.58E-08 | 1.10E-03 |
| FPR3 | 6.11E-04 | 6.18E-04 | 3.82E-01 | 3.21E-09 | 1.11E-01 | 3.04E-01 | 6.61E-03 | 8.32E-02 | 4.68E-03 | 8.74E-03 | 9.81E-08 | 1.13E-03 |
| TRAF5 | 5.22E-07 | 2.15E-02 | 2.85E-06 | 5.83E-04 | 2.17E-03 | 2.27E-01 | 8.26E-08 | 3.96E-01 | 2.53E-01 | 3.02E-03 | 9.90E-08 | 1.14E-03 |
| KPNA2 | 2.54E-01 | 1.50E-03 | 5.34E-07 | 1.43E-09 | 9.71E-02 | 6.71E-02 | 1.01E-01 | 5.40E-04 | 3.75E-01 | 1.01E-02 | 1.03E-07 | 1.19E-03 |
| BAX | 8.98E-03 | 5.76E-10 | 1.81E-09 | 8.87E-05 | 2.63E-04 | 5.74E-01 | 1.01E-03 | 2.51E-03 | 2.03E-01 | 1.21E-01 | 1.08E-07 | 1.25E-03 |
| CLDN10 | 1.26E-06 | 8.75E-07 | 1.77E-14 | 5.53E-05 | 3.19E-04 | 5.09E-03 | 1.83E-04 | 7.20E-08 | 3.15E-01 | 5.91E-01 | 1.10E-07 | 1.26E-03 |
| TM6SF1 | 3.71E-02 | 4.12E-02 | 6.99E-03 | 1.28E-05 | 1.28E-01 | 2.38E-01 | 1.50E-01 | 1.37E-01 | 7.26E-03 | 1.69E-01 | 1.13E-07 | 1.30E-03 |
| TUSC3 | 5.69E-03 | 5.33E-02 | 2.32E-02 | 2.44E-07 | 2.46E-04 | 1.02E-01 | 2.16E-01 | 2.11E-01 | 1.38E-01 | 1.68E-01 | 1.18E-07 | 1.35E-03 |
| ACTR1A | 1.24E-02 | 1.94E-01 | 3.48E-04 | 4.47E-04 | 7.04E-02 | 1.04E-01 | 7.86E-02 | 3.48E-01 | 1.39E-02 | 9.90E-02 | 1.21E-07 | 1.39E-03 |
| ARL6IP5 | 7.66E-02 | 5.71E-05 | 7.45E-02 | 7.93E-04 | 4.65E-01 | 5.59E-02 | 6.64E-02 | 2.72E-04 | 2.72E-02 | 1.55E-01 | 1.21E-07 | 1.40E-03 |
| DKK3 | 1.05E-08 | 4.66E-10 | 8.30E-24 | 5.06E-09 | 3.64E-04 | 1.29E-01 | 1.83E-08 | 1.02E-18 | 7.74E-01 | 2.17E-02 | 1.27E-07 | 1.46E-03 |
| OLFM1 | 1.19E-01 | 2.27E-02 | 1.92E-01 | 5.03E-04 | 1.54E-03 | 1.38E-01 | 1.57E-01 | 9.89E-03 | 1.16E-01 | 1.71E-01 | 1.31E-07 | 1.50E-03 |
| SPOCK2 | 1.17E-03 | 1.40E-01 | 7.99E-03 | 5.73E-04 | 5.04E-02 | 4.69E-01 | 2.25E-02 | 1.85E-01 | 4.19E-02 | 1.25E-02 | 1.34E-07 | 1.55E-03 |
| PLAU | 2.18E-01 | 4.72E-07 | 1.77E-13 | 1.31E-07 | 2.76E-02 | 4.45E-02 | 9.67E-04 | 5.72E-01 | 6.50E-02 | 3.73E-03 | 1.35E-07 | 1.56E-03 |
| TMPRSS3 | 5.78E-01 | 1.76E-03 | 4.48E-16 | 1.50E-08 | 4.58E-05 | 2.97E-01 | 2.78E-06 | 2.92E-04 | 5.05E-02 | 3.62E-03 | 1.36E-07 | 1.57E-03 |
| EIF4G3 | 3.40E-04 | 8.26E-03 | 3.47E-02 | 8.05E-06 | 3.94E-02 | 5.95E-01 | 1.07E-02 | 2.97E-02 | 8.87E-02 | 1.26E-01 | 1.37E-07 | 1.58E-03 |
| TMEM185B | 5.67E-04 | 5.88E-05 | 1.21E-02 | 2.32E-08 | 8.90E-04 | 8.53E-02 | 2.69E-03 | 3.47E-02 | 5.81E-01 | 2.19E-01 | 1.42E-07 | 1.64E-03 |
| PRAMEF10 | 1.91E-04 | 1.60E-01 | 2.54E-04 | 4.94E-14 | 1.13E-01 | 1.87E-01 | 2.10E-02 | 4.21E-02 | 2.78E-02 | 3.86E-01 | 1.43E-07 | 1.65E-03 |
| TNFAIP2 | 4.31E-09 | 1.56E-01 | 1.28E-04 | 1.85E-03 | 2.30E-04 | 2.51E-02 | 2.51E-03 | 1.59E-01 | 5.76E-01 | 1.70E-02 | 1.44E-07 | 1.66E-03 |
| TMEM156 | 7.26E-02 | 5.31E-02 | 1.58E-02 | 1.36E-04 | 8.67E-07 | 3.85E-02 | 2.64E-03 | 6.40E-01 | 3.95E-03 | 1.10E-01 | 1.44E-07 | 1.66E-03 |
| MYL12A | 4.96E-02 | 5.23E-02 | 1.59E-04 | 8.32E-18 | 8.50E-02 | 1.91E-01 | 4.12E-01 | 3.02E-03 | 1.26E-01 | 2.04E-02 | 1.47E-07 | 1.69E-03 |
| RARRES1 | 2.94E-06 | 4.74E-03 | 8.31E-03 | 3.54E-03 | 6.38E-02 | 2.62E-01 | 1.89E-01 | 9.75E-04 | 1.64E-01 | 2.44E-01 | 1.47E-07 | 1.70E-03 |
| BGN | 2.40E-06 | 8.08E-10 | 1.12E-01 | 1.77E-06 | 2.56E-01 | 4.72E-02 | 1.99E-02 | 4.85E-02 | 2.24E-01 | 2.34E-01 | 1.50E-07 | 1.73E-03 |
| SLC22A15 | 1.54E-04 | 3.64E-06 | 1.01E-18 | 2.49E-14 | 3.44E-04 | 1.18E-02 | 1.33E-05 | 3.27E-02 | 4.23E-02 | 8.54E-01 | 1.50E-07 | 1.73E-03 |
| SMYD2 | 1.35E-02 | 7.24E-02 | 3.08E-07 | 1.89E-02 | 6.94E-02 | 4.61E-01 | 6.21E-02 | 2.02E-02 | 6.08E-02 | 1.63E-01 | 1.51E-07 | 1.74E-03 |
| ITGAV | 1.95E-05 | 4.19E-08 | 3.02E-05 | 8.07E-14 | 1.58E-04 | 5.13E-01 | 5.69E-05 | 2.58E-09 | 5.49E-02 | 3.74E-01 | 1.52E-07 | 1.74E-03 |
| LAMB1 | 2.53E-06 | 1.64E-01 | 4.63E-09 | 6.16E-08 | 1.58E-03 | 6.58E-01 | 1.62E-06 | 1.04E-07 | 7.63E-02 | 4.30E-02 | 1.52E-07 | 1.75E-03 |
| S100A14 | 6.73E-02 | 1.27E-02 | 7.78E-09 | 5.94E-07 | 3.93E-03 | 6.25E-01 | 6.89E-05 | 7.86E-03 | 2.16E-01 | 1.04E-02 | 1.53E-07 | 1.75E-03 |
| ID3 | 1.49E-02 | 8.94E-09 | 9.74E-02 | 1.41E-04 | 6.79E-04 | 1.84E-01 | 7.32E-02 | 4.62E-01 | 7.85E-02 | 3.21E-02 | 1.54E-07 | 1.77E-03 |
| PYGB | 1.09E-01 | 2.16E-02 | 5.46E-04 | 4.27E-04 | 5.16E-03 | 2.11E-01 | 2.09E-04 | 5.39E-01 | 4.35E-02 | 1.83E-02 | 1.62E-07 | 1.86E-03 |
| STK39 | 7.77E-05 | 2.77E-03 | 3.97E-07 | 3.51E-05 | 9.13E-03 | 6.02E-03 | 2.73E-05 | 3.36E-07 | 2.75E-02 | 9.04E-01 | 1.64E-07 | 1.89E-03 |
| DERL3 | 4.06E-02 | 4.07E-05 | 4.65E-05 | 3.26E-02 | 5.17E-03 | 3.84E-01 | 1.83E-01 | 2.64E-01 | 2.25E-02 | 2.00E-02 | 1.68E-07 | 1.93E-03 |
| NR2C2AP | 8.07E-02 | 1.67E-04 | 4.52E-02 | 1.77E-03 | 2.66E-01 | 3.86E-01 | 3.42E-02 | 6.76E-03 | 1.21E-01 | 1.07E-02 | 1.69E-07 | 1.94E-03 |
| EPHA3 | 2.31E-21 | 2.94E-11 | 5.36E-15 | 3.71E-11 | 1.07E-05 | 7.63E-02 | 2.81E-10 | 1.41E-13 | 1.53E-02 | 8.67E-01 | 1.80E-07 | 2.06E-03 |
| HSPA2 | 1.79E-04 | 4.10E-02 | 1.02E-09 | 2.70E-20 | 8.54E-03 | 1.37E-01 | 5.55E-03 | 2.70E-09 | 7.10E-01 | 5.59E-02 | 1.81E-07 | 2.08E-03 |
| SULF1 | 8.28E-05 | 1.02E-01 | 1.37E-04 | 3.25E-02 | 2.50E-04 | 4.12E-01 | 2.72E-05 | 1.17E-04 | 3.24E-01 | 9.00E-02 | 1.85E-07 | 2.13E-03 |
| TAGLN2 | 8.60E-02 | 9.08E-03 | 4.71E-04 | 1.23E-12 | 1.29E-01 | 3.77E-01 | 2.03E-02 | 1.18E-01 | 2.15E-01 | 5.89E-03 | 1.85E-07 | 2.13E-03 |
| CACNA1C | 3.63E-01 | 3.83E-03 | 8.40E-15 | 1.32E-04 | 9.10E-04 | 2.09E-01 | 7.02E-12 | 3.79E-02 | 3.24E-01 | 2.44E-02 | 1.89E-07 | 2.17E-03 |
| TLR7 | 5.32E-05 | 2.45E-04 | 3.65E-07 | 4.86E-01 | 4.73E-02 | 3.88E-01 | 9.29E-03 | 7.03E-03 | 2.60E-02 | 5.42E-04 | 1.91E-07 | 2.20E-03 |
| TNFRSF12A | 5.06E-02 | 1.03E-07 | 9.22E-26 | 1.59E-16 | 1.04E-03 | 1.61E-01 | 6.87E-02 | 5.93E-07 | 6.84E-01 | 1.26E-03 | 1.98E-07 | 2.27E-03 |
| CD79A | 2.30E-01 | 2.23E-02 | 3.97E-03 | 3.92E-03 | 9.31E-02 | 1.29E-02 | 4.36E-03 | 3.36E-01 | 2.23E-01 | 3.70E-02 | 1.99E-07 | 2.28E-03 |
| MSL3 | 5.95E-04 | 2.66E-03 | 3.89E-04 | 4.15E-02 | 2.86E-03 | 4.10E-01 | 8.75E-02 | 3.25E-02 | 3.71E-01 | 1.93E-02 | 2.01E-07 | 2.31E-03 |
| ACSL4 | 3.21E-06 | 1.28E-01 | 2.65E-04 | 6.62E-07 | 2.29E-04 | 2.12E-01 | 4.38E-04 | 6.21E-03 | 2.32E-04 | 6.23E-01 | 2.02E-07 | 2.32E-03 |
| PPIC | 1.44E-04 | 6.87E-03 | 3.32E-04 | 6.31E-08 | 9.14E-03 | 4.37E-01 | 3.59E-03 | 9.30E-04 | 1.53E-06 | 5.12E-01 | 2.03E-07 | 2.33E-03 |
| FBXL7 | 2.89E-02 | 2.40E-02 | 3.73E-03 | 8.87E-04 | 1.20E-03 | 4.44E-01 | 8.86E-02 | 4.41E-02 | 2.15E-01 | 1.20E-01 | 2.05E-07 | 2.35E-03 |
| COL4A4 | 2.26E-08 | 2.26E-04 | 1.18E-12 | 9.01E-08 | 5.08E-05 | 2.43E-01 | 3.93E-06 | 1.10E-09 | 1.64E-01 | 5.64E-01 | 2.06E-07 | 2.37E-03 |
| SLC25A24 | 2.32E-07 | 1.86E-05 | 4.63E-04 | 2.26E-09 | 1.49E-04 | 2.91E-02 | 1.89E-06 | 3.62E-08 | 9.26E-01 | 1.59E-02 | 2.06E-07 | 2.37E-03 |
| ARHGAP9 | 3.16E-01 | 3.83E-04 | 5.30E-05 | 2.18E-04 | 9.67E-04 | 2.16E-01 | 1.05E-06 | 3.53E-01 | 8.16E-02 | 4.26E-03 | 2.09E-07 | 2.40E-03 |
| CLSTN1 | 2.68E-09 | 9.05E-04 | 4.57E-02 | 1.64E-06 | 9.14E-03 | 7.52E-01 | 4.92E-05 | 6.76E-02 | 9.21E-02 | 5.75E-03 | 2.10E-07 | 2.41E-03 |
| PYCARD | 1.95E-03 | 3.05E-06 | 1.68E-02 | 1.30E-04 | 5.70E-04 | 2.16E-01 | 3.30E-01 | 1.48E-02 | 3.82E-01 | 1.11E-02 | 2.10E-07 | 2.41E-03 |
| CDH6 | 1.81E-15 | 1.26E-07 | 9.08E-23 | 9.75E-11 | 1.08E-07 | 1.15E-03 | 6.17E-09 | 7.56E-08 | 5.95E-02 | 9.13E-01 | 2.12E-07 | 2.43E-03 |
| LAMA2 | 6.97E-16 | 1.57E-08 | 4.07E-24 | 6.27E-08 | 1.09E-03 | 3.89E-01 | 3.89E-09 | 2.35E-09 | 1.83E-02 | 5.67E-01 | 2.14E-07 | 2.45E-03 |
| KCTD9 | 5.99E-02 | 4.90E-03 | 5.88E-02 | 2.21E-02 | 1.37E-02 | 4.22E-01 | 9.18E-02 | 2.83E-02 | 9.51E-02 | 1.79E-01 | 2.15E-07 | 2.46E-03 |
| CD53 | 1.84E-03 | 1.17E-04 | 5.16E-02 | 7.09E-06 | 2.19E-01 | 2.51E-01 | 4.17E-03 | 3.75E-01 | 5.28E-02 | 2.39E-02 | 2.21E-07 | 2.53E-03 |
| FRMD6 | 2.59E-03 | 1.68E-06 | 1.28E-05 | 7.19E-11 | 9.46E-05 | 3.36E-02 | 4.65E-04 | 9.05E-07 | 9.06E-01 | 3.50E-02 | 2.21E-07 | 2.53E-03 |
| ALOX5AP | 2.16E-01 | 1.00E-07 | 8.82E-07 | 1.51E-12 | 7.04E-02 | 1.32E-01 | 5.23E-02 | 9.26E-02 | 3.75E-01 | 4.16E-02 | 2.24E-07 | 2.56E-03 |
| MYOF | 1.82E-04 | 1.62E-06 | 2.58E-05 | 1.99E-09 | 1.02E-04 | 7.53E-01 | 1.69E-07 | 1.14E-03 | 1.38E-01 | 9.00E-02 | 2.29E-07 | 2.63E-03 |
| TBC1D10C | 6.11E-02 | 2.33E-02 | 3.00E-07 | 1.49E-02 | 9.11E-03 | 3.44E-01 | 3.03E-02 | 2.05E-01 | 2.94E-01 | 1.34E-03 | 2.31E-07 | 2.65E-03 |
| FCAMR | 1.77E-01 | 1.72E-01 | 2.44E-08 | 9.69E-02 | 2.57E-01 | 2.28E-01 | 1.37E-05 | 1.40E-02 | 1.60E-04 | 4.19E-02 | 2.41E-07 | 2.77E-03 |
| SLC2A6 | 3.27E-05 | 3.89E-02 | 3.60E-04 | 4.39E-01 | 1.34E-02 | 1.57E-01 | 1.02E-01 | 2.71E-02 | 2.12E-01 | 3.08E-04 | 2.47E-07 | 2.84E-03 |
| MSN | 7.90E-04 | 1.17E-01 | 2.72E-04 | 2.36E-08 | 4.03E-02 | 3.96E-01 | 1.42E-01 | 3.16E-02 | 1.62E-01 | 9.95E-02 | 2.49E-07 | 2.86E-03 |
| PIK3R3 | 8.75E-03 | 3.51E-01 | 7.06E-08 | 1.98E-09 | 4.63E-05 | 6.30E-02 | 9.97E-02 | 8.22E-03 | 4.37E-01 | 2.43E-02 | 2.55E-07 | 2.93E-03 |
| FKBP1B | 8.54E-02 | 1.50E-05 | 3.44E-04 | 6.29E-03 | 3.16E-01 | 4.82E-01 | 7.20E-02 | 4.85E-03 | 1.15E-02 | 1.57E-02 | 2.59E-07 | 2.97E-03 |
| RCC2 | 4.60E-06 | 4.50E-02 | 4.32E-09 | 1.76E-04 | 2.24E-05 | 3.29E-01 | 7.64E-11 | 7.78E-02 | 5.43E-01 | 4.84E-04 | 2.64E-07 | 3.02E-03 |
| GOLM1 | 3.23E-09 | 3.66E-06 | 1.05E-10 | 9.47E-11 | 9.71E-05 | 3.59E-01 | 6.83E-03 | 6.38E-06 | 4.72E-01 | 1.57E-01 | 2.64E-07 | 3.03E-03 |
| DNALI1 | 1.91E-02 | 3.56E-04 | 4.13E-03 | 2.13E-03 | 1.88E-01 | 3.85E-02 | 2.00E-01 | 6.07E-03 | 8.70E-03 | 5.29E-01 | 2.65E-07 | 3.04E-03 |
| B3GNT3 | 3.93E-01 | 4.62E-05 | 6.10E-19 | 2.06E-11 | 8.56E-03 | 5.71E-02 | 1.80E-05 | 1.46E-01 | 3.34E-02 | 3.59E-01 | 2.69E-07 | 3.08E-03 |
| CTSG | 1.22E-01 | 2.24E-05 | 2.64E-02 | 1.20E-02 | 1.03E-01 | 1.57E-01 | 1.80E-01 | 2.52E-01 | 3.04E-03 | 1.42E-01 | 2.72E-07 | 3.11E-03 |
| ADPRH | 4.56E-02 | 7.01E-04 | 2.29E-02 | 1.69E-09 | 4.91E-03 | 3.05E-01 | 5.68E-02 | 3.31E-01 | 1.26E-01 | 1.07E-01 | 2.76E-07 | 3.16E-03 |
| FLNA | 5.33E-08 | 1.22E-04 | 4.58E-06 | 1.22E-06 | 4.13E-04 | 5.16E-01 | 1.20E-09 | 1.65E-05 | 4.77E-01 | 7.77E-03 | 2.79E-07 | 3.20E-03 |
| MCM3 | 3.80E-01 | 1.29E-04 | 1.53E-03 | 1.53E-06 | 1.53E-03 | 4.60E-02 | 4.26E-02 | 8.86E-02 | 6.71E-02 | 3.76E-01 | 2.86E-07 | 3.27E-03 |
| CALHM2 | 7.41E-02 | 1.30E-06 | 2.28E-06 | 5.58E-03 | 1.46E-03 | 4.56E-01 | 8.63E-04 | 1.16E-02 | 4.39E-01 | 1.57E-02 | 2.87E-07 | 3.29E-03 |
| PDGFD | 4.50E-16 | 2.40E-05 | 2.25E-11 | 1.88E-09 | 2.33E-05 | 1.79E-03 | 3.77E-06 | 1.55E-09 | 3.42E-02 | 9.69E-01 | 2.90E-07 | 3.32E-03 |
| LPCAT2 | 4.33E-03 | 1.38E-03 | 3.12E-01 | 1.04E-02 | 1.15E-03 | 2.16E-01 | 5.84E-03 | 1.10E-03 | 9.92E-02 | 3.54E-01 | 2.92E-07 | 3.34E-03 |
| UGCG | 6.12E-02 | 4.01E-03 | 6.99E-02 | 3.53E-16 | 1.79E-02 | 2.88E-01 | 1.76E-01 | 4.90E-04 | 3.12E-01 | 7.79E-02 | 2.98E-07 | 3.41E-03 |
| ENPP5 | 5.00E-09 | 2.58E-04 | 5.88E-13 | 4.88E-07 | 8.84E-08 | 5.45E-02 | 5.22E-03 | 7.01E-09 | 3.50E-03 | 9.45E-01 | 3.00E-07 | 3.43E-03 |
| PRKX | 2.18E-09 | 7.35E-03 | 1.83E-10 | 3.55E-08 | 3.30E-04 | 7.87E-01 | 2.42E-03 | 6.02E-03 | 7.09E-02 | 1.35E-01 | 3.03E-07 | 3.47E-03 |
| ROBO1 | 1.01E-11 | 2.25E-05 | 1.06E-04 | 1.14E-07 | 7.95E-04 | 1.73E-02 | 1.60E-05 | 4.69E-07 | 9.15E-01 | 7.75E-02 | 3.06E-07 | 3.50E-03 |
| ARPC4 | 1.18E-01 | 3.21E-06 | 1.30E-01 | 1.48E-08 | 3.45E-01 | 1.75E-01 | 1.60E-01 | 7.64E-02 | 5.86E-03 | 1.13E-03 | 3.07E-07 | 3.52E-03 |
| OLFML3 | 9.55E-04 | 7.31E-08 | 4.15E-02 | 1.27E-03 | 4.16E-01 | 3.78E-02 | 4.74E-02 | 5.42E-02 | 3.09E-01 | 1.06E-01 | 3.19E-07 | 3.65E-03 |
| RHBDF1 | 4.78E-01 | 8.53E-03 | 3.97E-17 | 1.43E-03 | 6.70E-06 | 3.25E-01 | 8.91E-09 | 2.86E-02 | 1.76E-01 | 1.43E-03 | 3.32E-07 | 3.80E-03 |
| ANXA3 | 6.82E-01 | 4.84E-03 | 2.10E-12 | 6.19E-07 | 3.45E-05 | 2.78E-03 | 2.74E-05 | 1.93E-05 | 3.12E-01 | 1.92E-02 | 3.40E-07 | 3.88E-03 |
| CTSA | 5.93E-03 | 6.98E-06 | 6.84E-03 | 6.27E-05 | 2.91E-01 | 3.96E-01 | 3.95E-05 | 1.48E-01 | 1.93E-04 | 1.72E-01 | 3.41E-07 | 3.90E-03 |
| VWF | 7.14E-05 | 7.38E-06 | 3.78E-13 | 1.15E-23 | 3.59E-07 | 7.55E-01 | 7.47E-08 | 3.03E-06 | 2.47E-01 | 2.00E-02 | 3.44E-07 | 3.94E-03 |
| ATP1A1 | 4.81E-02 | 1.31E-07 | 8.50E-02 | 2.23E-08 | 3.90E-03 | 7.28E-01 | 1.98E-02 | 4.69E-03 | 9.60E-02 | 3.97E-02 | 3.53E-07 | 4.03E-03 |
| KCNJ16 | 2.48E-05 | 4.33E-03 | 7.64E-14 | 2.78E-02 | 2.30E-05 | 1.28E-02 | 9.20E-04 | 1.02E-06 | 1.41E-02 | 9.68E-01 | 3.63E-07 | 4.15E-03 |
| THAP10 | 1.67E-01 | 2.93E-02 | 3.21E-02 | 1.90E-04 | 2.40E-02 | 2.61E-01 | 2.56E-01 | 4.23E-02 | 2.10E-01 | 6.72E-03 | 3.66E-07 | 4.18E-03 |
| ITGB5 | 7.77E-02 | 7.15E-04 | 1.63E-02 | 6.11E-10 | 2.16E-03 | 3.69E-01 | 5.03E-09 | 2.45E-03 | 6.43E-03 | 5.54E-01 | 3.66E-07 | 4.19E-03 |
| RGS10 | 9.73E-02 | 1.44E-06 | 3.29E-05 | 2.08E-05 | 2.21E-02 | 4.36E-01 | 1.39E-03 | 1.43E-03 | 4.58E-01 | 1.39E-02 | 3.71E-07 | 4.24E-03 |
| TPM2 | 3.68E-08 | 1.51E-01 | 6.09E-08 | 3.43E-03 | 9.91E-02 | 5.60E-01 | 1.61E-03 | 4.94E-03 | 1.83E-01 | 2.96E-02 | 3.79E-07 | 4.34E-03 |
| VOPP1 | 4.31E-01 | 7.47E-06 | 1.14E-03 | 1.07E-04 | 5.06E-03 | 5.72E-02 | 2.46E-01 | 1.86E-04 | 2.92E-01 | 7.37E-04 | 3.82E-07 | 4.36E-03 |
| PAQR4 | 7.25E-01 | 8.42E-08 | 6.54E-10 | 2.19E-05 | 6.15E-04 | 2.55E-01 | 6.19E-07 | 4.16E-02 | 5.08E-04 | 1.10E-02 | 3.83E-07 | 4.38E-03 |
| VWA5A | 2.01E-01 | 5.30E-03 | 6.70E-10 | 3.31E-04 | 1.03E-03 | 4.85E-01 | 3.96E-03 | 1.45E-02 | 5.77E-05 | 3.23E-01 | 3.85E-07 | 4.40E-03 |
| ARHGEF2 | 7.10E-01 | 1.23E-03 | 6.51E-03 | 2.88E-04 | 1.59E-03 | 1.38E-01 | 4.82E-06 | 4.37E-03 | 1.67E-01 | 7.55E-03 | 3.95E-07 | 4.51E-03 |
| PRICKLE1 | 9.97E-05 | 1.07E-02 | 2.26E-11 | 4.05E-05 | 2.81E-03 | 5.52E-02 | 2.23E-06 | 2.73E-05 | 3.45E-01 | 6.23E-01 | 3.98E-07 | 4.54E-03 |
| PAQR8 | 4.89E-02 | 2.63E-01 | 8.41E-02 | 9.54E-04 | 7.11E-03 | 1.07E-01 | 5.88E-03 | 8.33E-02 | 4.32E-01 | 8.42E-03 | 4.09E-07 | 4.67E-03 |
| SLC1A7 | 4.12E-01 | 5.17E-04 | 1.80E-05 | 1.39E-04 | 1.92E-02 | 5.32E-01 | 2.42E-03 | 6.65E-04 | 5.57E-02 | 1.84E-02 | 4.11E-07 | 4.69E-03 |
| CTSC | 2.14E-02 | 4.68E-06 | 1.54E-01 | 6.05E-07 | 2.66E-02 | 2.22E-01 | 3.91E-03 | 4.01E-02 | 1.83E-01 | 3.92E-01 | 4.21E-07 | 4.81E-03 |
| SGCB | 1.41E-01 | 1.49E-01 | 6.05E-02 | 1.15E-01 | 1.34E-01 | 2.15E-01 | 2.42E-03 | 7.49E-02 | 1.04E-01 | 4.64E-02 | 4.24E-07 | 4.85E-03 |
| CCL2 | 1.37E-01 | 2.15E-11 | 9.57E-05 | 1.98E-28 | 4.32E-03 | 4.08E-02 | 1.14E-07 | 3.02E-03 | 8.56E-01 | 5.79E-03 | 4.36E-07 | 4.97E-03 |
| STXBP1 | 1.08E-06 | 1.54E-01 | 7.56E-08 | 1.25E-03 | 7.63E-05 | 1.89E-01 | 5.57E-03 | 1.49E-01 | 3.61E-03 | 5.45E-01 | 4.38E-07 | 5.00E-03 |
| QSOX1 | 5.87E-04 | 1.39E-07 | 2.10E-07 | 3.10E-05 | 3.21E-02 | 5.09E-01 | 4.78E-07 | 1.96E-02 | 4.34E-01 | 5.35E-02 | 4.42E-07 | 5.05E-03 |
| SH3BGRL | 2.30E-08 | 5.47E-09 | 2.58E-01 | 3.78E-09 | 7.40E-02 | 1.95E-02 | 2.58E-01 | 7.35E-03 | 6.01E-03 | 4.27E-01 | 4.44E-07 | 5.07E-03 |
| DYNC2H1 | 7.06E-02 | 7.82E-02 | 2.21E-03 | 2.68E-02 | 1.91E-01 | 1.90E-01 | 4.48E-05 | 1.70E-01 | 2.76E-01 | 4.52E-02 | 4.47E-07 | 5.10E-03 |
| LEFTY1 | 2.11E-01 | 2.28E-03 | 2.87E-05 | 1.62E-02 | 2.75E-01 | 6.92E-03 | 7.35E-02 | 6.48E-02 | 1.39E-01 | 2.62E-01 | 4.49E-07 | 5.13E-03 |
| LTB | 2.58E-11 | 6.95E-03 | 6.02E-05 | 3.29E-03 | 8.39E-03 | 5.42E-02 | 5.78E-01 | 4.30E-02 | 3.56E-01 | 1.64E-03 | 4.53E-07 | 5.17E-03 |
| AKR1B10 | 1.97E-20 | 4.17E-09 | 2.85E-19 | 1.04E-10 | 4.01E-02 | 1.00E+00 | 1.16E-07 | 1.64E-04 | 5.06E-07 | 1.19E-02 | 4.58E-07 | 5.23E-03 |
| KRT8 | 5.34E-01 | 7.24E-04 | 5.05E-05 | 1.23E-09 | 1.03E-01 | 1.97E-01 | 5.30E-02 | 4.59E-03 | 7.61E-02 | 8.96E-02 | 4.84E-07 | 5.52E-03 |
| TCEAL3 | 3.78E-01 | 1.15E-04 | 3.63E-04 | 2.64E-06 | 5.22E-03 | 3.06E-01 | 1.14E-01 | 7.89E-02 | 1.12E-01 | 6.36E-02 | 4.92E-07 | 5.61E-03 |
| KCNAB2 | 1.70E-01 | 1.12E-03 | 7.31E-11 | 9.11E-05 | 1.80E-02 | 8.13E-01 | 2.24E-05 | 7.56E-03 | 1.64E-02 | 3.63E-02 | 5.05E-07 | 5.77E-03 |
| TGFB2 | 2.00E-04 | 2.46E-05 | 4.59E-13 | 1.21E-05 | 1.88E-08 | 7.72E-03 | 1.13E-02 | 9.04E-06 | 5.24E-02 | 9.91E-01 | 5.06E-07 | 5.77E-03 |
| AMOTL2 | 1.81E-01 | 4.69E-02 | 7.40E-07 | 4.05E-05 | 1.25E-03 | 1.45E-01 | 5.07E-03 | 8.74E-03 | 6.62E-01 | 1.62E-02 | 5.22E-07 | 5.96E-03 |
| FZD7 | 2.37E-01 | 6.45E-03 | 3.32E-04 | 1.13E-04 | 1.02E-02 | 1.39E-01 | 9.43E-03 | 2.79E-10 | 3.23E-01 | 3.40E-01 | 5.23E-07 | 5.96E-03 |
| PTGIS | 1.60E-08 | 2.36E-08 | 7.52E-08 | 7.52E-09 | 9.39E-02 | 8.94E-01 | 1.42E-09 | 6.43E-04 | 7.19E-02 | 5.84E-03 | 5.26E-07 | 6.00E-03 |
| OLFML1 | 5.12E-03 | 1.28E-04 | 8.02E-02 | 1.73E-01 | 2.97E-01 | 1.13E-01 | 3.30E-01 | 1.68E-02 | 2.30E-02 | 3.16E-02 | 5.38E-07 | 6.13E-03 |
| TREM2 | 1.97E-02 | 3.23E-06 | 4.51E-08 | 1.45E-03 | 2.60E-01 | 7.52E-01 | 9.47E-05 | 1.64E-03 | 1.79E-02 | 1.66E-02 | 5.40E-07 | 6.15E-03 |
| PDLIM3 | 1.21E-01 | 7.42E-04 | 1.15E-06 | 1.20E-21 | 2.43E-01 | 1.17E-01 | 9.79E-04 | 1.07E-03 | 5.42E-01 | 4.69E-02 | 5.56E-07 | 6.34E-03 |
| RERG | 1.31E-06 | 5.12E-01 | 1.44E-04 | 4.76E-04 | 2.81E-03 | 5.57E-02 | 1.12E-02 | 1.70E-05 | 4.22E-01 | 6.89E-02 | 5.59E-07 | 6.37E-03 |
| HTRA1 | 7.21E-03 | 1.12E-03 | 4.45E-08 | 2.90E-11 | 1.51E-01 | 3.67E-01 | 3.57E-01 | 4.01E-02 | 5.12E-02 | 9.94E-02 | 5.64E-07 | 6.43E-03 |
| EVC | 1.85E-03 | 3.18E-01 | 3.12E-04 | 2.12E-05 | 3.46E-02 | 5.94E-01 | 3.15E-07 | 2.55E-03 | 7.96E-02 | 4.44E-02 | 5.73E-07 | 6.53E-03 |
| DYNLL1 | 1.71E-01 | 1.70E-06 | 3.28E-02 | 1.09E-07 | 2.27E-02 | 3.51E-01 | 4.64E-01 | 1.48E-02 | 6.11E-03 | 1.28E-02 | 5.74E-07 | 6.54E-03 |
| CCR2 | 2.31E-05 | 1.40E-07 | 2.11E-09 | 5.14E-02 | 5.29E-03 | 2.00E-02 | 2.32E-03 | 2.82E-03 | 7.14E-02 | 9.24E-01 | 5.81E-07 | 6.62E-03 |
| SLC26A2 | 4.73E-01 | 6.85E-06 | 3.38E-02 | 2.42E-04 | 1.93E-04 | 4.35E-01 | 7.31E-06 | 3.46E-07 | 1.31E-01 | 5.59E-03 | 5.89E-07 | 6.72E-03 |
| HES4 | 4.43E-01 | 1.95E-04 | 7.14E-07 | 3.01E-04 | 9.46E-06 | 4.87E-02 | 1.24E-01 | 9.25E-03 | 4.42E-01 | 1.67E-02 | 6.18E-07 | 7.04E-03 |
| KRT19 | 3.63E-01 | 1.24E-01 | 1.72E-06 | 8.04E-04 | 3.92E-03 | 5.54E-02 | 5.76E-03 | 1.33E-03 | 4.55E-01 | 7.53E-02 | 6.20E-07 | 7.06E-03 |
| OSBPL3 | 4.14E-07 | 6.47E-04 | 2.04E-11 | 8.12E-04 | 1.51E-04 | 3.23E-01 | 2.38E-05 | 4.52E-02 | 6.29E-02 | 6.52E-01 | 6.22E-07 | 7.09E-03 |
| COL8A2 | 1.73E-04 | 4.36E-10 | 7.55E-11 | 1.83E-07 | 9.80E-04 | 3.87E-01 | 4.17E-02 | 6.22E-01 | 2.32E-02 | 1.01E-02 | 6.25E-07 | 7.12E-03 |
| CTHRC1 | 2.30E-01 | 1.17E-04 | 9.21E-16 | 7.06E-03 | 2.02E-02 | 4.53E-01 | 2.40E-01 | 1.12E-06 | 5.78E-02 | 8.24E-02 | 6.59E-07 | 7.51E-03 |
| PALLD | 4.94E-04 | 8.77E-02 | 8.59E-06 | 1.19E-05 | 7.45E-04 | 7.12E-01 | 1.34E-01 | 1.69E-09 | 1.25E-01 | 3.16E-02 | 6.62E-07 | 7.55E-03 |
| VCL | 4.51E-01 | 9.51E-02 | 3.82E-04 | 9.06E-07 | 3.54E-03 | 3.90E-01 | 1.38E-03 | 1.38E-05 | 7.06E-02 | 8.00E-02 | 6.66E-07 | 7.58E-03 |
| LIMK2 | 1.16E-01 | 3.03E-04 | 8.65E-11 | 1.70E-07 | 1.61E-06 | 3.10E-01 | 1.85E-03 | 2.87E-02 | 6.34E-01 | 2.16E-03 | 6.70E-07 | 7.63E-03 |
| DOCK8 | 8.01E-06 | 2.17E-02 | 4.79E-03 | 1.53E-09 | 2.09E-02 | 8.65E-01 | 6.34E-05 | 1.49E-03 | 2.51E-02 | 1.56E-01 | 6.79E-07 | 7.73E-03 |
| ABHD12 | 3.75E-02 | 1.64E-03 | 2.20E-04 | 1.69E-06 | 2.46E-01 | 1.82E-01 | 1.25E-04 | 2.42E-01 | 3.79E-01 | 7.36E-03 | 6.87E-07 | 7.83E-03 |
| SLC7A6OS | 3.25E-03 | 2.22E-01 | 3.60E-03 | 9.80E-04 | 9.41E-02 | 4.63E-01 | 1.22E-01 | 1.32E-01 | 7.93E-03 | 4.82E-02 | 6.91E-07 | 7.87E-03 |
| LYZ | 4.33E-10 | 2.61E-07 | 2.57E-03 | 1.92E-09 | 3.97E-01 | 4.29E-01 | 3.85E-02 | 6.07E-03 | 1.19E-01 | 1.06E-01 | 7.08E-07 | 8.06E-03 |
| SLC44A3 | 6.95E-07 | 5.75E-06 | 1.61E-06 | 9.99E-08 | 3.00E-06 | 2.67E-01 | 1.63E-03 | 6.66E-04 | 3.44E-06 | 8.30E-01 | 7.15E-07 | 8.14E-03 |
| TMEM106C | 4.81E-01 | 3.89E-09 | 2.04E-04 | 5.87E-04 | 5.18E-02 | 4.06E-01 | 1.66E-03 | 9.51E-04 | 2.87E-02 | 1.30E-01 | 7.25E-07 | 8.25E-03 |
| PARP8 | 7.87E-08 | 2.90E-04 | 4.74E-04 | 1.62E-07 | 3.72E-02 | 3.85E-01 | 9.40E-04 | 4.29E-02 | 5.87E-01 | 5.05E-02 | 7.42E-07 | 8.44E-03 |
| PLXDC2 | 2.06E-09 | 2.62E-07 | 6.99E-04 | 7.18E-08 | 6.85E-02 | 5.92E-01 | 1.64E-04 | 5.44E-04 | 1.13E-01 | 3.31E-01 | 7.53E-07 | 8.57E-03 |
| HPS1 | 1.83E-04 | 4.08E-02 | 1.23E-03 | 3.36E-01 | 1.17E-01 | 3.02E-01 | 9.95E-05 | 2.31E-01 | 5.59E-02 | 2.40E-02 | 7.74E-07 | 8.81E-03 |
| MAD2L1 | 7.71E-01 | 1.14E-04 | 7.41E-05 | 4.60E-02 | 5.47E-02 | 2.07E-01 | 1.90E-02 | 2.08E-03 | 3.15E-03 | 5.69E-03 | 7.77E-07 | 8.84E-03 |
| POF1B | 2.40E-03 | 6.31E-02 | 6.09E-09 | 3.17E-05 | 1.52E-01 | 1.43E-01 | 7.53E-07 | 4.34E-04 | 7.24E-01 | 2.94E-02 | 8.16E-07 | 9.28E-03 |
| NES | 5.87E-01 | 1.46E-03 | 3.33E-09 | 2.11E-06 | 1.61E-03 | 1.08E-01 | 1.07E-06 | 1.42E-03 | 3.50E-01 | 6.63E-02 | 8.22E-07 | 9.35E-03 |
| DNAJB4 | 1.96E-01 | 3.55E-01 | 1.08E-01 | 1.57E-09 | 4.66E-02 | 6.72E-02 | 2.49E-01 | 2.56E-04 | 8.15E-03 | 8.62E-02 | 8.26E-07 | 9.40E-03 |
| MVP | 3.59E-01 | 4.57E-01 | 1.20E-06 | 8.48E-05 | 2.87E-02 | 1.18E-01 | 3.62E-05 | 1.17E-01 | 3.46E-03 | 3.22E-02 | 8.29E-07 | 9.43E-03 |
| GLRB | 1.83E-01 | 3.16E-02 | 1.07E-03 | 9.60E-04 | 5.16E-02 | 3.48E-02 | 1.28E-01 | 1.59E-01 | 1.14E-01 | 4.14E-01 | 8.48E-07 | 9.65E-03 |
| ARPC5 | 1.38E-02 | 5.11E-06 | 2.32E-02 | 5.10E-08 | 1.39E-01 | 1.32E-01 | 1.02E-01 | 3.71E-01 | 2.08E-02 | 3.17E-01 | 8.49E-07 | 9.66E-03 |
| TMEM144 | 1.42E-02 | 2.10E-06 | 2.48E-06 | 1.42E-03 | 5.33E-03 | 1.94E-01 | 2.03E-02 | 4.23E-02 | 1.32E-03 | 8.43E-01 | 8.68E-07 | 9.87E-03 |
| PIWIL4 | 1.83E-02 | 2.07E-03 | 1.82E-13 | 2.86E-05 | 8.70E-03 | 1.03E-01 | 2.22E-04 | 5.58E-02 | 5.83E-02 | 8.76E-01 | 8.71E-07 | 9.90E-03 |
| ZMAT3 | 5.97E-11 | 2.31E-07 | 1.39E-08 | 4.24E-14 | 3.66E-03 | 4.33E-01 | 9.40E-07 | 7.57E-07 | 7.39E-05 | 6.87E-01 | 8.89E-07 | 1.01E-02 |
| EDNRA | 1.06E-02 | 2.92E-02 | 1.28E-01 | 3.01E-02 | 2.05E-02 | 8.27E-02 | 3.22E-02 | 4.08E-03 | 6.48E-01 | 1.40E-01 | 8.90E-07 | 1.01E-02 |
| APOL3 | 3.04E-04 | 1.35E-01 | 2.82E-05 | 6.20E-01 | 9.12E-02 | 1.88E-01 | 3.24E-05 | 5.60E-03 | 1.84E-08 | 8.65E-02 | 9.10E-07 | 1.03E-02 |
| PTPDC1 | 2.69E-01 | 2.09E-02 | 1.19E-06 | 5.60E-05 | 1.34E-03 | 2.70E-01 | 6.21E-04 | 7.42E-02 | 4.26E-01 | 6.47E-02 | 9.11E-07 | 1.04E-02 |
| FKBP14 | 2.63E-02 | 3.63E-02 | 1.66E-02 | 1.61E-02 | 4.19E-03 | 9.53E-01 | 2.78E-02 | 9.31E-05 | 1.19E-06 | 4.83E-02 | 9.23E-07 | 1.05E-02 |
| RHOH | 1.28E-03 | 6.45E-02 | 1.62E-04 | 1.04E-02 | 7.05E-02 | 1.35E-02 | 1.22E-02 | 4.91E-01 | 4.53E-01 | 1.23E-02 | 9.27E-07 | 1.05E-02 |
| CLIP4 | 4.45E-08 | 5.73E-04 | 8.81E-05 | 4.50E-14 | 2.86E-02 | 5.11E-01 | 7.29E-07 | 1.81E-04 | 8.06E-02 | 5.09E-01 | 9.34E-07 | 1.06E-02 |
| GPX7 | 4.95E-03 | 1.69E-06 | 1.89E-05 | 1.51E-03 | 2.05E-03 | 7.92E-01 | 1.39E-01 | 9.35E-06 | 1.48E-01 | 4.28E-02 | 9.38E-07 | 1.07E-02 |
| TSPO | 7.22E-02 | 4.51E-09 | 3.54E-02 | 3.20E-03 | 9.00E-02 | 6.16E-01 | 1.19E-02 | 1.40E-01 | 1.54E-01 | 7.82E-03 | 9.40E-07 | 1.07E-02 |
| RAB27B | 8.62E-03 | 8.68E-04 | 1.09E-02 | 1.40E-01 | 9.27E-02 | 1.95E-02 | 7.52E-01 | 6.64E-03 | 7.10E-02 | 2.86E-02 | 9.42E-07 | 1.07E-02 |
| MICAL1 | 1.99E-01 | 5.96E-05 | 6.47E-08 | 4.89E-06 | 4.73E-04 | 3.23E-01 | 6.63E-08 | 3.99E-01 | 1.27E-01 | 8.32E-02 | 9.43E-07 | 1.07E-02 |
| RNASE6 | 1.17E-05 | 1.87E-06 | 7.41E-03 | 1.90E-08 | 1.25E-01 | 4.34E-01 | 5.37E-02 | 2.86E-01 | 5.34E-02 | 1.73E-01 | 9.54E-07 | 1.08E-02 |
| ADD3 | 3.21E-07 | 6.14E-02 | 7.30E-01 | 4.38E-02 | 3.84E-03 | 1.20E-01 | 4.02E-03 | 6.13E-05 | 1.58E-01 | 1.39E-02 | 9.73E-07 | 1.10E-02 |
| CDKN1C | 1.41E-01 | 4.61E-02 | 9.16E-02 | 1.38E-05 | 3.49E-02 | 1.19E-01 | 1.01E-01 | 1.13E-01 | 4.51E-01 | 3.78E-02 | 9.78E-07 | 1.11E-02 |
| KDELR3 | 2.18E-06 | 2.05E-14 | 6.24E-08 | 2.50E-06 | 2.00E-04 | 3.62E-01 | 4.57E-01 | 2.88E-04 | 1.39E-06 | 3.17E-01 | 9.89E-07 | 1.12E-02 |
| HAND2 | 1.19E-02 | 3.17E-04 | 1.79E-02 | 3.75E-04 | 2.64E-02 | 4.30E-01 | 1.60E-04 | 1.71E-02 | 6.20E-01 | 1.42E-02 | 1.00E-06 | 1.14E-02 |
| SLFN13 | 5.14E-08 | 7.94E-08 | 2.52E-11 | 1.72E-07 | 4.00E-05 | 1.09E-01 | 5.56E-04 | 2.60E-03 | 1.85E-01 | 8.46E-01 | 1.05E-06 | 1.19E-02 |
| STK10 | 7.32E-02 | 5.07E-01 | 8.02E-02 | 4.34E-04 | 1.14E-02 | 2.56E-01 | 7.58E-06 | 1.40E-01 | 6.63E-02 | 8.24E-03 | 1.06E-06 | 1.20E-02 |
| IFT80 | 9.77E-05 | 3.47E-01 | 2.75E-03 | 2.48E-01 | 2.53E-04 | 3.03E-01 | 2.32E-05 | 1.38E-07 | 1.52E-01 | 9.26E-02 | 1.08E-06 | 1.23E-02 |
| CKAP2 | 4.46E-06 | 5.56E-02 | 3.63E-03 | 2.95E-02 | 3.36E-01 | 1.43E-02 | 2.23E-02 | 4.16E-04 | 3.95E-04 | 6.87E-01 | 1.11E-06 | 1.26E-02 |
| SEMA3E | 4.96E-01 | 3.54E-04 | 2.85E-09 | 1.36E-03 | 5.45E-03 | 7.39E-02 | 1.56E-04 | 2.70E-02 | 4.62E-02 | 4.99E-01 | 1.12E-06 | 1.27E-02 |
| SLC39A10 | 2.44E-06 | 1.08E-01 | 3.69E-02 | 1.48E-01 | 1.35E-02 | 4.01E-01 | 6.75E-02 | 6.57E-03 | 2.12E-01 | 1.57E-01 | 1.12E-06 | 1.27E-02 |
| SNAP25 | 2.40E-04 | 1.80E-03 | 5.35E-13 | 5.29E-04 | 3.00E-05 | 3.14E-03 | 4.63E-04 | 1.67E-01 | 1.57E-01 | 8.21E-01 | 1.13E-06 | 1.28E-02 |
| FCN1 | 2.71E-01 | 8.04E-04 | 4.00E-02 | 8.31E-05 | 2.61E-01 | 8.13E-02 | 1.12E-03 | 3.19E-01 | 1.58E-01 | 1.84E-02 | 1.13E-06 | 1.28E-02 |
| NPTX2 | 8.12E-01 | 2.75E-04 | 2.28E-12 | 5.30E-10 | 2.31E-02 | 1.28E-02 | 3.74E-02 | 1.27E-04 | 2.22E-01 | 4.40E-02 | 1.13E-06 | 1.28E-02 |
| B3GNT5 | 6.64E-01 | 6.50E-02 | 4.60E-08 | 1.01E-12 | 9.37E-04 | 8.92E-02 | 1.57E-06 | 2.63E-05 | 1.67E-01 | 1.66E-01 | 1.14E-06 | 1.29E-02 |
| COL5A2 | 1.99E-02 | 2.59E-04 | 6.85E-02 | 2.67E-08 | 1.49E-01 | 4.82E-01 | 4.40E-03 | 1.08E-05 | 3.55E-02 | 3.93E-01 | 1.15E-06 | 1.30E-02 |
| TPSAB1 | 2.34E-03 | 3.78E-04 | 2.36E-02 | 5.55E-03 | 2.41E-02 | 6.91E-02 | 3.27E-01 | 4.98E-02 | 4.48E-01 | 2.06E-01 | 1.18E-06 | 1.34E-02 |
| SLC38A1 | 3.95E-08 | 3.57E-05 | 3.30E-07 | 7.24E-09 | 1.02E-03 | 3.37E-01 | 1.95E-06 | 7.40E-06 | 4.98E-02 | 7.71E-01 | 1.20E-06 | 1.36E-02 |
| BEX4 | 1.97E-03 | 8.64E-05 | 3.47E-01 | 1.09E-04 | 6.81E-03 | 6.97E-02 | 8.75E-04 | 3.33E-02 | 1.01E-02 | 6.90E-01 | 1.21E-06 | 1.37E-02 |
| PIK3IP1 | 3.68E-03 | 6.68E-02 | 1.24E-05 | 6.90E-08 | 1.31E-04 | 1.68E-01 | 2.88E-05 | 8.33E-01 | 3.36E-04 | 8.91E-02 | 1.23E-06 | 1.39E-02 |
| GYPC | 1.80E-02 | 2.17E-04 | 1.32E-01 | 1.59E-04 | 1.10E-02 | 4.41E-01 | 4.54E-05 | 3.25E-02 | 5.15E-01 | 1.18E-02 | 1.23E-06 | 1.40E-02 |
| MTHFD2 | 3.09E-01 | 6.52E-08 | 9.61E-05 | 6.00E-14 | 1.65E-01 | 1.75E-01 | 9.04E-04 | 1.10E-06 | 4.95E-01 | 1.74E-02 | 1.24E-06 | 1.40E-02 |
| ERMP1 | 3.43E-04 | 3.62E-02 | 4.88E-05 | 7.21E-04 | 4.16E-03 | 6.60E-01 | 2.11E-01 | 7.86E-05 | 1.37E-01 | 1.15E-01 | 1.26E-06 | 1.43E-02 |
| TCEAL8 | 2.12E-02 | 7.38E-07 | 3.57E-01 | 8.98E-08 | 1.71E-01 | 2.36E-01 | 1.70E-01 | 1.62E-01 | 1.20E-02 | 3.55E-02 | 1.26E-06 | 1.43E-02 |
| PTPRE | 1.57E-02 | 1.01E-02 | 6.60E-02 | 5.08E-07 | 1.15E-03 | 2.47E-01 | 2.23E-03 | 1.51E-02 | 7.35E-01 | 7.79E-02 | 1.33E-06 | 1.51E-02 |
| IGSF3 | 5.62E-01 | 5.22E-03 | 2.62E-13 | 1.73E-05 | 4.09E-05 | 1.32E-01 | 2.84E-05 | 5.52E-02 | 8.73E-03 | 4.08E-01 | 1.34E-06 | 1.51E-02 |
| TPM4 | 2.24E-03 | 2.49E-09 | 2.99E-02 | 2.79E-15 | 4.99E-04 | 2.01E-02 | 3.06E-01 | 2.09E-01 | 5.44E-01 | 5.99E-02 | 1.35E-06 | 1.53E-02 |
| ITGA6 | 5.15E-06 | 2.92E-01 | 2.69E-05 | 4.76E-07 | 1.84E-03 | 5.12E-01 | 2.80E-03 | 1.41E-03 | 1.20E-01 | 2.43E-01 | 1.36E-06 | 1.54E-02 |
| CCDC102B | 1.61E-04 | 7.42E-07 | 4.60E-04 | 2.21E-06 | 5.35E-02 | 1.31E-01 | 2.06E-05 | 2.39E-05 | 1.58E-01 | 8.31E-01 | 1.37E-06 | 1.55E-02 |
| STARD4 | 2.81E-01 | 9.48E-02 | 3.51E-02 | 4.18E-04 | 5.17E-04 | 4.78E-01 | 1.67E-01 | 7.16E-02 | 1.83E-02 | 2.72E-02 | 1.38E-06 | 1.56E-02 |
| TESC | 9.94E-01 | 4.97E-07 | 1.29E-14 | 3.73E-09 | 5.15E-05 | 1.44E-02 | 1.14E-03 | 1.50E-04 | 1.39E-01 | 2.64E-02 | 1.39E-06 | 1.58E-02 |
| TMEM184B | 4.43E-02 | 2.25E-01 | 1.26E-03 | 7.73E-04 | 4.55E-03 | 3.05E-01 | 3.67E-02 | 2.84E-02 | 5.02E-01 | 3.03E-02 | 1.41E-06 | 1.60E-02 |
| MAPK13 | 7.35E-02 | 3.16E-02 | 2.38E-11 | 6.31E-03 | 2.44E-03 | 6.97E-01 | 1.47E-02 | 9.91E-02 | 1.37E-01 | 1.16E-01 | 1.41E-06 | 1.60E-02 |
| DYNLT1 | 2.85E-02 | 1.86E-05 | 2.06E-04 | 7.41E-05 | 6.06E-02 | 3.31E-01 | 7.28E-02 | 5.20E-01 | 2.68E-02 | 1.44E-01 | 1.49E-06 | 1.68E-02 |
| ZNF83 | 4.38E-02 | 5.60E-01 | 2.51E-12 | 1.01E-04 | 3.67E-04 | 1.98E-01 | 1.00E-02 | 3.19E-04 | 4.49E-02 | 3.27E-01 | 1.50E-06 | 1.70E-02 |
| FAIM | 5.16E-01 | 9.25E-05 | 1.26E-09 | 7.64E-09 | 1.96E-03 | 4.98E-01 | 1.02E-01 | 1.31E-03 | 6.19E-03 | 5.87E-02 | 1.50E-06 | 1.70E-02 |
| RIT1 | 3.22E-01 | 5.35E-04 | 9.33E-03 | 3.68E-07 | 7.24E-02 | 1.71E-01 | 1.35E-02 | 8.38E-02 | 3.25E-01 | 1.87E-01 | 1.50E-06 | 1.70E-02 |
| ARMCX2 | 6.18E-01 | 3.33E-03 | 8.46E-04 | 8.90E-02 | 5.54E-05 | 5.78E-02 | 3.59E-01 | 1.56E-02 | 1.91E-02 | 2.27E-02 | 1.51E-06 | 1.71E-02 |
| PLA2G4A | 1.94E-01 | 6.99E-04 | 1.60E-01 | 2.15E-04 | 2.86E-01 | 7.56E-02 | 5.25E-03 | 5.29E-02 | 2.20E-01 | 1.93E-01 | 1.53E-06 | 1.73E-02 |
| SULT1C4 | 8.47E-03 | 3.24E-06 | 9.02E-09 | 4.63E-06 | 3.24E-04 | 2.03E-02 | 3.34E-02 | 1.06E-01 | 2.13E-02 | 9.98E-01 | 1.53E-06 | 1.73E-02 |
| TNFAIP8 | 2.39E-02 | 4.97E-03 | 8.06E-04 | 1.09E-06 | 1.99E-03 | 7.93E-02 | 8.00E-03 | 3.97E-02 | 9.21E-01 | 1.09E-01 | 1.55E-06 | 1.75E-02 |
| ZNF14 | 3.25E-10 | 2.91E-02 | 3.58E-03 | 7.16E-03 | 1.88E-01 | 3.29E-01 | 2.13E-01 | 1.83E-01 | 1.99E-01 | 3.82E-02 | 1.57E-06 | 1.78E-02 |
| SLCO3A1 | 5.12E-04 | 3.75E-04 | 1.51E-06 | 1.41E-06 | 2.04E-05 | 1.80E-01 | 2.82E-07 | 1.05E-02 | 9.94E-01 | 5.97E-03 | 1.59E-06 | 1.80E-02 |
| EZR | 1.34E-02 | 2.50E-01 | 9.94E-09 | 2.02E-12 | 5.52E-01 | 1.58E-01 | 1.81E-02 | 2.79E-03 | 1.95E-01 | 3.02E-03 | 1.60E-06 | 1.81E-02 |
| HOXB2 | 4.04E-02 | 4.57E-01 | 9.74E-04 | 5.30E-04 | 3.86E-02 | 1.67E-01 | 5.61E-03 | 3.93E-01 | 1.38E-02 | 7.60E-02 | 1.60E-06 | 1.81E-02 |
| PLXNC1 | 1.34E-01 | 1.79E-03 | 8.42E-06 | 2.72E-08 | 1.10E-02 | 8.18E-01 | 1.89E-03 | 5.64E-05 | 1.31E-01 | 9.53E-02 | 1.60E-06 | 1.81E-02 |
| GAL3ST4 | 9.81E-01 | 9.29E-04 | 1.52E-11 | 8.45E-03 | 4.85E-03 | 1.11E-01 | 2.32E-05 | 4.10E-05 | 7.74E-02 | 1.04E-02 | 1.62E-06 | 1.83E-02 |
| TMSB4X | 1.14E-01 | 9.00E-08 | 4.46E-02 | 1.03E-05 | 1.01E-01 | 2.53E-01 | 3.43E-01 | 2.28E-01 | 3.04E-03 | 1.10E-01 | 1.65E-06 | 1.87E-02 |
| LGALS4 | 7.02E-02 | 8.10E-05 | 2.62E-06 | 1.46E-06 | 5.29E-03 | 9.43E-01 | 9.21E-04 | 1.21E-01 | 1.16E-06 | 5.62E-02 | 1.66E-06 | 1.87E-02 |
| LOXL1 | 8.27E-01 | 2.95E-09 | 1.72E-24 | 3.88E-05 | 2.73E-04 | 1.79E-01 | 2.01E-07 | 6.02E-10 | 1.40E-01 | 5.15E-02 | 1.69E-06 | 1.91E-02 |
| PIGF | 2.51E-02 | 8.07E-05 | 8.69E-03 | 1.09E-02 | 1.16E-01 | 2.13E-01 | 7.50E-02 | 5.02E-01 | 1.65E-01 | 8.73E-02 | 1.77E-06 | 2.00E-02 |
| SLC12A2 | 2.61E-14 | 9.98E-06 | 3.26E-06 | 2.73E-11 | 3.76E-05 | 1.85E-02 | 2.03E-11 | 8.60E-07 | 1.99E-01 | 9.90E-01 | 1.82E-06 | 2.06E-02 |
| BIN2 | 4.40E-02 | 1.94E-02 | 2.06E-01 | 4.13E-03 | 1.65E-01 | 9.56E-02 | 1.22E-03 | 6.67E-02 | 5.67E-01 | 4.19E-02 | 1.86E-06 | 2.10E-02 |
| ENAH | 5.68E-03 | 7.86E-02 | 2.37E-04 | 4.01E-06 | 5.50E-03 | 6.44E-01 | 2.51E-04 | 8.42E-07 | 3.04E-01 | 1.73E-01 | 1.86E-06 | 2.10E-02 |
| CASP4 | 6.71E-01 | 2.33E-04 | 1.82E-11 | 1.49E-07 | 2.20E-02 | 3.61E-01 | 2.93E-03 | 6.61E-02 | 3.99E-02 | 4.83E-02 | 1.87E-06 | 2.11E-02 |
| NFATC1 | 3.19E-03 | 1.24E-02 | 1.16E-04 | 7.62E-09 | 3.38E-03 | 1.47E-01 | 3.89E-04 | 5.58E-01 | 2.23E-01 | 2.63E-01 | 1.87E-06 | 2.12E-02 |
| SERPINB8 | 3.73E-01 | 4.74E-07 | 8.41E-10 | 4.59E-08 | 1.13E-02 | 7.47E-02 | 2.00E-03 | 3.10E-01 | 1.85E-01 | 2.55E-01 | 1.88E-06 | 2.12E-02 |
| PROM1 | 1.01E-02 | 1.51E-03 | 1.39E-10 | 5.21E-06 | 1.62E-05 | 6.02E-03 | 3.41E-08 | 1.75E-03 | 2.03E-01 | 9.92E-01 | 1.93E-06 | 2.18E-02 |
| TLR5 | 6.28E-02 | 6.33E-03 | 1.79E-06 | 1.57E-02 | 4.04E-03 | 1.85E-01 | 2.89E-11 | 8.32E-02 | 1.48E-02 | 8.44E-01 | 1.93E-06 | 2.18E-02 |
| F2RL1 | 2.20E-01 | 2.70E-05 | 4.50E-06 | 2.01E-11 | 1.50E-03 | 1.11E-02 | 1.35E-05 | 2.96E-03 | 8.37E-03 | 9.72E-01 | 1.95E-06 | 2.20E-02 |
| NRSN2 | 7.39E-01 | 1.58E-03 | 5.33E-09 | 7.12E-06 | 2.24E-04 | 3.37E-02 | 9.41E-02 | 1.42E-01 | 1.89E-01 | 1.77E-02 | 1.96E-06 | 2.21E-02 |
| RGS4 | 1.78E-03 | 1.48E-09 | 3.33E-16 | 3.67E-21 | 6.75E-07 | 2.49E-02 | 6.52E-02 | 6.63E-08 | 1.41E-01 | 9.87E-01 | 2.02E-06 | 2.28E-02 |
| MRAS | 4.76E-07 | 1.44E-02 | 1.47E-07 | 8.49E-07 | 2.76E-02 | 6.62E-01 | 4.58E-01 | 2.13E-03 | 2.37E-02 | 3.40E-02 | 2.04E-06 | 2.31E-02 |
| COL16A1 | 1.00E+00 | 1.47E-06 | 3.02E-22 | 1.31E-04 | 1.71E-03 | 9.12E-02 | 3.14E-12 | 9.10E-07 | 1.23E-01 | 6.43E-03 | 2.05E-06 | 2.32E-02 |
| CYYR1 | 1.48E-07 | 2.07E-06 | 6.43E-04 | 6.96E-10 | 3.99E-06 | 7.13E-02 | 1.34E-01 | 2.35E-07 | 5.59E-02 | 9.61E-01 | 2.05E-06 | 2.32E-02 |
| CORO1A | 6.36E-02 | 2.63E-03 | 2.35E-02 | 2.19E-02 | 1.17E-01 | 2.83E-02 | 5.79E-05 | 5.02E-01 | 4.59E-01 | 5.13E-03 | 2.05E-06 | 2.32E-02 |
| CLEC10A | 2.00E-01 | 6.72E-04 | 9.85E-03 | 7.65E-02 | 1.60E-01 | 1.28E-01 | 1.38E-01 | 4.21E-01 | 5.88E-02 | 2.93E-02 | 2.06E-06 | 2.32E-02 |
| FAS | 2.34E-03 | 3.75E-09 | 1.19E-03 | 2.40E-12 | 4.31E-02 | 3.53E-01 | 5.65E-04 | 1.48E-02 | 2.46E-03 | 8.06E-01 | 2.08E-06 | 2.34E-02 |
| FADD | 1.46E-03 | 1.23E-03 | 1.62E-01 | 1.04E-03 | 3.81E-01 | 2.75E-01 | 2.53E-01 | 6.57E-02 | 3.77E-02 | 4.61E-02 | 2.09E-06 | 2.36E-02 |
| CDCA4 | 2.00E-06 | 6.61E-03 | 2.66E-01 | 5.89E-02 | 6.38E-02 | 1.93E-01 | 7.72E-02 | 2.22E-02 | 5.37E-01 | 4.01E-03 | 2.15E-06 | 2.42E-02 |
| GAS2L3 | 6.27E-02 | 9.12E-03 | 3.94E-03 | 1.31E-06 | 3.73E-01 | 3.79E-01 | 7.17E-06 | 2.54E-04 | 1.39E-01 | 2.61E-01 | 2.15E-06 | 2.43E-02 |
| BTG2 | 7.12E-01 | 2.41E-07 | 1.05E-07 | 5.21E-24 | 2.20E-04 | 1.12E-01 | 5.82E-03 | 6.43E-03 | 1.82E-01 | 2.11E-01 | 2.17E-06 | 2.45E-02 |
| CD5 | 4.02E-05 | 2.43E-03 | 1.68E-05 | 3.57E-07 | 7.99E-02 | 4.50E-02 | 3.89E-05 | 6.71E-01 | 3.57E-01 | 7.45E-02 | 2.20E-06 | 2.48E-02 |
| PDE1A | 1.39E-06 | 3.62E-11 | 6.13E-08 | 5.44E-07 | 5.37E-04 | 3.04E-01 | 7.30E-08 | 3.90E-06 | 1.88E-01 | 7.39E-01 | 2.20E-06 | 2.49E-02 |
| CSGALNACT1 | 8.89E-02 | 5.66E-02 | 1.63E-02 | 1.19E-03 | 5.03E-04 | 3.97E-01 | 5.87E-02 | 4.07E-04 | 5.58E-01 | 5.36E-02 | 2.21E-06 | 2.49E-02 |
| RAB11FIP5 | 4.44E-01 | 1.98E-03 | 3.43E-03 | 5.51E-06 | 6.45E-03 | 2.39E-01 | 3.97E-04 | 2.48E-01 | 4.01E-02 | 2.50E-01 | 2.26E-06 | 2.55E-02 |
| ARHGAP27 | 2.91E-02 | 3.53E-05 | 5.27E-08 | 8.73E-07 | 6.01E-05 | 7.30E-01 | 5.04E-04 | 4.51E-01 | 1.13E-02 | 1.37E-02 | 2.29E-06 | 2.58E-02 |
| RSU1 | 1.48E-02 | 1.91E-03 | 6.39E-02 | 1.53E-06 | 1.99E-01 | 1.55E-01 | 4.69E-01 | 1.35E-01 | 1.13E-01 | 8.50E-02 | 2.31E-06 | 2.60E-02 |
| RAC2 | 1.26E-01 | 1.19E-02 | 1.80E-01 | 8.65E-03 | 2.38E-01 | 3.24E-02 | 3.49E-04 | 5.66E-01 | 1.67E-02 | 5.72E-02 | 2.31E-06 | 2.60E-02 |
| GJA4 | 1.83E-01 | 8.90E-03 | 5.31E-02 | 3.11E-05 | 1.36E-04 | 2.53E-03 | 1.11E-01 | 1.75E-01 | 6.71E-01 | 3.43E-02 | 2.34E-06 | 2.64E-02 |
| PDZK1IP1 | 8.75E-01 | 1.05E-06 | 3.95E-22 | 2.11E-15 | 1.44E-09 | 3.18E-01 | 1.66E-07 | 1.61E-04 | 2.90E-02 | 1.68E-02 | 2.34E-06 | 2.64E-02 |
| COX7A1 | 4.82E-02 | 6.75E-05 | 1.84E-03 | 3.10E-03 | 2.79E-01 | 2.58E-01 | 1.95E-02 | 7.14E-03 | 4.85E-01 | 1.39E-01 | 2.37E-06 | 2.68E-02 |
| FLVCR1 | 5.88E-03 | 4.65E-02 | 2.30E-04 | 1.21E-05 | 6.75E-03 | 4.11E-01 | 4.02E-02 | 6.26E-02 | 6.60E-01 | 8.85E-03 | 2.40E-06 | 2.70E-02 |
| P2RY10 | 4.86E-07 | 1.66E-02 | 1.27E-02 | 1.40E-03 | 2.86E-01 | 9.53E-02 | 2.93E-03 | 3.67E-02 | 1.43E-02 | 7.78E-01 | 2.45E-06 | 2.76E-02 |
| LAMC2 | 8.20E-02 | 2.09E-02 | 5.26E-18 | 7.10E-05 | 7.74E-05 | 5.87E-03 | 1.87E-13 | 1.07E-01 | 8.29E-02 | 9.45E-01 | 2.45E-06 | 2.76E-02 |
| FAR2 | 4.43E-02 | 1.54E-01 | 1.78E-02 | 1.05E-01 | 3.59E-01 | 2.67E-01 | 9.78E-02 | 1.72E-02 | 1.08E-01 | 7.45E-02 | 2.45E-06 | 2.76E-02 |
| RBP7 | 8.66E-03 | 3.94E-04 | 2.96E-03 | 1.36E-09 | 2.42E-03 | 2.14E-01 | 6.63E-01 | 1.13E-02 | 2.91E-01 | 5.04E-02 | 2.46E-06 | 2.77E-02 |
| RTN1 | 1.77E-03 | 4.66E-02 | 2.33E-03 | 1.05E-02 | 2.13E-01 | 1.83E-01 | 1.41E-03 | 3.28E-04 | 3.86E-03 | 7.86E-01 | 2.55E-06 | 2.87E-02 |
| SLC44A2 | 4.16E-02 | 9.54E-02 | 4.28E-01 | 4.95E-03 | 5.35E-03 | 3.60E-01 | 8.07E-02 | 6.02E-02 | 8.49E-02 | 8.87E-02 | 2.56E-06 | 2.89E-02 |
| CD74 | 4.52E-05 | 4.59E-04 | 2.06E-01 | 6.07E-04 | 2.75E-01 | 1.00E-01 | 3.03E-04 | 3.22E-01 | 8.72E-03 | 3.37E-01 | 2.57E-06 | 2.89E-02 |
| S100A13 | 1.07E-03 | 1.62E-12 | 2.88E-03 | 1.01E-06 | 3.97E-03 | 4.08E-01 | 3.82E-02 | 3.51E-04 | 7.28E-01 | 7.21E-02 | 2.67E-06 | 3.01E-02 |
| MAP1B | 4.91E-10 | 3.17E-07 | 1.22E-13 | 2.55E-13 | 9.22E-04 | 2.50E-02 | 1.25E-05 | 9.06E-09 | 2.47E-01 | 9.83E-01 | 2.69E-06 | 3.03E-02 |
| XPNPEP1 | 1.75E-02 | 4.36E-02 | 2.43E-03 | 9.64E-05 | 5.78E-03 | 1.17E-01 | 6.82E-02 | 5.91E-01 | 4.76E-02 | 3.64E-01 | 2.69E-06 | 3.04E-02 |
| SLC4A7 | 1.64E-01 | 4.07E-02 | 1.87E-01 | 7.68E-09 | 1.35E-02 | 5.95E-01 | 1.51E-04 | 1.59E-02 | 2.33E-01 | 6.78E-03 | 2.70E-06 | 3.04E-02 |
| ATP8B2 | 3.89E-01 | 4.66E-03 | 1.16E-04 | 2.90E-02 | 4.74E-04 | 5.72E-01 | 1.71E-08 | 1.38E-01 | 1.14E-01 | 1.03E-02 | 2.71E-06 | 3.05E-02 |
| ATP8B1 | 4.05E-06 | 5.02E-01 | 3.18E-02 | 3.55E-02 | 2.98E-03 | 6.55E-01 | 4.17E-05 | 1.32E-03 | 1.93E-03 | 2.78E-02 | 2.74E-06 | 3.09E-02 |
| CHI3L1 | 1.11E-04 | 2.72E-04 | 8.20E-10 | 1.36E-08 | 7.19E-03 | 2.32E-01 | 1.87E-05 | 5.84E-07 | 9.60E-01 | 5.90E-02 | 2.74E-06 | 3.09E-02 |
| ROR2 | 8.81E-01 | 1.79E-05 | 2.56E-16 | 1.41E-05 | 1.00E-02 | 2.42E-01 | 2.85E-06 | 2.42E-04 | 1.17E-01 | 1.05E-02 | 2.78E-06 | 3.14E-02 |
| SSPN | 7.59E-05 | 2.67E-01 | 4.95E-07 | 1.58E-03 | 1.19E-01 | 2.18E-02 | 2.14E-05 | 1.37E-09 | 8.15E-02 | 7.69E-01 | 2.80E-06 | 3.15E-02 |
| FAT1 | 2.04E-02 | 6.22E-03 | 3.90E-11 | 1.35E-12 | 1.68E-03 | 5.48E-01 | 3.75E-07 | 1.81E-04 | 3.03E-03 | 6.83E-01 | 2.84E-06 | 3.20E-02 |
| KRT23 | 1.04E-03 | 1.17E-10 | 4.47E-28 | 2.49E-37 | 7.58E-05 | 1.85E-01 | 9.63E-06 | 8.88E-03 | 7.61E-02 | 9.93E-01 | 2.87E-06 | 3.23E-02 |
| MMP11 | 8.83E-01 | 2.30E-03 | 1.37E-04 | 1.60E-02 | 7.89E-02 | 2.29E-02 | 6.19E-02 | 2.22E-02 | 1.33E-01 | 4.54E-02 | 2.91E-06 | 3.28E-02 |
| SHISA3 | 4.34E-01 | 3.76E-03 | 3.09E-02 | 3.33E-03 | 4.05E-03 | 3.78E-01 | 1.84E-03 | 1.78E-07 | 3.36E-01 | 7.49E-02 | 2.94E-06 | 3.32E-02 |
| HSPB6 | 1.91E-03 | 2.77E-01 | 1.46E-07 | 2.13E-11 | 2.30E-01 | 5.05E-01 | 3.40E-06 | 2.99E-02 | 6.81E-02 | 1.56E-01 | 2.96E-06 | 3.33E-02 |
| FZD6 | 5.20E-02 | 9.28E-05 | 2.57E-02 | 3.88E-04 | 1.70E-04 | 4.69E-01 | 2.11E-02 | 3.27E-02 | 2.82E-01 | 3.85E-01 | 2.96E-06 | 3.34E-02 |
| FSTL1 | 5.15E-07 | 4.98E-05 | 1.65E-05 | 6.42E-10 | 2.55E-03 | 2.29E-01 | 3.23E-01 | 1.33E-07 | 5.08E-01 | 2.05E-01 | 2.97E-06 | 3.34E-02 |
| PRSS23 | 1.97E-03 | 1.02E-07 | 1.73E-05 | 1.52E-10 | 2.37E-03 | 2.75E-01 | 4.51E-01 | 2.30E-07 | 4.87E-01 | 5.14E-02 | 2.99E-06 | 3.37E-02 |
| MPHOSPH6 | 3.99E-01 | 2.77E-02 | 1.29E-04 | 1.97E-04 | 1.11E-01 | 1.27E-01 | 5.62E-01 | 4.44E-03 | 2.66E-02 | 1.17E-02 | 3.00E-06 | 3.38E-02 |
| TRPV2 | 2.89E-02 | 2.06E-05 | 3.42E-02 | 2.34E-03 | 1.12E-01 | 2.33E-01 | 4.88E-06 | 7.99E-01 | 5.91E-02 | 1.56E-03 | 3.04E-06 | 3.42E-02 |
| XPR1 | 1.40E-02 | 6.56E-03 | 4.68E-02 | 9.81E-03 | 8.92E-02 | 6.13E-01 | 2.46E-01 | 4.20E-05 | 3.84E-03 | 2.43E-01 | 3.06E-06 | 3.44E-02 |
| DAPP1 | 1.73E-03 | 5.97E-03 | 6.19E-02 | 2.75E-05 | 1.21E-01 | 8.16E-02 | 1.69E-04 | 8.87E-03 | 7.03E-02 | 9.22E-01 | 3.08E-06 | 3.47E-02 |
| SPARC | 4.67E-06 | 1.02E-08 | 1.56E-03 | 8.14E-05 | 8.24E-03 | 2.59E-02 | 9.33E-02 | 9.12E-08 | 2.78E-01 | 8.66E-01 | 3.09E-06 | 3.48E-02 |
| RASGRP1 | 1.31E-07 | 7.93E-02 | 7.36E-03 | 4.36E-03 | 4.93E-02 | 1.08E-01 | 7.03E-02 | 4.02E-03 | 1.17E-02 | 9.40E-01 | 3.12E-06 | 3.51E-02 |
| MYOM1 | 4.31E-03 | 4.35E-01 | 4.81E-03 | 3.17E-03 | 5.48E-02 | 2.73E-01 | 4.11E-02 | 1.49E-02 | 4.36E-01 | 1.03E-02 | 3.16E-06 | 3.56E-02 |
| FREM2 | 5.89E-03 | 1.35E-01 | 1.08E-03 | 3.35E-02 | 5.58E-02 | 9.67E-01 | 5.53E-03 | 1.80E-02 | 8.03E-03 | 5.02E-02 | 3.25E-06 | 3.65E-02 |
| TWSG1 | 1.27E-04 | 6.70E-07 | 5.09E-01 | 8.31E-10 | 4.03E-02 | 7.72E-02 | 2.40E-03 | 3.03E-05 | 5.19E-02 | 6.03E-01 | 3.36E-06 | 3.78E-02 |
| C1QTNF7 | 4.65E-03 | 6.90E-01 | 1.77E-06 | 4.93E-02 | 4.53E-02 | 3.47E-02 | 6.90E-03 | 3.38E-07 | 2.35E-01 | 2.22E-01 | 3.44E-06 | 3.87E-02 |
| CKLF | 2.92E-03 | 1.37E-08 | 5.87E-02 | 4.57E-06 | 5.08E-03 | 2.32E-01 | 4.06E-03 | 7.04E-01 | 2.61E-01 | 2.32E-02 | 3.55E-06 | 3.99E-02 |
| GTF2F2 | 8.36E-03 | 1.45E-04 | 1.53E-01 | 2.92E-06 | 4.12E-01 | 1.67E-01 | 3.87E-01 | 7.94E-02 | 6.62E-03 | 8.11E-02 | 3.62E-06 | 4.07E-02 |
| FGD6 | 8.26E-06 | 3.78E-03 | 1.83E-02 | 1.26E-04 | 3.48E-05 | 9.12E-01 | 3.34E-02 | 7.11E-02 | 5.07E-03 | 2.50E-01 | 3.62E-06 | 4.07E-02 |
| CCDC8 | 4.45E-01 | 3.88E-05 | 1.24E-12 | 4.17E-02 | 2.08E-04 | 1.16E-01 | 5.35E-04 | 1.79E-01 | 1.25E-01 | 3.87E-01 | 3.64E-06 | 4.09E-02 |
| FMNL2 | 9.49E-04 | 4.53E-04 | 6.72E-02 | 1.54E-04 | 5.78E-03 | 8.80E-01 | 2.03E-03 | 1.84E-01 | 1.34E-01 | 2.21E-02 | 3.70E-06 | 4.16E-02 |
| SERPINB9 | 1.02E-01 | 2.16E-10 | 5.48E-04 | 2.41E-15 | 7.45E-04 | 9.24E-02 | 2.50E-06 | 7.56E-04 | 8.94E-01 | 2.07E-01 | 3.73E-06 | 4.19E-02 |
| LFNG | 9.81E-01 | 6.77E-04 | 1.19E-04 | 5.16E-02 | 1.15E-02 | 6.25E-02 | 4.96E-04 | 2.71E-02 | 1.63E-01 | 1.14E-03 | 3.76E-06 | 4.23E-02 |
| GLA | 6.49E-01 | 4.04E-08 | 8.78E-07 | 1.82E-09 | 3.23E-01 | 1.11E-01 | 1.51E-02 | 7.15E-02 | 1.44E-02 | 1.16E-01 | 3.78E-06 | 4.25E-02 |
| SFRP4 | 1.26E-05 | 2.01E-02 | 2.31E-16 | 1.32E-06 | 2.00E-01 | 5.09E-01 | 9.30E-04 | 1.72E-03 | 1.06E-01 | 4.62E-01 | 3.78E-06 | 4.25E-02 |
| SFXN3 | 2.89E-01 | 6.09E-04 | 2.03E-07 | 8.20E-07 | 6.84E-06 | 2.53E-01 | 6.84E-03 | 2.71E-04 | 4.29E-01 | 3.21E-01 | 3.80E-06 | 4.27E-02 |
| MCM2 | 1.00E+00 | 6.52E-02 | 5.32E-02 | 8.81E-03 | 1.10E-02 | 8.76E-02 | 4.32E-05 | 2.94E-02 | 2.87E-02 | 1.70E-02 | 3.82E-06 | 4.29E-02 |
| FGD3 | 7.51E-01 | 5.45E-02 | 2.28E-03 | 3.91E-03 | 6.39E-03 | 9.57E-02 | 4.41E-03 | 4.14E-02 | 3.26E-01 | 1.63E-02 | 3.85E-06 | 4.32E-02 |
| OLR1 | 1.74E-01 | 1.15E-02 | 1.30E-12 | 7.02E-08 | 3.82E-02 | 5.00E-01 | 6.45E-05 | 5.18E-06 | 3.96E-03 | 5.74E-01 | 3.87E-06 | 4.35E-02 |
| BIRC3 | 2.12E-01 | 1.37E-03 | 4.31E-05 | 2.16E-09 | 2.28E-03 | 3.84E-01 | 7.90E-05 | 1.17E-03 | 5.71E-01 | 1.31E-01 | 3.88E-06 | 4.35E-02 |
| TPBG | 3.35E-03 | 4.03E-04 | 5.44E-05 | 2.17E-06 | 5.63E-02 | 2.61E-01 | 9.26E-02 | 1.31E-01 | 5.25E-01 | 2.33E-01 | 3.89E-06 | 4.37E-02 |
| DOCK11 | 9.23E-04 | 3.32E-02 | 3.81E-01 | 1.22E-05 | 1.30E-01 | 6.77E-01 | 1.47E-03 | 2.48E-02 | 3.75E-02 | 1.83E-02 | 3.92E-06 | 4.40E-02 |
| WDR53 | 2.38E-01 | 1.76E-02 | 6.18E-03 | 3.73E-03 | 4.50E-02 | 2.56E-01 | 8.50E-02 | 2.64E-01 | 3.24E-01 | 6.53E-02 | 3.95E-06 | 4.43E-02 |
| PPP1R16B | 1.40E-01 | 9.12E-03 | 3.24E-01 | 2.53E-02 | 2.06E-04 | 4.66E-01 | 4.06E-02 | 1.36E-02 | 2.80E-01 | 9.48E-03 | 4.04E-06 | 4.53E-02 |
| PPIH | 7.20E-02 | 7.31E-05 | 4.64E-02 | 4.23E-02 | 4.28E-01 | 2.34E-01 | 1.98E-01 | 1.92E-01 | 1.03E-03 | 9.32E-02 | 4.05E-06 | 4.54E-02 |
| FHOD1 | 6.51E-01 | 1.38E-02 | 3.51E-05 | 1.30E-02 | 1.26E-02 | 2.41E-01 | 1.50E-08 | 3.11E-01 | 6.24E-02 | 5.77E-03 | 4.12E-06 | 4.62E-02 |
| HSPG2 | 8.20E-07 | 3.61E-03 | 9.55E-03 | 4.68E-03 | 1.82E-05 | 9.14E-01 | 1.59E-06 | 1.04E-01 | 1.49E-01 | 1.29E-01 | 4.23E-06 | 4.75E-02 |
| NAP1L3 | 5.87E-02 | 3.24E-05 | 6.52E-02 | 9.46E-04 | 2.22E-01 | 2.84E-01 | 3.10E-01 | 9.94E-02 | 2.87E-02 | 2.46E-01 | 4.26E-06 | 4.78E-02 |
| NQO1 | 9.31E-02 | 5.42E-01 | 3.05E-15 | 2.24E-03 | 4.44E-02 | 1.59E-01 | 1.60E-03 | 1.87E-03 | 4.79E-02 | 4.24E-01 | 4.30E-06 | 4.83E-02 |
| LY75 | 7.55E-07 | 1.75E-02 | 4.04E-02 | 1.04E-04 | 3.94E-02 | 5.19E-02 | 8.49E-01 | 1.91E-01 | 1.23E-01 | 4.90E-03 | 4.31E-06 | 4.84E-02 |
| VAMP5 | 3.64E-01 | 3.85E-12 | 5.54E-03 | 1.28E-02 | 1.33E-01 | 4.61E-01 | 1.21E-01 | 1.56E-01 | 6.30E-02 | 1.15E-03 | 4.35E-06 | 4.88E-02 |
| PSMD10 | 1.66E-01 | 3.24E-02 | 4.71E-01 | 3.27E-03 | 1.58E-02 | 1.44E-01 | 7.79E-02 | 2.19E-01 | 1.45E-04 | 1.90E-01 | 4.38E-06 | 4.92E-02 |
| ITGA9 | 2.62E-08 | 6.69E-04 | 3.39E-01 | 4.57E-05 | 8.09E-02 | 6.79E-01 | 1.20E-06 | 1.67E-06 | 1.98E-01 | 2.21E-02 | 4.41E-06 | 4.95E-02 |
| **Downregulated Genes** | ***P*-value of Individual Dataset** | | | | | | | | | | **Meta *P*-value** | **Adjusted Meta  *P*-value** |
|  | **Gerhard** | **GSE130970** | **GSE135251** | **GSE162694** | **GSE225740** | **GSE185051** | **GSE193066** | **GSE49541** | **GSE89632** | **GSE96971** |  |  |
| SLC16A10 | 1.30E-04 | 4.89E-05 | 2.62E-13 | 3.78E-06 | 3.21E-02 | 1.20E-02 | 1.53E-02 | 1.28E-03 | 3.41E-02 | 2.47E-02 | 1.66E-16 | 1.97E-12 |
| ADCY10 | 2.19E-04 | 2.14E-04 | 1.09E-02 | 7.71E-06 | 1.99E-04 | 4.98E-03 | 3.06E-05 | 7.20E-02 | 1.36E-02 | 4.32E-02 | 1.17E-15 | 1.38E-11 |
| CNDP1 | 5.84E-06 | 4.51E-03 | 1.31E-08 | 3.72E-03 | 2.48E-04 | 4.24E-03 | 4.36E-12 | 2.29E-05 | 1.37E-01 | 3.71E-03 | 2.01E-15 | 2.38E-11 |
| COL28A1 | 3.42E-11 | 2.71E-03 | 1.70E-12 | 1.94E-04 | 6.17E-02 | 7.34E-03 | 9.09E-04 | 6.79E-02 | 6.91E-04 | 6.56E-02 | 3.99E-14 | 4.72E-10 |
| CYP4F3 | 4.64E-03 | 2.27E-03 | 3.54E-05 | 3.34E-04 | 1.58E-04 | 7.92E-02 | 1.99E-04 | 8.46E-02 | 2.53E-02 | 1.16E-02 | 4.24E-14 | 5.02E-10 |
| **NCAM2** | 1.26E-04 | 2.66E-05 | 2.57E-12 | 7.79E-09 | 3.33E-08 | 1.67E-01 | 3.57E-05 | 9.53E-07 | 4.75E-02 | 4.73E-03 | 7.22E-14 | 8.54E-10 |
| CNKSR2 | 3.27E-09 | 7.83E-06 | 2.15E-07 | 5.50E-03 | 6.89E-03 | 1.01E-01 | 5.78E-05 | 1.34E-02 | 6.06E-02 | 3.77E-02 | 9.10E-14 | 1.08E-09 |
| AADAT | 4.92E-03 | 6.08E-02 | 8.39E-10 | 2.18E-05 | 1.99E-02 | 1.34E-01 | 2.08E-03 | 2.03E-04 | 4.63E-03 | 5.78E-03 | 1.24E-13 | 1.47E-09 |
| LARP1B | 9.15E-02 | 2.30E-03 | 1.93E-05 | 1.59E-02 | 1.20E-02 | 6.40E-02 | 8.71E-04 | 3.19E-02 | 3.32E-05 | 3.14E-02 | 2.63E-13 | 3.11E-09 |
| **SLC13A5** | 3.96E-02 | 2.10E-02 | 4.63E-04 | 2.87E-02 | 7.17E-10 | 4.69E-02 | 8.70E-02 | 1.57E-05 | 1.84E-02 | 1.35E-02 | 3.28E-13 | 3.89E-09 |
| RAPGEFL1 | 1.50E-07 | 8.16E-04 | 3.44E-02 | 5.83E-05 | 1.41E-03 | 1.07E-02 | 5.03E-02 | 1.63E-02 | 5.46E-02 | 8.74E-02 | 3.32E-13 | 3.93E-09 |
| SH3PXD2A | 1.07E-03 | 3.02E-02 | 3.29E-04 | 1.97E-03 | 1.03E-01 | 7.95E-03 | 4.27E-04 | 5.10E-02 | 3.13E-02 | 2.92E-02 | 3.37E-13 | 3.98E-09 |
| HORMAD2 | 1.83E-05 | 4.48E-02 | 1.40E-06 | 4.57E-04 | 1.45E-03 | 1.88E-01 | 1.59E-03 | 4.05E-03 | 1.04E-02 | 7.41E-03 | 3.58E-13 | 4.24E-09 |
| **KANK4** | 1.14E-05 | 8.80E-04 | 6.80E-04 | 1.51E-06 | 8.35E-03 | 2.67E-02 | 6.93E-04 | 1.92E-02 | 2.04E-01 | 8.37E-04 | 4.06E-13 | 4.80E-09 |
| REPS1 | 1.91E-04 | 1.64E-03 | 4.80E-02 | 5.60E-04 | 4.39E-03 | 9.03E-02 | 9.49E-04 | 2.88E-03 | 9.36E-02 | 2.08E-02 | 4.40E-13 | 5.20E-09 |
| **MME** | 3.25E-07 | 2.13E-03 | 9.26E-07 | 1.67E-06 | 2.23E-06 | 1.00E-01 | 8.20E-07 | 8.60E-03 | 1.59E-01 | 1.32E-02 | 9.01E-13 | 1.07E-08 |
| **NR5A2** | 7.04E-03 | 6.20E-04 | 4.63E-04 | 1.20E-02 | 3.35E-03 | 2.44E-01 | 1.04E-04 | 2.48E-03 | 8.04E-05 | 2.47E-02 | 1.35E-12 | 1.60E-08 |
| MAOA | 6.85E-06 | 2.67E-03 | 1.21E-07 | 1.06E-02 | 1.23E-04 | 1.29E-01 | 2.01E-02 | 1.20E-03 | 9.95E-02 | 3.41E-02 | 1.47E-12 | 1.73E-08 |
| PITPNM3 | 4.55E-10 | 8.53E-04 | 1.76E-10 | 3.69E-06 | 5.58E-02 | 8.39E-02 | 5.88E-02 | 8.69E-04 | 1.02E-01 | 3.99E-02 | 6.04E-12 | 7.14E-08 |
| GCNT2 | 3.34E-03 | 1.93E-05 | 2.10E-04 | 1.01E-02 | 6.75E-03 | 1.89E-01 | 1.09E-02 | 6.84E-02 | 8.11E-04 | 6.50E-02 | 8.63E-12 | 1.02E-07 |
| DCAF6 | 8.26E-04 | 5.55E-03 | 1.30E-10 | 1.47E-03 | 3.11E-04 | 1.61E-01 | 4.14E-10 | 6.63E-03 | 1.29E-01 | 7.39E-02 | 1.66E-11 | 1.96E-07 |
| AKAP6 | 1.27E-03 | 3.18E-04 | 4.86E-02 | 1.41E-03 | 8.68E-02 | 1.48E-02 | 2.94E-03 | 1.22E-01 | 1.06E-01 | 2.66E-03 | 2.09E-11 | 2.47E-07 |
| CGN | 3.07E-02 | 1.11E-08 | 1.17E-02 | 4.51E-02 | 1.75E-03 | 1.15E-02 | 2.83E-05 | 8.34E-02 | 1.59E-01 | 5.69E-02 | 2.88E-11 | 3.41E-07 |
| DOK6 | 6.57E-06 | 1.25E-06 | 7.40E-13 | 1.88E-05 | 3.98E-02 | 3.08E-01 | 7.46E-03 | 2.56E-03 | 2.42E-03 | 4.21E-02 | 3.05E-11 | 3.61E-07 |
| EPHB1 | 3.31E-09 | 1.74E-03 | 3.44E-04 | 6.56E-03 | 2.17E-02 | 2.53E-03 | 1.29E-02 | 1.19E-01 | 2.18E-01 | 2.00E-02 | 3.16E-11 | 3.73E-07 |
| SLCO1A2 | 2.06E-01 | 2.68E-04 | 2.48E-08 | 4.51E-03 | 1.27E-02 | 2.11E-02 | 2.82E-02 | 3.63E-02 | 8.36E-02 | 1.34E-02 | 3.37E-11 | 3.99E-07 |
| EGFLAM | 2.17E-13 | 3.06E-04 | 2.15E-15 | 4.27E-05 | 4.26E-04 | 4.03E-01 | 1.49E-04 | 1.45E-03 | 4.35E-03 | 8.29E-03 | 4.45E-11 | 5.26E-07 |
| IL6R | 1.20E-03 | 1.09E-05 | 3.59E-07 | 2.50E-03 | 1.08E-05 | 6.61E-02 | 1.15E-02 | 1.83E-01 | 6.42E-02 | 1.03E-01 | 6.12E-11 | 7.23E-07 |
| **ABCA6** | 6.76E-03 | 4.15E-03 | 2.74E-04 | 1.66E-02 | 1.95E-04 | 5.86E-02 | 4.55E-04 | 1.56E-01 | 1.64E-01 | 2.58E-02 | 6.25E-11 | 7.39E-07 |
| CBS | 1.56E-01 | 1.14E-04 | 1.49E-02 | 2.66E-02 | 2.43E-04 | 3.75E-02 | 8.25E-02 | 4.03E-05 | 7.79E-02 | 5.25E-02 | 8.99E-11 | 1.06E-06 |
| FAM151A | 2.02E-05 | 1.27E-03 | 3.33E-05 | 7.67E-08 | 3.41E-07 | 2.18E-01 | 2.76E-04 | 1.86E-03 | 1.17E-02 | 2.17E-01 | 9.61E-11 | 1.14E-06 |
| CYP2C19 | 9.64E-05 | 2.55E-04 | 3.53E-17 | 5.19E-11 | 9.45E-09 | 4.23E-01 | 7.34E-07 | 1.70E-07 | 2.04E-02 | 1.02E-02 | 1.02E-10 | 1.20E-06 |
| ENPP3 | 4.87E-05 | 2.61E-02 | 1.65E-05 | 6.10E-10 | 7.13E-04 | 2.32E-01 | 1.52E-03 | 7.98E-02 | 1.10E-01 | 9.94E-03 | 1.18E-10 | 1.39E-06 |
| ACADSB | 3.51E-04 | 9.06E-02 | 2.07E-13 | 1.83E-02 | 6.55E-05 | 1.45E-02 | 4.31E-07 | 1.30E-02 | 3.20E-01 | 4.39E-03 | 1.19E-10 | 1.41E-06 |
| AASS | 3.85E-05 | 9.93E-03 | 9.34E-03 | 7.73E-02 | 2.20E-03 | 1.25E-01 | 4.54E-05 | 2.26E-01 | 8.44E-03 | 4.19E-03 | 1.22E-10 | 1.44E-06 |
| DNM1 | 4.51E-06 | 3.39E-02 | 4.26E-03 | 2.40E-03 | 4.27E-02 | 5.54E-03 | 4.17E-02 | 1.10E-01 | 5.13E-02 | 1.73E-01 | 1.29E-10 | 1.52E-06 |
| ACACB | 3.74E-03 | 5.12E-05 | 2.21E-03 | 3.05E-04 | 5.68E-05 | 7.37E-02 | 3.48E-04 | 7.78E-02 | 2.78E-01 | 5.58E-02 | 2.31E-10 | 2.72E-06 |
| RORA | 3.56E-03 | 1.70E-04 | 2.56E-03 | 8.85E-03 | 2.94E-01 | 1.20E-02 | 7.24E-06 | 4.25E-02 | 1.48E-04 | 1.49E-01 | 3.46E-10 | 4.09E-06 |
| NAGS | 1.80E-02 | 5.38E-04 | 3.65E-03 | 3.93E-03 | 1.27E-06 | 9.27E-02 | 7.78E-03 | 5.69E-05 | 3.55E-01 | 5.04E-02 | 4.97E-10 | 5.86E-06 |
| PHLPP1 | 6.08E-02 | 3.88E-05 | 2.79E-07 | 4.33E-03 | 2.53E-05 | 1.27E-01 | 1.25E-03 | 6.62E-03 | 1.21E-01 | 2.17E-01 | 5.65E-10 | 6.66E-06 |
| BHMT | 8.44E-03 | 1.69E-01 | 1.46E-02 | 6.01E-03 | 5.34E-02 | 1.25E-02 | 2.14E-03 | 3.65E-02 | 2.25E-01 | 1.16E-02 | 5.80E-10 | 6.84E-06 |
| MAT1A | 1.32E-02 | 2.31E-03 | 3.42E-12 | 1.94E-01 | 6.62E-07 | 2.32E-01 | 2.69E-05 | 3.01E-05 | 2.49E-02 | 7.78E-02 | 6.30E-10 | 7.43E-06 |
| SLC17A2 | 1.42E-01 | 4.54E-02 | 1.54E-10 | 8.91E-04 | 9.97E-04 | 1.10E-01 | 7.24E-02 | 6.50E-03 | 8.72E-02 | 8.13E-02 | 6.55E-10 | 7.73E-06 |
| OAT | 3.60E-03 | 3.64E-04 | 1.63E-11 | 1.29E-01 | 1.54E-02 | 2.50E-01 | 1.47E-06 | 2.05E-05 | 1.00E-01 | 5.54E-02 | 7.44E-10 | 8.78E-06 |
| SORD | 3.64E-04 | 3.46E-03 | 1.80E-06 | 5.17E-02 | 8.98E-06 | 1.28E-01 | 6.31E-06 | 3.50E-03 | 3.53E-01 | 2.87E-02 | 9.71E-10 | 1.14E-05 |
| DNAJC12 | 1.12E-02 | 1.08E-01 | 2.28E-14 | 4.39E-02 | 1.17E-03 | 1.65E-01 | 7.03E-06 | 3.74E-03 | 1.09E-02 | 2.30E-01 | 1.09E-09 | 1.29E-05 |
| ATP7B | 3.43E-02 | 1.67E-04 | 1.37E-02 | 5.38E-07 | 3.71E-03 | 2.02E-01 | 1.46E-03 | 1.68E-01 | 5.88E-02 | 9.95E-02 | 1.22E-09 | 1.44E-05 |
| ECHDC2 | 9.81E-04 | 2.67E-04 | 2.86E-01 | 3.77E-04 | 6.23E-02 | 4.36E-02 | 4.09E-02 | 1.70E-06 | 9.85E-02 | 5.80E-02 | 1.44E-09 | 1.70E-05 |
| COBLL1 | 4.95E-02 | 1.19E-02 | 4.41E-04 | 2.03E-08 | 4.45E-05 | 3.11E-01 | 2.85E-11 | 9.82E-03 | 1.82E-01 | 3.28E-02 | 1.59E-09 | 1.87E-05 |
| PEG3 | 6.03E-05 | 2.37E-05 | 3.66E-06 | 8.69E-02 | 5.95E-03 | 5.27E-02 | 1.42E-03 | 1.86E-02 | 5.04E-02 | 3.96E-01 | 2.04E-09 | 2.40E-05 |
| KBTBD11 | 4.40E-06 | 4.11E-02 | 1.30E-08 | 2.62E-01 | 4.60E-05 | 1.43E-01 | 1.39E-01 | 3.56E-03 | 2.41E-04 | 2.92E-02 | 2.22E-09 | 2.62E-05 |
| ABAT | 4.64E-02 | 7.21E-03 | 2.39E-07 | 1.48E-01 | 2.05E-06 | 1.22E-01 | 2.10E-04 | 2.45E-01 | 4.03E-02 | 9.74E-03 | 2.26E-09 | 2.66E-05 |
| MT1X | 8.35E-03 | 1.77E-01 | 2.54E-03 | 1.03E-01 | 3.47E-04 | 1.80E-01 | 3.25E-06 | 2.43E-03 | 3.81E-03 | 1.48E-01 | 2.57E-09 | 3.03E-05 |
| SOX5 | 5.20E-02 | 5.49E-04 | 1.76E-02 | 2.39E-05 | 1.02E-01 | 2.06E-01 | 2.11E-02 | 2.08E-01 | 1.60E-02 | 8.98E-03 | 2.79E-09 | 3.29E-05 |
| SLCO1B3 | 9.24E-02 | 1.01E-04 | 2.28E-03 | 5.05E-04 | 3.71E-04 | 3.73E-01 | 3.31E-02 | 1.10E-02 | 9.95E-02 | 2.05E-02 | 2.84E-09 | 3.35E-05 |
| BSN | 1.56E-05 | 5.53E-04 | 1.01E-01 | 6.39E-04 | 4.19E-02 | 1.38E-01 | 1.91E-02 | 6.40E-02 | 5.72E-02 | 2.15E-01 | 3.08E-09 | 3.63E-05 |
| SLC47A1 | 3.40E-06 | 6.34E-05 | 2.29E-08 | 8.43E-04 | 2.01E-06 | 3.70E-01 | 6.64E-03 | 2.04E-03 | 2.37E-01 | 2.33E-02 | 3.21E-09 | 3.78E-05 |
| TCEA3 | 3.95E-02 | 1.17E-04 | 6.76E-08 | 6.89E-02 | 4.70E-03 | 1.44E-01 | 1.40E-03 | 1.69E-03 | 1.06E-01 | 2.85E-01 | 3.72E-09 | 4.38E-05 |
| CUX2 | 5.38E-02 | 1.95E-03 | 2.62E-03 | 2.25E-01 | 1.95E-04 | 4.20E-03 | 1.70E-03 | 3.22E-02 | 6.36E-04 | 3.49E-01 | 5.16E-09 | 6.08E-05 |
| MTHFD1 | 1.05E-02 | 3.49E-03 | 2.52E-04 | 5.46E-02 | 3.97E-04 | 1.79E-01 | 3.41E-06 | 1.64E-02 | 2.32E-01 | 1.78E-01 | 5.39E-09 | 6.35E-05 |
| RND2 | 2.34E-05 | 1.04E-02 | 6.75E-10 | 9.89E-02 | 1.44E-04 | 1.43E-01 | 4.65E-02 | 2.08E-04 | 2.63E-01 | 1.16E-01 | 5.68E-09 | 6.69E-05 |
| SULT1E1 | 1.56E-03 | 3.40E-02 | 2.44E-05 | 4.44E-06 | 1.28E-02 | 3.14E-03 | 1.45E-06 | 2.05E-01 | 1.79E-01 | 2.44E-01 | 5.84E-09 | 6.88E-05 |
| LYVE1 | 8.24E-04 | 1.31E-02 | 2.86E-07 | 3.21E-01 | 2.03E-02 | 2.10E-01 | 3.57E-08 | 3.38E-03 | 1.10E-01 | 2.66E-03 | 5.89E-09 | 6.93E-05 |
| RAPGEF2 | 7.68E-03 | 5.02E-05 | 5.41E-04 | 2.19E-01 | 6.76E-02 | 1.17E-01 | 6.99E-03 | 9.72E-02 | 3.78E-02 | 1.28E-01 | 5.99E-09 | 7.05E-05 |
| EPB41L5 | 3.95E-03 | 5.88E-03 | 1.74E-02 | 7.60E-03 | 2.97E-04 | 1.86E-01 | 2.21E-08 | 2.05E-02 | 3.07E-01 | 1.36E-01 | 6.26E-09 | 7.37E-05 |
| CACNA1H | 6.89E-04 | 7.15E-04 | 1.33E-05 | 1.92E-03 | 2.16E-04 | 8.92E-02 | 2.91E-03 | 3.35E-01 | 9.72E-02 | 1.63E-01 | 6.84E-09 | 8.06E-05 |
| ZHX3 | 5.18E-02 | 1.81E-03 | 1.18E-03 | 7.07E-04 | 9.94E-07 | 3.17E-02 | 3.82E-02 | 1.32E-02 | 1.26E-01 | 4.29E-01 | 7.07E-09 | 8.33E-05 |
| MT1F | 1.91E-02 | 2.15E-01 | 4.84E-04 | 2.14E-05 | 1.07E-02 | 4.23E-01 | 4.01E-06 | 1.15E-03 | 2.71E-02 | 1.46E-03 | 7.55E-09 | 8.89E-05 |
| HOMER2 | 3.78E-04 | 2.95E-09 | 1.30E-12 | 1.38E-03 | 8.54E-04 | 2.59E-01 | 3.55E-04 | 7.43E-05 | 6.07E-02 | 3.84E-01 | 8.62E-09 | 1.02E-04 |
| ABHD15 | 2.96E-02 | 1.33E-02 | 6.02E-09 | 1.05E-02 | 7.22E-07 | 3.34E-02 | 3.43E-02 | 1.54E-03 | 5.56E-01 | 2.95E-02 | 8.77E-09 | 1.03E-04 |
| ST3GAL6 | 3.57E-04 | 7.15E-03 | 5.52E-05 | 8.22E-04 | 2.63E-03 | 5.30E-01 | 7.67E-04 | 8.08E-03 | 2.22E-02 | 1.60E-01 | 1.23E-08 | 1.45E-04 |
| NEK10 | 6.40E-03 | 1.87E-02 | 1.63E-01 | 5.56E-05 | 1.97E-01 | 1.04E-01 | 5.75E-03 | 4.34E-02 | 7.44E-02 | 1.25E-01 | 1.32E-08 | 1.55E-04 |
| MT1H | 2.32E-02 | 3.01E-01 | 3.28E-03 | 9.19E-05 | 1.64E-01 | 1.73E-01 | 1.33E-05 | 2.08E-03 | 3.36E-02 | 3.80E-02 | 1.34E-08 | 1.57E-04 |
| RELN | 4.69E-02 | 1.49E-03 | 6.18E-04 | 6.43E-02 | 4.24E-02 | 3.75E-02 | 1.17E-04 | 2.76E-01 | 1.03E-01 | 1.82E-01 | 1.63E-08 | 1.92E-04 |
| CAPN5 | 2.22E-02 | 4.33E-04 | 1.31E-06 | 2.14E-01 | 2.95E-02 | 3.62E-02 | 1.70E-03 | 2.02E-03 | 3.99E-01 | 5.13E-02 | 1.67E-08 | 1.97E-04 |
| **ASPG** | 5.20E-02 | 4.77E-07 | 2.43E-04 | 7.10E-03 | 2.01E-06 | 5.93E-01 | 2.54E-02 | 2.63E-04 | 3.75E-02 | 4.93E-02 | 1.87E-08 | 2.21E-04 |
| A1CF | 1.94E-02 | 1.07E-02 | 1.67E-04 | 2.91E-01 | 9.37E-05 | 1.93E-02 | 8.11E-09 | 1.25E-01 | 3.30E-02 | 2.66E-01 | 1.88E-08 | 2.21E-04 |
| DRD1 | 8.36E-04 | 1.61E-06 | 1.32E-01 | 8.50E-10 | 1.59E-01 | 1.31E-01 | 4.72E-03 | 7.52E-02 | 2.16E-01 | 4.71E-02 | 1.89E-08 | 2.22E-04 |
| ASB9 | 1.63E-03 | 3.64E-03 | 1.80E-04 | 4.21E-03 | 4.35E-02 | 2.90E-01 | 6.58E-02 | 5.58E-03 | 2.42E-01 | 1.16E-01 | 2.08E-08 | 2.45E-04 |
| POU6F2 | 4.29E-02 | 2.49E-02 | 1.30E-03 | 2.76E-04 | 2.46E-01 | 1.73E-01 | 1.42E-02 | 1.33E-01 | 1.32E-01 | 7.48E-03 | 2.14E-08 | 2.52E-04 |
| SIRT1 | 2.95E-01 | 6.46E-03 | 2.90E-06 | 1.52E-01 | 4.49E-02 | 2.26E-01 | 2.43E-05 | 4.57E-02 | 1.37E-02 | 1.63E-03 | 2.44E-08 | 2.87E-04 |
| ABCC9 | 1.08E-05 | 8.17E-04 | 4.09E-05 | 2.57E-04 | 7.00E-03 | 3.92E-02 | 6.85E-09 | 1.23E-01 | 6.12E-01 | 9.49E-03 | 2.68E-08 | 3.15E-04 |
| MT1G | 1.64E-02 | 4.18E-01 | 1.46E-03 | 1.93E-03 | 5.23E-02 | 2.82E-01 | 2.54E-07 | 1.13E-03 | 1.38E-02 | 6.10E-03 | 2.70E-08 | 3.18E-04 |
| CPNE6 | 5.24E-06 | 2.05E-04 | 1.06E-08 | 2.84E-09 | 9.98E-02 | 2.06E-01 | 2.39E-01 | 9.18E-02 | 1.38E-01 | 2.13E-02 | 2.83E-08 | 3.33E-04 |
| NTN4 | 9.34E-02 | 3.07E-05 | 2.21E-10 | 9.20E-04 | 2.03E-01 | 1.50E-01 | 2.82E-06 | 1.49E-01 | 1.91E-01 | 1.05E-02 | 2.89E-08 | 3.39E-04 |
| ATP11C | 5.87E-03 | 1.85E-04 | 9.00E-04 | 2.35E-01 | 4.16E-02 | 1.65E-01 | 6.42E-04 | 8.66E-03 | 2.86E-01 | 5.68E-02 | 2.99E-08 | 3.51E-04 |
| KCNN2 | 6.19E-06 | 2.33E-02 | 1.68E-15 | 7.63E-06 | 5.93E-07 | 2.48E-01 | 1.50E-09 | 4.07E-05 | 7.61E-02 | 4.60E-01 | 3.25E-08 | 3.82E-04 |
| ADRA1A | 9.31E-04 | 1.28E-03 | 1.13E-01 | 1.91E-04 | 6.60E-04 | 2.79E-02 | 1.09E-04 | 6.21E-03 | 4.64E-01 | 2.02E-01 | 3.65E-08 | 4.29E-04 |
| PPP1R1A | 2.00E-03 | 3.43E-04 | 3.70E-14 | 4.82E-04 | 7.23E-05 | 2.91E-01 | 1.11E-07 | 2.86E-04 | 3.33E-02 | 5.03E-01 | 4.31E-08 | 5.06E-04 |
| COL6A6 | 5.28E-03 | 2.77E-01 | 1.95E-03 | 7.35E-04 | 2.95E-01 | 1.01E-01 | 1.19E-02 | 4.52E-02 | 7.29E-02 | 2.26E-02 | 4.50E-08 | 5.29E-04 |
| ADCY1 | 4.01E-02 | 2.98E-07 | 1.05E-13 | 6.82E-08 | 8.83E-05 | 1.48E-02 | 3.11E-03 | 6.86E-04 | 5.93E-01 | 1.93E-01 | 5.07E-08 | 5.96E-04 |
| NEU3 | 3.54E-01 | 4.65E-03 | 2.23E-02 | 3.73E-05 | 8.37E-02 | 1.18E-02 | 8.99E-03 | 1.59E-01 | 2.00E-01 | 1.06E-03 | 5.16E-08 | 6.07E-04 |
| ITCH | 4.43E-02 | 1.49E-03 | 2.61E-03 | 2.07E-01 | 3.32E-03 | 2.76E-01 | 5.29E-04 | 1.64E-01 | 1.57E-01 | 1.69E-03 | 6.05E-08 | 7.10E-04 |
| ABCA8 | 1.33E-01 | 6.14E-04 | 2.01E-02 | 9.97E-04 | 1.67E-02 | 1.68E-01 | 4.30E-03 | 2.96E-01 | 1.81E-01 | 4.54E-02 | 6.56E-08 | 7.71E-04 |
| CA14 | 3.06E-04 | 2.89E-02 | 9.29E-06 | 4.04E-03 | 2.63E-04 | 5.12E-01 | 6.25E-03 | 6.41E-02 | 3.26E-02 | 2.27E-01 | 7.30E-08 | 8.57E-04 |
| CADM2 | 3.15E-16 | 6.49E-08 | 1.24E-07 | 6.88E-06 | 5.23E-05 | 5.86E-01 | 2.54E-02 | 2.53E-01 | 8.03E-03 | 5.56E-03 | 7.55E-08 | 8.87E-04 |
| USH2A | 2.14E-04 | 6.33E-04 | 3.76E-09 | 1.88E-04 | 3.24E-03 | 2.14E-01 | 1.65E-03 | 6.17E-01 | 5.01E-02 | 3.81E-03 | 8.70E-08 | 1.02E-03 |
| ERBB3 | 1.26E-01 | 4.53E-06 | 6.83E-04 | 1.52E-03 | 3.31E-02 | 9.83E-02 | 7.19E-04 | 3.59E-01 | 2.70E-01 | 3.74E-03 | 8.89E-08 | 1.04E-03 |
| GNMT | 3.04E-01 | 6.08E-02 | 1.02E-07 | 1.13E-01 | 1.47E-02 | 2.80E-01 | 2.11E-04 | 5.98E-05 | 7.51E-02 | 5.15E-02 | 9.50E-08 | 1.12E-03 |
| CDC14B | 1.19E-02 | 1.56E-01 | 1.49E-03 | 1.12E-01 | 8.39E-04 | 7.96E-02 | 2.78E-04 | 4.10E-02 | 4.48E-01 | 4.95E-02 | 9.66E-08 | 1.13E-03 |
| PTPRD | 2.26E-02 | 1.64E-02 | 1.67E-08 | 3.97E-03 | 4.72E-01 | 1.79E-01 | 3.47E-03 | 1.89E-01 | 8.46E-04 | 1.64E-02 | 1.01E-07 | 1.18E-03 |
| SYT1 | 2.00E-09 | 5.87E-08 | 5.07E-16 | 3.49E-01 | 5.03E-02 | 2.08E-01 | 2.92E-01 | 1.01E-03 | 5.18E-03 | 1.02E-03 | 1.03E-07 | 1.20E-03 |
| HECTD1 | 3.97E-02 | 3.63E-08 | 7.65E-03 | 6.32E-03 | 5.29E-02 | 1.04E-01 | 3.47E-05 | 3.57E-01 | 2.77E-01 | 7.16E-02 | 1.15E-07 | 1.35E-03 |
| RMND5A | 1.74E-03 | 5.88E-04 | 4.91E-03 | 4.00E-01 | 6.05E-03 | 4.08E-02 | 7.77E-04 | 3.08E-02 | 1.26E-01 | 3.05E-01 | 1.16E-07 | 1.36E-03 |
| ST3GAL1 | 8.66E-03 | 1.10E-02 | 2.22E-06 | 5.34E-08 | 8.41E-09 | 3.57E-01 | 5.69E-03 | 2.23E-03 | 2.41E-01 | 3.01E-01 | 1.29E-07 | 1.51E-03 |
| CTNNA3 | 1.66E-04 | 5.05E-04 | 8.68E-07 | 4.35E-07 | 2.86E-02 | 7.18E-02 | 1.87E-03 | 8.70E-02 | 2.17E-01 | 5.25E-01 | 1.36E-07 | 1.59E-03 |
| CYP4F12 | 1.13E-01 | 9.63E-03 | 3.01E-01 | 2.28E-07 | 2.30E-02 | 1.19E-01 | 5.53E-05 | 2.54E-02 | 1.28E-01 | 2.13E-01 | 1.36E-07 | 1.60E-03 |
| MPDZ | 1.49E-02 | 1.57E-06 | 4.28E-04 | 5.61E-03 | 3.88E-05 | 6.81E-02 | 4.97E-02 | 2.90E-01 | 4.96E-01 | 1.81E-02 | 1.53E-07 | 1.80E-03 |
| MOCS1 | 1.41E-03 | 1.30E-02 | 1.10E-08 | 3.96E-01 | 2.24E-07 | 1.55E-01 | 3.39E-02 | 6.02E-02 | 5.63E-02 | 2.28E-01 | 1.55E-07 | 1.82E-03 |
| PPP4R4 | 4.34E-11 | 3.77E-04 | 1.15E-05 | 1.98E-01 | 5.76E-01 | 1.76E-02 | 4.84E-07 | 1.56E-01 | 2.46E-05 | 7.06E-03 | 1.74E-07 | 2.04E-03 |
| ALAD | 2.47E-01 | 1.09E-03 | 2.67E-05 | 7.75E-05 | 1.33E-03 | 3.45E-01 | 1.73E-02 | 3.99E-03 | 2.95E-01 | 4.55E-02 | 1.76E-07 | 2.06E-03 |
| ACOX2 | 2.12E-01 | 3.39E-02 | 1.28E-05 | 1.05E-07 | 8.36E-04 | 2.16E-01 | 5.98E-05 | 2.78E-07 | 2.60E-01 | 2.39E-01 | 1.88E-07 | 2.21E-03 |
| NEU4 | 3.04E-04 | 3.92E-03 | 3.64E-06 | 9.38E-05 | 4.87E-08 | 2.37E-01 | 4.22E-01 | 8.82E-04 | 2.62E-01 | 4.14E-02 | 1.98E-07 | 2.32E-03 |
| SLC25A13 | 5.05E-04 | 8.47E-03 | 6.15E-06 | 9.00E-02 | 8.73E-05 | 1.51E-01 | 4.52E-03 | 1.09E-03 | 6.89E-01 | 2.25E-02 | 1.98E-07 | 2.32E-03 |
| SEC14L2 | 2.89E-02 | 2.02E-03 | 1.63E-05 | 1.58E-04 | 4.77E-06 | 4.94E-02 | 1.88E-04 | 2.10E-04 | 7.80E-01 | 1.10E-01 | 2.05E-07 | 2.41E-03 |
| STX17 | 1.03E-03 | 3.46E-05 | 2.98E-04 | 1.36E-01 | 7.49E-03 | 5.80E-02 | 8.79E-05 | 1.40E-01 | 5.92E-01 | 4.08E-02 | 2.15E-07 | 2.53E-03 |
| HAO2 | 1.66E-06 | 7.15E-02 | 8.00E-07 | 7.30E-05 | 3.80E-05 | 2.39E-01 | 3.09E-03 | 3.30E-04 | 5.94E-01 | 6.70E-02 | 2.16E-07 | 2.53E-03 |
| ASB4 | 2.92E-04 | 8.18E-04 | 5.63E-04 | 1.67E-04 | 3.41E-02 | 4.70E-01 | 1.93E-01 | 1.42E-01 | 1.32E-01 | 3.76E-03 | 2.18E-07 | 2.56E-03 |
| FAM184A | 3.15E-01 | 7.18E-07 | 1.42E-01 | 3.71E-03 | 1.39E-04 | 1.32E-01 | 3.02E-03 | 2.98E-01 | 3.98E-02 | 4.59E-02 | 2.24E-07 | 2.62E-03 |
| HLX | 1.20E-01 | 1.37E-03 | 1.09E-02 | 1.45E-01 | 5.42E-04 | 1.69E-02 | 2.89E-01 | 8.90E-03 | 1.21E-01 | 2.73E-01 | 2.40E-07 | 2.81E-03 |
| STIM1 | 2.32E-02 | 6.28E-02 | 6.88E-03 | 2.04E-01 | 4.77E-03 | 1.42E-01 | 1.42E-04 | 1.76E-01 | 2.19E-01 | 1.50E-01 | 2.46E-07 | 2.88E-03 |
| BCHE | 2.24E-03 | 3.11E-02 | 1.21E-06 | 1.36E-03 | 2.11E-03 | 7.01E-01 | 3.50E-03 | 3.11E-04 | 2.49E-01 | 1.67E-03 | 2.57E-07 | 3.01E-03 |
| KLC4 | 3.25E-04 | 2.52E-04 | 8.42E-03 | 5.38E-03 | 1.91E-03 | 9.43E-02 | 3.29E-02 | 2.52E-02 | 3.11E-01 | 5.20E-01 | 2.76E-07 | 3.23E-03 |
| RIN3 | 3.98E-03 | 4.75E-02 | 4.47E-04 | 1.06E-01 | 1.78E-02 | 2.08E-02 | 4.05E-02 | 1.70E-01 | 2.14E-01 | 3.84E-01 | 2.88E-07 | 3.38E-03 |
| EPHX2 | 4.60E-03 | 4.19E-03 | 3.50E-05 | 3.39E-05 | 7.65E-05 | 1.03E-01 | 1.77E-04 | 2.54E-04 | 2.30E-01 | 6.62E-01 | 2.90E-07 | 3.39E-03 |
| PLAG1 | 9.36E-09 | 7.06E-06 | 5.39E-02 | 2.61E-02 | 1.76E-01 | 1.89E-01 | 1.48E-02 | 5.11E-01 | 3.55E-02 | 1.51E-03 | 2.99E-07 | 3.50E-03 |
| CES3 | 1.28E-02 | 2.78E-03 | 1.05E-02 | 5.35E-05 | 1.61E-06 | 2.79E-01 | 6.68E-03 | 2.12E-03 | 4.83E-01 | 2.12E-01 | 3.00E-07 | 3.51E-03 |
| NRIP1 | 2.51E-02 | 8.75E-04 | 8.10E-02 | 2.27E-01 | 1.18E-01 | 1.57E-01 | 1.59E-01 | 1.47E-01 | 6.18E-02 | 3.91E-02 | 3.27E-07 | 3.83E-03 |
| CYP2E1 | 5.28E-02 | 2.95E-01 | 5.28E-07 | 1.36E-01 | 1.81E-04 | 3.13E-01 | 3.77E-03 | 5.99E-02 | 1.06E-01 | 5.19E-02 | 3.29E-07 | 3.85E-03 |
| ALDH6A1 | 6.20E-02 | 3.20E-02 | 2.20E-05 | 5.28E-01 | 5.27E-05 | 7.35E-02 | 2.23E-05 | 5.48E-02 | 2.59E-01 | 1.29E-02 | 3.46E-07 | 4.05E-03 |
| CORO2A | 2.99E-02 | 5.20E-03 | 1.41E-02 | 3.88E-01 | 1.63E-05 | 5.54E-03 | 1.09E-01 | 1.75E-01 | 1.20E-01 | 1.79E-01 | 3.56E-07 | 4.16E-03 |
| COL25A1 | 4.05E-18 | 2.04E-04 | 3.52E-09 | 3.56E-03 | 3.07E-01 | 2.36E-01 | 7.21E-04 | 4.51E-01 | 2.27E-02 | 6.95E-03 | 3.63E-07 | 4.25E-03 |
| EXPH5 | 3.90E-02 | 1.42E-02 | 3.02E-04 | 2.03E-03 | 2.13E-03 | 5.42E-02 | 7.93E-03 | 7.77E-02 | 6.14E-01 | 2.25E-01 | 3.95E-07 | 4.63E-03 |
| L3MBTL4 | 5.30E-03 | 1.79E-02 | 1.53E-01 | 2.38E-03 | 1.36E-01 | 6.35E-01 | 1.55E-02 | 4.30E-02 | 7.58E-03 | 2.56E-02 | 4.11E-07 | 4.81E-03 |
| ZNF546 | 1.22E-01 | 2.14E-04 | 3.96E-04 | 1.17E-03 | 9.58E-02 | 1.68E-01 | 2.24E-04 | 4.34E-01 | 2.20E-01 | 6.90E-03 | 4.44E-07 | 5.20E-03 |
| PDE4DIP | 1.64E-03 | 5.60E-04 | 1.07E-02 | 6.65E-05 | 6.10E-05 | 3.42E-02 | 1.58E-03 | 1.99E-01 | 5.26E-01 | 2.80E-01 | 4.65E-07 | 5.44E-03 |
| NET1 | 3.41E-03 | 9.33E-03 | 2.44E-05 | 1.80E-04 | 4.45E-02 | 4.29E-01 | 1.45E-05 | 3.69E-02 | 5.30E-01 | 5.48E-03 | 4.84E-07 | 5.67E-03 |
| ATAD3C | 8.88E-02 | 2.06E-03 | 7.07E-05 | 2.43E-02 | 1.34E-03 | 3.45E-01 | 8.90E-02 | 3.00E-01 | 2.24E-02 | 1.88E-01 | 5.01E-07 | 5.86E-03 |
| FOLH1 | 1.75E-03 | 8.67E-04 | 2.01E-08 | 3.54E-03 | 3.28E-07 | 4.80E-01 | 2.48E-05 | 1.16E-02 | 5.45E-01 | 2.01E-02 | 5.08E-07 | 5.95E-03 |
| MIOS | 1.49E-02 | 6.48E-03 | 2.33E-03 | 1.89E-04 | 1.44E-02 | 2.92E-01 | 2.45E-03 | 7.08E-02 | 6.56E-01 | 1.32E-02 | 5.57E-07 | 6.52E-03 |
| MOGAT1 | 5.36E-03 | 1.38E-03 | 1.72E-02 | 6.53E-11 | 4.55E-02 | 1.07E-01 | 3.38E-01 | 4.17E-01 | 1.41E-01 | 2.30E-03 | 5.63E-07 | 6.59E-03 |
| HAP1 | 4.67E-16 | 1.15E-04 | 5.50E-06 | 7.09E-05 | 5.33E-02 | 2.94E-01 | 4.24E-02 | 2.47E-01 | 3.37E-01 | 1.05E-01 | 5.83E-07 | 6.82E-03 |
| SYT9 | 1.15E-04 | 1.91E-03 | 1.23E-01 | 1.06E-01 | 4.07E-04 | 1.94E-01 | 4.93E-03 | 6.35E-03 | 1.36E-01 | 5.05E-01 | 5.85E-07 | 6.84E-03 |
| TSHR | 7.16E-03 | 8.78E-03 | 9.50E-07 | 5.73E-06 | 2.38E-01 | 2.02E-01 | 1.60E-01 | 4.12E-01 | 7.09E-03 | 4.41E-02 | 5.90E-07 | 6.90E-03 |
| MED13 | 2.01E-01 | 1.71E-05 | 8.11E-03 | 1.75E-05 | 5.44E-02 | 1.01E-01 | 2.08E-02 | 5.93E-01 | 8.78E-02 | 2.01E-02 | 6.26E-07 | 7.32E-03 |
| SPAG9 | 4.52E-02 | 2.25E-08 | 5.53E-04 | 3.13E-02 | 1.81E-01 | 1.90E-01 | 5.48E-06 | 2.58E-02 | 5.98E-01 | 1.60E-02 | 6.35E-07 | 7.43E-03 |
| PPM1E | 2.52E-05 | 1.20E-02 | 4.05E-04 | 1.92E-07 | 3.24E-02 | 4.66E-01 | 3.12E-01 | 1.12E-01 | 1.53E-01 | 3.57E-03 | 6.64E-07 | 7.77E-03 |
| SLC31A1 | 3.06E-03 | 7.40E-03 | 1.07E-03 | 1.73E-01 | 4.82E-03 | 5.90E-02 | 1.23E-03 | 3.60E-02 | 6.95E-01 | 1.14E-01 | 6.77E-07 | 7.92E-03 |
| LBX2 | 1.15E-01 | 1.08E-01 | 4.57E-02 | 2.12E-02 | 1.31E-03 | 3.96E-01 | 2.97E-01 | 8.74E-03 | 6.43E-02 | 3.87E-02 | 6.88E-07 | 8.05E-03 |
| IHH | 1.00E-01 | 9.77E-04 | 7.76E-03 | 2.02E-01 | 2.35E-03 | 1.81E-03 | 3.75E-01 | 7.58E-03 | 3.83E-01 | 1.86E-02 | 7.13E-07 | 8.34E-03 |
| ANKRD35 | 1.00E-09 | 4.14E-03 | 3.27E-06 | 3.15E-02 | 4.39E-02 | 6.28E-01 | 1.58E-03 | 3.10E-04 | 3.30E-02 | 3.59E-01 | 7.29E-07 | 8.52E-03 |
| GLUD1 | 3.67E-03 | 1.13E-03 | 6.91E-07 | 2.47E-01 | 1.82E-04 | 2.60E-01 | 1.13E-03 | 2.88E-01 | 2.02E-01 | 1.08E-01 | 7.91E-07 | 9.24E-03 |
| PDK4 | 1.38E-03 | 4.00E-04 | 1.07E-04 | 3.74E-02 | 6.92E-03 | 6.14E-01 | 1.09E-03 | 1.30E-02 | 2.79E-01 | 1.58E-01 | 7.97E-07 | 9.32E-03 |
| KCNMA1 | 2.07E-01 | 8.04E-02 | 3.86E-03 | 3.01E-04 | 4.18E-02 | 3.46E-02 | 4.47E-03 | 3.18E-03 | 1.93E-02 | 7.22E-01 | 8.32E-07 | 9.73E-03 |
| ETNK2 | 1.49E-03 | 1.94E-02 | 6.90E-07 | 1.09E-02 | 5.90E-05 | 3.14E-02 | 1.02E-01 | 2.10E-04 | 5.78E-01 | 3.73E-01 | 8.36E-07 | 9.78E-03 |
| AKR1D1 | 3.36E-05 | 2.46E-02 | 6.45E-11 | 1.98E-04 | 1.67E-06 | 7.39E-01 | 6.45E-06 | 5.47E-03 | 2.88E-01 | 6.06E-02 | 8.38E-07 | 9.80E-03 |
| ING1 | 7.93E-05 | 4.35E-04 | 1.06E-07 | 6.14E-01 | 2.76E-03 | 3.11E-01 | 4.45E-02 | 7.77E-03 | 1.30E-01 | 9.70E-03 | 8.63E-07 | 1.01E-02 |
| LIPC | 7.42E-03 | 1.36E-01 | 1.20E-08 | 9.61E-02 | 5.15E-05 | 6.16E-01 | 1.67E-02 | 3.44E-04 | 5.65E-03 | 2.46E-01 | 8.83E-07 | 1.03E-02 |
| F11 | 1.11E-01 | 2.64E-03 | 2.91E-02 | 5.97E-08 | 1.54E-01 | 3.02E-02 | 1.11E-07 | 6.22E-03 | 7.30E-01 | 6.11E-02 | 8.95E-07 | 1.05E-02 |
| DCXR | 1.21E-01 | 1.31E-01 | 1.82E-04 | 1.26E-01 | 4.48E-04 | 3.24E-01 | 2.61E-01 | 4.22E-06 | 1.15E-01 | 5.61E-02 | 9.68E-07 | 1.13E-02 |
| RALGAPA2 | 7.28E-03 | 5.42E-04 | 4.96E-02 | 4.72E-03 | 2.12E-02 | 6.45E-02 | 3.34E-03 | 5.91E-02 | 8.30E-01 | 9.53E-02 | 9.79E-07 | 1.14E-02 |
| CPLX1 | 2.11E-01 | 1.99E-03 | 1.82E-02 | 7.58E-02 | 5.62E-02 | 1.35E-02 | 3.43E-01 | 1.05E-02 | 2.37E-01 | 1.69E-01 | 9.86E-07 | 1.15E-02 |
| HLF | 1.01E-02 | 2.95E-03 | 9.99E-04 | 4.42E-01 | 1.10E-03 | 5.87E-01 | 1.61E-05 | 8.12E-03 | 7.93E-02 | 4.37E-03 | 9.87E-07 | 1.15E-02 |
| ADH6 | 7.93E-02 | 7.64E-02 | 1.95E-08 | 6.13E-03 | 4.98E-05 | 2.17E-01 | 9.26E-05 | 9.43E-04 | 7.23E-01 | 3.48E-02 | 9.98E-07 | 1.17E-02 |
| TFR2 | 3.01E-01 | 1.59E-02 | 1.97E-05 | 2.11E-01 | 1.52E-02 | 2.60E-01 | 3.80E-02 | 1.94E-02 | 2.15E-01 | 6.51E-02 | 1.03E-06 | 1.20E-02 |
| MYO16 | 8.94E-02 | 1.25E-03 | 4.41E-03 | 3.14E-02 | 2.10E-05 | 5.69E-02 | 1.56E-03 | 8.42E-02 | 1.49E-01 | 7.30E-01 | 1.09E-06 | 1.27E-02 |
| ZC3H12C | 9.60E-03 | 9.81E-08 | 1.77E-02 | 8.76E-02 | 3.44E-01 | 9.41E-02 | 1.51E-01 | 3.24E-01 | 1.08E-01 | 1.19E-02 | 1.09E-06 | 1.28E-02 |
| SIRT5 | 4.19E-02 | 7.66E-02 | 1.13E-02 | 2.09E-01 | 1.11E-01 | 3.25E-01 | 4.07E-06 | 1.76E-05 | 2.56E-01 | 1.17E-01 | 1.10E-06 | 1.28E-02 |
| MAMLD1 | 2.75E-02 | 9.52E-04 | 4.39E-04 | 2.28E-01 | 1.52E-02 | 2.21E-01 | 5.43E-01 | 1.65E-02 | 5.80E-02 | 4.28E-02 | 1.14E-06 | 1.33E-02 |
| MET | 2.21E-03 | 2.74E-02 | 9.72E-05 | 7.19E-01 | 3.15E-02 | 6.87E-02 | 8.49E-06 | 7.95E-02 | 2.15E-01 | 9.51E-03 | 1.14E-06 | 1.34E-02 |
| N4BP2L1 | 2.54E-03 | 2.77E-03 | 7.90E-03 | 8.96E-02 | 1.69E-01 | 6.78E-01 | 6.66E-03 | 6.75E-02 | 4.80E-03 | 1.25E-01 | 1.16E-06 | 1.35E-02 |
| CYP4A11 | 1.08E-01 | 5.01E-01 | 5.49E-05 | 6.29E-02 | 4.35E-03 | 9.66E-02 | 9.76E-06 | 9.40E-02 | 2.02E-01 | 8.69E-02 | 1.16E-06 | 1.36E-02 |
| ALDH7A1 | 2.12E-03 | 1.60E-01 | 4.55E-08 | 8.03E-03 | 4.33E-05 | 5.63E-01 | 3.61E-05 | 5.04E-05 | 4.24E-01 | 6.35E-03 | 1.25E-06 | 1.46E-02 |
| DIXDC1 | 7.74E-02 | 1.12E-04 | 5.09E-02 | 5.16E-03 | 3.59E-01 | 1.07E-01 | 2.56E-02 | 4.27E-02 | 4.83E-01 | 1.45E-02 | 1.27E-06 | 1.48E-02 |
| SARDH | 1.59E-01 | 2.79E-02 | 2.77E-07 | 7.84E-03 | 4.03E-04 | 2.38E-01 | 1.23E-01 | 6.04E-04 | 2.07E-01 | 4.02E-01 | 1.28E-06 | 1.49E-02 |
| MT1M | 2.25E-03 | 3.13E-01 | 2.53E-04 | 4.72E-01 | 8.54E-03 | 3.53E-01 | 6.48E-06 | 3.34E-04 | 1.36E-03 | 1.93E-02 | 1.32E-06 | 1.54E-02 |
| EFHD1 | 1.57E-02 | 1.81E-01 | 2.13E-06 | 1.63E-02 | 1.28E-02 | 2.99E-01 | 1.53E-03 | 2.24E-03 | 8.16E-03 | 6.33E-01 | 1.32E-06 | 1.54E-02 |
| RCL1 | 1.33E-08 | 1.28E-01 | 1.96E-06 | 3.48E-01 | 5.42E-03 | 5.16E-01 | 1.07E-04 | 4.12E-03 | 4.74E-04 | 1.72E-01 | 1.37E-06 | 1.59E-02 |
| HMGCS2 | 5.02E-04 | 2.74E-02 | 1.50E-06 | 1.50E-01 | 6.22E-03 | 3.35E-01 | 4.84E-05 | 1.28E-04 | 5.58E-01 | 9.76E-02 | 1.38E-06 | 1.61E-02 |
| ELMO1 | 5.28E-02 | 2.69E-04 | 9.43E-05 | 3.70E-03 | 1.05E-01 | 3.61E-01 | 1.63E-01 | 1.26E-01 | 3.15E-01 | 5.07E-02 | 1.42E-06 | 1.65E-02 |
| ACADL | 1.33E-03 | 1.05E-02 | 4.29E-01 | 1.25E-04 | 4.45E-03 | 1.95E-01 | 4.28E-02 | 4.77E-02 | 4.16E-01 | 3.21E-02 | 1.43E-06 | 1.67E-02 |
| MAP2K1 | 1.38E-04 | 2.45E-01 | 4.38E-09 | 2.87E-01 | 8.79E-03 | 2.64E-01 | 1.75E-05 | 1.21E-04 | 2.46E-01 | 1.40E-01 | 1.58E-06 | 1.84E-02 |
| GLYAT | 4.24E-02 | 3.79E-01 | 6.84E-08 | 3.17E-04 | 7.37E-05 | 1.75E-01 | 3.27E-06 | 1.55E-05 | 5.36E-01 | 6.26E-02 | 1.64E-06 | 1.91E-02 |
| PRPSAP1 | 6.63E-02 | 1.49E-01 | 7.60E-05 | 7.75E-04 | 7.12E-03 | 3.77E-01 | 1.40E-03 | 2.04E-05 | 2.39E-01 | 3.60E-01 | 1.72E-06 | 2.00E-02 |
| THSD4 | 9.95E-05 | 2.08E-03 | 1.29E-01 | 8.31E-02 | 5.54E-02 | 5.57E-02 | 1.16E-01 | 5.15E-01 | 1.85E-01 | 6.13E-02 | 1.75E-06 | 2.04E-02 |
| ERRFI1 | 8.88E-04 | 1.35E-01 | 1.04E-03 | 2.57E-01 | 1.68E-02 | 4.29E-01 | 1.51E-03 | 3.95E-02 | 2.58E-01 | 6.90E-02 | 1.82E-06 | 2.13E-02 |
| C6 | 3.01E-02 | 2.55E-01 | 8.67E-03 | 7.12E-02 | 1.52E-02 | 3.06E-01 | 1.83E-04 | 5.48E-02 | 3.90E-01 | 7.81E-02 | 1.84E-06 | 2.14E-02 |
| KLF13 | 2.70E-01 | 3.40E-02 | 3.42E-05 | 6.85E-04 | 1.69E-03 | 9.32E-02 | 8.85E-02 | 5.65E-04 | 3.35E-01 | 3.93E-01 | 1.96E-06 | 2.29E-02 |
| NUBPL | 1.44E-04 | 1.64E-02 | 4.70E-02 | 1.41E-01 | 4.65E-05 | 2.72E-01 | 1.77E-02 | 5.33E-01 | 1.71E-01 | 2.87E-02 | 2.12E-06 | 2.48E-02 |
| ABCC11 | 8.42E-11 | 5.29E-02 | 4.71E-02 | 3.67E-03 | 7.24E-02 | 7.89E-02 | 1.09E-01 | 3.42E-01 | 1.48E-02 | 5.09E-01 | 2.18E-06 | 2.54E-02 |
| TMCO6 | 6.74E-02 | 6.69E-03 | 1.76E-02 | 3.48E-03 | 2.01E-03 | 1.98E-01 | 1.50E-01 | 2.41E-01 | 1.29E-01 | 4.17E-01 | 2.23E-06 | 2.60E-02 |
| CLCN4 | 5.61E-07 | 8.30E-04 | 2.14E-02 | 2.63E-01 | 2.10E-03 | 1.68E-01 | 1.03E-01 | 1.02E-01 | 5.25E-01 | 4.76E-02 | 2.23E-06 | 2.60E-02 |
| DNMBP | 3.17E-04 | 1.03E-02 | 9.76E-02 | 3.93E-02 | 5.46E-02 | 3.19E-01 | 1.44E-02 | 4.29E-01 | 6.22E-02 | 2.06E-01 | 2.24E-06 | 2.61E-02 |
| KCNH7 | 5.83E-03 | 3.54E-02 | 3.54E-04 | 2.27E-03 | 1.12E-01 | 7.07E-02 | 1.79E-04 | 8.79E-02 | 9.04E-01 | 1.71E-02 | 2.29E-06 | 2.66E-02 |
| GLP2R | 1.77E-06 | 1.25E-02 | 6.97E-04 | 1.69E-02 | 1.19E-04 | 3.28E-01 | 7.95E-04 | 3.78E-01 | 1.94E-01 | 3.05E-01 | 2.30E-06 | 2.68E-02 |
| PFKFB1 | 2.23E-02 | 1.36E-03 | 1.77E-01 | 1.66E-03 | 2.23E-01 | 4.37E-02 | 1.36E-03 | 6.74E-04 | 8.73E-02 | 6.78E-01 | 2.31E-06 | 2.70E-02 |
| PRRG4 | 7.40E-03 | 2.59E-03 | 3.09E-06 | 1.21E-01 | 3.20E-02 | 6.48E-01 | 5.30E-03 | 1.77E-02 | 4.02E-01 | 3.56E-03 | 2.35E-06 | 2.74E-02 |
| RNF165 | 9.11E-01 | 1.29E-04 | 1.02E-03 | 1.22E-05 | 6.72E-05 | 1.03E-01 | 1.32E-01 | 2.46E-04 | 7.13E-02 | 2.31E-02 | 2.39E-06 | 2.78E-02 |
| WDR59 | 3.41E-01 | 8.05E-08 | 1.14E-01 | 1.31E-05 | 2.72E-03 | 1.51E-01 | 3.25E-01 | 1.15E-01 | 2.77E-03 | 1.91E-01 | 2.41E-06 | 2.81E-02 |
| MLANA | 5.24E-03 | 8.87E-02 | 7.98E-03 | 7.63E-04 | 3.96E-01 | 6.58E-02 | 5.11E-01 | 1.33E-01 | 2.18E-02 | 1.25E-02 | 2.43E-06 | 2.83E-02 |
| CNKSR3 | 1.00E-02 | 2.58E-03 | 1.83E-03 | 9.60E-02 | 7.83E-04 | 1.48E-01 | 2.37E-03 | 1.47E-02 | 7.28E-01 | 2.52E-01 | 2.68E-06 | 3.13E-02 |
| MTMR4 | 9.33E-02 | 7.41E-07 | 9.05E-03 | 2.94E-05 | 1.48E-02 | 3.92E-02 | 2.62E-02 | 8.08E-03 | 9.91E-01 | 7.80E-02 | 2.78E-06 | 3.23E-02 |
| TM6SF2 | 2.30E-02 | 8.38E-03 | 1.28E-01 | 2.18E-02 | 4.04E-03 | 1.29E-01 | 4.66E-02 | 2.37E-04 | 8.56E-01 | 4.55E-02 | 2.84E-06 | 3.30E-02 |
| TRIM24 | 3.38E-01 | 1.29E-02 | 7.85E-03 | 9.38E-05 | 6.92E-04 | 1.00E-01 | 5.77E-04 | 5.73E-01 | 2.04E-01 | 2.65E-02 | 2.87E-06 | 3.34E-02 |
| ABCA5 | 3.13E-04 | 1.67E-04 | 5.35E-02 | 9.78E-04 | 6.17E-01 | 7.12E-02 | 3.37E-04 | 1.67E-01 | 2.92E-01 | 6.32E-02 | 2.89E-06 | 3.36E-02 |
| ENOSF1 | 3.80E-02 | 3.53E-05 | 1.11E-01 | 4.89E-05 | 5.16E-01 | 4.90E-01 | 2.90E-02 | 9.03E-05 | 8.09E-02 | 2.44E-03 | 2.93E-06 | 3.41E-02 |
| TTPAL | 1.99E-04 | 1.85E-01 | 1.68E-04 | 7.10E-03 | 2.70E-03 | 1.58E-01 | 3.61E-02 | 2.04E-02 | 7.36E-01 | 1.23E-01 | 2.98E-06 | 3.47E-02 |
| NDST3 | 4.00E-01 | 1.40E-01 | 3.47E-04 | 2.90E-02 | 2.50E-01 | 1.08E-01 | 2.62E-03 | 1.24E-02 | 3.22E-01 | 6.27E-03 | 3.04E-06 | 3.54E-02 |
| HSD17B14 | 9.40E-03 | 2.88E-03 | 7.16E-09 | 6.96E-04 | 3.80E-03 | 2.90E-01 | 8.77E-04 | 1.48E-04 | 2.57E-03 | 9.74E-01 | 3.37E-06 | 3.92E-02 |
| CYP2A13 | 3.23E-04 | 9.19E-02 | 1.14E-01 | 5.93E-01 | 3.97E-02 | 9.11E-02 | 3.12E-04 | 2.27E-01 | 1.24E-01 | 2.88E-03 | 3.37E-06 | 3.92E-02 |
| RBL2 | 1.84E-01 | 2.35E-05 | 1.85E-03 | 4.30E-02 | 3.31E-02 | 1.20E-01 | 7.13E-04 | 1.00E-01 | 7.99E-01 | 3.31E-03 | 3.41E-06 | 3.96E-02 |
| GFRA1 | 8.70E-07 | 8.72E-04 | 1.77E-07 | 1.95E-03 | 1.42E-05 | 7.84E-02 | 8.24E-02 | 2.31E-01 | 8.53E-01 | 4.00E-02 | 3.46E-06 | 4.02E-02 |
| EGFR | 4.86E-02 | 4.06E-06 | 2.86E-03 | 5.16E-02 | 1.65E-03 | 5.27E-02 | 5.09E-07 | 3.74E-01 | 6.51E-01 | 1.06E-01 | 3.49E-06 | 4.06E-02 |
| OSBPL6 | 3.20E-02 | 1.52E-02 | 4.52E-07 | 1.17E-05 | 7.55E-04 | 5.93E-01 | 2.00E-03 | 2.51E-01 | 4.04E-01 | 9.69E-04 | 3.78E-06 | 4.39E-02 |
| ZYG11B | 3.20E-02 | 2.07E-04 | 1.24E-02 | 6.68E-01 | 1.10E-01 | 5.00E-02 | 2.48E-03 | 1.36E-01 | 2.53E-01 | 3.59E-02 | 3.80E-06 | 4.42E-02 |
| KLHDC10 | 1.39E-01 | 4.83E-07 | 3.69E-03 | 1.77E-04 | 1.89E-03 | 5.28E-02 | 3.86E-05 | 1.09E-01 | 9.71E-01 | 2.29E-02 | 3.80E-06 | 4.42E-02 |
| SMOC1 | 2.67E-03 | 5.81E-02 | 3.37E-02 | 4.39E-02 | 2.84E-04 | 7.29E-03 | 1.87E-06 | 9.04E-02 | 4.09E-01 | 6.56E-01 | 3.83E-06 | 4.45E-02 |
| KDR | 1.16E-01 | 1.43E-05 | 1.76E-06 | 2.13E-01 | 5.08E-01 | 7.72E-02 | 5.23E-05 | 1.42E-01 | 2.20E-01 | 2.52E-02 | 3.84E-06 | 4.47E-02 |
| PIK3R4 | 3.36E-02 | 1.26E-02 | 2.00E-03 | 4.13E-01 | 3.62E-02 | 4.22E-01 | 1.11E-04 | 2.22E-02 | 3.33E-01 | 3.80E-02 | 4.19E-06 | 4.87E-02 |
| IL17RC | 7.00E-04 | 1.69E-04 | 2.06E-04 | 4.07E-03 | 1.17E-06 | 9.13E-02 | 3.76E-02 | 3.93E-06 | 2.24E-01 | 9.57E-01 | 4.24E-06 | 4.93E-02 |

**Supplementary Table 2. Functions of Significantly Differentially Expressed Genes (DEGs).**

| **Upregulated Genes** | | | | | | |
| --- | --- | --- | --- | --- | --- | --- |
| **Gene**  **Name** | **Adj** **meta *P-*value** | **Functional overview** | **Association with liver fibrosis/MASLD** | **Driver of liver fibrosis/MASLD** | **Known association with other liver diseases** | **Top expressing major cell types** |
| CCL19 | 2.99E-22 | Facilitates Lymphocyte recirculation and homing | Positively correlated with fibrosis(4, 5) | Not observed | Not observed | Mesenchyme, MPs, Mast cells |
| DPT | 4.66E-18 | Accelerates collagen fibril formation (6) | Positively correlated with severe NAFLD/fibrosis(7, 8) | Not observed | Not observed | Mesenchyme, Hepatocytes, Cholangiocytes |
| SOX4 | 1.32E-17 | Binds with high affinity to the T-cell enhancer | Increased in patients with NASH-fibrosis, activated HSCs(9) | Not observed | Increased in LSECs in fibrotic mouse models (10) | Cholangiocytes, pDCs, Mast cells |
| IGFBP7 | 1.51E-17 | Stimulates cell adhesion | Positively correlated with NAFLD severity/fibrosis (RNA(5, 8), Serum(11)) | Loss of IGFBP7 limited NAFLD in Zebrafish and mouse models(12, 13) and limited HSC activation(14) | Not observed | Mesenchyme, Endothelia, Mesothelia |
| AQP1 | 2.18E-16 | Membrane protein that facilitates movement of water | Positively associated with hepatic arterial capillary cell proliferation(15), severe NAFLD (8) | Not observed | Positively associated with invasion/migration of HCC cells *in vitro*(16), HBV-associated fibrosis(17), Biliary fibrosis(18). Negatively associated with HCC progression(19) | Cholangiocytes, Endothelia, Mesothelia |
| GRAMD1B | 2.07E-14 | Maintains cholesterol levels(20) | Not observed | Not observed | Positively associated in mouse HFD models and patients with MASLD(21), deletion limited NASH progression(22) | pDCs, Cholangiocytes, Hepatocytes |
| CAPN2 | 3.23E-14 | Protease involved in fibrosis resolution | Not observed | Not observed | Positively associated with HBV-fibrosis(23), HCC progression(24) | Cholangiocytes, Endothelia, Mesenchyme |
| LOXL4 | 4.71E-14 | Modulate the formation of a collagenous extracellular matrix | Increased in patients with steatohepatitis(25) | Not observed | Increased in HCC and associated with poor prognosis(26, 27), Wilsons Disease and PBC(28) | Cholangiocytes, Hepatocytes, Mesothelia |
| SOX9 | 6.30E-14 | Transcription factor, involved in scar formation | Positively correlated with fibrosis(4, 7), NASH/Fibrosis(9, 29); HSC activation(9), Severe NAFLD(8, 30) | Promoted liver fibrosis through transcription of ECM proteins(31), HSC activation(32, 33) and hepatocyte death(34) | HCV-fibrosis(35), Biliary Atresia(36) | Cholangiocytes, Hepatocytes, Mesenchyme |
| CCL21 | 7.56E-14 | Initiates adaptive and innate immune responses; recruit leukocytes | Positively correlated with fibrosis(4, 5) | Not observed | Increased in chronic Hepatitis C(37) and primary sclerosing cholangitis (PSC)(38) | Endothelia, Mesenchyme, Hepatocytes |
| IL32 | 1.23E-13 | Induces production of inflammatory cytokines(39) | Positively correlated with NAS score, liver fibrosis stage(5, 40-42) | Recombinant IL32 promoted insulin resistance(43). Targeting IL32 reduced collagen I expression in organoids(44) | Positively associated with HCC, liver fibrosis, and chronic HBV(45, 46) | T cells, ILCs, Hepatocytes |
| RASL11B | 3.38E-13 | Small GTPase, predicted to regulate SOX9 and TGFBR1 | Not observed | Not observed | Not observed | Mesenchyme, Cholangiocytes, Hepatocytes |
| CCL20 | 3.74E-13 | Chemotactic factor that attracts lymphocytes, neutrophils, and dendritic cells towards sites of injury | Positively correlated with fibrosis stage in NAFLD patients, produced by HSCs(42, 47-49) | Neutralizing antibodies limited HSC activation *in vitro* (50) | Positively associated with HBV-Fibrosis(51) | T cells, Cholangiocytes, Hepatocytes |
| DCDC2 | 9.70E-13 | Binds tubulin and regulates microtubule polymerization, primary cilia signaling | Negatively correlated with fibrosis (underlying cause not reported)(52) | Not observed | Pathogenic variants associated with neonatal sclerosing cholangitis, portal hypertension, cholestatic liver disease(53-55) | Cholangiocytes, Hepatocytes, Mesenchyme |
| THBS2 | 1.80E-12 | Mediates cell-cell and cell-matrix adhesions | Positively correlated with NASH-fibrosis(8, 56-62), Serum(11, 56) | Drives HSC activation and fibrosis in mice(57, 63) | Serum levels positively correlated with fibrosis stage in HCV patients(64) | Mesothelia, Mesenchyme, Endothelia |
| BACE2 | 2.21E-12 | Proteolytic processing of the amyloid precursor protein (APP) | Positively correlated with MASH-activity Score(65) | Not observed | Not observed | Mast cells, Cholangiocytes, Endothelia |
| FBLN5 | 5.32E-12 | Organization of elastic fibers, promotes adhesion of endothelial cells to extracellular matrix | Positively correlated with NASH-fibrosis(58) (49, 66) | Not observed | HBV-HCV fibrosis(67) | Mesenchyme, T cells, Endothelia |
| ADAMTSL2 | 7.37E-12 | Secreted glycoprotein, binds cell surface and extracellular matrix; interacts with latent TGFβ1 | Protein levels correlated with fibrosis stage in MASLD(68) | Not observed | Not observed | Mesenchyme, Endothelia, Hepatocytes |
| CXCL6 | 9.41E-12 | Chemotactic for neutrophils and granulocytes, involved in angiogenesis | Positively correlated with severe NASH and fibrosis(4, 8, 69, 70) | Not observed | Increased in hepatic progenitor cells in patients with PSC(71), biliary atresia(72) | Cholangiocytes, Mesothelia, Hepatocytes |
| SH3YL1 | 1.10E-11 | Androgen receptor, associated with generation of reactive oxygen species | Increased in patients with MASH(73) | Not observed | Not observed | Cholangiocytes, Hepatocytes, T cells |
| LUM | 1.11E-11 | Regulate collagen fibril organization | Levels positively correlated with severe NAFLD/ fibrosis(8, 59-61, 66, 74) | Not observed | Increased circulating levels associated with HCC(75) | Mesenchyme, Mesothelia, Hepatocytes |
| PODN | 1.75E-11 | Binds type 1 collagen | Not observed | Not observed | Associated with HBV-related HCC(76) | Mesothelia, Mesenchyme, Endothelia |
| CCDC146 | 1.93E-11 | Centrosome protein, found in microtubule organizing center | Not observed | Not observed | Tumor specific antigen increased in HCC(77) | Mesenchyme, ILCs, Endothelia |
| TMED3 | 2.17E-11 | Involved in cell cycle and antigen processing presenting pathways | Not observed | Not observed | Increased in patients with HCC, associated with metastasis(78) | Mesothelia, pDCs, Endothelia |
| CCND2 | 2.87E-11 | Essential for the control of the cell cycle at the G1/S transition | Positively correlated with liver fibrosis(79) | Not observed | Increased promoter methylation and reduced expression associated with HCC progression(80) | ILCs, T cells, Mesothelia |
| PRR15L | 5.11E-11 | No clear evidence. | Not observed | Not observed | Not observed | Cholangiocytes, Hepatocytes, Mesothelia |
| SUSD2 | 7.79E-11 | Negatively regulates cell cycle | Not observed | Not observed | Reduced expression in HCC, loss may promote metastasis(81) | Mesenchyme, pDCs, Mesothelia |
| RAB25 | 8.51E-11 | Small GTPase involved in membrane trafficking | Not observed | Not observed | Altered expression in HCC(82) (83) | Cholangiocytes, Hepatocytes, Mesenchyme |
| C7 | 1.15E-10 | Component of the complement complex, involved in host immunity and bacterial pathogenesis | Expression positively correlated with severe NAFLD(8), Increased in serum from patients with NASH(84) | Not observed | Protein levels in tissue or extracellular vesicles correlated with HCC risk(85) or cirrhosis(86). Gene variants associated with reduced fibrosis, increased HCC in HCV patients(87) | Mesenchyme, Endothelia, Hepatocytes |
| CD58 | 1.37E-10 | Immunoglobulin, facilitates T lymphocyte adhesion and activation | Not observed | Not observed | Associated with HCC in HBV and HCV patients(88) | Endothelia, MPs, Mast cells |
| **Downregulated genes** | | | | | | |
| **Gene Name** | **Adj** **meta *P*-value** | **Functional overview** | **Association with liver fibrosis/NAFLD** | **Driver of liver fibrosis/NAFLD** | **Known association with other liver Disease** | **Top 3 expressing major cell types** |
| SLC16A10 | 1.97E-12 | Aromatic amino acid transporter (MCT10) | Not observed | Not observed | Not observed | MPs, Hepatocytes, Cholangiocytes |
| ADCY10 | 1.38E-11 | Soluble Adenylyl cyclase, catalyzes ATP to cAMP | Not observed | Not observed | Altered in mouse HFD models(22) | Hepatocytes, Cholangiocytes, Endothelia |
| CNDP1 | 2.38E-11 | Zn2+ dependent Matrix Metalloprotease | Activity negatively associated with cirrhosis(89) | Not observed | Predicted microvascular invasion in HCC(90) | Hepatocytes, T cells, ILCs |
| COL28A1 | 4.72E-10 | Fibrillar collagen | Not observed | Not observed | Not observed | Mesenchyme, Cholangiocytes, Plasma B cells |
| CYP4F3 | 5.02E-10 | Cytochrome p450 family member, involved in fatty acid metabolism | Not observed | Not observed | Not observed | Hepatocytes, Mast cells, Cholangiocytes |
| NCAM2 | 8.54E-10 | Immunoglobulin, regulates neuronal differentiation(91) | Not observed | Not observed | Positively associated with HCC(92) | Mesothelia, Mesenchyme, Endothelia |
| CNKSR2 | 1.08E-09 | Scaffolding protein, regulates Ras signaling; couples signal transduction to membrane/cytoskeletal remodeling | Not observed | Not observed | Not observed | Mast cells, B cells, Cholangiocytes |
| AADAT | 1.47E-09 | Transaminase, acts on aminoadipate, kynurenine, methionine, glutamate, tryptophan, aspartate and hydroxykynurenine(93) (94) | Not observed | Not observed | Not observed | Mast cells, Hepatocytes, Mesenchyme |
| LARP1B | 3.11E-09 | RNA binding protein, facilitates pre-RNA maturation | Not observed | Not observed | Not observed | Hepatocytes, Plasma B cells, Cholangiocytes |
| SLC13A5 | 3.89E-09 | Sodium-dependent citrate transporter | Not observed | Not observed | Associated with Steatosis(95) (96). Depletion *in vivo* limits HCC cell proliferation(97) | Hepatocytes, Endothelia, Cholangiocytes |
| RAPGEFL1 | 3.93E-09 | Ras signal transduction, binds MHL1 and affects cytoskeleton(98) | Not observed | Not observed | Not observed | Mesothelia, pDCs, Endothelia |
| SH3PXD2A | 3.98E-09 | Adaptor protein, facilitates formation of invadopodia and podosomes, matrix degradation | Not observed | Not observed | Increased in exosomes in patients with HCC(99) | Mesenchyme, Mesothelia, Endothelia |
| HORMAD2 | 4.24E-09 | Involved in meiosis | Negatively correlated with fibrosis progression in NAFLD(100) | Not observed | Not observed | ILCs, T cells, MPs |
| KANK4 | 4.80E-09 | Negatively regulates actin polymerization | Not observed | Not observed | Not observed | Mesenchyme, Endothelia, MPs |
| REPS1 | 5.20E-09 | Adapter protein implicated in regulating EGF receptors and Ral-GTPases | Not observed | Not observed | Not observed | pDCs, ILCs, MPs |
| MME | 1.07E-08 | Membrane metallo-endopeptidase with thermolysin-like specificity | Not observed | Not observed | Not observed | pDCs, Endothelia, Hepatocytes |
| NR5A2 | 1.60E-08 | Orphan nuclear receptor, regulates gene transcription | Negative trend in NASH(101) | Loss of NR5A2 in mice worsened steatosis, inflammation, and fibrosis in some models of NAFLD(101, 102), reduced injury in others(103) | Biomarker of HCC(104), Exacerbated NAFLD in animal models(104), Prevented toxicant-induced liver injury(105) | Cholangiocytes, Hepatocytes, Endothelia |
| MAOA | 1.73E-08 | Deaminase involved in metabolism of neurotransmitters | Not observed | Not observed | Downregulated in HCC patients(106), Liver damage in obese mice(107), hepatic encephalopathy(108, 109) | Hepatocytes, Cholangiocytes, Endothelia |
| PITPNM3 | 7.14E-08 | Membrane associated phosphatidylinositol transferase | Hypermethylation of gene positively associated with Fatty Liver Index(110) | Not observed | Related to prognosis of HCC(111) | Endothelia, Cholangiocytes, Hepatocytes |
| GCNT2 | 1.02E-07 | beta-1,6-N-acetylglucos-aminyltransferase | Not observed | Not observed | Not observed | Endothelia, B cells, Hepatocytes |

The top 30 upregulated and 20 downregulated DEGs were selected based on adjusted *P*-values from the meta-analysis. Their functions and relevant studies and the top three associated cell types were included.

**Supplementary Table 3. Confirmation of top DEGs identified in our meta-analysis using an independent cohort**

| **Gene Name** | **Meta-analysis finding** | **log2 Fold Change** | ***P*-value** | **Adjusted *P*-value** |
| --- | --- | --- | --- | --- |
| **SH3YL1** | **Upregulated** | **0.62** | **3.26E-04** | **7.74E-03** |
| **CAPN2** | **Upregulated** | **0.55** | **2.43E-04** | **7.74E-03** |
| **PODN** | **Upregulated** | **1.12** | **3.94E-04** | **7.74E-03** |
| **IGFBP7** | **Upregulated** | **0.75** | **8.69E-04** | **1.28E-02** |
| **LUM** | **Upregulated** | **1.02** | **1.61E-03** | **1.90E-02** |
| **FBLN5** | **Upregulated** | **0.95** | **2.32E-03** | **2.28E-02** |
| **ADAMTSL2** | **Upregulated** | **0.59** | **3.11E-03** | **2.63E-02** |
| CXCL6 | Upregulated | 1.20 | 7.43E-03 | 5.08E-02 |
| BACE2 | Upregulated | 0.61 | 7.74E-03 | 5.08E-02 |
| LOXL4 | Upregulated | 0.83 | 1.54E-02 | 9.05E-02 |
| THBS2 | Upregulated | 0.72 | 1.69E-02 | 9.05E-02 |
| CGN | Downregulated | -0.35 | 2.71E-02 | 1.33E-01 |
| IL32 | Upregulated | 0.83 | 3.59E-02 | 1.36E-01 |
| SOX9 | Upregulated | 0.64 | 3.04E-02 | 1.36E-01 |
| DPT | Upregulated | 0.88 | 3.59E-02 | 1.36E-01 |
| COL28A1 | Downregulated | -0.99 | 3.69E-02 | 1.36E-01 |
| SOX4 | Upregulated | 0.51 | 4.34E-02 | 1.39E-01 |
| RASL11B | Upregulated | 1.04 | 4.71E-02 | 1.39E-01 |
| DCDC2 | Upregulated | 0.66 | 4.96E-02 | 1.39E-01 |
| NCAM2 | Downregulated | -0.76 | 4.10E-02 | 1.39E-01 |
| CYP4F3 | Downregulated | -0.21 | 4.76E-02 | 1.39E-01 |

**Supplemental Table 4. DEGs identified by our meta-analysis predicted to be secreted into the bloodstream based on the Human Protein Atlas (**[**https://www.proteinatlas.org/humanproteome/tissue/secretome**](https://www.proteinatlas.org/humanproteome/tissue/secretome)**) (112)**

| **DEGs Predicted to be Secreted** | | | | | |
| --- | --- | --- | --- | --- | --- |
| Upregulated DEGs | | | | | Downregulated DEGs |
| CCL19 | BACE2 | MFAP4 | FMOD | CCDC3 | CNDP1 |
| DPT | FBLN5 | Col3A1 | SERPINE2 | MGP | Col28A1 |
| IGFBP7 | ADAMTSL2 | LAMC3 | TIMP1 | SERPINH1 | EGFLAM |
| LOXL4 | CXCL6 | ENPP2 | ASPN | NTS | IL6R |
| CCL21 | LUM | WNT4 | PDGFA | CTSK | CYP2C19 |
| IL32 | PODN | AEBP1 | ANXA2 | EMCN | ENPP3 |
| CCL20 | C7 | LTBP2 | EFEMP2 | Col1A2 | ACACB |
| THBS2 | ITGBL1 | FAP | SFRP5 | FGFR2 | NAGS |

|  | **F4 vs F0**  **(Govaere et al, PMID 33268509)** | **F2-4 vs F0-1**  **(Sanyal et al., PMID 36528237)** |
| --- | --- | --- |
| **Upregulated** | IGFBP7, THBS2, ADAMTSL2, MFAP4, SORD, AKR1D1, BGN, DCXR, AKR1B10, CHI3L1, CHST9, GDF15, GOLM1, IGSF3, LAMC2, LRRC32, LY75, MSH2, NFASC, SHBG, SVEP1, TREM2, VWF | ADAMTSL2, C7, NFSAC |
| **Downregulated** | AKR1D1, DCXR, SORD | KDR |

**Supplementary Table 5. Genes from our study identified as serum markers associated with MASLD fibrosis.**

**Supplementary Table 6: Cell-type specific markers used for deconvolution analysis**

| **MP** | **pDC** | **ILC** | **T Cell** | **B Cell** | **Plasma B cell** | **Mast Cell** | **Endothelia** | **Mesenchyme** | **Mesothelia** | **Hepatocyte** | **Cholangiocyte** |
| --- | --- | --- | --- | --- | --- | --- | --- | --- | --- | --- | --- |
| LYZ | IRF7 | GNLY | TRAC | MS4A1 | IGLC2 | TPSB2 | FCN3 | TAGLN | PLA2G2A | APOA1 | FXYD2 |
| C1QA | PLD4 | CCL3 | CD3D | CD79B | IGKC | TPSAB1 | IFI27 | ACTA2 | CCDC80 | APOC1 | ANXA4 |
| C1QB | LILRA4 | NKG7 | IL7R | CD37 | IGHA1 | CTSG | DNASE1L3 | MYL9 | ITLN1 | ORM1 | TM4SF4 |
| S100A9 | IRF8 | XCL1 | CD3E | VPREB3 | IGHG1 | CPA3 | GNG11 | RGS5 | PRG4 | TTR | KRT7 |
| S100A8 | CCDC50 | KLRF1 | TRBC2 | IGHD | IGHG3 | TPSD1 | ID1 | ADIRF | UPK3B | FGB | SPP1 |
| CST3 | PPP1R14B | CMC1 | CD2 | LINC00926 | IGHA2 | MS4A2 | RAMP2 | CALD1 | PTGIS | FGG | CLDN10 |
| C1QC | SERPINF1 | CD7 | IL32 | BANK1 | IGHGP | VWA5A | MGP | BGN | C1R | APOH | CD24 |
| FTL | RNASE6 | XCL2 | CD3G | HVCN1 | IGHG2 | HDC | TM4SF1 | MYH11 | EGFL6 | FGA | ELF3 |
| AIF1 | BCL11A | KLRD1 | CD8A | ARHGAP24 | IGHG4 | KIT | SLC9A3R2 | NDUFA4L2 | CFB | FABP1 | TACSTD2 |
| RP11-1143G9.4 | SCT | IL2RB | TRBC1 | FCRLA | IGLC7 | IL1RL1 | CRHBP | GPX3 | C1S | TF | LGALS4 |
| HLA-DRB1 | PTCRA | PRF1 | CD8B | CD83 | IGLL5 | RP11-354E11.2 | CLDN5 | IGFBP5 | WFDC2 | AHSG | TNFRSF12A |
| HLA-DRB5 | IL3RA | TRDC | CCL5 | FCER2 | IGLC6 | AC004791.2 | FABP4 | TPM1 | IGFBP6 | ORM2 | CLDN3 |
| MS4A7 | TSPAN13 | CCL4 | AC092580.4 | RP11-693J15.5 | SSR4 | SLC18A2 | IGFBP4 | PLAC9 | PROCR | FGL1 | MMP7 |
| LST1 | PTPRS | CD160 | CD69 | CD22 | IGKV1-12 | FLJ21408 | CLEC14A | DSTN | RARRES1 | ALDOB | EPCAM |
| CTSS | LINC00996 | FGFBP2 | GZMK | FCMR | XBP1 | RAB27B | RNASE1 | RERGL | RAMP1 | KNG1 | SOX4 |
| FCN1 | LILRB4 | CD247 | DUSP2 | AC079767.4 | SEC11C | GATA2 | ENG | c11orf96 | CLDN1 | APOC2 | CA2 |
| SAT1 | LRRC26 | CTSW | TNF | POU2F2 | FKBP11 | LTC4S | PLVAP | MT1M | GAS1 | SAA1 | CYR61 |
| CD5L | SMPD3 | GZMA | ZFP36L2 | ADAM2B | PRDX4 | DHRS9 | VWF | PPP1R14A | CEMIP | APOB | CXCL6 |
| MS4A6A | GAPT | CST7 | CLEC2D | BLK | AL928768.3 | CAPG | ID3 | MFGE8 | TMEM98 | MGST1 | FGGY |
| CTSB | MAP1A | KLRC1 | LCK | SMIM14 | HSP90B1 | MLPH | SPARC | FRZB | MT1G | CYP2E1 | CLDN4 |
| GPX1 | TRAF4 | CCL4L2 | S100A4 | RALGPS2 | TNFRSF17 | FCER1A | CCL14 | COLEC11 | CFH | AZGP1 | ATP1B1 |
| CFD | C12orf75 | SPON2 | SIT1 | HLA-DQA2 | IGLV6-57 | ACOT7 | HSPG2 | MYLK | TMEM176A | SERPINC1 | AKR1C1 |
| FGL2 | MPEG1 | IFITM1 | GZMH | HLADOB | IGLV3-1 | AREG | PLPP1 | CRIP1 | CYSTM1 | HRG | SH3YL1 |
| CD68 | GPR183 | MATK | CD40LG | TNFRSF13C | UBE2J1 | ALOX5 | IFITM3 | TINAGL1 | MT1X | ASGR1 | MTRNR2L8 |
| PSAP | PTPRE | CCL3L3 | SPOCK2 | KIAA0125 | PIM2 | LAPTM4A | EGFL7 | GEM | SOD2 | ANG | EGR1 |
| CPVL | PLP2 | PYHIN1 | GPR171 | HLA-DMB | IGKV4-1 | CD9 | A2M | COX7A1 | MIF | APCS | UGT2B15 |
| LIPA | SEC61B | HOPX | CD48 | 1-Mar | IGLL1 | SAMSN1 | TIMP3 | CSRP2 | S100A10 | AGXT | MTRNR2L12 |
| MARCO | RNASET2 | GZMM | KLGR1 | CD19 | SDF2L1 | CD63 | PCAT19 | SELM | S100A6 | ITIH2 | PERP |
| CD14 | SPCS1 | FCER1G | TRAT1 | NCF1 | JSRP1 | STMN1 | GSN | MFAP4 |  | CP | PLPP2 |
| CD163 | VIMP | CHST12 | RPS29 | CCR7 | SSR3 | CTNNBL1 | MPDC1 | CRISPLD2 |  | GTSA1 | CYP3A5 |
| SLC40A1 |  | TXK | LAT | FAM129C | SPCS3 | ADRB2 | PLPP3 | ISYNA1 |  | ADH1B | BICC1 |
| SPI1 |  | ARPC5L | RPS27 | RPS11 | ERLEC1 | ARHGAP18 | RBP7 | PDGFRB |  | A1BG | SORBS2 |
| SEPP1 |  | APMAP | ACAP1 | LAPTM5 | MANF | PLIN2 | SDPR | PRKCDBP |  | APOA2 | DCDC2 |
| CYBB |  | STK17A | LYAR |  | CRELD2 | JUN | CRIP2 | COX4I2 |  |  |  |
| IFI30 |  | RAC2 | TUBA4A |  | LMAN1 | GLUL | CAV1 | PHLDA2 |  |  |  |
| CTSA |  | LAT2 | PTPN7 |  | PLPP5 | SERPINB1 | ACKR1 | FXYD1 |  |  |  |
| HMOX1 |  | IFNG | TNFAIP3 |  | TXNDC11 | BEX4 | TFF3 |  |  |  |  |
| HLA-DMA |  | IFITM2 | IL2RG |  | PDIA4 | CPM | RAMP3 |  |  |  |  |
| IL1B |  | EOMES | PTPRC |  | FKBP2 | LMO4 | HES1 |  |  |  |  |
| TYMP |  | HCST | RHOH |  | SPCS2 | LMNA | HSPB1 |  |  |  |  |
|  |  | ITGB2 |  |  | MYDGF |  | S100A16 |  |  |  |  |
|  |  | PTGDR |  |  |  |  | APP |  |  |  |  |
|  |  | NFKBIA |  |  |  |  | ECSCR.1 |  |  |  |  |
|  |  | TMIGD2 |  |  |  |  | EMCN |  |  |  |  |
|  |  |  |  |  |  |  | HYAL2 |  |  |  |  |
|  |  |  |  |  |  |  | CTSL |  |  |  |  |
|  |  |  |  |  |  |  | PTRF |  |  |  |  |

**Supplementary Table 7: Cell-subtype specific markers used for deconvolution analysis**

| **Cell Type** | **Gene Name** | **Cell Type** | **Gene Name** | **Cell Type** | **Gene Name** | **Cell Type** | **Gene Name** | **Cell Type** | **Gene Name** | **Cell Type** | **Gene Name** |
| --- | --- | --- | --- | --- | --- | --- | --- | --- | --- | --- | --- |
| Tmo (1) | G0S2 | cDC (1) | CLEC9A | CD4+ T cell (2) | LDHB | Plasma B cell 1 | IGHA2 | MV Endothelia (3) Hepatic Artery | SOX17 | Hepatocyte | APOA1 |
|  | THBS1 |  | IDO1 |  | FXYD5 |  | IGLL1 |  | BCAM |  | ORM1 |
|  | EREG |  | BASP1 |  | RPSA |  | DNAJB9 |  | GJA4 |  | TTR |
|  | FPR1 |  | C1orf54 |  | TRAT1 |  | PDIA4 |  | PODXL |  | FGB |
|  | ACSL1 |  | SNX3 |  | FLT3LG |  | MANF |  | PALMD |  | APOA2 |
| Tmo (2) | LILRA5 | pDC | IRF7 | CD8+ T cell (1) | TMA7 | Plasma B cell 2 | IGHG1 | SA Endothelia (1) | VWA1 | Cholangiocyte 1 | SDC4 |
|  | RNF144B |  | PLD4 |  | LINC00152 |  | IGHG3 |  | FLT1 |  | EGR1 |
|  | ZEB2 |  | LILRA4 |  | STK4 |  | IGHGP |  | TMEM88 |  | BEX1 |
|  | SERPINB9 |  | CCDC50 |  | TUBA4A |  | IGHG2 |  | COL4A1 |  | RHOB |
|  | RASGEF1B |  | PPP1R14B |  |  |  | IGHG4 |  | RAB13 |  | ERRFI1 |
| Tmo (3) | HCK | NK cell (1) | ADGRE5 | CD8+ T cell (2) | CRTAM | Mast cell | TPSB2 | SA Endothelia (2) |  | Cholangiocyte 2 | MTRNR2L12 |
|  | PILRA |  | SYTL3 |  | ITM2A |  | TPSAB1 |  | ACKR1 |  | CLDN4 |
|  | LILRB2 |  | RP5-1171I10.5 |  | LYAR |  | CTSG |  | FKBP1A |  | CYP3A5 |
|  | LYN |  | TMEM2 |  | ACAP1 |  | CPA3 |  |  |  | SPINT2 |
|  | WARS |  | TRA2A |  | EVL |  | TPSD1 |  |  |  | NGFRAP1 |
| SAMac (2) |  | NK cell (2) | IFITM2 | CD8+ T cell (3) | NCR3 | LSEC | CLEC4G | VSMC | MT1M | Cholangiocyte 3 | AKR1C1 |
|  | GPNMB |  | FASLG |  | CH17-373J23.1 |  | FCN2 |  | PHLDA2 |  | SORBS2 |
|  | LGALS3 |  | TXK |  | SLC4A10 |  | CLEC1B |  | PLN |  | CLDN7 |
|  | IGSF6 |  | TIGIT |  | EEF1A1 |  | CLEC4M |  | COX7A1 |  | SERPINA5 |
|  |  |  | SRGN |  | AQP3 |  | OIT3 |  | SELM |  | MT-ND3 |
| KC (1) |  | cNK cell | GNLY | Cycling T cell | CDKN3 | Lymphatic Endothelila | MMRN1 | HSC | GEM |  |  |
|  | PLD3 |  | FGFBP2 |  | GAPDH |  | PPFIBP1 |  | MAP1B |  |  |
|  | VMO1 |  | PRF1 |  | PFN1 |  | PROX1 |  | LHFP |  |  |
|  | BLVRB |  | SPON2 |  | ACTB |  | PDPN |  | SEPT4 |  |  |
|  |  |  | PYHIN1 |  | IDH2 |  | NRP2 |  | CRYAB |  |  |
| KC (2) | MSR1 | Cycling ILC/Cycling NK | MCM7 | B cell 1 | RP5-887A10.1 | MV Endothelia (1) Portal Vein | CD320 | SAMes/Myofibroblast | LUM |  |  |
|  | MARCKS |  | ASF1B |  | AC079767.4 |  | LDB2 |  | IGFBP3 |  |  |
|  | LY96 |  | H2AFX |  | BLK |  | SRPX |  | COL6A2 |  |  |
|  | FPR3 |  | NUCKS1 |  | AIM2 |  | EDN1 |  | COL6A1 |  |  |
|  | MAFB |  | ARL6IP1 |  | CD19 |  | SCGB3A1 |  | DPT |  |  |
| cDC (2) | CD1E | CD4+ T cell (1) | RPL17 | B cell 2 | FCER2 | MV Endothelia (2) Central Vein | LYPD2 | Mesothelia | PLA2G2A |  |  |
|  | C15orf48 |  | RPL34 |  | FAM129C |  | INMT |  | ITLN1 |  |  |
|  | CTSH |  | RPL36A |  | RP11-231C14.7 |  | IL1R1 |  | PRG4 |  |  |
|  | PKIB |  | RPS4Y1 |  | NCF1 |  | RSPO3 |  | UPK3B |  |  |
|  | SGK1 |  | RORA |  | MEF2C |  | FBLN2 |  | PTGIS |  |  |

**Supplementary Table 8. Deconvolution revealed changes in specific cell populations between F3-4 and F0-1.**

| **Cell Type** | **RNA-Seq+Microarray** | | | **RNA-Seq** | | | | **Microarray** | | |
| --- | --- | --- | --- | --- | --- | --- | --- | --- | --- | --- |
|  | **Adj. *P*-value**  **(2 sided)** | **Adj. *P*-value greater** | **Adj. *P*-value smaller** | **Adj. *P*-value**  **(2 sided)** | | **Adj. *P-*value greater** | **Adj. *P*-value smaller** | **Adj. *P*-value**  **(2 sided)** | **Adj. *P*-value greater** | **Adj. *P*-value smaller** |
| **TMo (1)** | **0.005** | **0.040** | 1.000 | **0.028** | 0.318 | | 1.000 | 0.912 | 0.785 | 1.000 |
| **TMo (2)** | 0.082 | 0.395 | 1.000 | 0.226 | 0.635 | | 1.000 | 1.000 | 1.000 | 1.000 |
| **TMo (3)** | 0.133 | 0.364 | 1.000 | 0.225 | 1.000 | | 1.000 | 1.000 | 1.000 | 1.000 |
| **SAMac (2)** | **0.006** | **1.02E-06** | 1.000 | 0.138 | **0.001** | | 1.000 | 0.097 | **0.006** | 1.000 |
| **KC (1)** | **0.002** | 1.000 | 0.904 | **0.002** | 1.000 | | 1.000 | 1.000 | 1.000 | 1.000 |
| **KC (2)** | **0.028** | **0.004** | 1.000 | 0.196 | **0.011** | | 1.000 | 0.602 | 1.000 | 1.000 |
| **CDC (2)** | **0.003** | **0.023** | 1.000 | **0.009** | 0.141 | | 1.000 | 1.000 | 1.000 | 1.000 |
| **CDC (1)** | 0.265 | 0.364 | 1.000 | 0.226 | 0.093 | | 1.000 | 1.000 | 1.000 | 1.000 |
| **pDC** | **0.003** | **0.020** | 1.000 | **3.41E-04** | **0.003** | | 1.000 | 1.000 | 1.000 | 1.000 |
| **NK cell (1)** | **0.014** | 0.006 | 1.000 | **0.013** | **0.011** | | 1.000 | 1.000 | 1.000 | 1.000 |
| **NK cell (2)** | **0.031** | **0.010** | 1.000 | **9.36E-05** | **0.001** | | 1.000 | 1.000 | 1.000 | 1.000 |
| **cNK cell** | **0.035** | **0.023** | 1.000 | 0.085 | **0.029** | | 1.000 | 1.000 | 1.000 | 1.000 |
| **Cycling ILC** | **0.009** | **0.010** | 1.000 | 0.196 | 0.404 | | 1.000 | **0.046** | **0.003** | 1.000 |
| **CD4+ T cell (1)** | **0.041** | 1.000 | 1.000 | 0.196 | 1.000 | | 1.000 | 1.000 | 1.000 | 1.000 |
| **CD4+ T cell (2)** | **0.024** | **0.017** | 1.000 | 0.085 | **0.006** | | 1.000 | 1.000 | 1.000 | 1.000 |
| **CD8+ T cell (1)** | 0.133 | **0.002** | 1.000 | 0.349 | 0.067 | | 1.000 | 0.570 | **0.040** | 1.000 |
| **CD8+ T cell (2)** | **0.011** | **0.009** | 1.000 | **0.009** | **0.005** | | 1.000 | 1.000 | 1.000 | 1.000 |
| **CD8+ T cell (3)** | 0.083 | 1.000 | 1.000 | 0.196 | 1.000 | | 1.000 | 1.000 | 1.000 | 1.000 |
| **Cycling T cell** | **0.041** | **0.002** | 1.000 | 0.226 | 0.124 | | 1.000 | 0.168 | **0.011** | 1.000 |
| **B cell (1)** | **0.011** | 0.057 | 1.000 | **0.004** | 0.169 | | 1.000 | 1.000 | 1.000 | 1.000 |
| **B cell (2)** | **0.024** | **0.006** | 1.000 | **0.034** | **0.014** | | 1.000 | 1.000 | 1.000 | 1.000 |
| **Plasma B cell (1)** | **0.018** | 0.057 | 1.000 | 0.138 | 0.810 | | 1.000 | 0.671 | **0.051** | 1.000 |
| **Plasma B cell (2)** | 0.056 | **0.006** | 1.000 | 0.138 | **0.049** | | 1.000 | 1.000 | 0.663 | 1.000 |
| **Mast cell** | 0.083 | **0.006** | 1.000 | **0.020** | **0.014** | | 1.000 | 1.000 | 1.000 | 1.000 |
| **LSEC** | 0.090 | 1.000 | **3.25E-04** | 0.158 | 1.000 | | **0.002** | 1.000 | 1.000 | 1.000 |
| **Lymphatic**  **Endothelia** | 0.265 | 0.097 | 1.000 | 0.244 | 0.580 | | 1.000 | 1.000 | 0.825 | 1.000 |
| **MVEnd (1)**  **(Portal Vein)** | **2.84E-06** | **0.039** | 1.000 | **9.36E-05** | **0.019** | | 1.000 | 0.197 | 1.000 | 1.000 |
| **MVEnd (2)**  **(Central Vein)** | **0.030** | **0.005** | 1.000 | 0.196 | **0.049** | | 1.000 | 0.602 | 0.539 | 1.000 |
| **MVEnd (3)**  **(Hepatic Artery)** | **0.005** | **4.83E-04** | 1.000 | **0.002** | **4.02E-06** | | 1.000 | 1.000 | 1.000 | 1.000 |
| **SAEnd (1)** | **0.013** | **0.006** | 1.000 | **0.035** | **0.003** | | 1.000 | 1.000 | 1.000 | 1.000 |
| **SAEnd (2)** | **0.017** | 0.273 | 1.000 | **0.002** | 0.318 | | 1.000 | 1.000 | 1.000 | 1.000 |
| **VSMC** | **3.52E-04** | **0.023** | 1.000 | **0.036** | **0.004** | | 1.000 | **0.012** | 1.000 | 1.000 |
| **HSC** | **9.87E-08** | **2.54E-06** | 1.000 | **9.38E-08** | **1.83E-10** | | 1.000 | 0.494 | 1.000 | 1.000 |
| **SAMes** | **2.58E-11** | **3.15E-15** | 1.000 | **6.32E-11** | **1.23E-13** | | 1.000 | 0.063 | **0.004** | 1.000 |
| **Mesothelia** | 0.265 | 0.582 | 1.000 | 0.356 | 1.000 | | 1.000 | 1.000 | 1.000 | 1.000 |
| **Hepatocyte** | **4.26E-05** | 1.000 | 1.000 | **0.007** | 1.000 | | 0.821 | **0.023** | 1.000 | 1.000 |
| **Cholangiocyte (1)** | **0.006** | 0.364 | 1.000 | **0.019** | 0.364 | | 1.000 | 1.000 | 1.000 | 1.000 |
| **Cholangiocyte (2)** | **0.014** | **0.009** | 1.000 | 0.067 | **0.032** | | 1.000 | 1.000 | 1.000 | 1.000 |
| **Cholangiocyte (3)** | **0.047** | 1.000 | 1.000 | 0.196 | 1.000 | | 1.000 | 1.000 | 1.000 | 1.000 |

Statistics were calculated to identify differences in cell populations between F3-4 and F0-1 (2 sided), in addition to whether cell populations were greater or smaller in F3-4 vs F0-1.

**Supplementary Table 9. Enrichment of ligand-receptor pairs in major cell types.**

| **Process** | **Ligand  Receptor** | **Expression** | **MP** | **pDC** | **ILC** | **T cell** | **B cell** | **Plasma B cell** | **Mast cell** | **Endothelia** | **Mesenchyme** | **Mesothelia** | **Hepatocyte** | **Cholangiocyte** |
| --- | --- | --- | --- | --- | --- | --- | --- | --- | --- | --- | --- | --- | --- | --- |
| **Unknown** | **EFEMP2** | Avg. | 0.02 | 0.01 | 0.05 | 0.01 | 0.01 | 0.02 | 0.00 | 0.41 | 0.94 | **1.63** | 0.05 | 0.28 |
|  |  | Prop. | 1.14 | 0.70 | 1.27 | 0.27 | 0.29 | 4.38 | 0.00 | 18.77 | 29.03 | **76.85** | 7.19 | 14.60 |
|  | **AQP1** | Avg. | 0.07 | 0.03 | 0.06 | 0.05 | 0.04 | 0.07 | 0.00 | **3.30** | 0.18 | **3.22** | 0.64 | **3.31** |
|  |  | Prop. | 4.27 | 1.75 | 1.77 | 1.23 | 1.33 | 9.89 | 0.00 | **57.51** | 7.20 | **87.96** | 31.25 | **66.46** |
| **ECM Interactions** | **COL4A1** | Avg. | 0.03 | 0.00 | 0.02 | 0.01 | 0.02 | 0.02 | 0.00 | **2.16** | 0.84 | 0.07 | 0.09 | 0.29 |
|  |  | Prop. | 1.72 | 0.00 | 0.55 | 0.33 | 0.52 | 3.15 | 0.00 | **50.83** | 22.06 | 10.19 | 10.94 | 14.84 |
|  | ITGB1 | Avg. | **1.12** | 0.31 | **1.23** | **1.03** | 0.76 | 0.39 | 0.62 | **3.93** | **5.29** | **1.77** | **2.02** | **3.44** |
|  |  | Prop. | 43.63 | 17.19 | 26.45 | 21.70 | 17.99 | 34.27 | 15.71 | **76.35** | **76.00** | **81.48** | **78.13** | **68.77** |
|  | **LUM** | Avg. | 0.01 | 0.00 | 0.01 | 0.00 | 0.01 | 0.01 | 0.00 | 0.03 | **3.55** | 0.93 | 0.03 | 0.03 |
|  |  | Prop. | 0.65 | 0.00 | 0.21 | 0.15 | 0.17 | 1.91 | 0.00 | 1.31 | 14.27 | 21.30 | 1.88 | 1.22 |
|  | ITGB1 | Avg. | **1.12** | 0.31 | **1.23** | **1.03** | 0.76 | 0.39 | 0.62 | **3.93** | **5.29** | **1.77** | **2.02** | **3.44** |
|  |  | Prop. | 43.63 | 17.19 | 26.45 | 21.70 | 17.99 | 34.27 | 15.71 | **76.35** | **76.00** | **81.48** | **78.13** | **68.77** |
|  | **SPP1** | Avg. | 0.76 | 0.07 | 0.07 | 0.06 | 0.07 | 0.09 | 0.00 | 0.23 | 0.19 | 0.05 | **3.10** | **14.32** |
|  |  | Prop. | 6.51 | 3.16 | 1.89 | 1.72 | 2.26 | 13.37 | 0.00 | 6.09 | 5.43 | 5.56 | 46.88 | **79.58** |
|  | ITGB1 | Avg. | **1.12** | 0.31 | **1.23** | **1.03** | 0.76 | 0.39 | 0.62 | **3.93** | **5.29** | **1.77** | **2.02** | **3.44** |
|  |  | Prop. | 43.63 | 17.19 | 26.45 | 21.7 | 17.99 | 34.27 | 15.71 | **76.35** | **76.00** | **81.48** | **78.13** | **68.77** |
|  | **MMP7** | Avg. | 0.02 | 0.01 | 0.02 | 0.02 | 0.02 | 0.01 | 0.00 | 0.03 | 0.05 | 0.04 | 0.33 | **4.73** |
|  |  | Prop. | 0.92 | 0.70 | 0.62 | 0.47 | 0.46 | 1.91 | 0.00 | 1.53 | 1.90 | 6.48 | 15.31 | **57.94** |
|  | **CDH6** | Avg. | 0.00 | 0.00 | 0.01 | 0.01 | 0.00 | 0.00 | 0.00 | 0.01 | **1.24** | 0.01 | 0.26 | 0.56 |
|  |  | Prop. | 0.16 | 0.00 | 0.11 | 0.15 | 0.06 | 0.90 | 0.00 | 0.38 | 28.71 | 1.85 | 19.06 | 22.53 |
|  | **VIM** | Avg. | **13.24** | **11.33** | **2.77** | **8.28** | **4.15** | **4.71** | **16.72** | **34.99** | **30.07** | **14.69** | **1.26** | **3.88** |
|  |  | Prop. | **91.60** | **96.14** | 41.45 | **73.28** | **64.08** | **91.69** | **87.14** | **87.98** | **96.69** | **97.22** | **59.06** | **66.58** |
|  | CD44 | Avg. | **3.47** | **1.04** | **1.35** | **2.78** | **1.34** | 0.51 | **5.76** | 0.09 | 0.49 | 0.55 | 0.07 | 0.16 |
|  |  | Prop. | **68.70** | 41.05 | 26.87 | 49.05 | 35.34 | 48.54 | **67.14** | 4.60 | 16.85 | 43.52 | 9.38 | 7.75 |
| **Immune cell recruitment and interactions** | SELPLG | Avg. | 0.51 | **1.71** | 0.58 | 0.50 | 0.08 | 0.13 | 0.03 | 0.04 | 0.02 | 0.01 | 0.02 | 0.03 |
|  |  | Prop. | 23.31 | **56.14** | 14.04 | 13.89 | 3.07 | 23.15 | 1.43 | 2.01 | 0.95 | 1.85 | 4.06 | 1.96 |
|  | SELL | Avg. | 0.71 | **4.27** | 0.52 | 0.48 | **2.70** | 0.07 | 0.05 | 0.05 | 0.02 | 0.00 | 0.01 | 0.03 |
|  |  | Prop. | 20.82 | **84.21** | 9.07 | 10.21 | **50.78** | 12.13 | 1.43 | 2.51 | 0.86 | 0.00 | 2.81 | 1.54 |
|  | HLA-A | Avg. | **18.08** | **14.09** | **40.26** | **36.04** | **20.58** | **7.93** | **13.47** | **30.29** | **7.45** | **7.57** | **8.93** | **12.90** |
|  |  | Prop. | **95.51** | **97.19** | **98.58** | **98.37** | **97.34** | **95.06** | **80.00** | **99.40** | **80.30** | **96.30** | **96.25** | **91.30** |
|  | **CD3D** | Avg. | 0.35 | 0.37 | 0.73 | **8.88** | 0.45 | 0.36 | 0.44 | 0.10 | 0.17 | 0.10 | 0.16 | 0.18 |
|  |  | Prop. | 14.41 | 18.25 | 12.85 | **82.88** | 14.52 | 35.62 | 10.00 | 4.39 | 4.48 | 12.96 | 17.81 | 7.36 |
|  | HLA-B | Avg. | **35.75** | **39.06** | **61.16** | **56.65** | **39.35** | **23.44** | **17.27** | **50.44** | **9.75** | **14.32** | **9.56** | **12.33** |
|  |  | Prop. | **98.71** | **99.30** | **99.59** | **99.48** | **99.07** | **99.55** | **91.43** | **99.87** | **84.56** | **99.07** | **97.19** | **91.54** |
|  | **CD3D** | Avg. | 0.35 | 0.37 | 0.73 | **8.88** | 0.45 | 0.36 | 0.44 | 0.10 | 0.17 | 0.10 | 0.16 | 0.18 |
|  |  | Prop. | 14.41 | 18.25 | 12.85 | **82.88** | 14.52 | 35.62 | 10.00 | 4.39 | 4.48 | 12.96 | 17.81 | 7.36 |
|  | B2M | Avg. | **156.36** | **126.50** | **191.19** | **208.10** | **118.13** | **83.89** | **139.11** | **152.99** | **51.39** | **51.06** | **35.47** | **42.84** |
|  |  | Prop. | **99.98** | **100.00** | **100.00** | **100.00** | **100.00** | **99.89** | **100.00** | **100.00** | **99.32** | **100.00** | **100.00** | **99.41** |
|  | **CD3D** | Avg. | 0.35 | 0.37 | 0.73 | **8.88** | 0.45 | 0.36 | 0.44 | 0.10 | 0.17 | 0.10 | 0.16 | 0.18 |
|  |  | Prop. | 14.41 | 18.25 | 12.85 | **82.88** | 14.52 | 35.62 | 10.00 | 4.39 | 4.48 | 12.96 | 17.81 | 7.36 |
|  | **CCL19** | Avg. | 0.06 | 0.00 | 0.01 | 0.01 | 0.00 | 0.02 | 0.05 | 0.03 | **2.22** | 0.04 | 0.04 | 0.02 |
|  |  | Prop. | 2.20 | 0.00 | 0.13 | 0.20 | 0.17 | 2.47 | 1.43 | 1.26 | 5.21 | 5.56 | 1.88 | 1.01 |
|  | **CXCR3** | Avg. | 0.04 | **2.25** | 0.22 | 0.91 | 0.28 | 0.12 | 0.00 | 0.01 | 0.01 | 0.01 | 0.02 | 0.01 |
|  |  | Prop. | 3.32 | **60.00** | 6.40 | 21.54 | 8.91 | 16.74 | 0.00 | 0.68 | 0.18 | 0.93 | 1.88 | 0.53 |
|  | SYK | Avg. | 0.66 | **1.37** | 0.38 | 0.03 | 0.63 | 0.11 | 0.08 | 0.02 | 0.02 | 0.00 | 0.03 | 0.11 |
|  |  | Prop. | 30.89 | 49.47 | 9.74 | 0.81 | 18.91 | 17.64 | 2.86 | 1.01 | 0.63 | 0.00 | 4.69 | 6.89 |
|  | **FCGR2A** | Avg. | **1.33** | 0.01 | 0.04 | 0.02 | 0.04 | 0.02 | 0.54 | 0.03 | 0.08 | 0.10 | 0.01 | 0.03 |
|  |  | Prop. | 44.00 | 0.70 | 1.02 | 0.63 | 1.79 | 4.72 | 2.86 | 1.66 | 4.03 | 14.81 | 3.13 | 1.72 |
|  | **VCAN** | Avg. | **3.39** | 0.06 | 0.05 | 0.05 | 0.02 | 0.03 | 0.11 | 0.07 | 0.31 | 0.00 | 0.26 | 0.54 |
|  |  | Prop. | 29.44 | 3.51 | 1.31 | 1.27 | 0.64 | 5.84 | 1.43 | 2.96 | 7.88 | 0.00 | 19.38 | 19.26 |
|  | CD44 | Avg. | **3.47** | **1.04** | **1.35** | **2.78** | **1.34** | 0.51 | **5.76** | 0.09 | 0.49 | 0.55 | 0.07 | 0.16 |
|  |  | Prop. | **68.70** | 41.05 | 26.87 | 49.05 | 35.34 | 48.54 | **67.14** | 4.60 | 16.85 | 43.52 | 9.38 | 7.75 |
|  | **ANXA1** | Avg. | **3.53** | 0.39 | **2.97** | **6.16** | 0.31 | 0.27 | **30.24** | **2.89** | **1.62** | **6.17** | 0.17 | 0.27 |
|  |  | Prop. | **65.82** | 17.19 | 44.85 | **63.73** | 9.49 | 36.29 | **88.57** | **54.36** | 31.93 | **93.52** | 20.00 | 10.72 |
|  | FPR1 | Avg. | **1.11** | 0.01 | 0.02 | 0.02 | 0.02 | 0.01 | 0.17 | 0.02 | 0.02 | 0.01 | 0.01 | 0.02 |
|  |  | Prop. | 32.91 | 0.35 | 0.49 | 0.64 | 0.75 | 2.47 | 2.86 | 0.85 | 0.68 | 0.93 | 2.19 | 0.80 |
|  | **ANXA1** | Avg. | **3.53** | 0.39 | **2.97** | **6.16** | 0.31 | 0.27 | **30.24** | **2.89** | **1.62** | **6.17** | 0.17 | 0.27 |
|  |  | Prop. | **65.82** | 17.19 | 44.85 | **63.73** | 9.49 | 36.29 | **88.57** | **54.36** | 31.93 | **93.52** | 20.00 | 10.72 |
|  | **FPR3** | Avg. | **1.25** | 0.01 | 0.02 | 0.01 | 0.01 | 0.02 | 0.03 | 0.02 | 0.01 | 0.01 | 0.01 | 0.01 |
|  |  | Prop. | 36.95 | 0.70 | 0.49 | 0.38 | 0.40 | 4.38 | 1.43 | 1.14 | 0.50 | 0.93 | 1.25 | 0.53 |
| **Fibrosis** | **PDGFD** | Avg. | 0.01 | 0.00 | 0.46 | 0.08 | 0.02 | 0.01 | 0.00 | 0.31 | 0.06 | 0.45 | 0.07 | **1.04** |
|  |  | Prop. | 0.59 | 0.00 | 10.98 | 2.05 | 0.40 | 3.26 | 0.00 | 12.10 | 3.31 | 36.11 | 9.06 | 37.87 |
|  | **PDGFRA** | Avg. | 0.00 | 0.00 | 0.00 | 0.00 | 0.00 | 0.00 | 0.00 | 0.02 | 0.28 | 0.91 | 0.03 | 0.00 |
|  |  | Prop. | 0.20 | 0.00 | 0.04 | 0.02 | 0.00 | 0.34 | 0.00 | 1.28 | 8.88 | **58.33** | 4.06 | 0.36 |
| **Endothelial regulation** | MFGE8 | Avg. | 0.03 | 0.05 | 0.12 | 0.08 | 0.03 | 0.05 | 0.00 | 0.23 | **6.61** | 0.24 | 0.10 | 0.20 |
|  |  | Prop. | 1.65 | 2.11 | 3.11 | 2.15 | 0.75 | 9.55 | 0.00 | 11.47 | **75.54** | 30.56 | 10.63 | 10.15 |
|  | **PDGFRB** | Avg. | 0.01 | 0.00 | 0.10 | 0.01 | 0.02 | 0.02 | 0.00 | 0.01 | **3.82** | 0.09 | 0.02 | 0.04 |
|  |  | Prop. | 0.44 | 0.00 | 2.07 | 0.28 | 0.46 | 3.15 | 0.00 | 0.71 | **64.09** | 6.48 | 3.44 | 1.81 |

Ligands and receptors with an average expression greater than 1 and proportional expression in more than 50% of cells were used as cutoff criteria. Interactions between ligands and receptors within the same cell type were highlighted (boxed), as well as potential cell-to-cell communications where both average and proportional expression met the cutoff thresholds. Avg (Average expression): Mean expression level of the ligand or receptor. Prop (Proportional expression): Percentage of cells in which the ligand or receptor is expressed. Bolded genes represent DEGs identified by the meta-analysis.

**Supplementary Table 11: Ligand and receptor average and proportional expression in sub-cell types.**

| **Process** | **Ligand**  **Receptor** | **Expression** | **Tmo_1_** | **Tmo_2_** | **Tmo_3_** | **SAMac_1_** | **SAMac_2_** | **KC_1_** | **KC_2_** | **CDC_1_** | **CDC_2_** | **Cycling MP_1_** | **Cycling MP_2_** | **Cycling MP_3_** | **Cycling MP_4_** | **pDC** | **NK_1_** | **NK_2_** | **NK_3_** | **Cycling NK_1_** | **Cycling NK_2_** | **CD4+ T cell_1_** | **CD4+ T cell_2_** | **CD8+ T cell_1_** | **CD8+ T cell_2_** | **CD8+ T cell_3_** | **Cycling T cell** | **B cell_1_** | **B cell_2_** | **Plasma B cell_1_** | **Plasma B cell_2_** | **Mast cells** | **LSEC** | **Lymphatic Endothelia** | **MV Endothelia (PV)** | **MV Endothelia (CV)** | **MV Endothelia (HA)** | **SA Endothelia_1_** | **SA Endothelia_2_** | **VSMC** | **HSC** | **Myofibroblast** | **Mesothelia** | **Hepatocyte** | **Cholangiocyte_1_** | **Cholangiocyte_2_** | **Cholangiocyte_3_** |
| --- | --- | --- | --- | --- | --- | --- | --- | --- | --- | --- | --- | --- | --- | --- | --- | --- | --- | --- | --- | --- | --- | --- | --- | --- | --- | --- | --- | --- | --- | --- | --- | --- | --- | --- | --- | --- | --- | --- | --- | --- | --- | --- | --- | --- | --- | --- | --- |
| **Immune cell interactions** | SELPLG | Avg. | <1 | <1 | <1 | 0 | 0 | <1 | 0 | 0 | <1 | <1 | 0 | <1 | 0 | **2** | 0 | 0 | **1** | 0 | **1** | 0 | <1 | 0 | <1 | 0 | 0 | 0 | 0 | 0 | 0 | 0 | 0 | 0 | 0 | 0 | 0 | 0 | 0 | 0 | 0 | 0 | 0 | 0 | 0 | 0 | 0 |
|  |  | Prop. | 15 | 13 | 42 | 19 | 23 | 31 | 10 | 25 | 28 | 38 | 32 | **57** | 0 | **56** | 2 | 8 | 28 | 8 | 46 | 8 | 19 | 5 | 16 | 18 | 24 | 3 | 3 | 22 | 24 | 1 | 1 | 3 | 2 | 1 | 2 | 2 | 2 | 0 | 1 | 2 | 2 | 4 | 1 | 1 | 4 |
|  | SELL | Avg. | **3** | **1** | 0 | 0 | 0 | 0 | 0 | 0 | <1 | <1 | 0 | **1** | 0 | **4** | 0 | 0 | **1** | 0 | **2** | 0 | **1** | 0 | 0 | 0 | <1 | **2** | **3** | 0 | 0 | 0 | 0 | 0 | 0 | 0 | 0 | 0 | 0 | 0 | 0 | 0 | 0 | 0 | 0 | 0 | 0 |
|  |  | Prop. | 46 | 18 | 19 | 18 | 12 | 14 | 7 | 22 | 29 | 40 | 14 | **82** | 17 | **84** | 1 | 3 | 21 | 5 | 41 | 3 | 23 | 2 | 5 | 2 | 20 | 49 | **53** | 9 | 16 | 1 | 1 | 2 | 5 | 11 | 1 | 1 | 4 | 1 | 0 | 2 | 0 | 3 | 1 | 1 | 4 |
|  | **VCAN** | Avg. | **21** | **7** | **1** | **2** | 0 | 0 | 0 | 0 | 0 | <1 | <1 | 0 | 0 | 0 | 0 | 0 | 0 | 0 | 0 | 0 | 0 | 0 | 0 | 0 | 0 | 0 | 0 | 0 | 0 | 0 | 0 | 0 | 0 | 0 | 0 | 0 | 0 | 0 | 0 | **2** | 0 | 0 | <1 | 0 | <1 |
|  |  | Prop. | **94** | 49 | 34 | 46 | 9 | 2 | 5 | 15 | 5 | 29 | 25 | 11 | 8 | 4 | 1 | 2 | 1 | 4 | 2 | 2 | 1 | 1 | 1 | 2 | 3 | 1 | 1 | 6 | 6 | 1 | 1 | 7 | 1 | 11 | 2 | 2 | 5 | 1 | 3 | 46 | 0 | 19 | 13 | 16 | 33 |
|  | CD44 | Avg. | **8** | **5** | **3** | **3** | **2** | **3** | **5** | **3** | **1** | **2** | **3** | <1 | **3** | **1** | **2** | **1** | **1** | **1** | **1** | **3** | **3** | **3** | **3** | **2** | **3** | **2** | <1 | <1 | 0 | **6** | 0 | 0 | 0 | 0 | 0 | 0 | 0 | 0 | <1 | <1 | <1 | 0 | 0 | 0 | 0 |
|  |  | Prop. | **80** | **52** | **76** | **70** | **60** | **79** | **62** | **78** | 43 | **77** | **86** | **57** | **50** | 41 | 17 | 32 | 26 | 40 | 35 | 36 | **61** | 32 | **53** | 48 | **70** | 42 | 26 | 45 | **52** | **67** | 5 | 3 | 4 | 3 | 10 | 3 | 3 | 8 | 20 | 36 | 44 | 9 | 4 | 5 | 18 |
|  | **COL4A1** | Avg. | 0 | 0 | 0 | 0 | 0 | 0 | 0 | 0 | 0 | 0 | 0 | 0 | 0 | 0 | 0 | 0 | 0 | 0 | 0 | 0 | 0 | 0 | 0 | 0 | 0 | 0 | 0 | 0 | 0 | 0 | **3** | <1 | 0 | 0 | **1** | **4** | **2** | 0 | <1 | **2** | 0 | 0 | 0 | 0 | 0 |
|  |  | Prop. | 1 | 1 | 1 | 2 | 2 | 3 | 1 | 2 | 1 | 1 | 5 | 0 | 0 | 0 | 0 | 1 | 1 | 1 | 1 | 0 | 1 | 0 | 0 | 0 | 2 | 0 | 1 | 2 | 4 | 0 | **59** | 36 | 20 | 23 | 41 | **74** | 46 | 11 | 24 | **54** | 10 | 11 | 10 | 12 | 27 |
|  | ITGB1 | Avg. | <1 | **1** | **1** | <1 | **1** | **1** | **2** | <1 | <1 | <1 | **1** | **1** | <1 | 0 | **1** | **1** | **1** | **1** | **2** | <1 | **1** | <1 | <1 | 0 | **1** | <1 | 0 | <1 | 0 | <1 | **5** | **4** | **4** | **3** | **5** | **4** | **3** | **5** | **6** | **5** | **2** | **2** | **4** | **3** | **5** |
|  |  | Prop. | 24 | 21 | 49 | 43 | **52** | **60** | 33 | **54** | 37 | **56** | **70** | **68** | 42 | 17 | 15 | 29 | 29 | 49 | **53** | 13 | 33 | 12 | 21 | 9 | 44 | 23 | 11 | 37 | 32 | 16 | **78** | **78** | **76** | **84** | **82** | **78** | **69** | **69** | **82** | **84** | **81** | **78** | **59** | **65** | **87** |
| **Unknown** | **EFEMP2** | Avg. | 0 | 0 | 0 | 0 | 0 | 0 | 0 | 0 | 0 | 0 | 0 | 0 | 0 | 0 | 0 | 0 | 0 | 0 | 0 | 0 | 0 | 0 | 0 | 0 | 0 | 0 | 0 | 0 | 0 | 0 | <1 | 0 | 0 | 0 | 0 | <1 | 0 | <1 | <1 | **2** | **2** | 0 | 0 | 0 | 0 |
|  |  | Prop. | 1 | 1 | 1 | 1 | 2 | 3 | 0 | 1 | 1 | 1 | 5 | 0 | 0 | 1 | 1 | 1 | 1 | 3 | 2 | 0 | 0 | 0 | 0 | 0 | 0 | 0 | 1 | 6 | 3 | 0 | 22 | 25 | 17 | 24 | 22 | 23 | 10 | 16 | 30 | **71** | **77** | 7 | 12 | 14 | 18 |
|  | **AQP1** | Avg. | 0 | 0 | 0 | 0 | 0 | 0 | 0 | 0 | 0 | 0 | 0 | 0 | 0 | 0 | 0 | 0 | 0 | 0 | 0 | 0 | 0 | 0 | 0 | 0 | 0 | 0 | 0 | 0 | 0 | 0 | <1 | 0 | **5** | <1 | **5** | **4** | **4** | 0 | 0 | 0 | **3** | <1 | **3** | **3** | **5** |
|  |  | Prop. | 2 | 1 | 4 | 4 | 5 | 8 | 1 | 6 | 3 | 3 | 11 | 4 | 8 | 2 | 0 | 2 | 2 | 4 | 5 | 1 | 2 | 1 | 1 | 1 | 4 | 1 | 2 | 11 | 9 | 0 | 15 | 8 | **83** | 30 | **74** | **60** | **74** | 4 | 8 | 15 | **88** | 31 | **52** | **67** | **81** |
| **Immune cell recruitment** | SYK | Avg. | <1 | <1 | <1 | <1 | <1 | 0 | 0 | <1 | <1 | <1 | <1 | <1 | 0 | **1** | 0 | 0 | 0 | <1 | 0 | 0 | 0 | 0 | 0 | 0 | 0 | <1 | <1 | 0 | 0 | 0 | 0 | 0 | 0 | 0 | 0 | 0 | 0 | 0 | 0 | 0 | 0 | 0 | 0 | 0 | 0 |
|  |  | Prop. | 21 | 12 | 29 | 38 | 40 | 28 | 12 | 46 | 29 | **57** | **73** | **64** | 25 | 49 | 5 | 13 | 7 | 36 | 19 | 1 | 1 | 1 | 1 | 1 | 3 | 22 | 15 | 14 | 21 | 3 | 1 | 1 | 1 | 1 | 1 | 1 | 1 | 1 | 1 | 0 | 0 | 5 | 5 | 6 | 11 |
|  | **FCGR2A** | Avg. | **1** | <1 | **1** | **1** | **2** | **1** | <1 | **2** | <1 | **1** | **2** | 0 | <1 | 0 | 0 | 0 | 0 | 0 | 0 | 0 | 0 | 0 | 0 | 0 | 0 | 0 | 0 | 0 | 0 | <1 | 0 | 0 | 0 | 0 | 0 | 0 | 0 | 0 | 0 | 0 | 0 | 0 | 0 | 0 | 0 |
|  |  | Prop. | 30 | 13 | 48 | 47 | **69** | 48 | 18 | **64** | 19 | **69** | **84** | 0 | 33 | 1 | 0 | 1 | 2 | 1 | 2 | 1 | 1 | 0 | 1 | 0 | 2 | 2 | 1 | 5 | 5 | 3 | 2 | 13 | 1 | 2 | 1 | 1 | 1 | 3 | 5 | 4 | 15 | 3 | 2 | 1 | 3 |
|  | **ANXA1** | Avg. | **7** | **3** | **3** | **3** | **3** | **1** | **3** | **5** | **3** | **6** | **5** | **2** | **3** | 0 | **1** | **2** | **5** | **2** | **5** | **5** | **8** | **4** | **6** | **4** | **5** | 0 | 0 | 0 | 0 | **30** | 0 | **3** | **3** | 0 | **3** | **3** | **4** | <1 | **1** | **5** | **6** | 0 | 0 | 0 | 0 |
|  |  | Prop. | **75** | 37 | **68** | **71** | **73** | **53** | 40 | **90** | **57** | **93** | **91** | **79** | **58** | 17 | 17 | 40 | **66** | **53** | **83** | 47 | **80** | 32 | **73** | **76** | **72** | 12 | 6 | 25 | 49 | **89** | 10 | **54** | **52** | 17 | **62** | **62** | **76** | 17 | 33 | **76** | **94** | 20 | 9 | 8 | 18 |
|  | FPR1 | Avg. | **3** | **2** | **1** | **1** | <1 | 0 | <1 | <1 | 0 | 0 | **1** | 0 | 0 | 0 | 0 | 0 | 0 | 0 | 0 | 0 | 0 | 0 | 0 | 0 | 0 | 0 | 0 | 0 | 0 | 0 | 0 | 0 | 0 | 0 | 0 | 0 | 0 | 0 | 0 | 0 | 0 | 0 | 0 | 0 | 0 |
|  |  | Prop. | **53** | 23 | 49 | 42 | 28 | 28 | 23 | 29 | 8 | 37 | 48 | 7 | 0 | 0 | 1 | 0 | 0 | 2 | 2 | 1 | 1 | 1 | 1 | 1 | 0 | 1 | 1 | 2 | 3 | 3 | 1 | 1 | 1 | 0 | 1 | 1 | 1 | 1 | 1 | 0 | 1 | 2 | 1 | 1 | 1 |
| **Immune cell signaling** | **ANXA1** | Avg. | **7** | **3** | **3** | **3** | **3** | **1** | **3** | **5** | **3** | **6** | **5** | **2** | **3** | 0 | **1** | **2** | **5** | **2** | **5** | **5** | **8** | **4** | **6** | **4** | **5** | 0 | 0 | 0 | 0 | **30** | 0 | **3** | **3** | 0 | **3** | **3** | **4** | <1 | **1** | **5** | **6** | 0 | 0 | 0 | 0 |
|  |  | Prop. | **75** | 37 | **68** | **71** | **73** | **53** | 40 | **90** | **57** | **93** | **91** | **79** | **58** | 17 | 17 | 40 | **66** | **53** | **83** | 47 | **80** | 32 | **73** | **76** | **72** | 12 | 6 | 25 | 49 | **89** | 10 | **54** | **52** | 17 | **62** | **62** | **76** | 17 | 33 | **76** | **94** | 20 | 9 | 8 | 18 |
|  | **FPR3** | Avg. | 0 | 0 | 0 | <1 | **2** | **3** | **4** | <1 | 0 | 0 | 0 | 0 | <1 | 0 | 0 | 0 | 0 | 0 | 0 | 0 | 0 | 0 | 0 | 0 | 0 | 0 | 0 | 0 | 0 | 0 | 0 | 0 | 0 | 0 | 0 | 0 | 0 | 0 | 0 | 0 | 0 | 0 | 0 | 0 | 0 |
|  |  | Prop. | 4 | 3 | 11 | 35 | **61** | **76** | **56** | 45 | 13 | 33 | 34 | 4 | 33 | 1 | 0 | 1 | 1 | 1 | 1 | 0 | 0 | 0 | 0 | 0 | 0 | 0 | 0 | 3 | 6 | 1 | 2 | 1 | 2 | 0 | 2 | 1 | 1 | 0 | 0 | 2 | 1 | 1 | 0 | 0 | 1 |
|  | **LUM** | Avg. | 0 | 0 | 0 | 0 | 0 | 0 | 0 | 0 | 0 | 0 | 0 | 0 | 0 | 0 | 0 | 0 | 0 | 0 | 0 | 0 | 0 | 0 | 0 | 0 | 0 | 0 | 0 | 0 | 0 | 0 | 0 | 0 | 0 | 0 | 0 | 0 | 0 | 0 | 0 | **24** | <1 | 0 | 0 | 0 | 0 |
|  |  | Prop. | 0 | 0 | 1 | 1 | 2 | 1 | 0 | 1 | 0 | 1 | 0 | 0 | 0 | 0 | 0 | 0 | 0 | 0 | 1 | 0 | 0 | 0 | 0 | 0 | 0 | 0 | 0 | 2 | 1 | 0 | 1 | 2 | 1 | 0 | 1 | 2 | 2 | 1 | 3 | **88** | 21 | 2 | 1 | 1 | 2 |
|  | ITGB1 | Avg. | 1 | **1** | **1** | <1 | **1** | **1** | **2** | <1 | <1 | <1 | **1** | **1** | <1 | 0 | **1** | **1** | **1** | **1** | **2** | <1 | **1** | <1 | <1 | 0 | **1** | <1 | 0 | <1 | 0 | <1 | **5** | **4** | **4** | **3** | **5** | **4** | **3** | **5** | **6** | **5** | **2** | **2** | **4** | **3** | **5** |
|  |  | Prop. | 24 | 21 | 49 | 43 | **52** | **60** | 33 | **54** | 37 | **56** | **70** | **68** | 42 | 17 | 15 | 29 | 29 | 49 | **53** | 13 | 33 | 12 | 21 | 9 | 44 | 23 | 11 | 37 | 32 | 16 | **78** | **78** | **76** | **84** | **82** | **78** | **69** | **69** | **82** | **84** | **81** | **78** | **59** | **65** | **87** |
|  | **SPP1** | Avg. | 0 | 0 | 0 | <1 | **3** | 0 | 0 | 0 | 0 | 0 | **20** | 0 | 0 | 0 | 0 | 0 | 0 | 0 | 0 | 0 | 0 | 0 | 0 | 0 | 0 | 0 | 0 | 0 | 0 | 0 | 0 | 0 | 0 | 0 | 0 | 0 | 0 | 0 | 0 | 0 | 0 | **3** | **6** | **19** | **15** |
|  |  | Prop. | 2 | 1 | 4 | 8 | 16 | 5 | 1 | 7 | 3 | 10 | **52** | 0 | 8 | 3 | 1 | 3 | 2 | 3 | 3 | 1 | 3 | 1 | 2 | 1 | 5 | 2 | 3 | 10 | 18 | 0 | 4 | 8 | 2 | 3 | 5 | 7 | 9 | 4 | 7 | 8 | 6 | 47 | **57** | **90** | **86** |
|  | CD44 | Avg. | **8** | **5** | **3** | **3** | **2** | **3** | **5** | **3** | **1** | **2** | **3** | <1 | **3** | **1** | **2** | **1** | **1** | **1** | **1** | **3** | **3** | **3** | **3** | **2** | **3** | **2** | <1 | <1 | 0 | **6** | 0 | 0 | 0 | 0 | 0 | 0 | 0 | 0 | <1 | <1 | <1 | 0 | 0 | 0 | 0 |
|  |  | Prop. | **80** | **52** | **76** | **70** | **60** | **79** | **62** | **78** | 43 | **77** | **86** | **57** | **50** | 41 | 17 | 32 | 26 | 40 | 35 | 36 | **61** | 32 | **53** | 48 | **70** | 42 | 26 | 45 | **52** | **67** | 5 | 3 | 4 | 3 | 10 | 3 | 3 | 8 | 20 | 36 | 44 | 9 | 4 | 5 | 18 |
|  | **SPP1** | Avg. | 0 | 0 | 0 | <1 | **3** | 0 | 0 | 0 | 0 | 0 | **20** | 0 | 0 | 0 | 0 | 0 | 0 | 0 | 0 | 0 | 0 | 0 | 0 | 0 | 0 | 0 | 0 | 0 | 0 | 0 | 0 | 0 | 0 | 0 | 0 | 0 | 0 | 0 | 0 | 0 | 0 | **3** | **6** | **19** | **15** |
|  |  | Prop. | 2 | 1 | 4 | 8 | 16 | 5 | 1 | 7 | 3 | 10 | **52** | 0 | 8 | 3 | 1 | 3 | 2 | 3 | 3 | 1 | 3 | 1 | 2 | 1 | 5 | 2 | 3 | 10 | 18 | 0 | 4 | 8 | 2 | 3 | 5 | 7 | 9 | 4 | 7 | 8 | 6 | 47 | **57** | **90** | **86** |
|  | ITGB1 | Avg. | <1 | **1** | **1** | <1 | **1** | **1** | **2** | <1 | <1 | <1 | **1** | **1** | <1 | 0 | **1** | **1** | **1** | **1** | **2** | <1 | **1** | <1 | <1 | 0 | **1** | <1 | 0 | <1 | 0 | <1 | **5** | **4** | **4** | **3** | **5** | **4** | **3** | **5** | **6** | **5** | **2** | **2** | **4** | **3** | **5** |
|  |  | Prop. | 24 | 21 | 49 | 43 | **52** | **60** | 33 | **54** | 37 | **56** | **70** | **68** | 42 | 17 | 15 | 29 | 29 | 49 | **53** | 13 | 33 | 12 | 21 | 9 | 44 | 23 | 11 | 37 | 32 | 16 | **78** | **78** | **76** | **84** | **82** | **78** | **69** | **69** | **82** | **84** | **81** | **78** | **59** | **65** | **87** |
|  | **CD40LG** | Avg. | 0 | 0 | 0 | 0 | 0 | 0 | 0 | 0 | 0 | 0 | 0 | 0 | 0 | 0 | 0 | 0 | 0 | 0 | 0 | **2** | **2** | 0 | 0 | **2** | <1 | 0 | 0 | 0 | 0 | 0 | 0 | 0 | 0 | 0 | 0 | 0 | 0 | 0 | 0 | 0 | 0 | 0 | 0 | 0 | 0 |
|  |  | Prop. | 0 | 1 | 1 | 1 | 2 | 0 | 1 | 2 | 3 | 5 | 0 | 0 | 0 | 2 | 2 | 4 | 1 | 3 | 2 | 25 | 43 | 4 | 10 | **52** | 18 | 2 | 1 | 3 | 13 | 0 | 0 | 1 | 0 | 0 | 1 | 0 | 1 | 1 | 0 | 1 | 1 | 2 | 1 | 0 | 2 |
|  | ITGB2 | Avg. | **5** | **4** | **5** | **5** | **4** | **2** | **2** | **5** | **4** | **5** | **4** | **4** | **1** | **1** | **6** | **5** | **8** | **4** | **7** | **2** | **2** | **4** | **4** | **2** | **4** | **1** | <1 | 0 | 0 | 0 | 0 | 0 | 0 | 0 | 0 | 0 | 0 | 0 | 0 | 0 | 0 | 0 | 0 | 0 | 0 |
|  |  | Prop. | **70** | **54** | **87** | **83** | **82** | **65** | 33 | **91** | **72** | **93** | **98** | **89** | 33 | 44 | **54** | **76** | **81** | **82** | **88** | 31 | **53** | 40 | **68** | **60** | **76** | 30 | 22 | 21 | **51** | 4 | 5 | 7 | 3 | 3 | 6 | 4 | 6 | 2 | 2 | 4 | 7 | 15 | 6 | 2 | 12 |
|  | **CCL19** | Avg. | 0 | 0 | 0 | 0 | 0 | 0 | 0 | 0 | 0 | 0 | 0 | 0 | 0 | 0 | 0 | 0 | 0 | 0 | 0 | 0 | 0 | 0 | 0 | 0 | 0 | 0 | 0 | 0 | 0 | 0 | 0 | 0 | 0 | 0 | 0 | 0 | 0 | 0 | <1 | **13** | 0 | 0 | 0 | 0 | 0 |
|  |  | Prop. | 0 | 0 | 1 | 1 | 6 | 1 | 1 | 6 | 1 | 4 | 2 | 0 | 0 | 0 | 0 | 0 | 0 | 1 | 0 | 0 | 0 | 0 | 0 | 0 | 1 | 0 | 0 | 2 | 3 | 1 | 1 | 4 | 1 | 0 | 1 | 1 | 2 | 1 | 5 | 20 | 6 | 2 | 1 | 1 | 1 |
| **Immune cell signaling (cont.)** | **CXCR3** | Avg. | 0 | 0 | 0 | 0 | 0 | 0 | 0 | 0 | 0 | 0 | 0 | 0 | 0 | **2** | 0 | 0 | 0 | 0 | 0 | 0 | **1** | <1 | **1** | 0 | **2** | 0 | 0 | 0 | 0 | 0 | 0 | 0 | 0 | 0 | 0 | 0 | 0 | 0 | 0 | 0 | 0 | 0 | 0 | 0 | 0 |
|  |  | Prop. | 1 | 1 | 1 | 2 | 3 | 1 | 1 | 7 | 13 | 9 | 7 | 43 | 0 | **60** | 2 | 9 | 6 | 9 | 12 | 8 | 31 | 8 | 30 | 8 | 47 | 14 | 2 | 11 | 23 | 0 | 1 | 2 | 0 | 2 | 1 | 1 | 1 | 0 | 0 | 0 | 1 | 2 | 0 | 0 | 1 |
|  | MFGE8 | Avg. | 0 | 0 | 0 | 0 | 0 | 0 | 0 | 0 | 0 | 0 | 0 | 0 | 0 | 0 | 0 | 0 | 0 | 0 | 0 | 0 | 0 | 0 | 0 | 0 | 0 | 0 | 0 | 0 | 0 | 0 | 0 | 0 | 0 | 0 | 0 | 0 | 0 | **9** | **6** | **1** | 0 | 0 | 0 | 0 | 0 |
|  |  | Prop. | 0 | 1 | 1 | 1 | 2 | 2 | 2 | 3 | 1 | 6 | 7 | 7 | 0 | 2 | 1 | 3 | 3 | 16 | 19 | 1 | 3 | 1 | 2 | 1 | 9 | 1 | 1 | 12 | 6 | 0 | 10 | 18 | 10 | 11 | 10 | 10 | 14 | **82** | **80** | 42 | 31 | 11 | 11 | 8 | 13 |
|  | **PDGFRB** | Avg. | 0 | 0 | 0 | 0 | 0 | 0 | 0 | 0 | 0 | 0 | 0 | 0 | 0 | 0 | 0 | 0 | 0 | 0 | 0 | 0 | 0 | 0 | 0 | 0 | 0 | 0 | 0 | 0 | 0 | 0 | 0 | 0 | 0 | 0 | 0 | 0 | 0 | **5** | **4** | **2** | 0 | 0 | 0 | 0 | 0 |
|  |  | Prop. | 0 | 0 | 1 | 0 | 0 | 0 | 0 | 1 | 0 | 2 | 2 | 0 | 0 | 0 | 0 | 0 | 5 | 0 | 9 | 0 | 0 | 0 | 1 | 0 | 0 | 0 | 1 | 5 | 1 | 0 | 1 | 0 | 1 | 1 | 1 | 0 | 1 | **62** | **73** | 48 | 6 | 3 | 3 | 1 | 1 |
|  | **VIM** | Avg. | **18** | **9** | **10** | **11** | **11** | **10** | **15** | **18** | **14** | **23** | **20** | **25** | **12** | **11** | **1** | **3** | **3** | **9** | **11** | **6** | **11** | **4** | **9** | **8** | **15** | **5** | **3** | **6** | **3** | **17** | **3** | **58** | **37** | **8** | **50** | **31** | **48** | **30** | **29** | **35** | **15** | **1** | **4** | **3** | **6** |
|  |  | Prop. | **93** | **68** | **96** | **93** | **91** | **98** | **87** | **98** | **87** | **99** | **100** | **100** | **92** | **96** | 15 | 48 | 47 | **85** | **91** | **54** | **90** | 37 | **85** | **88** | **91** | **72** | **53** | **93** | **91** | **87** | 41 | **100** | **98** | **84** | **99** | **98** | **100** | **95** | **97** | **100** | **97** | **59** | **57** | **63** | **84** |
|  | CD44 | Avg. | **8** | **5** | **3** | **3** | **2** | **3** | **5** | **3** | **1** | **2** | **3** | <1 | **3** | **1** | **2** | **1** | **1** | **1** | **1** | **3** | **3** | **3** | **3** | **2** | **3** | **2** | <1 | <1 | 0 | **6** | 0 | 0 | 0 | 0 | 0 | 0 | 0 | 0 | <1 | <1 | <1 | 0 | 0 | 0 | 0 |
|  |  | Prop. | **80** | **52** | **76** | **70** | **60** | **79** | **62** | **78** | 43 | **77** | **86** | **57** | **50** | 41 | 17 | 32 | 26 | 40 | 35 | 36 | **61** | 32 | **53** | 48 | **70** | 42 | 26 | 45 | **52** | **67** | 5 | 3 | 4 | 3 | 10 | 3 | 3 | 8 | 20 | 36 | 44 | 9 | 4 | 5 | 18 |
|  | HLA-A | Avg. | **14** | **13** | **20** | **18** | **23** | **21** | **15** | **18** | **15** | **16** | **18** | **20** | **14** | **14** | **33** | **39** | **47** | **27** | **39** | **29** | **32** | **39** | **45** | **38** | **35** | **23** | **18** | **8** | **7** | **13** | **32** | **23** | **25** | **33** | **33** | **33** | **27** | **5** | **8** | **12** | **8** | **9** | **10** | **14** | **15** |
|  |  | Prop. | **90** | **82** | **99** | **97** | **99** | **100** | **90** | **99** | **94** | **99** | **100** | **100** | **100** | **97** | **96** | **100** | **99** | **99** | **99** | **95** | **100** | **97** | **100** | **100** | **100** | **98** | **96** | **95** | **96** | **80** | **99** | **100** | **100** | **100** | **100** | **99** | **99** | **69** | **89** | **94** | **96** | **96** | **80** | **95** | **98** |
|  | **CD3D** | Avg. | 0 | <1 | 0 | 0 | 0 | 0 | <1 | 0 | <1 | 0 | 0 | 0 | <1 | 0 | **1** | <1 | <1 | 0 | 0 | **8** | **8** | **10** | **10** | **9** | **10** | <1 | 0 | 0 | <1 | 0 | 0 | 0 | 0 | 0 | 0 | 0 | 0 | 0 | 0 | 0 | 0 | 0 | 0 | 0 | 0 |
|  |  | Prop. | 10 | 13 | 17 | 14 | 17 | 8 | 13 | 18 | 20 | 26 | 14 | 46 | 33 | 18 | 12 | 13 | 12 | 19 | 19 | **72** | **88** | **67** | **92** | **94** | **91** | 19 | 9 | 19 | **54** | 10 | 4 | 6 | 5 | 2 | 5 | 3 | 5 | 4 | 4 | 7 | 13 | 18 | 9 | 5 | 11 |
|  | HLA-B | Avg. | **23** | **26** | **42** | **35** | **42** | **47** | **33** | **33** | **33** | **30** | **41** | **43** | **30** | **39** | **47** | **61** | **71** | **44** | **64** | **45** | **52** | **53** | **71** | **73** | **50** | **44** | **33** | **22** | **25** | **17** | **51** | **23** | **46** | **60** | **64** | **56** | **40** | **7** | **12** | **14** | **14** | **10** | **12** | **10** | **17** |
|  |  | Prop. | **96** | **97** | **100** | **100** | **100** | **100** | **97** | **100** | **98** | **100** | **100** | **100** | **100** | **99** | **98** | **100** | **100** | **100** | **100** | **99** | **100** | **99** | **100** | **100** | **100** | **99** | **99** | **99** | **100** | **91** | **100** | **99** | **100** | **100** | **100** | **100** | **100** | **74** | **93** | **97** | **99** | **97** | **86** | **91** | **98** |
|  | **CD3D** | Avg. | 0 | <1 | 0 | 0 | 0 | 0 | <1 | 0 | <1 | 0 | 0 | 0 | <1 | 0 | **1** | <1 | <1 | 0 | 0 | **8** | **8** | **10** | **10** | **9** | **10** | <1 | 0 | 0 | <1 | 0 | 0 | 0 | 0 | 0 | 0 | 0 | 0 | 0 | 0 | 0 | 0 | 0 | 0 | 0 | 0 |
|  |  | Prop. | 10 | 13 | 17 | 14 | 17 | 8 | 13 | 18 | 20 | 26 | 14 | 46 | 33 | 18 | 12 | 13 | 12 | 19 | 19 | **72** | **88** | **67** | **92** | **94** | **91** | 19 | 9 | 19 | **54** | 10 | 4 | 6 | 5 | 2 | 5 | 3 | 5 | 4 | 4 | 7 | 13 | 18 | 9 | 5 | 11 |
|  | B2M | Avg. | **114** | **108** | **166** | **139** | **173** | **249** | **200** | **125** | **108** | **120** | **133** | **99** | **264** | **127** | **161** | **167** | **243** | **137** | **200** | **184** | **202** | **213** | **234** | **204** | **181** | **135** | **95** | **90** | **77** | **139** | **175** | **87** | **157** | **190** | **176** | **156** | **123** | **42** | **58** | **63** | **51** | **35** | **40** | **40** | **50** |
|  |  | Prop. | **100** | **100** | **100** | **100** | **100** | **100** | **100** | **100** | **100** | **100** | **100** | **100** | **100** | **100** | **100** | **100** | **100** | **100** | **100** | **100** | **100** | **100** | **100** | **100** | **100** | **100** | **100** | **100** | **100** | **100** | **100** | **100** | **100** | **100** | **100** | **100** | **100** | **99** | **100** | **100** | **100** | **100** | **99** | **100** | **100** |
|  | **CD3D** | Avg. | 0 | <1 | 0 | 0 | 0 | 0 | <1 | 0 | <1 | 0 | 0 | 0 | <1 | 0 | **1** | <1 | <1 | 0 | 0 | **8** | **8** | **10** | **10** | **9** | **10** | <1 | 0 | 0 | <1 | 0 | 0 | 0 | 0 | 0 | 0 | 0 | 0 | 0 | 0 | 0 | 0 | 0 | 0 | 0 | 0 |
|  |  | Prop. | 10 | 13 | 17 | 14 | 17 | 8 | 13 | 18 | 20 | 26 | 14 | 46 | 33 | 18 | 12 | 13 | 12 | 19 | 19 | **72** | **88** | **67** | **92** | **94** | **91** | 19 | 9 | 19 | **54** | 10 | 4 | 6 | 5 | 2 | 5 | 3 | 5 | 4 | 4 | 7 | 13 | 18 | 9 | 5 | 11 |
| **ECM Interactions** | **MMP7** | Avg. | 0 | 0 | 0 | 0 | 0 | 0 | 0 | 0 | 0 | 0 | 0 | 0 | 0 | 0 | 0 | 0 | 0 | 0 | 0 | 0 | 0 | 0 | 0 | 0 | 0 | 0 | 0 | 0 | 0 | 0 | 0 | 0 | 0 | 0 | 0 | 0 | 0 | 0 | 0 | 0 | 0 | 0 | **2** | **5** | **7** |
|  |  | Prop. | 0 | 0 | 1 | 1 | 2 | 1 | 0 | 1 | 1 | 1 | 7 | 0 | 0 | 1 | 0 | 1 | 1 | 1 | 2 | 0 | 1 | 0 | 0 | 0 | 2 | 1 | 0 | 1 | 3 | 0 | 1 | 2 | 0 | 0 | 2 | 2 | 2 | 1 | 2 | 5 | 6 | 15 | 36 | **70** | **59** |
|  | **CDH6** | Avg. | 0 | 0 | 0 | 0 | 0 | 0 | 0 | 0 | 0 | 0 | 0 | 0 | 0 | 0 | 0 | 0 | 0 | 0 | 0 | 0 | 0 | 0 | 0 | 0 | 0 | 0 | 0 | 0 | 0 | 0 | 0 | 0 | 0 | 0 | 0 | 0 | 0 | **2** | <1 | 0 | 0 | 0 | <1 | 0 | <1 |
|  |  | Prop. | 0 | 0 | 0 | 0 | 0 | 0 | 0 | 0 | 0 | 0 | 2 | 0 | 0 | 0 | 0 | 0 | 0 | 0 | 0 | 0 | 0 | 0 | 0 | 0 | 0 | 0 | 0 | 1 | 0 | 0 | 0 | 1 | 0 | 0 | 1 | 0 | 0 | 35 | 28 | 8 | 2 | 19 | 20 | 16 | 38 |
| **Fibrosis** | **PDGFD** | Avg. | 0 | 0 | 0 | 0 | 0 | 0 | 0 | 0 | 0 | 0 | 0 | 0 | 0 | 0 | <1 | 0 | 0 | 0 | 0 | 0 | 0 | 0 | 0 | 0 | 0 | 0 | 0 | 0 | 0 | 0 | 0 | 0 | 0 | 0 | <1 | <1 | 0 | 0 | 0 | 0 | 0 | 0 | **1** | <1 | **1** |
|  |  | Prop. | 0 | 0 | 0 | 1 | 1 | 1 | 1 | 1 | 0 | 2 | 2 | 0 | 8 | 0 | 6 | 13 | 12 | 12 | 14 | 1 | 1 | 2 | 5 | 2 | 1 | 0 | 1 | 3 | 4 | 0 | 2 | 5 | 1 | 1 | 27 | 23 | 4 | 0 | 1 | 18 | 36 | 9 | 28 | 40 | 45 |
|  | **PDGFRA** | Avg. | 0 | 0 | 0 | 0 | 0 | 0 | 0 | 0 | 0 | 0 | 0 | 0 | 0 | 0 | 0 | 0 | 0 | 0 | 0 | 0 | 0 | 0 | 0 | 0 | 0 | 0 | 0 | 0 | 0 | 0 | 0 | 0 | 0 | 0 | 0 | 0 | 0 | 0 | 0 | **2** | <1 | 0 | 0 | 0 | 0 |
|  |  | Prop. | 0 | 0 | 0 | 0 | 0 | 0 | 0 | 0 | 0 | 0 | 0 | 0 | 0 | 0 | 0 | 0 | 0 | 0 | 0 | 0 | 0 | 0 | 0 | 0 | 0 | 0 | 0 | 0 | 0 | 0 | 1 | 1 | 2 | 0 | 1 | 1 | 2 | 0 | 0 | **60** | **58** | 4 | 0 | 0 | 1 |

Ligands and receptors with an average expression (Avg) greater than 1 and proportional expression (Prop) in more than 50% of cells were used as cutoff criteria. Interactions between ligands and receptors within the same cell type were highlighted (boxed), as well as potential cell-to-cell communications where both average and proportional expression met the cutoff thresholds. Bolded genes represent DEGs identified by the meta-analysis.

**References**

1.	Love MI, Huber W, Anders S. Moderated estimation of fold change and dispersion for RNA-seq data with DESeq2. Genome Biol 2014;15:550.

2.	Edgington ES. An Additive Method for Combining Probability Values from Independent Experiments. The Journal of Psychology 1972;80:351–363.

3.	Szklarczyk D, Kirsch R, Koutrouli M, Nastou K, Mehryary F, Hachilif R, Gable AL, et al. The STRING database in 2023: protein-protein association networks and functional enrichment analyses for any sequenced genome of interest. Nucleic Acids Res 2023;51:D638–D646.

4.	Sanchez JI, Parra ER, Jiao J, Solis Soto LM, Ledesma DA, Saldarriaga OA, Stevenson HL, et al. Cellular and Molecular Mechanisms of Liver Fibrosis in Patients with NAFLD. Cancers (Basel) 2023;15.

5.	Yao K, Tarabra E, Sia D, Morotti R, Fawaz R, Valentino P, Santoro N, et al. Transcriptomic profiling of a multiethnic pediatric NAFLD cohort reveals genes and pathways associated with disease. Hepatol Commun 2022;6:1598–1610.

6.	Takeda U, Utani A, Wu J, Adachi E, Koseki H, Taniguchi M, Matsumoto T, et al. Targeted disruption of dermatopontin causes abnormal collagen fibrillogenesis. J Invest Dermatol 2002;119:678–683.

7.	Li Y, Yuan SL, Yin JY, Yang K, Zhou XG, Xie W, Wang Q. Differences of core genes in liver fibrosis and hepatocellular carcinoma: Evidence from integrated bioinformatics and immunohistochemical analysis. World J Gastrointest Oncol 2022;14:1265–1280.

8.	Moylan CA, Pang H, Dellinger A, Suzuki A, Garrett ME, Guy CD, Murphy SK, et al. Hepatic gene expression profiles differentiate presymptomatic patients with mild versus severe nonalcoholic fatty liver disease. Hepatology 2014;59:471–482.

9.	He L, Yuan H, Liang J, Hong J, Qu C. Expression of hepatic stellate cell activation-related genes in HBV-, HCV-, and nonalcoholic fatty liver disease-associated fibrosis. PLoS One 2020;15:e0233702.

10.	Li P, Xie W, Wei H, Yang F, Chen Y, Li Y. Transcriptome Analyses of Liver Sinusoidal Endothelial Cells Reveal a Consistent List of Candidate Genes Associated with Endothelial Dysfunction and the Fibrosis Progression. Curr Issues Mol Biol 2024;46:7997–8014.

11.	Lai M, Dillon ST, Gu X, Morhardt TL, Xu Y, Chan NY, Xiong B, et al. Serum protein risk stratification score for diagnostic evaluation of metabolic dysfunction-associated steatohepatitis. Hepatol Commun 2024;8.

12.	Wang Y, Bo J, Zhao Z, Han Y, Zhang Q, Liu L. Depletion of Igfbp7 alleviates zebrafish NAFLD progression through inhibiting hepatic ferroptosis. Life Sci 2023;332:122086.

13.	Zhang H, Ma Y, Cheng X, Wu D, Huang X, Chen B, Ren Y, et al. Targeting epigenetically maladapted vascular niche alleviates liver fibrosis in nonalcoholic steatohepatitis. Sci Transl Med 2021;13:eabd1206.

14.	Liu LX, Huang S, Zhang QQ, Liu Y, Zhang DM, Guo XH, Han DW. Insulin-like growth factor binding protein-7 induces activation and transdifferentiation of hepatic stellate cells in vitro. World J Gastroenterol 2009;15:3246–3253.

15.	Yokomori H, Oda M, Yoshimura K, Kaneko F, Hibi T. Aquaporin-1 associated with hepatic arterial capillary proliferation on hepatic sinusoid in human cirrhotic liver. Liver Int 2011;31:1554–1564.

16.	Pelagalli A, Nardelli A, Fontanella R, Zannetti A. Inhibition of AQP1 Hampers Osteosarcoma and Hepatocellular Carcinoma Progression Mediated by Bone Marrow-Derived Mesenchymal Stem Cells. Int J Mol Sci 2016;17.

17.	Zhao D, Yu S, Guo P, Zhang X, Tang Y, Dong C, Zhao S, et al. Identification of potential plasma markers for hepatitis B virus-related chronic hepatitis and liver fibrosis/cirrhosis. J Med Virol 2022;94:3900–3910.

18.	Huebert RC, Jagavelu K, Hendrickson HI, Vasdev MM, Arab JP, Splinter PL, Trussoni CE, et al. Aquaporin-1 promotes angiogenesis, fibrosis, and portal hypertension through mechanisms dependent on osmotically sensitive microRNAs. Am J Pathol 2011;179:1851–1860.

19.	Ciurea AM, Vere CC, Popp CG, Streba CT, Calita M, Pirici D, Cercelaru L, et al. E-cadherin and aquaporin 1 co-expression analysis in hepatocellular carcinoma: a pilot study. Rom J Morphol Embryol 2021;62:427–434.

20.	Naito T, Saheki Y. GRAMD1-mediated accessible cholesterol sensing and transport. Biochim Biophys Acta Mol Cell Biol Lipids 2021;1866:158957.

21.	Yang G, Liu R, Rezaei S, Liu X, Wan Y-JY. Machine Learning to Identify Molecular Markers for Metabolic Disease Development Using Mouse Models. bioRxiv 2023:2023.2003.2011.532149.

22.	Wang X, Cai B, Yang X, Sonubi OO, Zheng Z, Ramakrishnan R, Shi H, et al. Cholesterol Stabilizes TAZ in Hepatocytes to Promote Experimental Non-alcoholic Steatohepatitis. Cell Metab 2020;31:969–986 e967.

23.	Feng R, Du W, Lui P, Zhang J, Liu Y. CAPN2 acts as an indicator of hepatitis B virus to induce hepatic fibrosis. J Cell Biochem 2020;121:2428–2436.

24.	Ma XL, Zhu KY, Chen YD, Tang WG, Xie SH, Zheng H, Tong Y, et al. Identification of a novel Calpain-2-SRC feed-back loop as necessity for beta-Catenin accumulation and signaling activation in hepatocellular carcinoma. Oncogene 2022;41:3554–3569.

25.	Starmann J, Falth M, Spindelbock W, Lanz KL, Lackner C, Zatloukal K, Trauner M, et al. Gene expression profiling unravels cancer-related hepatic molecular signatures in steatohepatitis but not in steatosis. PLoS One 2012;7:e46584.

26.	Zhao L, Pei R, Ding Y, Su Z, Li D, Zhu S, Xu L, et al. LOXL4 Shuttled by Tumor Cells-derived Extracellular Vesicles Promotes Immune Escape in Hepatocellular Carcinoma by Activating the STAT1/PD-L1 Axis. J Immunother 2024;47:64–76.

27.	Li R, Wang Y, Zhang X, Feng M, Ma J, Li J, Yang X, et al. Exosome-mediated secretion of LOXL4 promotes hepatocellular carcinoma cell invasion and metastasis. Mol Cancer 2019;18:18.

28.	Vadasz Z, Kessler O, Akiri G, Gengrinovitch S, Kagan HM, Baruch Y, Izhak OB, et al. Abnormal deposition of collagen around hepatocytes in Wilson's disease is associated with hepatocyte specific expression of lysyl oxidase and lysyl oxidase like protein-2. J Hepatol 2005;43:499–507.

29.	Wang Z, Zhao Z, Xia Y, Cai Z, Wang C, Shen Y, Liu R, et al. Potential biomarkers in the fibrosis progression of nonalcoholic steatohepatitis (NASH). J Endocrinol Invest 2022;45:1379–1392.

30.	Feng J, Wei T, Cui X, Wei R, Hong T. Identification of key genes and pathways in mild and severe nonalcoholic fatty liver disease by integrative analysis. Chronic Dis Transl Med 2021;7:276–286.

31.	Athwal VS, Pritchett J, Martin K, Llewellyn J, Scott J, Harvey E, Zaitoun AM, et al. SOX9 regulated matrix proteins are increased in patients serum and correlate with severity of liver fibrosis. Sci Rep 2018;8:17905.

32.	Wu S, Li J, Zhan Y. H3K18 lactylation accelerates liver fibrosis progression through facilitating SOX9 transcription. Exp Cell Res 2024;440:114135.

33.	Zhu C, Kim K, Wang X, Bartolome A, Salomao M, Dongiovanni P, Meroni M, et al. Hepatocyte Notch activation induces liver fibrosis in nonalcoholic steatohepatitis. Sci Transl Med 2018;10.

34.	Wang C, Deng J, Deng H, Kang Z, Huang Z, Ding Z, Dong L, et al. A Novel Sox9/lncRNA H19 Axis Contributes to Hepatocyte Death and Liver Fibrosis. Toxicol Sci 2020;177:214–225.

35.	Athwal VS, Pritchett J, Llewellyn J, Martin K, Camacho E, Raza SM, Phythian-Adams A, et al. SOX9 predicts progression toward cirrhosis in patients while its loss protects against liver fibrosis. EMBO Mol Med 2017;9:1696–1710.

36.	El-Araby HA, Saber MA, Radwan NM, Taie DM, Adawy NM, Sira AM. SOX9 in biliary atresia: New insight for fibrosis progression. Hepatobiliary Pancreat Dis Int 2021;20:154–162.

37.	Bonacchi A, Petrai I, Defranco RM, Lazzeri E, Annunziato F, Efsen E, Cosmi L, et al. The chemokine CCL21 modulates lymphocyte recruitment and fibrosis in chronic hepatitis C. Gastroenterology 2003;125:1060–1076.

38.	Grant AJ, Goddard S, Ahmed-Choudhury J, Reynolds G, Jackson DG, Briskin M, Wu L, et al. Hepatic expression of secondary lymphoid chemokine (CCL21) promotes the development of portal-associated lymphoid tissue in chronic inflammatory liver disease. Am J Pathol 2002;160:1445–1455.

39.	Aass KR, Kastnes MH, Standal T. Molecular interactions and functions of IL-32. J Leukoc Biol 2021;109:143–159.

40.	Xu X, Dong Y, Liu J, Zhang P, Yang W, Dai L. Regulation of Inflammation, Lipid Metabolism, and Liver Fibrosis by Core Genes of M1 Macrophages in NASH. J Inflamm Res 2024;17:9975–9986.

41.	Baselli GA, Dongiovanni P, Rametta R, Meroni M, Pelusi S, Maggioni M, Badiali S, et al. Liver transcriptomics highlights interleukin-32 as novel NAFLD-related cytokine and candidate biomarker. Gut 2020;69:1855–1866.

42.	Schilcher K, Dayoub R, Kubitza M, Riepl J, Klein K, Buechler C, Melter M, et al. Saturated Fat-Mediated Upregulation of IL-32 and CCL20 in Hepatocytes Contributes to Higher Expression of These Fibrosis-Driving Molecules in MASLD. Int J Mol Sci 2023;24.

43.	Dali-Youcef N, Vix M, Costantino F, El-Saghire H, Lhermitte B, Callari C, D'Agostino J, et al. Interleukin-32 Contributes to Human Nonalcoholic Fatty Liver Disease and Insulin Resistance. Hepatol Commun 2019;3:1205–1220.

44.	Sasidharan K, Caddeo A, Jamialahmadi O, Noto FR, Tomasi M, Malvestiti F, Ciociola E, et al. IL32 downregulation lowers triglycerides and type I collagen in di-lineage human primary liver organoids. Cell Rep Med 2024;5:101352.

45.	Zhao D, Zhang X, Tang Y, Guo P, Ai R, Hou M, Wang Y, et al. Identification and Validation of Novel Biomarkers for Hepatocellular Carcinoma, Liver Fibrosis/Cirrhosis and Chronic Hepatitis B via Transcriptome Sequencing Technology. J Hepatocell Carcinoma 2022;9:389–403.

46.	Xu Q, Pan X, Shu X, Cao H, Li X, Zhang K, Lu J, et al. Increased interleukin-32 expression in chronic hepatitis B virus-infected liver. J Infect 2012;65:336–342.

47.	Chu X, Jin Q, Chen H, Wood GC, Petrick A, Strodel W, Gabrielsen J, et al. CCL20 is up-regulated in non-alcoholic fatty liver disease fibrosis and is produced by hepatic stellate cells in response to fatty acid loading. J Transl Med 2018;16:108.

48.	Hanson A, Piras IS, Wilhelmsen D, Still CD, Chu X, Petrick A, Gerhard GS, et al. Chemokine ligand 20 (CCL20) expression increases with NAFLD stage and hepatic stellate cell activation and is regulated by miR-590-5p. Cytokine 2019;123:154789.

49.	He W, Huang C, Zhang X, Wang D, Chen Y, Zhao Y, Li X. Identification of transcriptomic signatures and crucial pathways involved in non-alcoholic steatohepatitis. Endocrine 2021;73:52–64.

50.	Heo YJ, Choi SE, Lee N, Jeon JY, Han SJ, Kim DJ, Kang Y, et al. CCL20 induced by visfatin in macrophages via the NF-kappaB and MKK3/6-p38 signaling pathways contributes to hepatic stellate cell activation. Mol Biol Rep 2020;47:4285–4293.

51.	Song J, Liu L, Wang Z, Xie D, Azami NLB, Lu L, Huang Y, et al. CCL20 and CD8A as potential diagnostic biomarkers for HBV-induced liver fibrosis in chronic hepatitis B. Heliyon 2024;10:e28329.

52.	Liu QQ, Chen J, Ma T, Huang W, Lu CH. DCDC2 inhibits hepatic stellate cell activation and ameliorates CCl(4)-induced liver fibrosis by suppressing Wnt/beta-catenin signaling. Sci Rep 2024;14:9425.

53.	Syryn H, Hoorens A, Grammatikopoulos T, Deheragoda M, Symoens S, Vande Velde S, Van Biervliet S, et al. Two cases of DCDC2-related neonatal sclerosing cholangitis with developmental delay and literature review. Clin Genet 2021;100:447–452.

54.	Azabdaftari A, Sczakiel HL, Danyel M, Kohlmaier B, Mache CJ, Stalke A, Pfister ED, et al. Biallelic known and novel DCDC2 variants in cholestatic liver disease: Phenotype-genotype observations in four children. Liver Int 2023;43:1089–1095.

55.	Kaur P, Lal BB, Janakiraman D, Dheivamani N, Pandey S, Bavdekar A, Shah A, et al. Portal hypertension in doublecortin domain-containing protein 2 (DCDC2)-related neonatal sclerosing cholangitis. J Pediatr Gastroenterol Nutr 2025;80:189–196.

56.	Kozumi K, Kodama T, Murai H, Sakane S, Govaere O, Cockell S, Motooka D, et al. Transcriptomics Identify Thrombospondin-2 as a Biomarker for NASH and Advanced Liver Fibrosis. Hepatology 2021;74:2452–2466.

57.	Kimura T, Iwadare T, Wakabayashi SI, Kuldeep S, Nakajima T, Yamazaki T, Aomura D, et al. Thrombospondin 2 is a key determinant of fibrogenesis in non-alcoholic fatty liver disease. Liver Int 2024;44:483–496.

58.	Pantano L, Agyapong G, Shen Y, Zhuo Z, Fernandez-Albert F, Rust W, Knebel D, et al. Molecular characterization and cell type composition deconvolution of fibrosis in NAFLD. Sci Rep 2021;11:18045.

59.	Mahmoudi A, Butler AE, De Vincentis A, Jamialahmadi T, Sahebkar A. Microarray-based Detection of Critical Overexpressed Genes in the Progression of Hepatic Fibrosis in Non-alcoholic Fatty Liver Disease: A Protein-protein Interaction Network Analysis. Curr Med Chem 2024;31:3631–3652.

60.	Jerala M, Hauptman N, Kojc N, Zidar N. Expression of Fibrosis-Related Genes in Liver and Kidney Fibrosis in Comparison to Inflammatory Bowel Diseases. Cells 2022;11.

61.	Lou Y, Tian GY, Song Y, Liu YL, Chen YD, Shi JP, Yang J. Characterization of transcriptional modules related to fibrosing-NAFLD progression. Sci Rep 2017;7:4748.

62.	Li JZ, Yang L, Xiao MX, Li N, Huang X, Ye LH, Zhang HC, et al. Spatial and Single-Cell Transcriptomics Reveals the Regional Division of the Spatial Structure of MASH Fibrosis. Liver Int 2025;45:e16125.

63.	Zhang N, Wu X, Zhang W, Sun Y, Yan X, Xu A, Han Q, et al. Targeting thrombospondin-2 retards liver fibrosis by inhibiting TLR4-FAK/TGF-beta signaling. JHEP Rep 2024;6:101014.

64.	Iwadare T, Kimura T, Tanaka N, Yamazaki T, Wakabayashi SI, Okumura T, Kobayashi H, et al. Circulating thrombospondin 2 levels reflect fibrosis severity and disease activity in HCV-infected patients. Sci Rep 2022;12:18900.

65.	Gross S, Danielyan L, Buechler C, Kubitza M, Klein K, Schwab M, Melter M, et al. Hepatic Amyloid Beta-42-Metabolizing Proteins in Liver Steatosis and Metabolic Dysfunction-Associated Steatohepatitis. Int J Mol Sci 2024;25.

66.	Yang X, Cheng QN, Wu JF, Ai WB, Ma L. Analysis of key genes and related transcription factors in liver fibrosis based on bioinformatic technology. Int J Clin Exp Pathol 2021;14:444–454.

67.	Bracht T, Schweinsberg V, Trippler M, Kohl M, Ahrens M, Padden J, Naboulsi W, et al. Analysis of disease-associated protein expression using quantitative proteomics-fibulin-5 is expressed in association with hepatic fibrosis. J Proteome Res 2015;14:2278–2286.

68.	Corey KE, Pitts R, Lai M, Loureiro J, Masia R, Osganian SA, Gustafson JL, et al. ADAMTSL2 protein and a soluble biomarker signature identify at-risk non-alcoholic steatohepatitis and fibrosis in adults with NAFLD. J Hepatol 2022;76:25–33.

69.	Cai X, Li Z, Zhang Q, Qu Y, Xu M, Wan X, Lu L. CXCL6-EGFR-induced Kupffer cells secrete TGF-beta1 promoting hepatic stellate cell activation via the SMAD2/BRD4/C-MYC/EZH2 pathway in liver fibrosis. J Cell Mol Med 2018;22:5050–5061.

70.	Wu C, Cheng D, Peng Y, Li Y, Fu C, Wang Y, Fu L, et al. Hepatic BRD4 Is Upregulated in Liver Fibrosis of Various Etiologies and Positively Correlated to Fibrotic Severity. Front Med (Lausanne) 2021;8:683506.

71.	Govaere O, Cockell S, Van Haele M, Wouters J, Van Delm W, Van den Eynde K, Bianchi A, et al. High-throughput sequencing identifies aetiology-dependent differences in ductular reaction in human chronic liver disease. J Pathol 2019;248:66–76.

72.	Zhang J, Luo Y, Feng M, Xia Q. Identification of Liver Immune Microenvironment-Related Hub Genes in Liver of Biliary Atresia. Front Pediatr 2021;9:786422.

73.	Hur YK, Lee HE, Yoo JY, Park YN, Lee IH, Bae YS. NADPH oxidase 4-SH3 domain-containing YSC84-like 1 complex participates liver inflammation and fibrosis. Free Radic Biol Med 2025;227:246–259.

74.	Charlton M, Viker K, Krishnan A, Sanderson S, Veldt B, Kaalsbeek AJ, Kendrick M, et al. Differential expression of lumican and fatty acid binding protein-1: new insights into the histologic spectrum of nonalcoholic fatty liver disease. Hepatology 2009;49:1375–1384.

75.	Lee YT, Sun N, Kim M, Wang JJ, Tran BV, Zhang RY, Qi D, et al. Circulating Tumor Cell-Based Messenger RNA Scoring System for Prognostication of Hepatocellular Carcinoma: Translating Tissue-Based Messenger RNA Profiling Into a Noninvasive Setting. Liver Transpl 2022;28:200–214.

76.	Si T, Ning X, Chen H, Hu Z, Dun L, Zheng N, Huang P, et al. ANTXR1 as a potential prognostic biomarker for hepatitis B virus-related hepatocellular carcinoma identified by a weighted gene correlation network analysis. J Gastrointest Oncol 2021;12:3079–3092.

77.	Fu J, Chen F, Lin Y, Gao J, Chen A, Yang J. Discovery and characterization of tumor antigens in hepatocellular carcinoma for mRNA vaccine development. J Cancer Res Clin Oncol 2023;149:4047–4061.

78.	Zheng H, Yang Y, Han J, Jiang WH, Chen C, Wang MC, Gao R, et al. TMED3 promotes hepatocellular carcinoma progression via IL-11/STAT3 signaling. Sci Rep 2016;6:37070.

79.	Chen W, Zhao W, Yang A, Xu A, Wang H, Cong M, Liu T, et al. Integrated analysis of microRNA and gene expression profiles reveals a functional regulatory module associated with liver fibrosis. Gene 2017;636:87–95.

80.	Qian Y, Wang JW, Fang Y, Yuan XD, Fan YC, Gao S, Wang K. Measurement of Cyclin D2 (CCND2) Gene Promoter Methylation in Plasma and Peripheral Blood Mononuclear Cells and Alpha-Fetoprotein Levels in Patients with Hepatitis B Virus-Associated Hepatocellular Carcinoma. Med Sci Monit 2020;26:e927444.

81.	Sheng ZG, Chen MH. TPM4 aggravates the malignant progression of hepatocellular carcinoma through negatively regulating SUSD2. Eur Rev Med Pharmacol Sci 2020;24:4756–4765.

82.	He H, Dai F, Yu L, She X, Zhao Y, Jiang J, Chen X, et al. Identification and characterization of nine novel human small GTPases showing variable expressions in liver cancer tissues. Gene Expr 2002;10:231–242.

83.	Geng D, Zhao W, Feng Y, Liu J. Overexpression of Rab25 promotes hepatocellular carcinoma cell proliferation and invasion. Tumour Biol 2016;37:7713–7718.

84.	Hou W, Janech MG, Sobolesky PM, Bland AM, Samsuddin S, Alazawi W, Syn WK. Proteomic screening of plasma identifies potential noninvasive biomarkers associated with significant/advanced fibrosis in patients with nonalcoholic fatty liver disease. Biosci Rep 2020;40.

85.	Shi Y, Dong H, Sun S, Wu X, Fang J, Zhao J, Han J, et al. Protein-centric omics analysis reveals circulating complements linked to non-viral liver diseases as potential therapeutic targets. Clin Mol Hepatol 2024;30:80–97.

86.	Zertuche-Martinez C, Velazquez-Enriquez JM, Gonzalez-Garcia K, Santos-Alvarez JC, Romero-Tlalolini MLA, Pina-Canseco S, Perez-Campos Mayoral L, et al. Discovery of candidate biomarkers from plasma-derived extracellular vesicles of patients with cirrhosis and hepatocellular carcinoma: an exploratory proteomic study. Mol Omics 2024;20:483–495.

87.	de Lima RE, de Holanda Martins CM, do Carmo RF, Aroucha D, Pereira L, Vasconcelos LRS, Moura P. Two sides of a coin: GG genotype of C7 provides protection against fibrosis severity while showing a higher risk for hepatocellular carcinoma in patients with hepatitis C. Hum Immunol 2018;79:702–707.

88.	Lee CF, Ling ZQ, Zhao T, Lee KR. Distinct expression patterns in hepatitis B virus- and hepatitis C virus-infected hepatocellular carcinoma. World J Gastroenterol 2008;14:6072–6077.

89.	Peters V, Jansen EE, Jakobs C, Riedl E, Janssen B, Yard BA, Wedel J, et al. Anserine inhibits carnosine degradation but in human serum carnosinase (CN1) is not correlated with histidine dipeptide concentration. Clin Chim Acta 2011;412:263–267.

90.	Huang XW, Li Y, Jiang LN, Zhao BK, Liu YS, Chen C, Zhao D, et al. Nomogram for preoperative estimation of microvascular invasion risk in hepatocellular carcinoma. Transl Oncol 2024;45:101986.

91.	Parcerisas A, Pujadas L, Ortega-Gasco A, Perello-Amoros B, Viais R, Hino K, Figueiro-Silva J, et al. NCAM2 Regulates Dendritic and Axonal Differentiation through the Cytoskeletal Proteins MAP2 and 14-3-3. Cereb Cortex 2020;30:3781–3799.

92.	Takahashi S, Kato K, Nakamura K, Nakano R, Kubota K, Hamada H. Neural cell adhesion molecule 2 as a target molecule for prostate and breast cancer gene therapy. Cancer Sci 2011;102:808–814.

93.	Okuno E, Tsujimoto M, Nakamura M, Kido R. 2-Aminoadipate-2-oxoglutarate aminotransferase isoenzymes in human liver: a plausible physiological role in lysine and tryptophan metabolism. Enzyme Protein 1993;47:136–148.

94.	Wolin SL, Cedervall T. The La protein. Annu Rev Biochem 2002;71:375–403.

95.	Li L, Li H, Garzel B, Yang H, Sueyoshi T, Li Q, Shu Y, et al. SLC13A5 is a novel transcriptional target of the pregnane X receptor and sensitizes drug-induced steatosis in human liver. Mol Pharmacol 2015;87:674–682.

96.	Sun Q, Niu Q, Guo Y, Zhuang Y, Li X, Liu J, Li N, et al. Regulation on Citrate Influx and Metabolism through Inhibiting SLC13A5 and ACLY: A Novel Mechanism Mediating the Therapeutic Effects of Curcumin on NAFLD. J Agric Food Chem 2021;69:8714–8725.

97.	Li Z, Li D, Choi EY, Lapidus R, Zhang L, Huang SM, Shapiro P, et al. Silencing of solute carrier family 13 member 5 disrupts energy homeostasis and inhibits proliferation of human hepatocarcinoma cells. J Biol Chem 2017;292:13890–13901.

98.	Brieger A, Adryan B, Wolpert F, Passmann S, Zeuzem S, Trojan J. Cytoskeletal scaffolding proteins interact with Lynch-Syndrome associated mismatch repair protein MLH1. Proteomics 2010;10:3343–3355.

99.	Wu ZH, Li C, Zhang YJ, Lin R. Bioinformatics Study Revealed Significance of Exosome Transcriptome in Hepatocellular Carcinoma Diagnosis. Front Cell Dev Biol 2022;10:813701.

100.	Ryaboshapkina M, Hammar M. Human hepatic gene expression signature of non-alcoholic fatty liver disease progression, a meta-analysis. Sci Rep 2017;7:12361.

101.	Zhao R, Guo Z, Lu K, Chen Q, Riaz F, Zhou Y, Yang L, et al. Hepatocyte-specific NR5A2 deficiency induces pyroptosis and exacerbates non-alcoholic steatohepatitis by downregulating ALDH1B1 expression. Cell Death Dis 2024;15:770.

102.	Miranda DA, Krause WC, Cazenave-Gassiot A, Suzawa M, Escusa H, Foo JC, Shihadih DS, et al. LRH-1 regulates hepatic lipid homeostasis and maintains arachidonoyl phospholipid pools critical for phospholipid diversity. JCI Insight 2018;3.

103.	Wagner M, Choi S, Panzitt K, Mamrosh JL, Lee JM, Zaufel A, Xiao R, et al. Liver receptor homolog-1 is a critical determinant of methyl-pool metabolism. Hepatology 2016;63:95–106.

104.	Nishimagi A, Kobayashi M, Sugimoto K, Kofunato Y, Sato N, Haga J, Ishigame T, et al. Aberrant phosphorylation of human LRH1 at serine 510 is predictable of hepatocellular carcinoma recurrence. Clin Exp Med 2023;23:4985–4995.

105.	Joo MS, Koo JH, Kim TH, Kim YS, Kim SG. LRH1-driven transcription factor circuitry for hepatocyte identity: Super-enhancer cistromic analysis. EBioMedicine 2019;40:488–503.

106.	Pang YY, Li JD, Gao L, Yang X, Dang YW, Lai ZF, Liu LM, et al. The clinical value and potential molecular mechanism of the downregulation of MAOA in hepatocellular carcinoma tissues. Cancer Med 2020;9:8004–8019.

107.	Yang JH, Byeon EH, Kang D, Hong SG, Yang J, Kim DR, Yun SP, et al. Fermented Soybean Paste Attenuates Biogenic Amine-Induced Liver Damage in Obese Mice. Cells 2023;12.

108.	Rao VL, Butterworth RF. Alterations of [3H]8-OH-DPAT and [3H]ketanserin binding sites in autopsied brain tissue from cirrhotic patients with hepatic encephalopathy. Neurosci Lett 1994;182:69–72.

109.	Rao VL, Giguere JF, Layrargues GP, Butterworth RF. Increased activities of MAOA and MAOB in autopsied brain tissue from cirrhotic patients with hepatic encephalopathy. Brain Res 1993;621:349–352.

110.	Ko YK, Kim H, Lee Y, Lee YS, Gim JA. DNA Methylation Patterns According to Fatty Liver Index and Longitudinal Changes from the Korean Genome and Epidemiology Study (KoGES). Curr Issues Mol Biol 2022;44:1149–1168.

111.	Zhou F, Kang Q, Ma J, Cai J, Chen Y, Qu K, Li F. Integrated analysis of RNA-seq in hepatocellular carcinoma reveals competing endogenous RNA network composed of circRNA, lncRNA, and mRNA. Medicine (Baltimore) 2023;102:e32915.

112.	Uhlen M, Karlsson MJ, Hober A, Svensson AS, Scheffel J, Kotol D, Zhong W, et al. The human secretome. Sci Signal 2019;12.
